# TfOH-Promoted Reaction of 2,4-Diaryl-1,1,1-Trifluorobut-3-yn-2-oles with Arenes: Synthesis of 1,3-Diaryl-1-CF_3_-Indenes and Versatility of the Reaction Mechanisms

**DOI:** 10.3390/molecules23123079

**Published:** 2018-11-25

**Authors:** Aleksey V. Zerov, Anna N. Kazakova, Irina A. Boyarskaya, Taras L. Panikorovskii, Vitalii V. Suslonov, Olesya V. Khoroshilova, Aleksander V. Vasilyev

**Affiliations:** 1 Department of Organic Chemistry, Institute of Chemistry, Saint Petersburg State University, Universitetskaya nab., 7/9, Saint Petersburg 199034, Russia; lfdse@mail.ru (A.V.Z.); a.kazakova0@gmail.com (A.N.K.); iralbo@yahoo.com (I.A.B.); 2 Department of Crystallography, Institute of Earth Sciences, Saint Petersburg State University, Universitetskaya nab., 7/9, Saint Petersburg 199034, Russia; taras.panikorovsky@spbu.ru; 3 Center for X-ray Diffraction Studies, Research park, St. Petersburg State University, Universitetskiy pr. 26, Saint Petersburg, Petrodvoretz198504, Russia; v.suslonov@spbu.ru (V.V.S.); o_khorosh@mail.ru (O.V.K.); 4 Department of Chemistry, Saint Petersburg State Forest Technical University, Institutsky per., 5, Saint Petersburg 194021, Russia

**Keywords:** trifluoromethyl propargyl alcohols, trifluoromethyl indenes, triflic acid, propargyl cations, cationic reaction mechanism

## Abstract

The TfOH-mediated reactions of 2,4-diaryl-1,1,1-trifluorobut-3-yn-2-oles (CF_3_-substituted diaryl propargyl alcohols) with arenes in CH_2_Cl_2_ afford 1,3-diaryl-1-CF_3_-indenes in yields up to 84%. This new process for synthesis of such CF_3_-indenes is complete at room temperature within one hour. The synthetic potential, scope, and limitations of this reaction were illustrated by more than 70 examples. The proposed reaction mechanism invokes the formation of highly reactive CF_3_-propargyl cation intermediates that can be trapped at the two mesomeric positions by the intermolecular nucleophilic attack of an arene partner with a subsequent intramolecular ring closure.

## 1. Introduction

Acetylene compounds are of great importance for chemistry, biology, medicine, materials science, and other fields of science and technology [1,2,3,4,5,6,7,8,9,10,11]. Fluorinated acetylene derivatives are useful building blocks in organic synthesis for the preparation of new substances and materials with valuable practical properties. The presence of fluorine atoms in organic compounds gives the compounds unique characteristics, such as high lipophilicity and biological activity, heat resistance, nonlinear optical and liquid crystal properties, and so forth [12,13,14,15,16]. Synthesis of new organofluorine derivatives is an actual goal of modern organic chemistry. 

Among the variety of acetylene compounds, propargyl alcohols play an important role in the synthesis of miscellaneous substances. For instance, they have been widely used in Friedel-Crafts alkylation catalyzed by Brønsted [17,18,19,20,21,22,23] or Lewis [24,25,26,27,28,29,30,31,32,33,34,35] acids. However, reactions of trifloromethyl-substituted propargyl alcohols in electrophilic media have not been studied yet.

Based on our work on the electrophilic activation of unsaturated compounds (alkynes, alkenes, allenes) [36], we undertook a special study on the transformation of trifluoromethyl-substituted propargyl alcohols. The main goal of this work was to investigate reactions of 2,4-diaryl-1,1,1-trifluorobut-3-yn-2-oles (CF_3_-propargyl alcohols) with arenes under the action of various Brønsted and Lewis acids.

**Figure 1:**
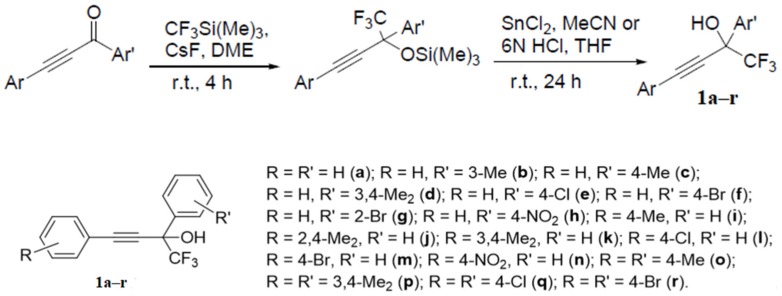
Starting CF_3_-propargyl alcohols used in this study.

The starting diaryl-substituted CF_3_-propargyl alcohols **1a**–**r** bearing various substituents in aromatic rings are shown in Figure 1. They were obtained from the corresponding 1,3-diarylpropynones by trifluoromethylation-*O*-trimethylsilylation of the carbonyl group followed by a desilylation stage (see synthetic procedures in the Supplementary Materials). 

## 2. Results and Discussion

**Scheme 1:**
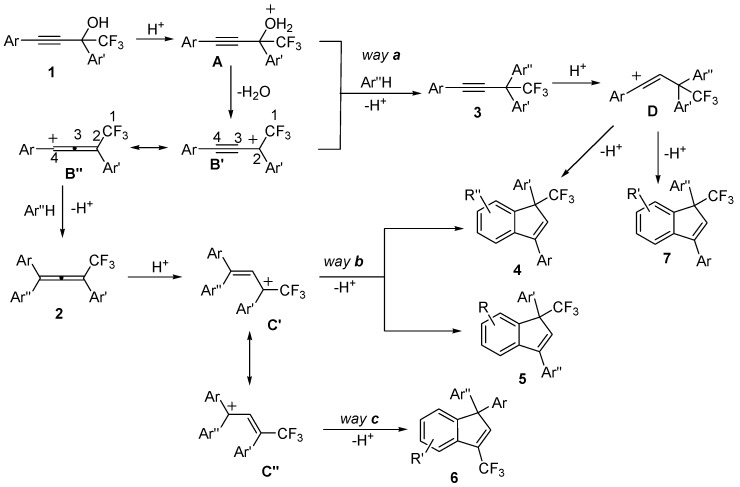
Plausible mechanisms of acid-promoted reactions of CF_3_-alcohols **1** with arenes.

One may propose several ways of conducting transformations of alcohols **1** in acidic media (Scheme 1). First, the protonation of the hydroxyl group takes place with the formation of cation **A**. Elimination of water from it gives the propargyl cation **B**, which may be presented as two mesomeric forms, **B′**↔**B″**, having two electrophilic reactive centers on carbons C^2^ and C^4^, respectively. Species **A** and **B′**, with their electrophilic center on carbon C^2^, may react with the arene, Ar″H, leading to alkyne **3** (*way **a***). Protonation of the latter gives rise to the vinyl cation **D**, which may undergo cyclization into the aryl groups Ar**′** or Ar″, with the formation of indenes **4** or **7**, respectively. 

Another reaction pathway is the reaction of the arene with species **B″** onto its electrophilic carbon C^4^, which affords allene **2**. Protonation of the latter gives the mesomeric allyl cation **C′**↔**C″**. Species **C**′ may be cyclized into both rings Ar″ and Ar, leading to indenes **4** and **5**, respectively (*way **b***). One more possible pathway for this allyl cation is cyclization through its resonance form **C″**, giving rise to indene **6** (*way **c***). 

To estimate the electronic characteristics of the initial intermediates **A** and **B** of these reactions, DFT (density functional theory) calculations of species **Aa** and **Ba** (**B′a**↔**B″a**) derived at the protonation of alcohol **1a** were carried out (Table 1). Energies of the HOMO (highest occupied molecular orbital) and LUMO (lowest unoccupied molecular orbital), charge distribution, the contribution of the atomic orbitals into the molecular orbital, and the global electrophilicity index ω [37,38] were calculated. The calculations show that species **Ba** should be a rather active electrophile, since it is characterized by a large value of the electrophilicity index ω, of 7.59 e, compared to species **Aa**, with ω of 3.92 e. The cation **Aa** has a large positive charge of 1.00 e on carbon C^2^. This carbon gives a large contribution into the LUMO of 13.2%. This proves that carbon C^2^ in the species **Aa** is an electrophilic reactive center according to both charge and orbital factors. 

Contrary to that, the cation **Ba** has a larger positive charge, of 0.23, on carbon C^4^. However, carbon C^2^ gives a larger contribution into the LUMO, of 28.5%. This suggests that in this species, the electrophilic reactivity of the atom C^4^ is ruled under charge control, but the reactivity of the atom C^2^ may be explained by orbital control.

Thus, there are three main pathways, **a**, **b**, and **c**, for the reactions of CF_3_-propargyl alcohols with arenes, proceeding through various cationic intermediates which may lead to various CF_3_-indenes (Scheme 1). A key point in this reaction mechanism is a possible dual reactivity of propargyl cations **B** (**B′**↔**B″**), which may finally lead to different indene structures.

To determine the dependence of the reaction pathway on the substituents in the aromatic rings in alcohols **1** and arenes, starting substrates containing various donor and acceptor substituents in aryl moieties were investigated in these reactions.

First, we conducted reactions of alcohol **1a** with benzene under the action of different Brønsted and Lewis acids (Table 2). In all cases, indene **4aa** was obtained. However, a better result with the highest yield of **4aa** was achieved for the reaction with the use of 1.5 equivalents of trifluoromethanesulfonic acid СF_3_SO_3_H (triflic acid, TfOH) at room temperature for 1 h (entry 3, Table 2).

**Scheme 2:**
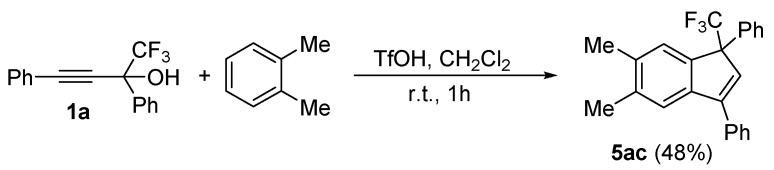
TfOH-promoted reaction of **1** with *o*-xylene in TfOH; reaction conditions: TfOH, CH_2_Cl_2_, molar ratio of **1**:benzene:TfOH = 1:1.1:1.5, room temperature, 1 h.

**Figure 2:**
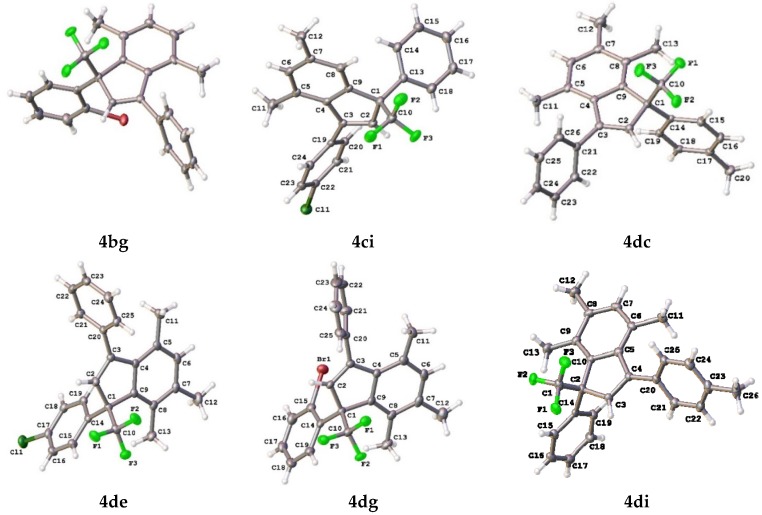

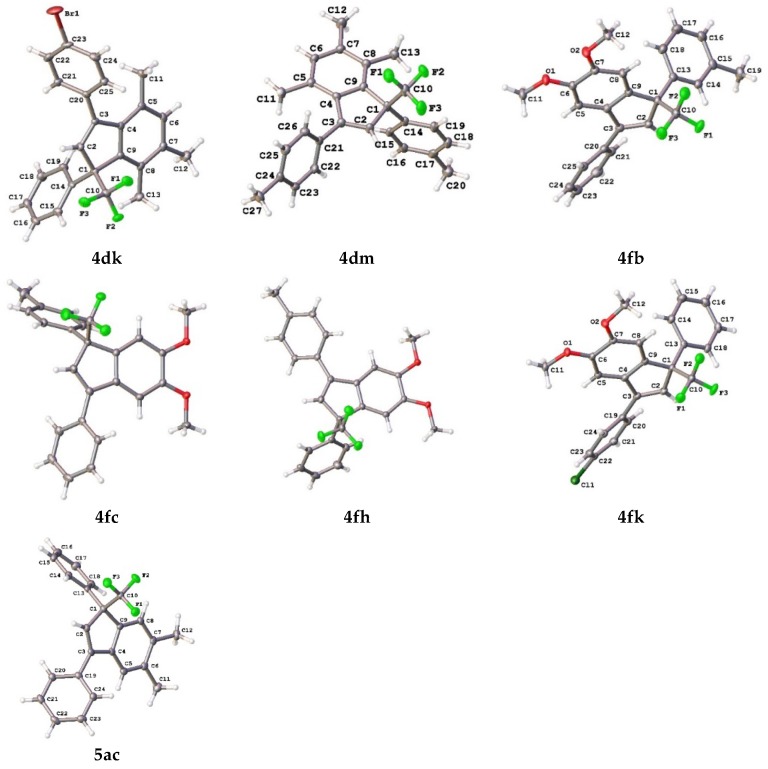
X-ray crystal structures of compounds **4bg** (CCDC 1568593), **4ci** (CCDC 1578216), **4dc** (CCDC 1568602), **4de** (CCDC 1568599), **4dg** (CCDC 1568594), **4di** (CCDC 1563374), **4dk** (CCDC 1568596), **4dm** (CCDC 1568600), **4fb** (CCDC 1568595), **4fc** (CCDC 1568597), **4fh** (CCDC 1568603), **4fk** (CCDC 1568598), and **5ac** (CCDC 1568601) (ellipsoid contour of probability levels is 50%), Green sticks are fluorine atoms.

Maintaining these conditions (1.5 equiv. of TfOH, r.t., 1 h), we conducted reactions of other alcohols **1** with various arenes, benzene (Table 3), ortho-xylene (Scheme 2), para-xylene (Table 4), meta-xylene (Table 5), pseudocumene (1,2,4-trimethylbenzene, Table 6), and veratrole (1,2-dimethoxybenzene, Table 7). These reactions led to compounds **3**, **4**, **5**, and **6**. Structures of these substances were determined by means of ^1^H, ^13^C, and ^19^F-NMR, HRMS, and X-ray single crystal structure analysis (see Figure 2).

In principle, the structures of the target indenes **4**, **5**, and **6** reveal the reaction pathway of their formation (ways **a**, **b**, **c** in Scheme 1) and key intermediates of these transformations (**A**, **B**, **C**, and **D** in Scheme 1). These data are shown in Table 3, Table 4, Table 5, Table 6 and Table 7 for every reaction. In some cases, it is not possible to unequivocally distinguish the reaction pathways based only on the structures of the compounds obtained. However, many reactions clearly point out the mechanism of the formation of the final products.

The data in Table 3 show that for alcohols **1** having phenyl or aryl rings with acceptor groups at the acetylene bond, the only reaction products are indenes of the general structure **4a**, obtained as a result of cyclization into a phenyl ring (entries 1–3, 5–8, 12–14, 17, and 18). These compounds may be formed via *pathway* **a** or **b** (Scheme 1). 

Alcohols **1** bearing donor methyl groups in the aryl substituent at the triple bond react with benzene to form a mixture of indenes of types of **4a** and **5a**. The latter is the main reaction product (entries 9–11). Compounds **5a** are formed at the cyclization into the electron-rich aryl ring (not into the phenyl one) at carbon C^4^ in cations **C** (*way* **a**, Scheme 1).

Alcohol **1d**, with a 3,4-dimethylphenyl ring at carbon C^2^, additionally gave indenes **6a** and **6b**, which were formed by *way* **c** only (see Scheme 1). 

Alcohol **1a** in reaction with *o*-xylene afforded indene **5ac** (Scheme 2). Again, one may propose two possible directions for the formation of this compound: *way* **a** or **b** (see Scheme 1).

In almost all cases for the reactions of alcohols **1** with *p*-xylene, indenes of the general structure **4b** were obtained (Table 4). These compounds may be formed by *way* **a** through the vinyl cation **D** or *way* **b** through the cation **C**′ (Scheme 1).

**Scheme 3:**
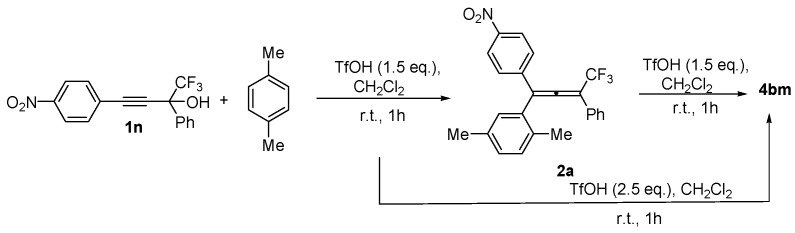
TfOH-promoted reaction of **1n** with *p*-xylene in TfOH.

Additional proof for the proceeding of the reaction of alcohol **1n** with *p*-xylene in *way* **b** was the isolation of allene **2a**, which then was transformed into indene **4bm** in TfOH (Scheme 3). 

Based on the structure of the reaction products **4c** and **5c** obtained from alcohols **1** and *m*-xylene (Table 5), one may assume that in all cases, the *m*-xylene molecule is attacked by the electrophilic center C^4^ of species **B″**, which leads to the formation of the corresponding indenes in *way* **b** (Scheme 1). The presence of electron-withdrawing substituents in the aryl ring at the atom C^4^ prevents electrophilic substitution into this ring, and only indenes **4ci**–**4cm** were isolated (entries 10–12, 15, 16). 

Reactions of alcohols **1** with electron-rich pseudocumene afforded two types of indene structures, **4d** and **4e**, formed by electrophilic substitution onto the pseudocumene moiety only (Table 6). Taking into account that the most active position for electrophilic attack in the pseudocumene molecule is the atom C^5^ and that the first reaction occurs in this particular position, one may propose that indenes **4da**–**do** are formed in *way* **b** through cations **B″** and **C′**, and indenes **4ea**–**4eo** in *way* **a** through cations **A** (or **B′**) and **D** (see Scheme 1). The structures of compounds **4d** and **4e** and positions of the methyl groups in the indene core were determined by H,H and H,F NOESY correlations between the methyl substituents, CF_3_ group, and aromatic indene protons (see the Supporting Information). 

Surprisingly, reactions of alcohols **1** with veratrole yielded mixtures of alkyne **3** and indene **4f**. Moreover, treatment of alkyne **3** with TfOH (1.5 eq.) in CH_2_Cl_2_ at room temperature for 1 h gave indene **4f**. This data unambiguously proves that reactions with veratrole proceed in *way* **a** with the participation of cations **A** (or **B′**) and **D** (see Scheme 1).

Summarizing the data obtained on the TfOH-promoted reactions of CF_3_-propargyl alcohols **1** with different arenes, leading to CF_3_-indenes (Table 3, Table 4, Table 5, Table 6 and Table 7), one may conclude that these indenes may be formed in several reaction pathways (Scheme 1), depending on the structures of the starting alcohol **1** and the nucleophilicity of the arene. Key intermediates of these reactions are o-protonated forms **A** of the alcohols and the mesomeric propargyl cations **B** (**B′**↔**B″**) generated from alcohols **1** in acidic media (see Scheme 1). Most probably, reactions with electron-rich arenes, pseudocumene (Table 6), and veratrole (Table 7) may proceed through cations **A** (*way* **a** in Scheme 1), which are sufficiently electrophilic (see data on DFT calculations in Table 2) to react with such donating arenes. Reactions with other less nucleophilic arenes, benzene, and xylenes (Table 3, Table 4 and Table 5) may go both in *way* **a** and **b** (Scheme 1) due to the dual reactivity of the propargyl cation **B**. However, *way* **b** through the allenyl resonance form **B″** may be more preferable; see the reactions with *m*-xylene that proceed mainly in this way (Table 5). Construction of the indene core at the final stages of the reaction depends on the nucleophilicity of the aryl rings Ar, Ar′, and Ar″ in the intermediate species **C** and **D**. Electrophilic cyclization takes place in the more-donating aromatic moiety.

It should be noted that many of the reactions studied lead to the exclusive formation of only one of CF_3_-indene **4** or **5** in good yields. Such CF_3_-indenes are rather rare substrates, and there are only a few reports on their synthesis [39,40,41,42,43,44].

## 3. Conclusions

We have studied, for the first time, reactions of diaryl-substituted CF_3_-propargyl alcohols with arenes under the action of the superacid TfOH. The reaction proceeds through the intermediate formation of several cationic species, which finally lead to the formation of the synthetically hardly available 1,3-diaryl-1-CF_3_-indenes. 

## Figures and Tables

**Table 1:** Selected electronic characteristics (DFT calculations) of cations **Aa** and **Ba** (**B′a**↔**B″a**) derived at the protonation of alcohol **1a**.

Caption	E_HOMO_, eV	E_LUMO_, eV	ω ^a^, eV	q(C^2^) ^b^, e	q(C^4^) ^b^, e	k(C^2^)_LUMO_^c^, %	k(C^4^)_LUMO_^c^, %
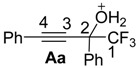	−7.40	−3.57	3.92	1.00	−0.34	13.2	9.4
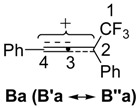	−7.69	−5.03	7.59	0.043	0.23	28.5	19.9

^a^ Global electrophilicity index ω = (E_HOMO_ + E_LUMO_)^2^/8(E_LUMO_−E_HOMO_). ^b^ Natural charges. ^c^ Contribution of the atomic orbital into the molecular orbital.

**Table 2:**
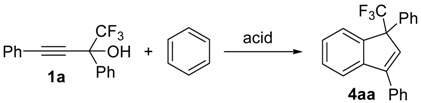
Acid-promoted reaction of **1a** with benzene.

Entry	Reaction Conditions ^a^			Yield of 4aa, ^b^ %
	Acid	Temperature, °C	Time, h	
1	TfOH (50 eq.)	r.t.	1	30
2	TfOH (50 eq.) **^c^**	−35	1	45
3	TfOH (1.5 eq.)	r.t.	1	57
4	FSO_3_H (86 eq.) **^c^**	−75	1	44
5	H_2_SO_4_ (5 eq.)	r.t.	1	40
6	AlCl_3_ (2 eq.)	r.t.	1	33
7	FeCl_3_ (1eq.)	r.t.	1	40
8	BF_3_ × Et_2_O (2 eq.)	r.t.	72	27 **^d^**
9	Sc(OTf)_3_ (0.1 eq.) **^e^**	85	1	42
10	Cu(OTf)_2_ (0.1 eq.) **^e^**	85	1	30

^a^ Reaction conditions: acid, solvent CH_2_Cl_2_, molar ratio of **1**:benzene = 1:50. ^b^ Complete conversion of **1a**. **^c^** Cosolvent was CH_2_Cl_2_. **^d^** Conversion of **1a** was 60%. **^e^** Cosolvent was 1,2-dichloroethane.^r.t.^ room temperature.

**Table 3:**
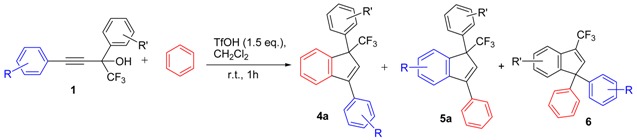
TfOH-promoted reaction of alcohols **1a**–**f** with benzene; reaction conditions: TfOH, CH_2_Cl_2_, molar ratio of **1**:benzene:TfOH = 1:50:1.5, room temperature, 1 h.

Entry	Alcohol	Reaction Products 4a, 5a, and 6 (Yield, %, Ratio of Isomers)	Possible Reaction Way and Intermediates from Scheme 1
1	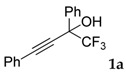	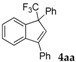 (57%)	*way***a**: **A** (or **B′**), **D** *way* **b**: **B″**, **C′**
2	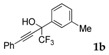	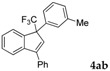 (48%)	*way***b**: **B″**, **C′**
3	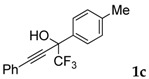	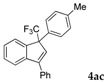 (69%)	*way***b**: **B″**, **C′**
4	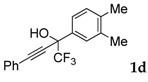	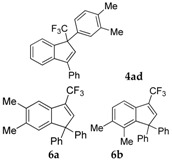 total yield of 47% (**4ad**:**6a**:**6b** = 5:2:1)	4**ad**—*way* **b**: **B″**, **C′** **6a, 6b**—*way* **c**: **B″**, **C″**
5	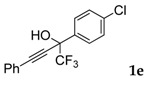	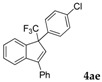 (73%)	*way***a**: **A** (or **B′**), **D** *way* **b**: **B″**, **C′**
6	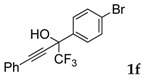	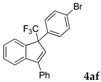 (80%)	*way***a**: **A** (or **B′**), **D** *way* **b**: **B″**, **C′**
7	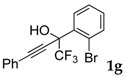	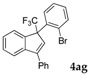 (66%)	*way***a**: **A** (or **B′**), **D** *way* **b**: **B″**, **C′**
8 **^a^**	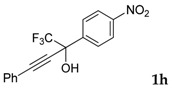	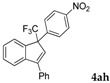 (70%)	*way***a**: **A** (or **B′**), **D** *way* **b**: **B″**, **C′**
9	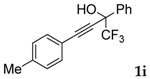	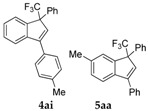 total yield of 72% (**4ai**:**5aa** = 1:3.8)	*way***a**: **A** (or **B′**), **D** *way* **b**: **B″**, **C′**
10	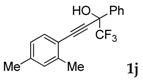	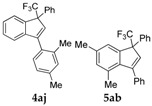 total yield of 47% (**4aj**:**5ab** = 1:7)	*way***a**: **A** (or **B′**), **D** *way* **b**: **B″**, **C′**
11	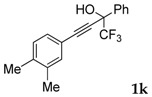	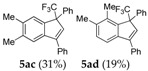	*way***b**: **B″**, **C′**
12	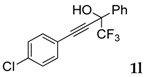	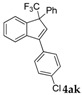 (59%)	*way***a**: **A** (or **B′**), **D** *way* **b**: **B″**, **C′**
13	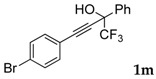	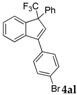 (51%)	*way***a**: **A** (or **B′**), **D** *way* **b**: **B″**, **C′**
14 ^a^	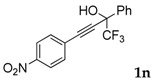	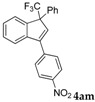 (62%)	*way***a**: **A** (or **B′**), **D** *way* **b**: **B″**, **C′**
15	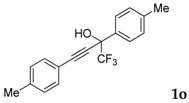	Complex mixture of reaction products	-
16	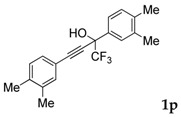	Complex mixture of reaction products	-
17	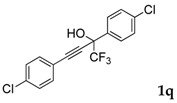	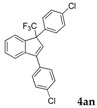 (70%)	*way***a**: **A** (or **B′**), **D** *way* **b**: **B″**, **C′**
18	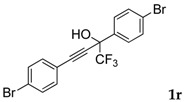	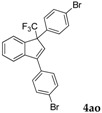 (80%)	*way***a**: **A** (or **B′**), **D** *way* **b**: **B″**, **C′**

^a^ Amount of TfOH was 2.5 equiv.

**Table 4:**
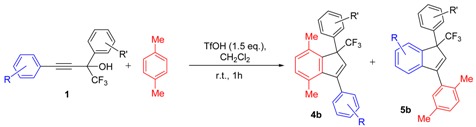
TfOH-promoted reaction of **1** with *p*-xylene; reaction conditions: TfOH, CH_2_Cl_2_, molar ratio of **1**:*p*-xylene:TfOH = 1:1.1:1.5, room temperature, 1 h.

Entry	Alcohol	Reaction Products 4 and 5 (Yield, %, Ratio of Isomers)	Possible Reaction Way and Intermediates from Scheme 1
1	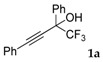	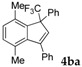 (75%)	*way***a**: **A** (or **B′**), **D** *way* **b**: **B″**, **C′**
2	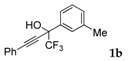	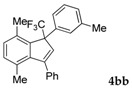 (66%)	*way***b**: **B″**, **C′**
3	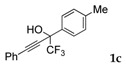	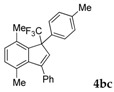 (44%)	*way***a**: **A** (or **B′**), **D** *way* **b**: **B″**, **C′**
4	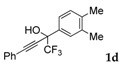	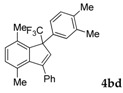 (72%)	*way***b**: **B″**, **C′**
5	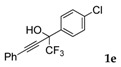	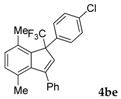 (60%)	*way***a**: **A** (or **B′**), **D** *way* **b**: **B″**, **C′**
6	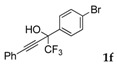	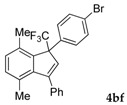 (74%)	*way***a**: **A** (or **B′**), **D** *way* **b**: **B″**, **C′**
7	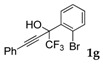	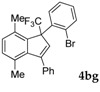 (45%)	*way***a**: **A** (or **B′**), **D** *way* **b**: **B″**, **C′**
8 ^a^	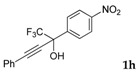	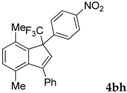 (84%)	*way***a**: **A** (or **B′**), **D** *way* **b**: **B″**, **C′**
9	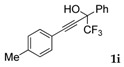	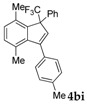 (71%)	*way***a**: **A** (or **B′**), **D** *way* **b**: **B″**, **C′**
10	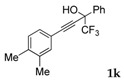	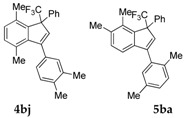 total yield of 28% (**4bj**:**5ba** = 1.5:1) 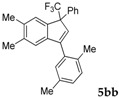 (34%)	*way***b**: **B″**, **C′**
11	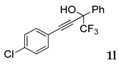	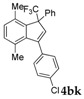 (68%)	*way***a**: **A** (or **B′**), **D** *way* **b**: **B″**, **C′**
12	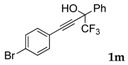	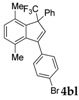 (58%)	*way***a**: **A** (or **B′**), **D** *way* **b**: **B″**, **C′**
13 ^a^	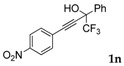	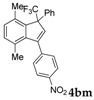 (70%)	*way***a**: **A** (or **B′**), **D** *way* **b**: **B″**, **C′**
14	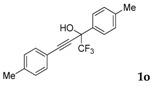	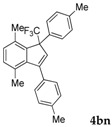 (59%)	*way***a**: **A** (or **B′**), **D** *way* **b**: **B″**, **C′**
15	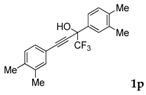	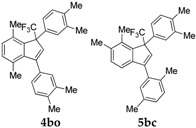 total yield of 25% (**4bo**:**5bc** = 1.4:1) 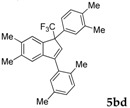 (25%)	*way***b**: **B″**, **C′**
16	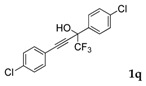	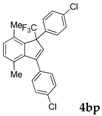 (74%)	*way***a**: **A** (or **B′**), **D** *way* **b**: **B″**, **C′**
17	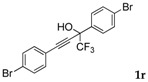	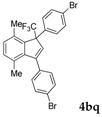 (78%)	*way***a**: **A**, **B**′, **D** *way***b**: **B″**, **C′**

^a^ Amount of TfOH was 2.5 equiv.

**Table 5:**
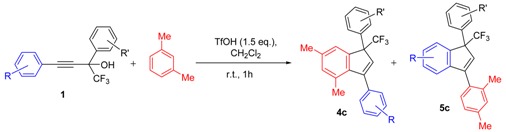
TfOH-promoted reaction of **1** with *m*-xylene; reaction conditions: TfOH, CH_2_Cl_2_, molar ratio of **1**:*m*-xylene:TfOH = 1:1.1:1.5, room temperature, 1 h.

Entry	Alcohol	Reaction Products 4 and 5 (Yield, %, Ratio of Isomers)	Possible Reaction Way and Intermediates from Scheme 1
1	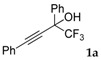	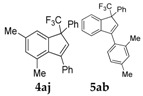 total yield of 63% (**4aj**:**5ab** = 6:1)	*way***b**: **B″**, **C′**
2	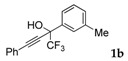	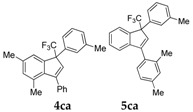 total yield of 66% (**4ca**:**5ca** = 3:1)	*way***b**: **B″**, **C′**
3	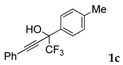	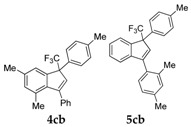 total yield of 69% (**4cb**:**5cb** = 6:1)	*way***b**: **B″**, **C′**
4	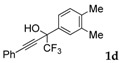	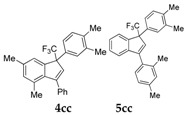 total yield of 69% (**4cc**:**5cc** = 5.7:1)	*way***b**: **B″**, **C′**
5	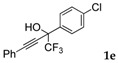	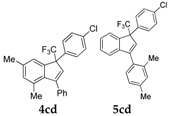 total yield of 60% (**4cd**:**5cd** = 4:1)	*way***b**: **B″**, **C′**
6	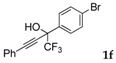	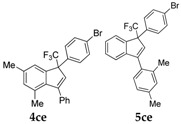 total yield of 56% (**4ce**:**5ce** = 4.9:1)	*way***b**: **B″**, **C′**
7	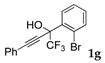	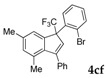 (40%)	*way***b**: **B″**, **C′**
8 ^a^	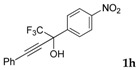	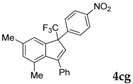 (54%)	*way***b**: **B″**, **C′**
9	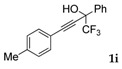	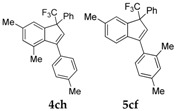 total yield of 56% (**4ch**:**5cf** = 2.8:1)	*way***b**: **B″**, **C′**
10	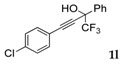	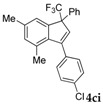 (63%)	*way***b**: **B″**, **C′**
11	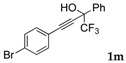	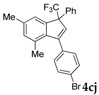 (58%)	*way***b**: **B″**, **C′**
12 ^a^	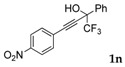	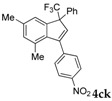 (63%)	*way***b**: **B″**, **C′**
13	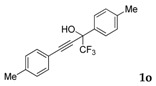	Complex mixture of reaction products	-
14	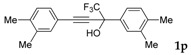	Complex mixture of reaction products	-
15	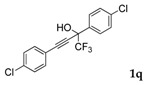	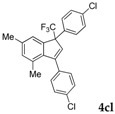 (64%)	*way***b**: **B″**, **C′**
16	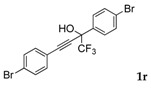	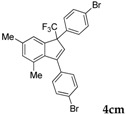 (50%)	*way***b**: **B″**, **C′**

^a^ Amount of TfOH was 2.5 equiv.

**Table 6:**
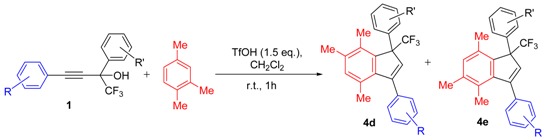
TfOH-promoted reaction of **1** with pseudocumene; reaction conditions: TfOH, CH_2_Cl_2_, molar ratio of **1**:pseudocumene:TfOH = 1:1.1:1.5, room temperature, 1 h.

Entry	Alcohol	Reaction Products 4d and 4e (Yield, %, Ratio of Isomers)	Possible Reaction Way and Intermediates from Scheme 1
1	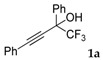	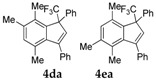 total yield of 75% (**4da**:**4ea** = 11.5:1)	**4da**—*way***b**: **B″**, **C′** **4ea**—*way***a**: **A** (or **B′**), **D**
2	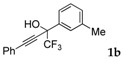	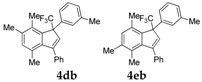 total yield of 69% (**4db**: **4eb** = 11.5:1)	**4db**—*way***b**: **B″**, **C′** **4eb**—*way***a**: **A** (or **B′**), **D**
3	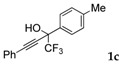	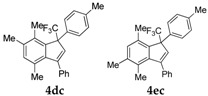 total yield of 50% (**4dc**:**4ec** = 13:1)	**4dc**—*way***b**: **B″**, **C′** **4ec**—*way***a**: **A** (or **B′**), **D**
4	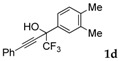	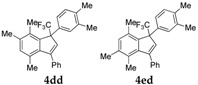 total yield of 67% (**4dd**:**4ed** = 15.7:1)	**4dd**—*way***b**: **B″**, **C′** **4ed**—*way***a**: **A** (or **B′**), **D**
5	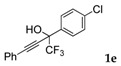	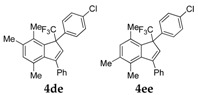 total yield of 66% (**4de**:**4ee** = 11.5:1)	**4de**—*way***b**: **B″**, **C′** **4ee**—*way***a**: **A** (or **B′**), **D**
6	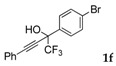	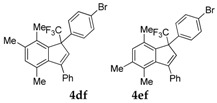 total yield of 80% (**4df**:**4ef** = 24:1)	**4df**—*way***b**: **B″**, **C′** **4ef**—*way***a**: **A** (or **B′**), **D**
7	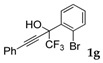	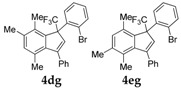 total yield of 70% (**4dg**:**4eg** = 2.7:1)	**4eg**—*way***b**: **B″**, **C′** **4fg**—*way***a**: **A** (or **B′**), **D**
8 ^a^	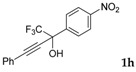	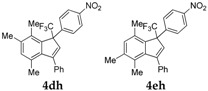 total yield of 58% (**4dh**:**4eh** = 1.8:1)	**4dh**—*way***b**: **B″**, **C′** **4eh**—*way***a**: **A** (or **B′**), **D**
9	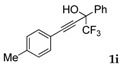	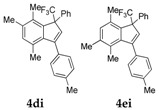 total yield of 51% (**4di**:**4ei** = 12:1)	**4di**—*way***b**: **B″**, **C′** **4ei**—*way***a**: **A** (or **B′**), **D**
10	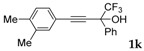	Complex mixture of reaction products	-
11	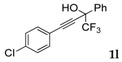	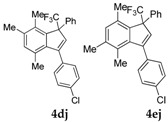 total yield of 54% (**4dj**:**4ej** = 11.5:1)	**4dj**—*way***b**: **B″**, **C′** **4ej**—*way***a**: **A** (or **B′**), **D**
12	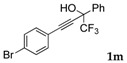	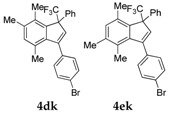 total yield of 49% (**4dk**:**4ek** = 10:1)	**4dk**—*way***b**: **B″**, **C′** **4ek**—*way***a**: **A** (or **B′**), **D**
13	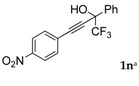	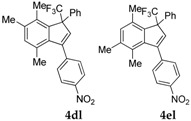 total yield of 66% (**4dl**:**4el** = 13:1)	4**dl**—*way* **b**: **B″**, **C′** **4el**—*way* **a**: **A** (or **B′**), **D**
14	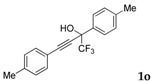	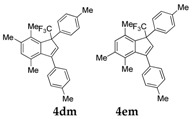 total yield of 65% (**4dm**:**4em** = 6:1)	**4dm**—*way***b**: **B″**, **C′** **4em**—*way***a**: **A** (or **B′**), **D**
15	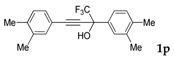	Complex mixture of reaction products	-
16	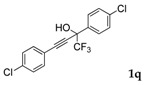	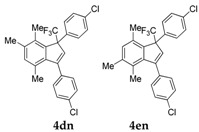 total yield of 49% (**4dn**:**4en** = 10:1)	**4dn**—*way***b**: **B″**, **C′** **4en**—*way***a**: **A** (or **B′**), **D**
17	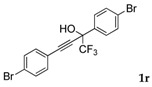	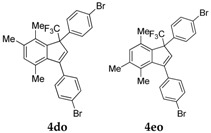 total yield of 57% (**4do**:**4eo** = 11.5:1)	**4do**—*way***b**: **B″**, **C′** **4eo**—*way***a**: **A** (or **B′**), **D**

^a^ Amount of TfOH was 2.5 equiv.

**Table 7:**
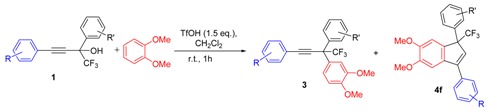
TfOH-promoted reaction of **1** with veratrole; reaction conditions: TfOH, CH_2_Cl_2_, molar ratio of **1**:veratrole:TfOH = 1:1.1:1.5, room temperature, 1 h.

Entry	Alcohol	Reaction Products 4f and 3 (Yield, %, Ratio of Isomers)	Possible Reaction Way and Intermediates from Scheme 1
1 ^a^	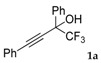	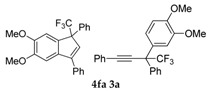 total yield of 60% (**4fa**:**3a** = 2.8:1)	*way***a**: **A** (or **B′**), **D**
2	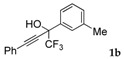	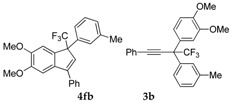 total yield of 49% (**4gb**:**3b** = 3.5:1)	*way***a**: **A** (or **B′**), **D**
3	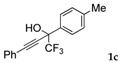	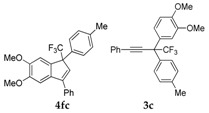 total yield of 98% (**4fc**:**3c** = 4.9:1)	*way***a**: **A** (or **B′**), **D**
4	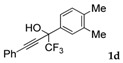	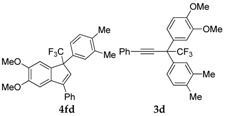 total yield of 76% (**4fd**:**3d** = 5:1)	*way***a**: **A** (or **B′**), **D**
5	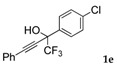	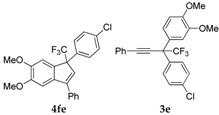 total yield of 44% (**4fe**:**3e** = 6:1)	*way***a**: **A** (or **B′**), **D**
6	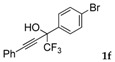	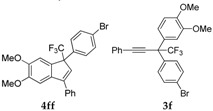 total yield of 70% (**4ff**:**3f** = 4:1)	*way***a**: **A** (or **B′**), **D**
7 ^a^	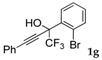	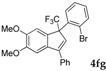 (82%)	*way***a**: **A** (or **B′**), **D**
8	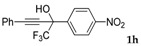	Complex mixture of reaction products	-
9	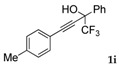	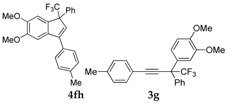 total yield of 54% (**4fh**:**3g** = 13:1)	*way***a**: **A** (or **B′**), **D**
10 ^a^	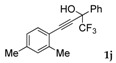	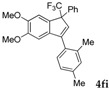 (67%)	*way***a**: **A** (or **B′**), **D**
11 ^a^	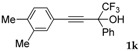	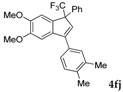 (57%)	*way***a**: **A** (or **B′**), **D**
12	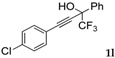	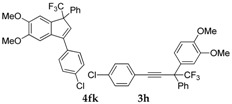 total yield of 65% (**4fk**:**3h** = 10:1)	*way***a**: **A** (or **B′**), **D**
13	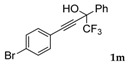	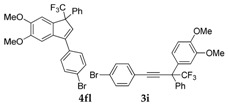 total yield of 36% (**4fl**:**3i** = 7:1)	*way***a**: **A** (or **B′**), **D**
14	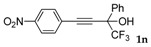	Complex mixture of reaction products	-
15 ^a^	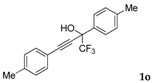	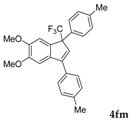 (69%)	*way***a**: **A** (or **B′**), **D**
16	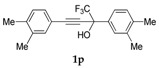	Complex mixture of reaction products	-
17	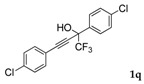 1q	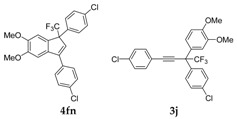 total yield of 50% (**4fn**:**3j** = 2.8:1)	*way***a**: **A** (or **B′**), **D**
18	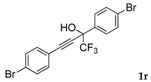	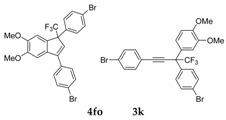 total yield of 59% (**4fo**:**3k** = 2:1)	*way***a**: **A** (or **B′**), **D**

^a^ Amount of TfOH was 2.5 equiv.

## Supplementary Materials

The following are available online. Experimental procedures, characterization of compounds, copies of NMR spectra, and data on DFT calculations.
