# Supplementary material for: TfOH-Promoted Reaction of 2,4-Diaryl-1,1,1-Trifluorobut-3-yn-2-oles with Arenes: Synthesis of 1,3-Diaryl-1-CF3-Indenes and Versatility of the Reaction Mechanisms
Source: Molecules. 2018 Nov 25;23(12):3079. doi: 10.3390/molecules23123079 (PMC6321356; doi:10.3390/molecules23123079)

checkCIF/PLATON report

You have not supplied any structure factors. As a result the full set of tests cannot be run.

THIS REPORT IS FOR GUIDANCE ONLY. IF USED AS PART OF A REVIEW PROCEDURE FOR PUBLICATION, IT SHOULD NOT REPLACE THE EXPERTISE OF AN EXPERIENCED CRYSTALLOGRAPHIC REFEREE.

No syntax errors found. CIF dictionary Interpreting this report

Datablock: AMP13

```
Bond precision:  C-C = 0.0031 A          Wavelength=0.71073

Cell:            a=30.9293(14)     b=10.8472(4)     c=10.0272(4)
                 alpha=90          beta=90          gamma=90
Temperature:     100 K

                Calculated                 Reported
Volume          3364.1(2)                  3364.1(2)
Space group     P b c a                    P b c a
Hall group      -P 2ac 2ab                 -P 2ac 2ab
Moiety formula  C20 H19 N3 O2              C20 H19 N3 O2
Sum formula     C20 H19 N3 O2              C20 H19 N3 O2
Mr              333.38                     333.38
Dx,g cm-3       1.316                      1.316
Z               8                          8
Mu (mm-1)       0.087                      0.087
F000            1408.0                     1408.0
F000'           1408.57
h,k,lmax        40,14,13                   40,14,13
Nref            3858                       3856
Tmin,Tmax       0.979,0.983                0.969,1.000
Tmin'           0.974

Correction method= # Reported T Limits: Tmin=0.969 Tmax=1.000
AbsCorr = MULTI-SCAN

Data completeness= 0.999          Theta(max)= 27.499

R(reflections)= 0.0620( 3308)     wR2(reflections)= 0.1227( 3856)

S = 1.271                  Npar= 227
```

The following ALERTS were generated. Each ALERT has the format
test-name_ALERT_alert-type_alert-level.
Click on the hyperlinks for more details of the test.

Alert level G

```
PLAT007_ALERT_5_G Number of Unrefined Donor-H Atoms ..............          1 Report
PLAT012_ALERT_1_G No     _shelx_res_checksum found in CIF ........     Please Check
PLAT395_ALERT_2_G Deviating  X-O-Y  Angle from 120 Deg for  O1          103.7 Degree
PLAT793_ALERT_4_G The Model has Chirality at C3      (Centro SPGR)          S Verify
PLAT793_ALERT_4_G The Model has Chirality at C5      (Centro SPGR)          R Verify
```

```
   0 ALERT level A = Most likely a serious problem - resolve or explain
   0 ALERT level B = A potentially serious problem, consider carefully
   0 ALERT level C = Check. Ensure it is not caused by an omission or oversight
   5 ALERT level G = General information/check it is not something unexpected

   1 ALERT type 1 CIF construction/syntax error, inconsistent or missing data
   1 ALERT type 2 Indicator that the structure model may be wrong or deficient
   0 ALERT type 3 Indicator that the structure quality may be low
   2 ALERT type 4 Improvement, methodology, query or suggestion
   1 ALERT type 5 Informative message, check
```

It is advisable to attempt to resolve as many as possible of the alerts in all categories. Often the minor alerts point to easily fixed oversights, errors and omissions in your CIF or refinement strategy, so attention to these fine details can be worthwhile. In order to resolve some of the more serious problems it may be necessary to carry out additional measurements or structure refinements. However, the purpose of your study may justify the reported deviations and the more serious of these should normally be commented upon in the discussion or experimental section of a paper or in the "special_details" fields of the CIF. checkCIF was carefully designed to identify outliers and unusual parameters, but every test has its limitations and alerts that are not important in a particular case may appear. Conversely, the absence of alerts does not guarantee there are no aspects of the results needing attention. It is up to the individual to critically assess their own results and, if necessary, seek expert advice.

Publication of your CIF in IUCr journals

A basic structural check has been run on your CIF. These basic checks will be run on all CIFs submitted for publication in IUCr journals (*Acta Crystallographica*, *Journal of Applied Crystallography*, *Journal of Synchrotron Radiation*); however, if you intend to submit to *Acta Crystallographica Section C* or *E* or *IUCrData*, you should make sure that full publication checks are run on the final version of your CIF prior to submission.

Publication of your CIF in other journals

Please refer to the *Notes for Authors* of the relevant journal for any special instructions relating to CIF submission.

PLATON version of 27/03/2017; check.def file version of 24/03/2017

Datablock AMP13 - ellipsoid plot

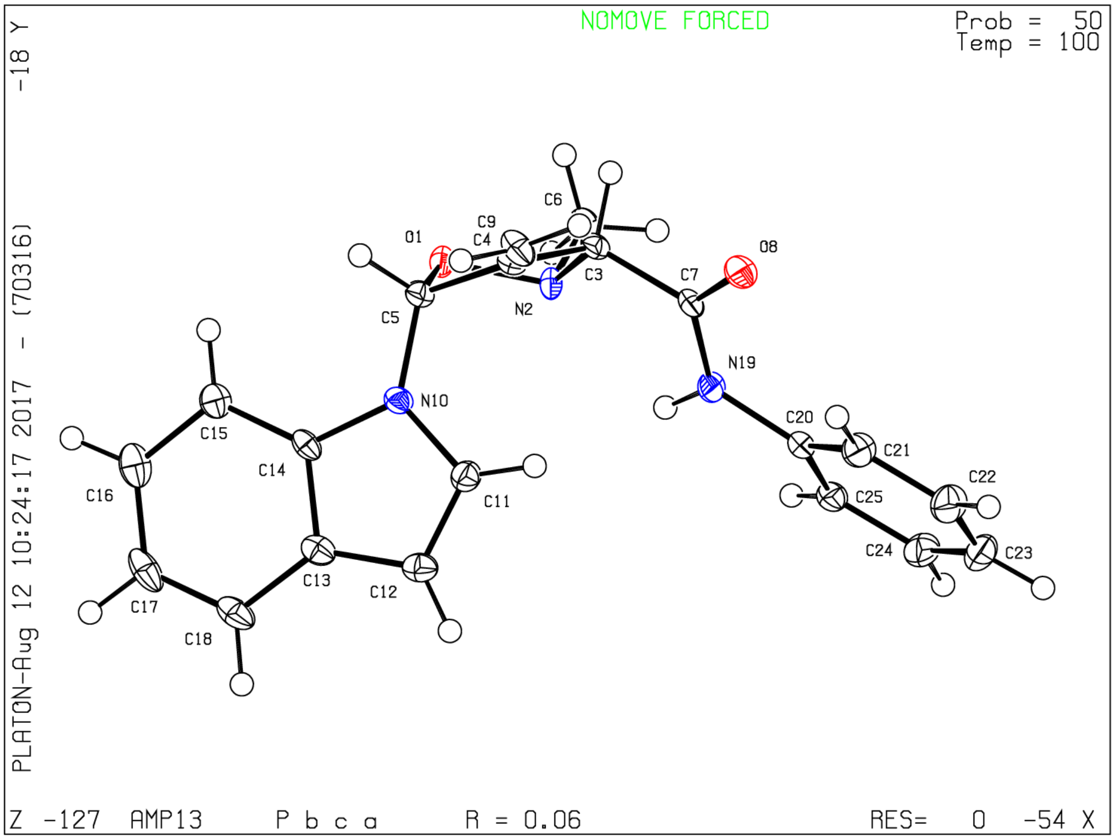

Supplement: Supplementary file 1 [file molecules-23-03079-s001.zip › supplyment/checkcif 4fh.pdf]
