# Supplementary material for: TfOH-Promoted Reaction of 2,4-Diaryl-1,1,1-Trifluorobut-3-yn-2-oles with Arenes: Synthesis of 1,3-Diaryl-1-CF3-Indenes and Versatility of the Reaction Mechanisms
Source: Molecules. 2018 Nov 25;23(12):3079. doi: 10.3390/molecules23123079 (PMC6321356; doi:10.3390/molecules23123079)
Supplement: Supplementary file 1 [file molecules-23-03079-s001.zip › supplyment/SI part 2-edited.docx]

**Original-NMR spectra (^1^H, ^13^C, DEPT, ^19^F, NOESY-HH and NOESY-HF)**


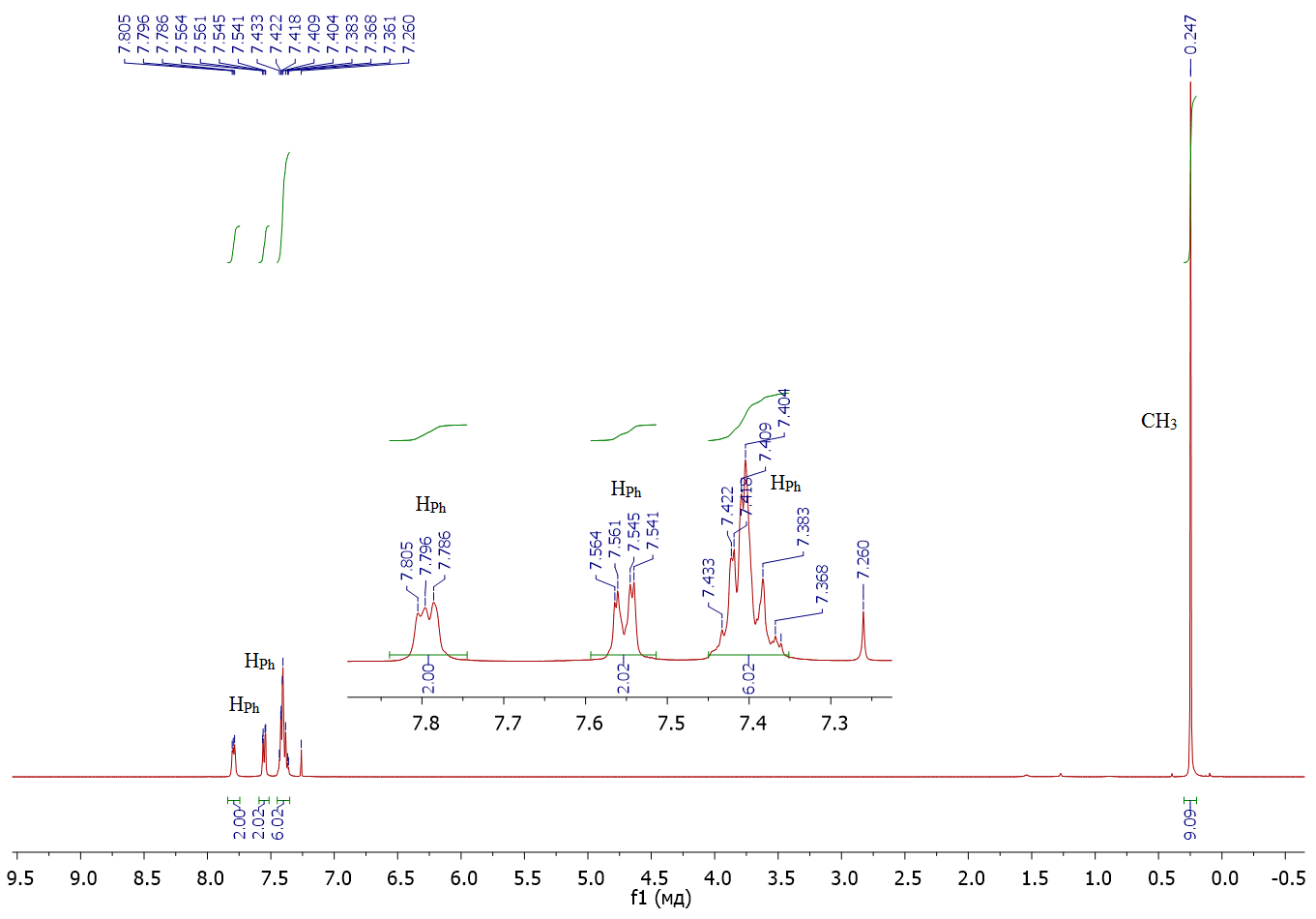


Figure S1. ^1^Н-NMR spectrum of the compound **Ia** (CDCl_3_, 400 MHz).


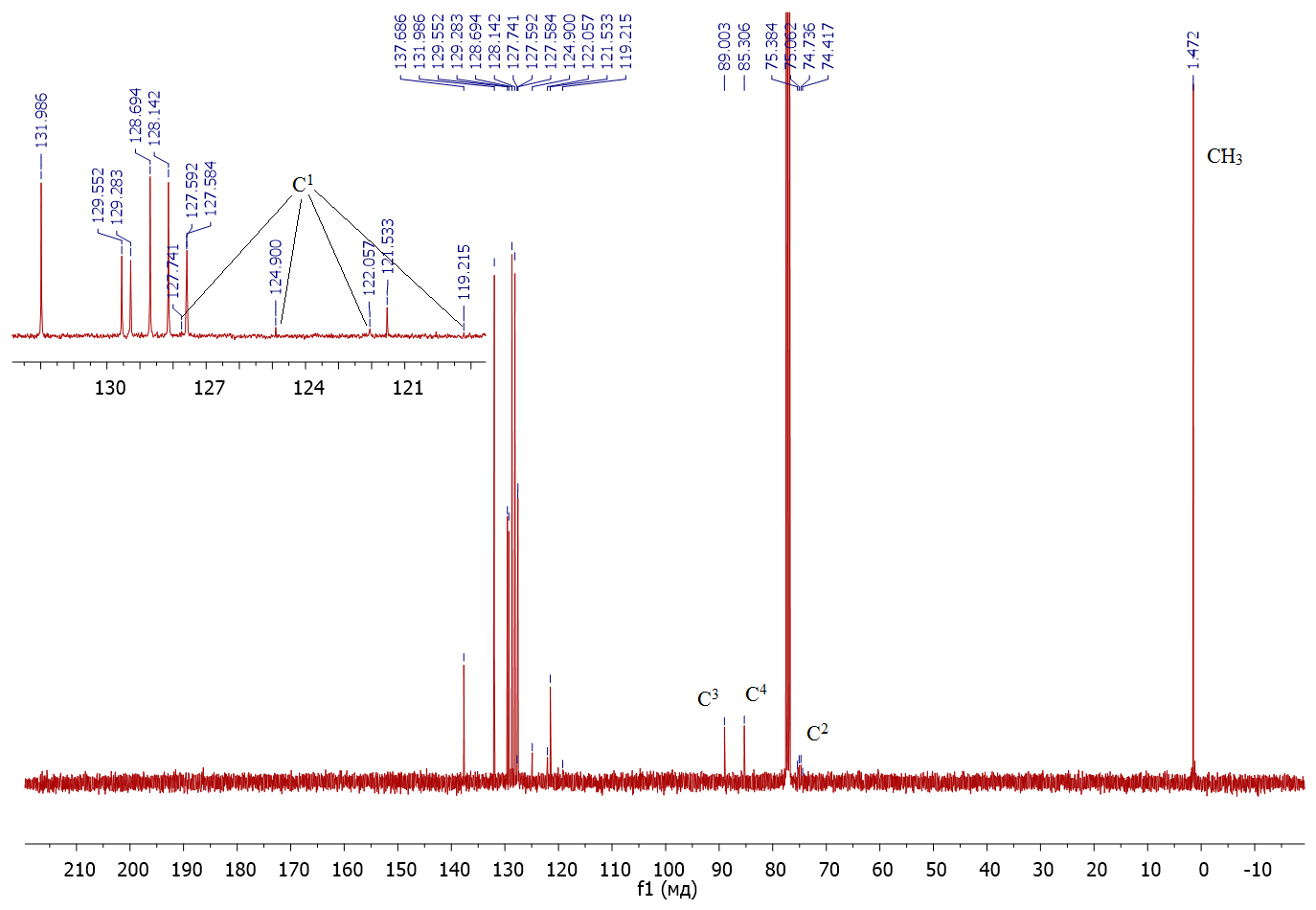


Figure S2. ^13^C-NMR spectrum of the compound **Ia** (CDCl_3_, 100 MHz).


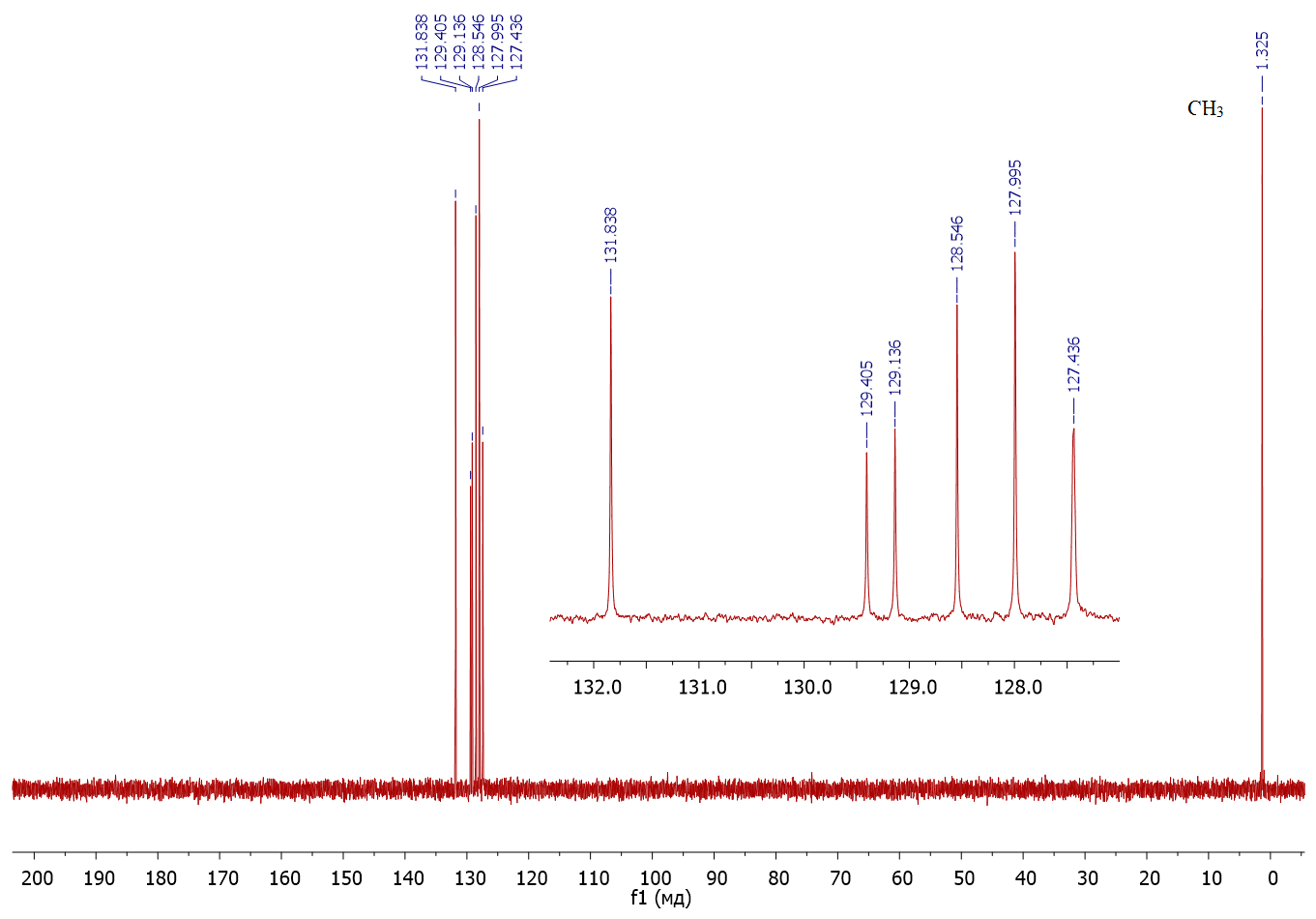


Figure S3. DEPT spectrum of the compound **Ia** (CDCl_3_, 100 MHz).


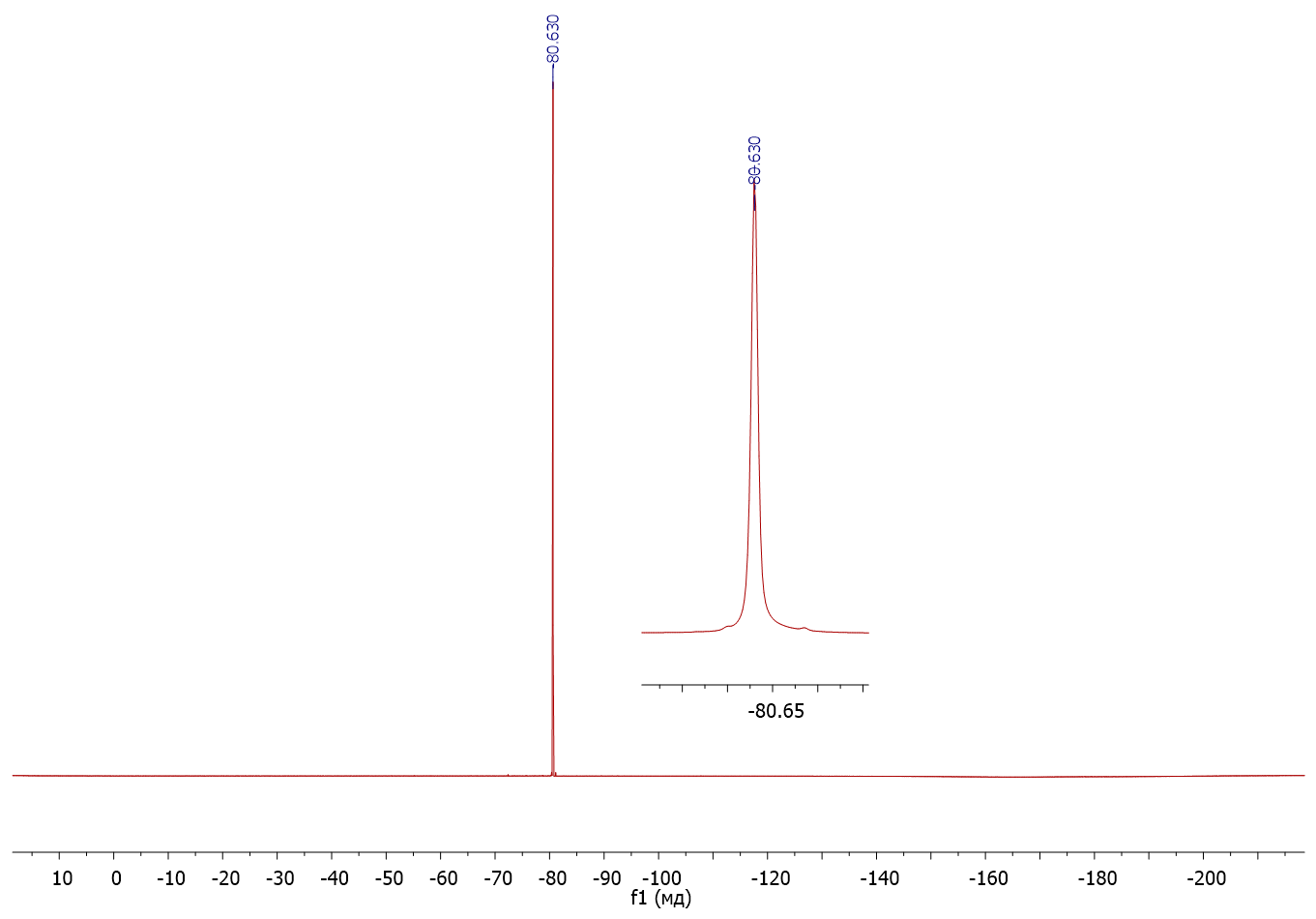

Figure S4. ^19^F-NMR spectrum of the compound **Ia** (CDCl_3_, 376 MHz).


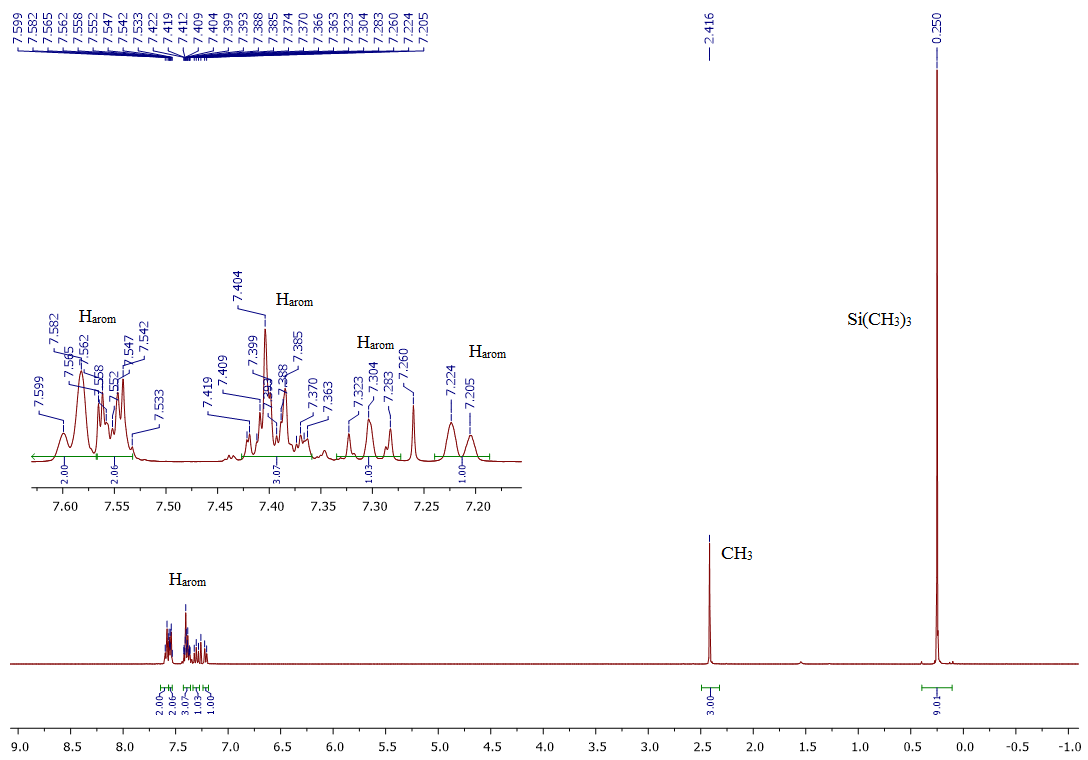


Figure S5. ^1^Н-NMR spectrum of the compound **Ib** (CDCl_3_, 400 MHz).


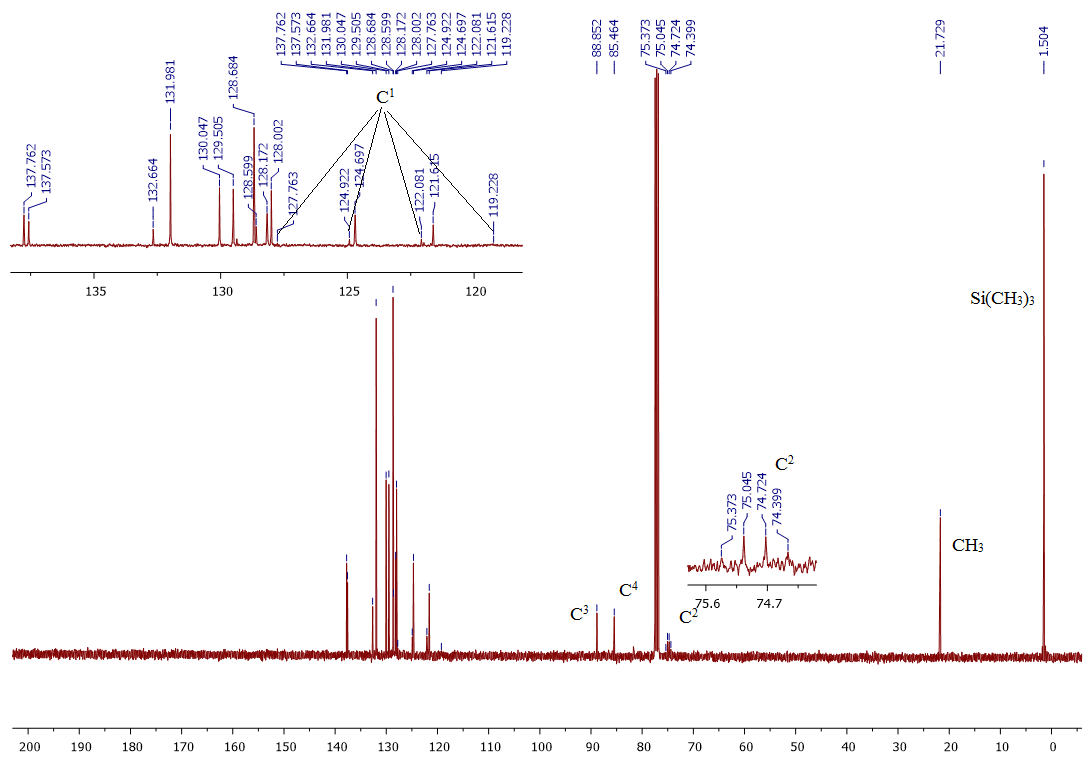


Figure S6. ^13^C-NMR spectrum of the compound **Ib** (CDCl_3_, 100 MHz).


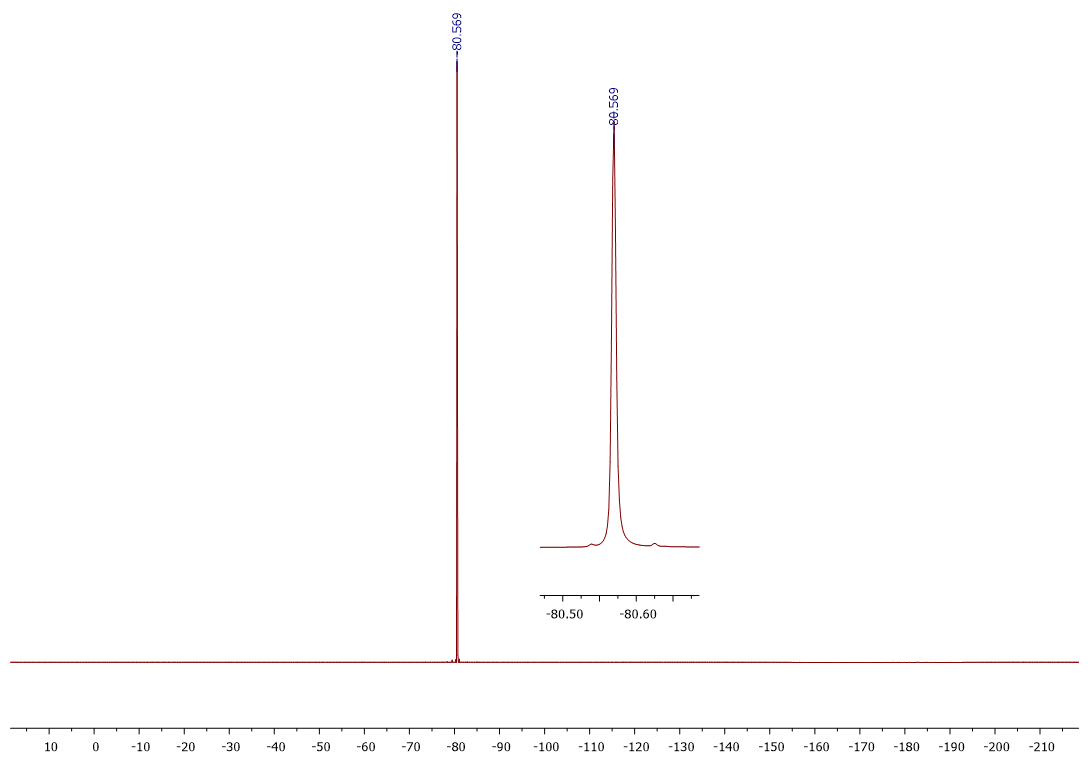


Figure S7. ^19^F-NMR spectrum of the compound **Ib** (CDCl_3_, 376 MHz).


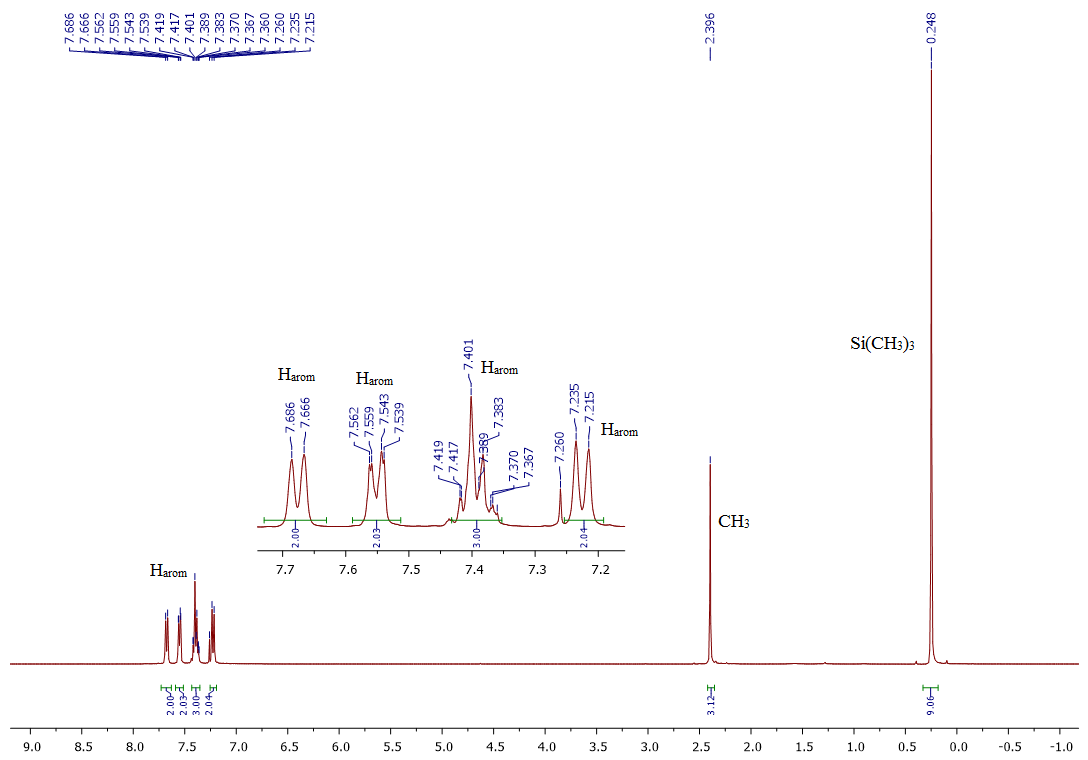


Figure S8. ^1^Н-NMR spectrum of the compound **Ic** (CDCl_3_, 400 MHz).


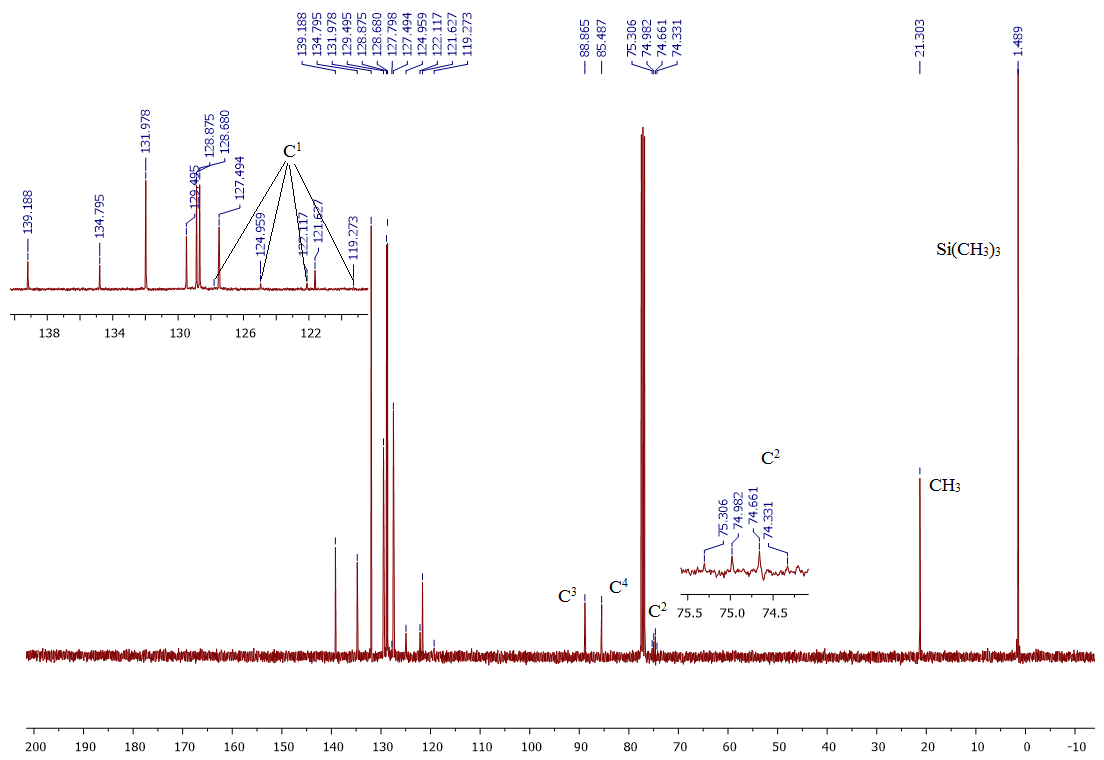


Figure S9. ^13^C-NMR spectrum of the compound **Ic** (CDCl_3_, 100 MHz).


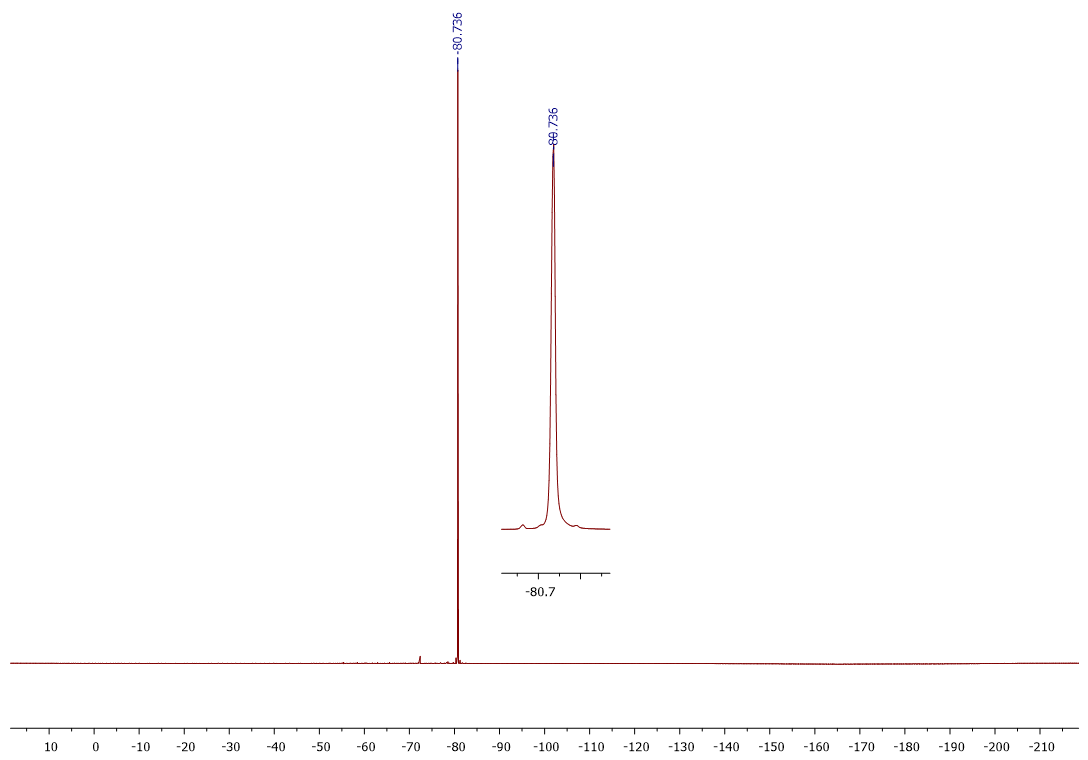


Figure S10. ^19^F-NMR spectrum of the compound **Ic** (CDCl_3_, 376 MHz).


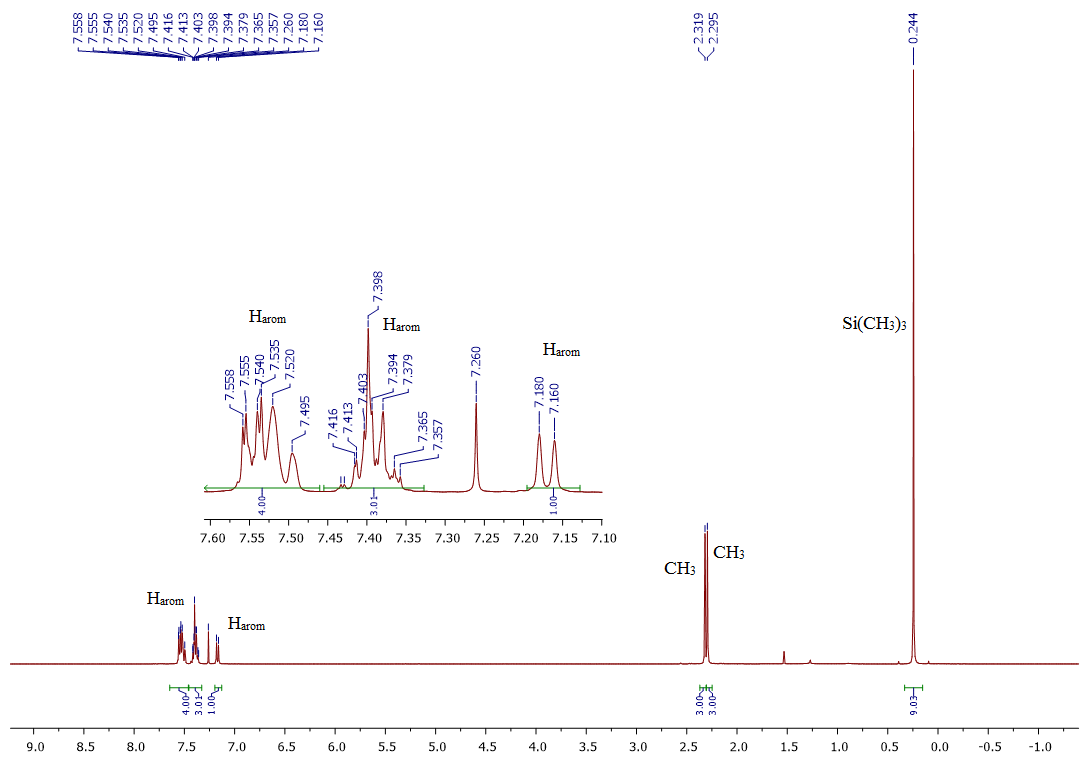


Figure S11. ^1^Н-NMR spectrum of the compound **Id** (CDCl_3_, 400 MHz).


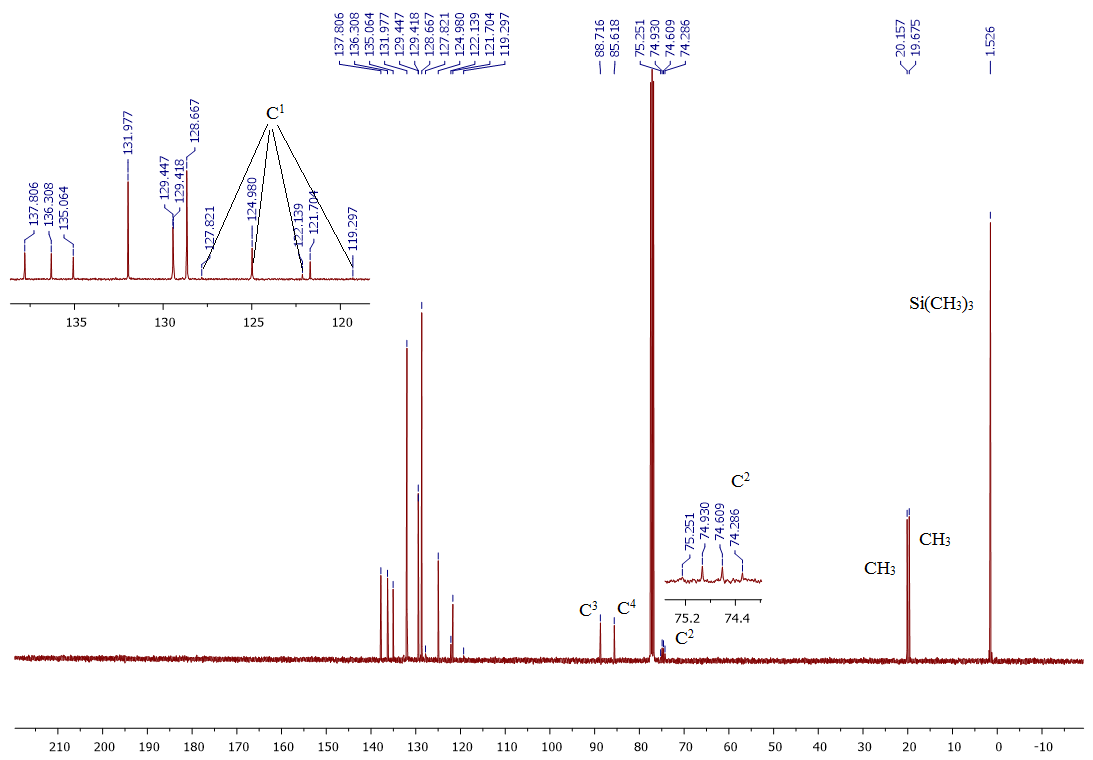


Figure S12. ^13^C-NMR spectrum of the compound **Id** (CDCl_3_, 100 MHz).


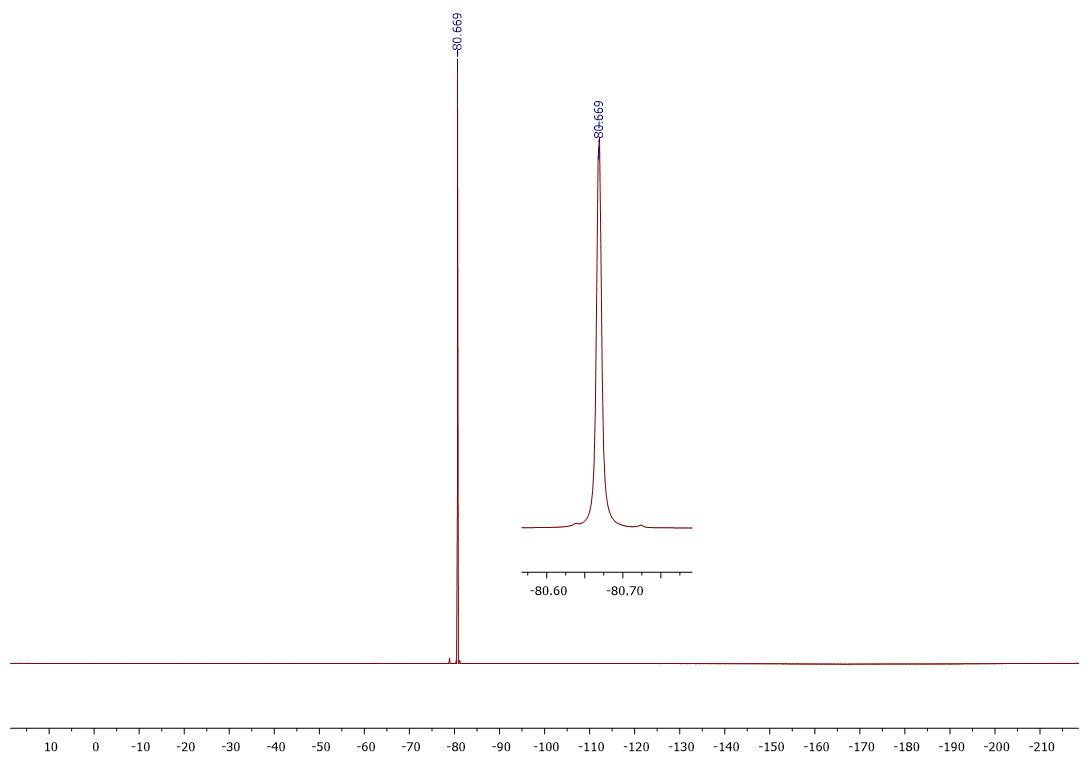


Figure S13. ^19^F-NMR spectrum of the compound **Id** (CDCl_3_, 376 MHz).


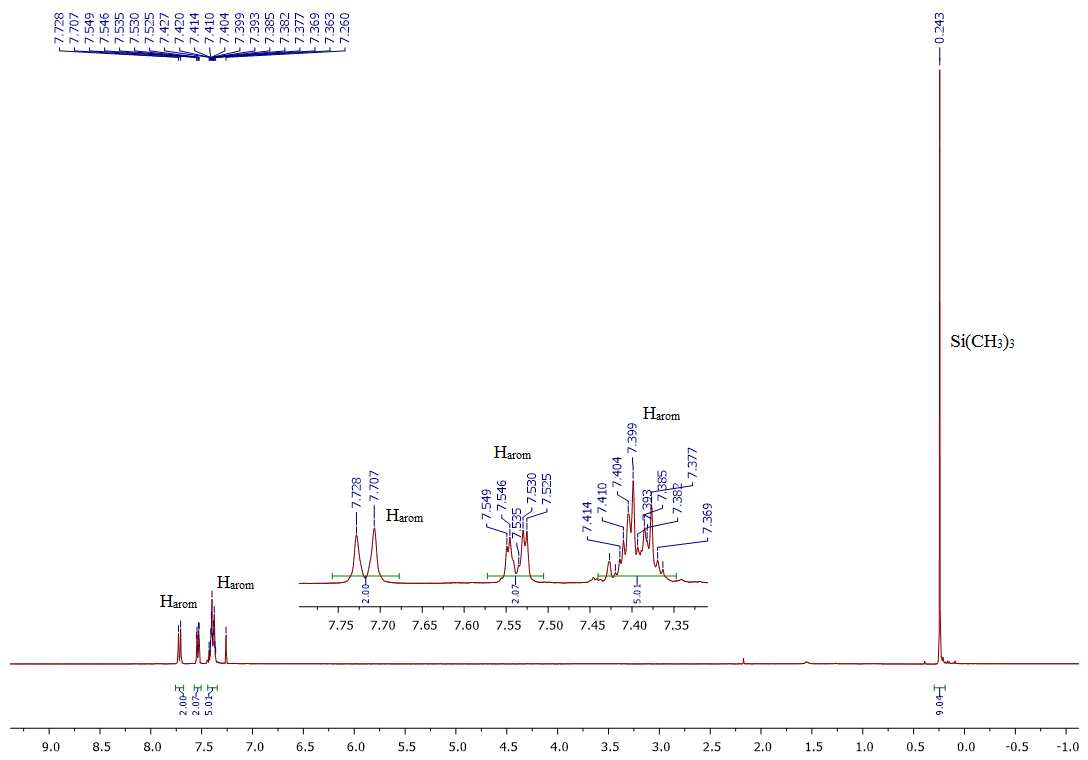


Figure S14. ^1^Н-NMR spectrum of the compound **Ie** (CDCl_3_, 400 MHz).


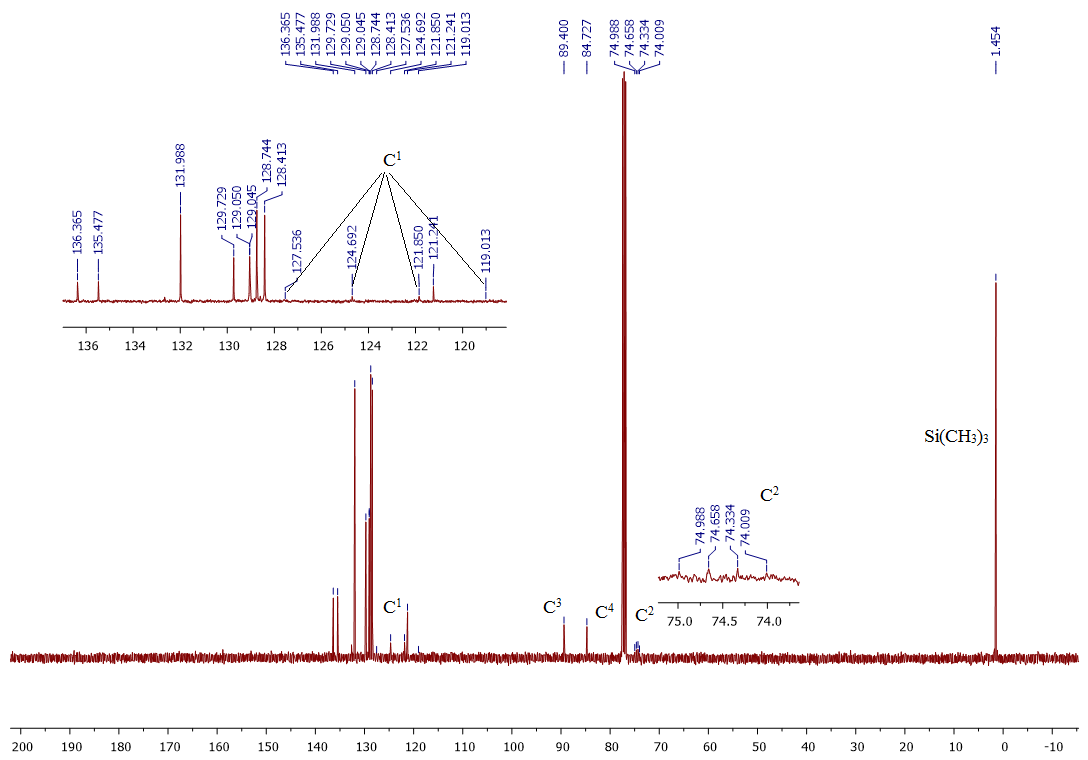


Figure S15. ^13^C-NMR spectrum of the compound **Ie** (CDCl_3_, 100 MHz).


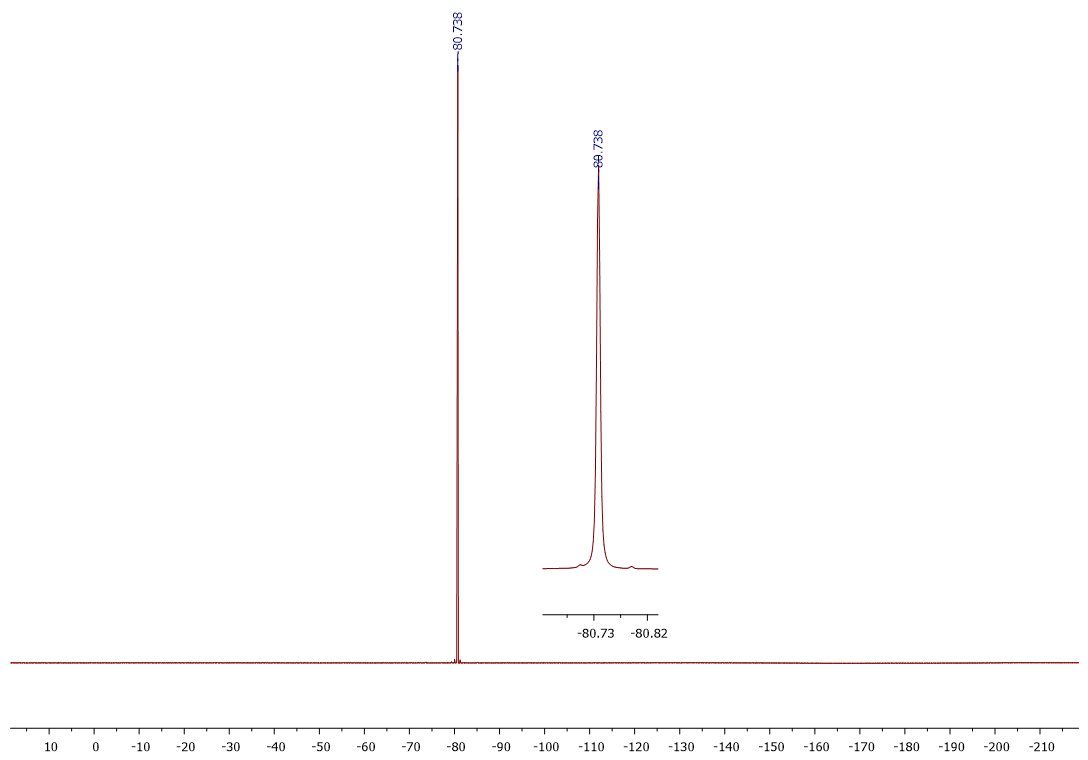


Figure S16. ^19^F-NMR spectrum of the compound **Ie** (CDCl_3_, 376 MHz).


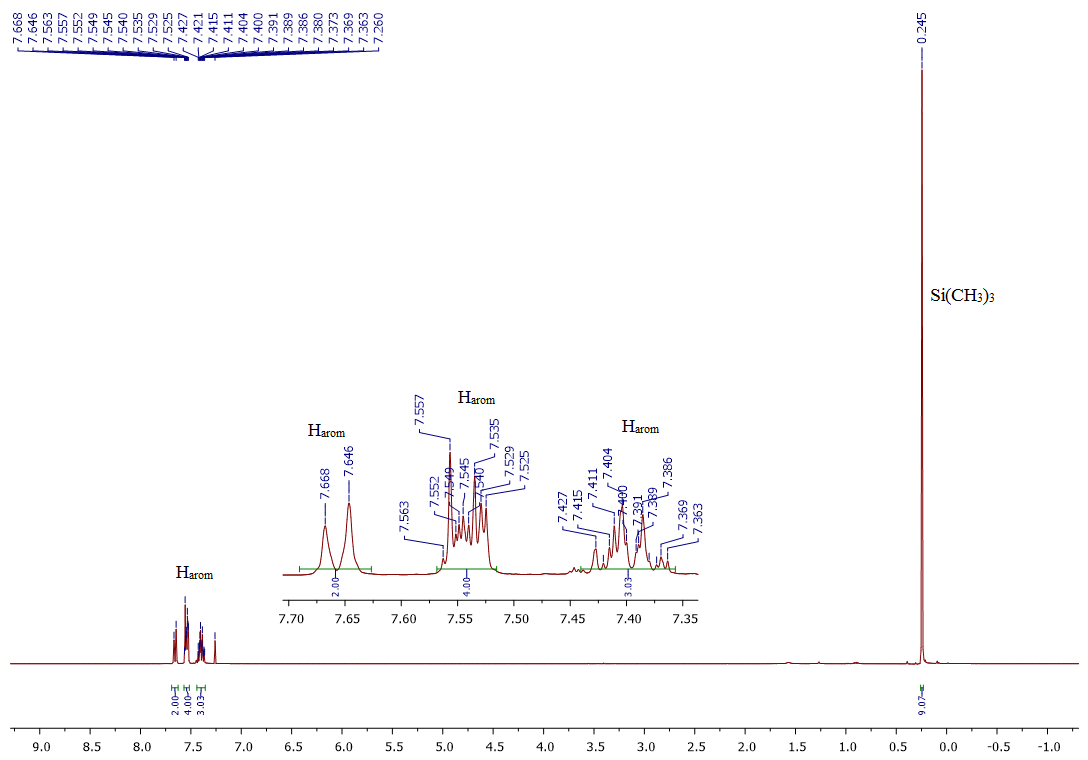


Figure S17. ^1^Н-NMR spectrum of the compound **If** (CDCl_3_, 400 MHz).


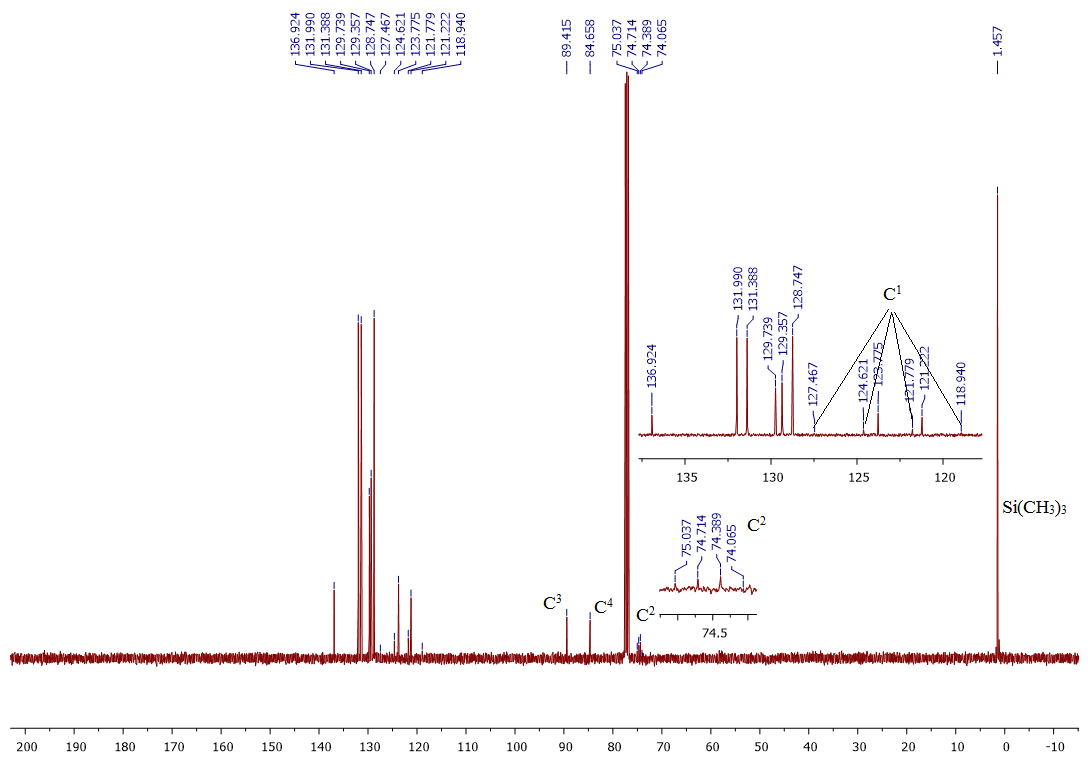


Figure S18. ^13^C-NMR spectrum of the compound **If** (CDCl_3_, 100 MHz).


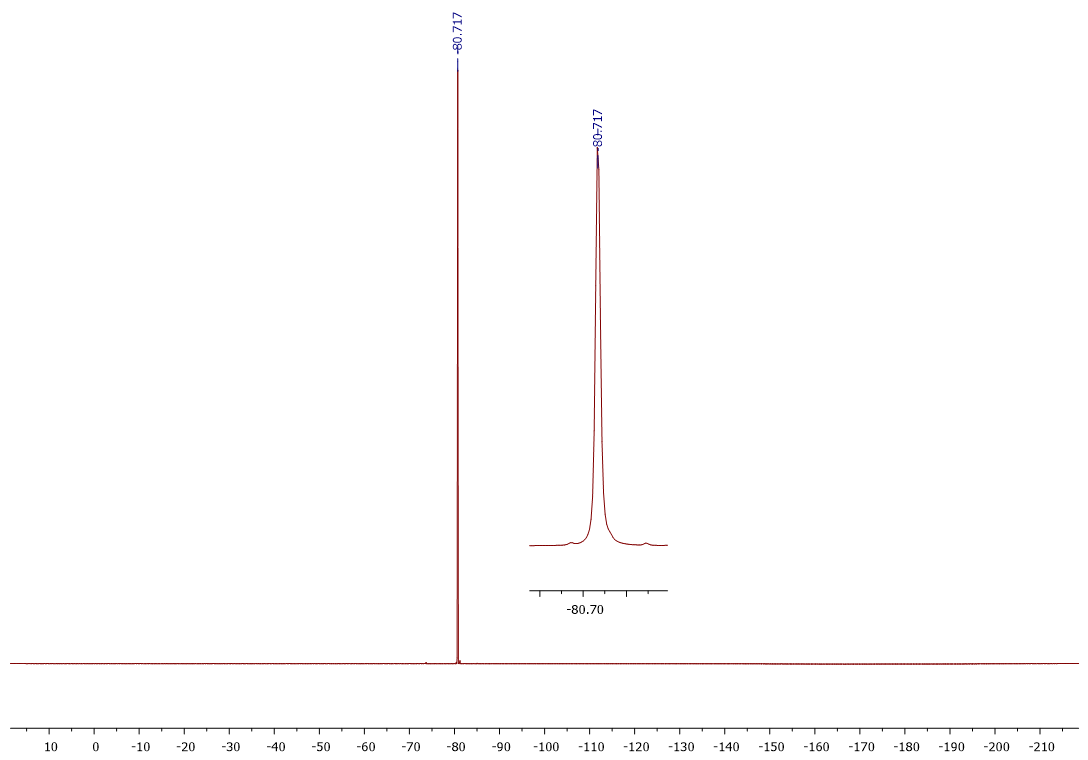


Figure S19. ^19^F-NMR spectrum of the compound **If** (CDCl_3_, 376 MHz).


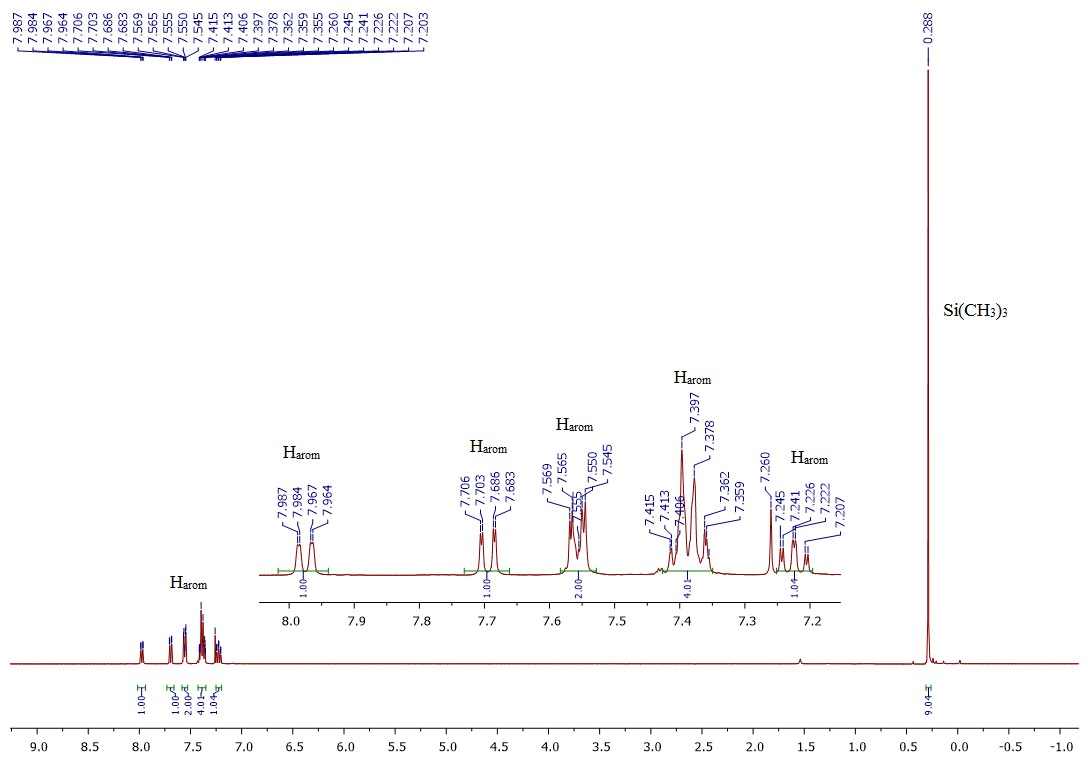


Figure S20. ^1^Н-NMR spectrum of the compound **Ig** (CDCl_3_, 400 MHz).


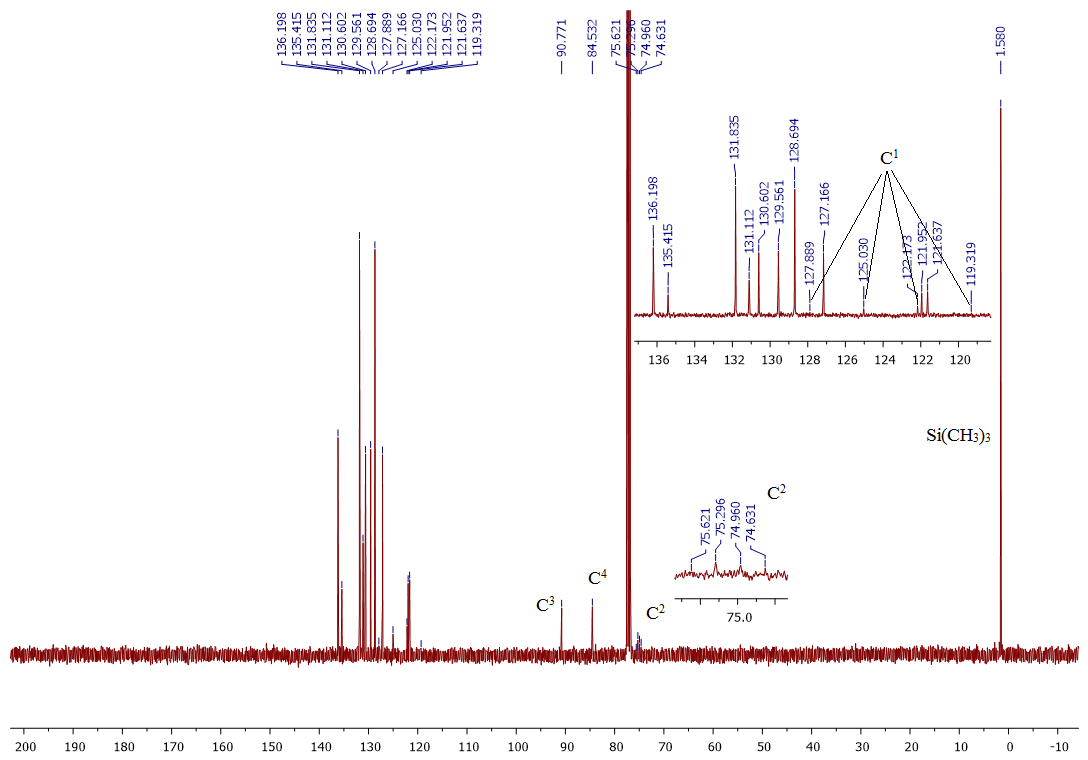


Figure S21. ^13^C-NMR spectrum of the compound **Ig** (CDCl_3_, 100 MHz).


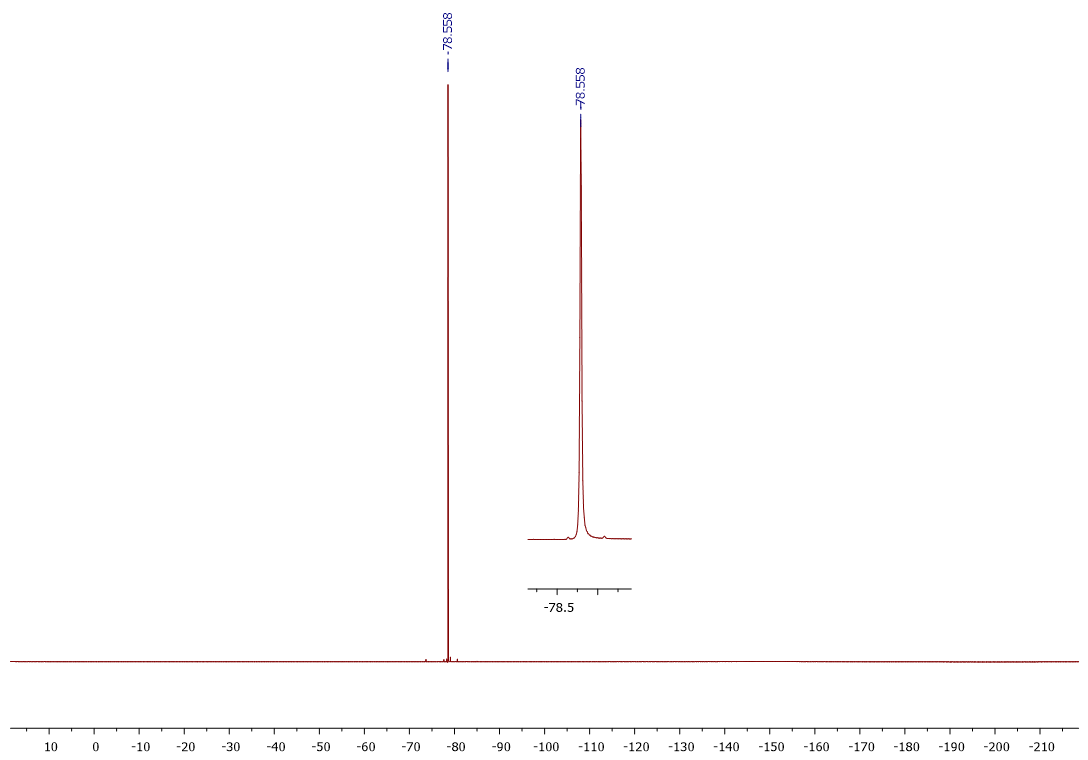


Figure S22. ^19^F-NMR spectrum of the compound **Ig** (CDCl_3_, 376 MHz).


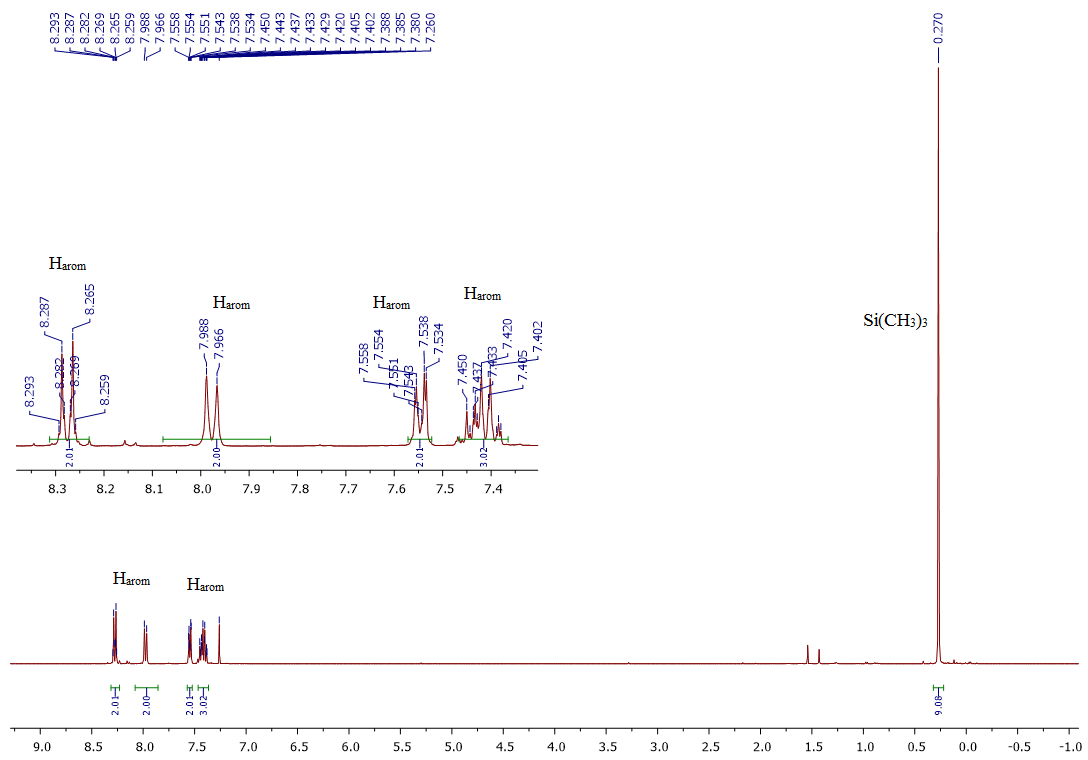


Figure S23. ^1^Н-NMR spectrum of the compound **Ih** (CDCl_3_, 400 MHz).


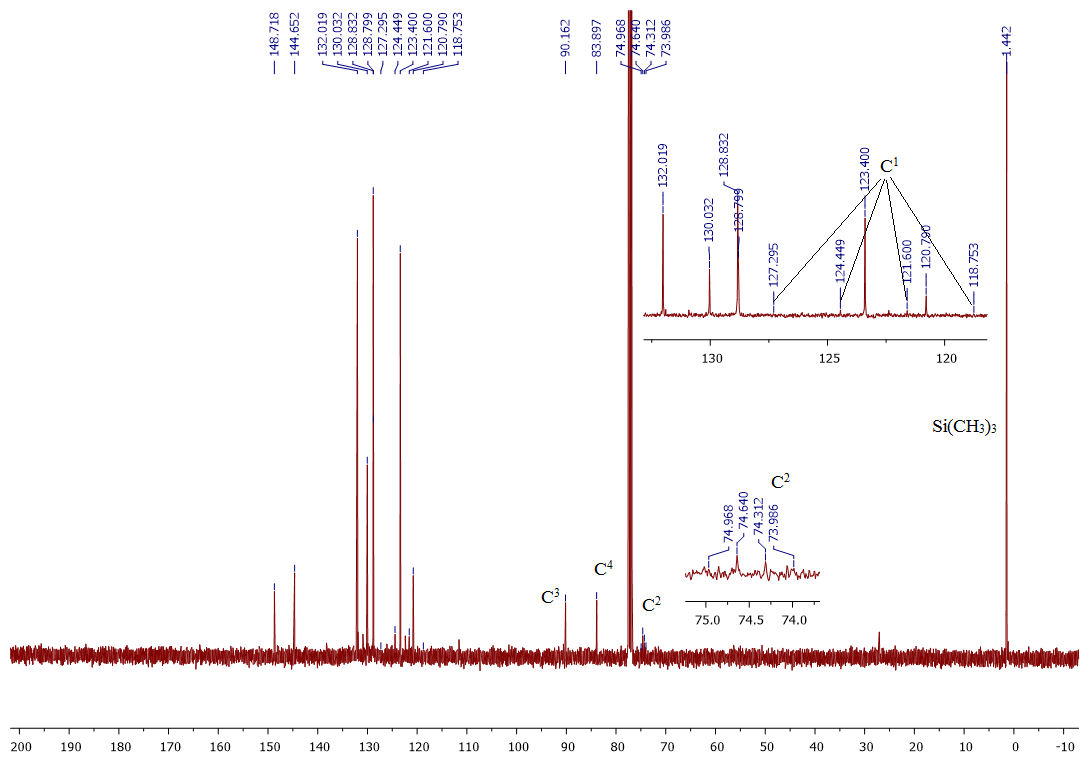


Figure S24. ^13^C-NMR spectrum of the compound **Ih** (CDCl_3_, 100 MHz).


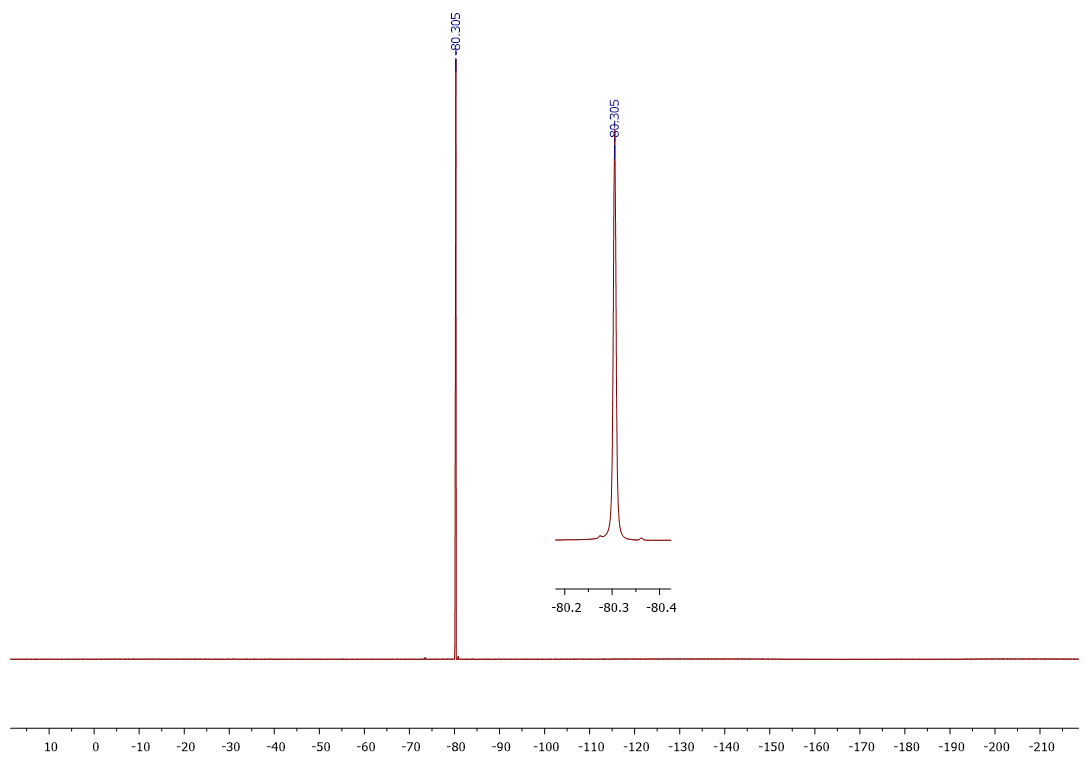


Figure S25. ^19^F-NMR spectrum of the compound **Ih** (CDCl_3_, 376 MHz).


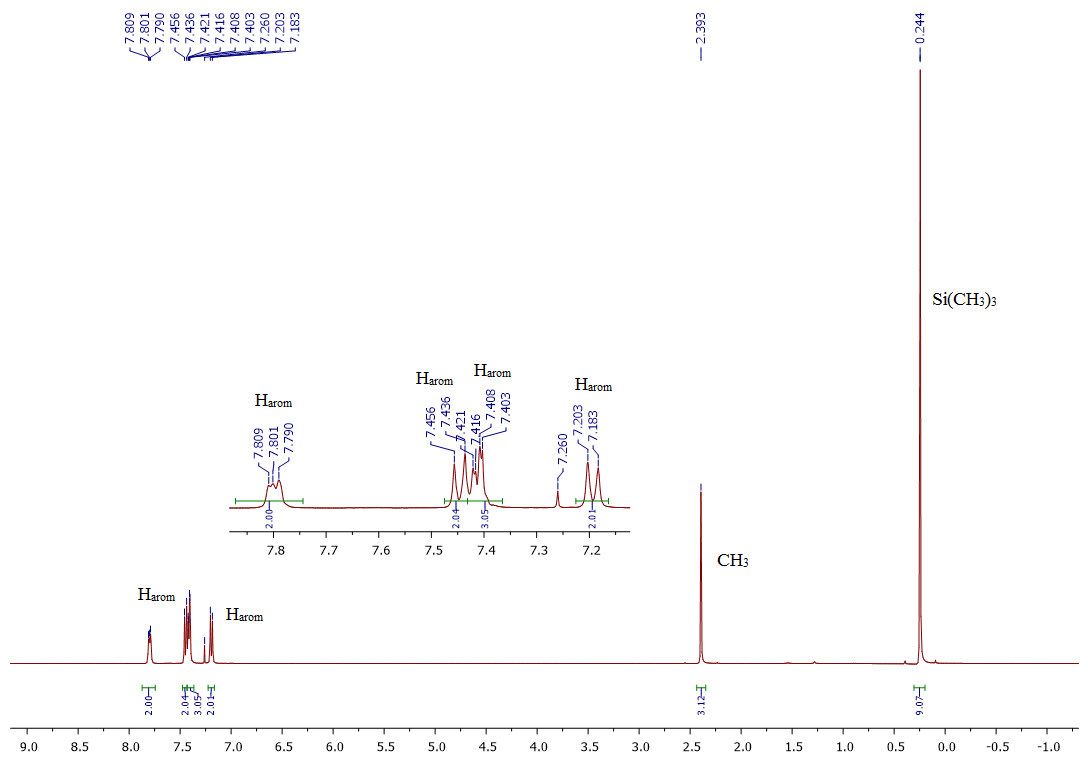


Figure S26. ^1^Н-NMR spectrum of the compound **Ii** (CDCl_3_, 400 MHz).


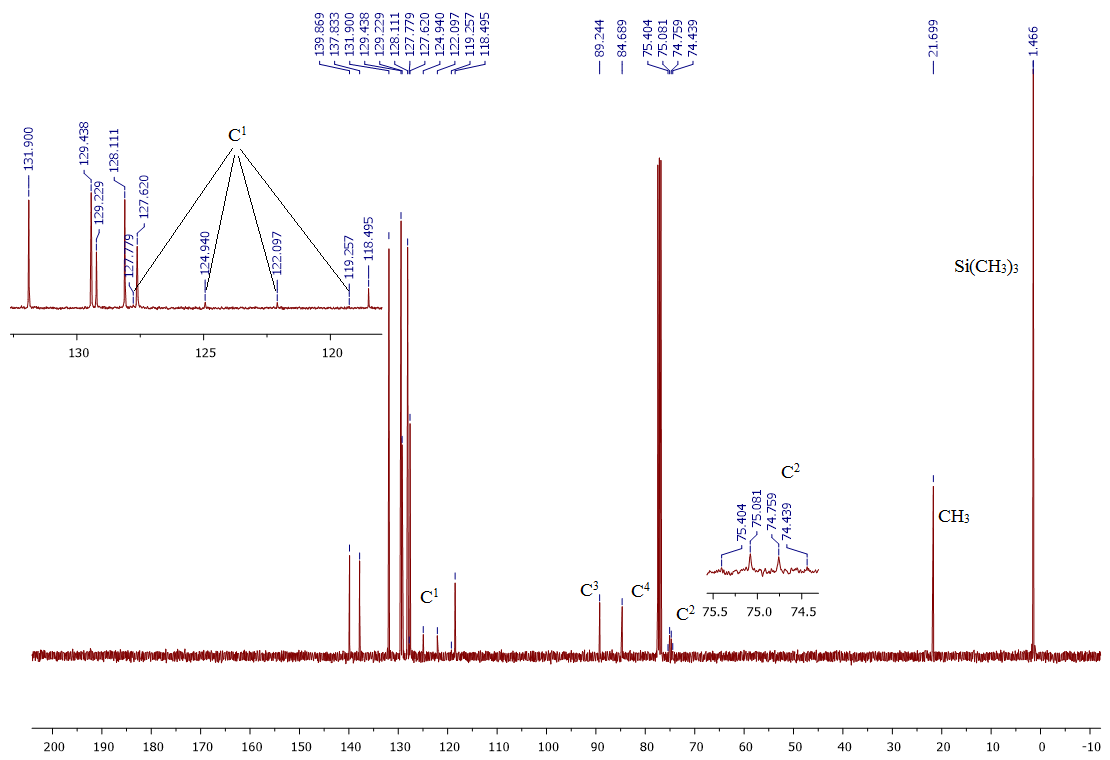


Figure S27. ^13^C-NMR spectrum of the compound **Ii** (CDCl_3_, 100 MHz).


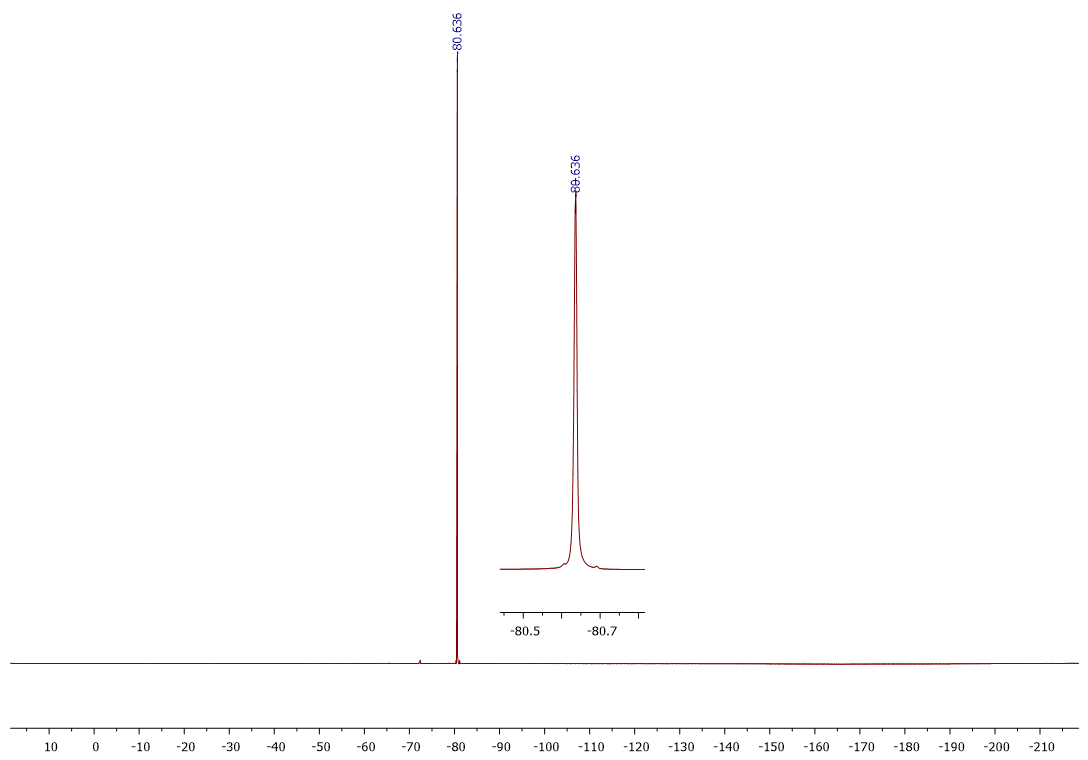


Figure S28. ^19^F-NMR spectrum of the compound **Ii** (CDCl_3_, 376 MHz).


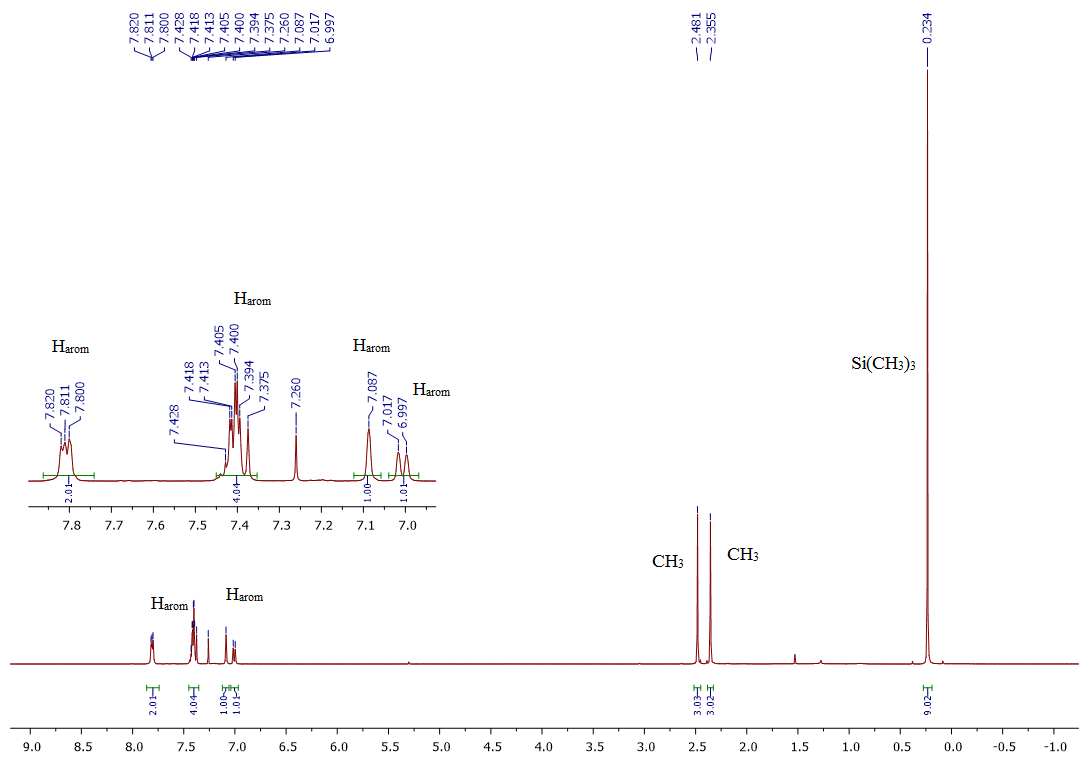


Figure S29. ^1^Н-NMR spectrum of the compound **Ij** (CDCl_3_, 400 MHz).


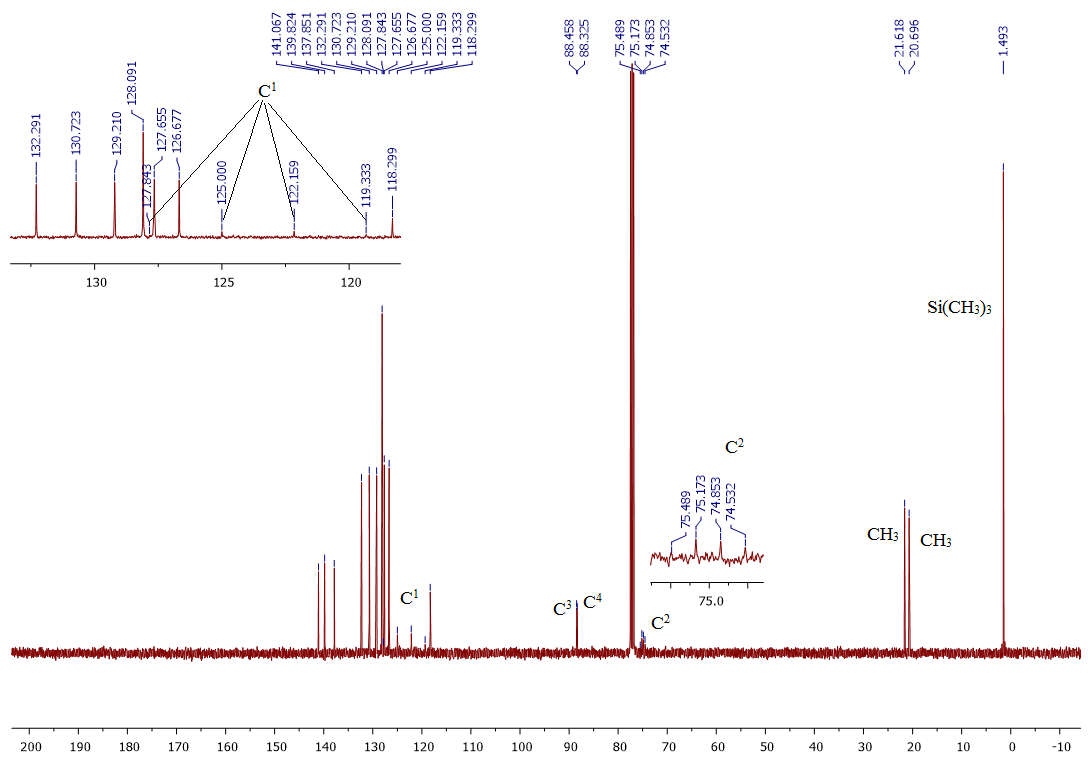


Figure S30. ^13^C-NMR spectrum of the compound **Ij** (CDCl_3_, 100 MHz).


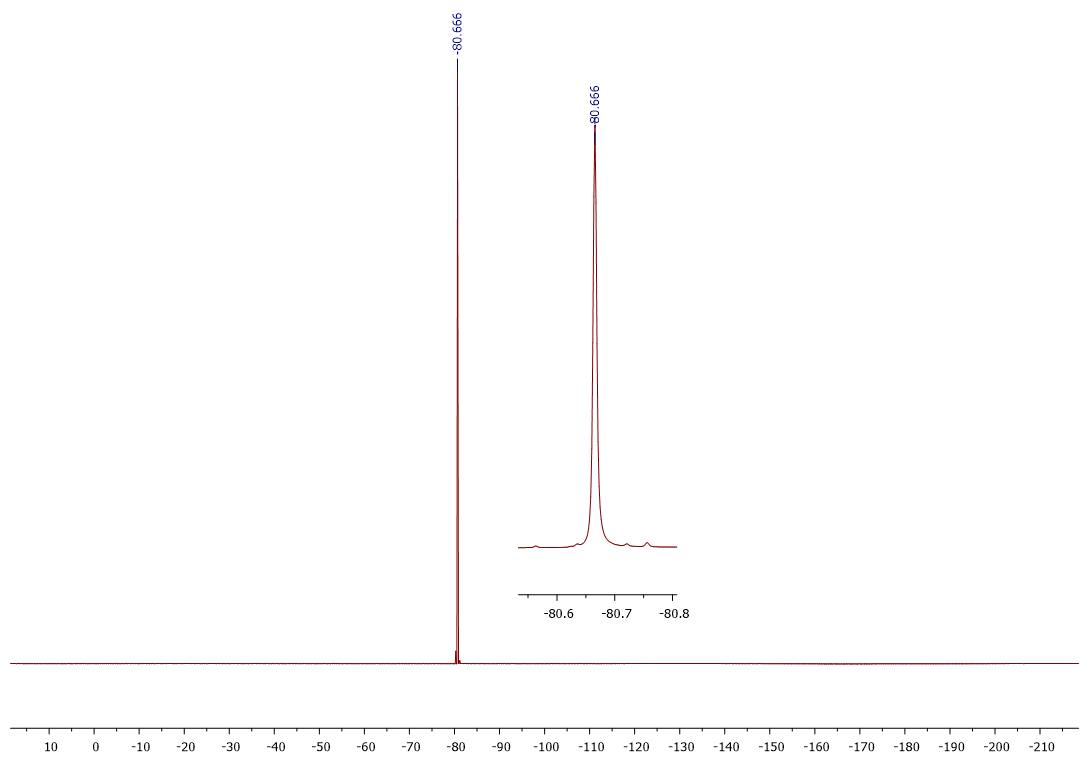


Figure S31. ^19^F-NMR spectrum of the compound **Ij** (CDCl_3_, 376 MHz).


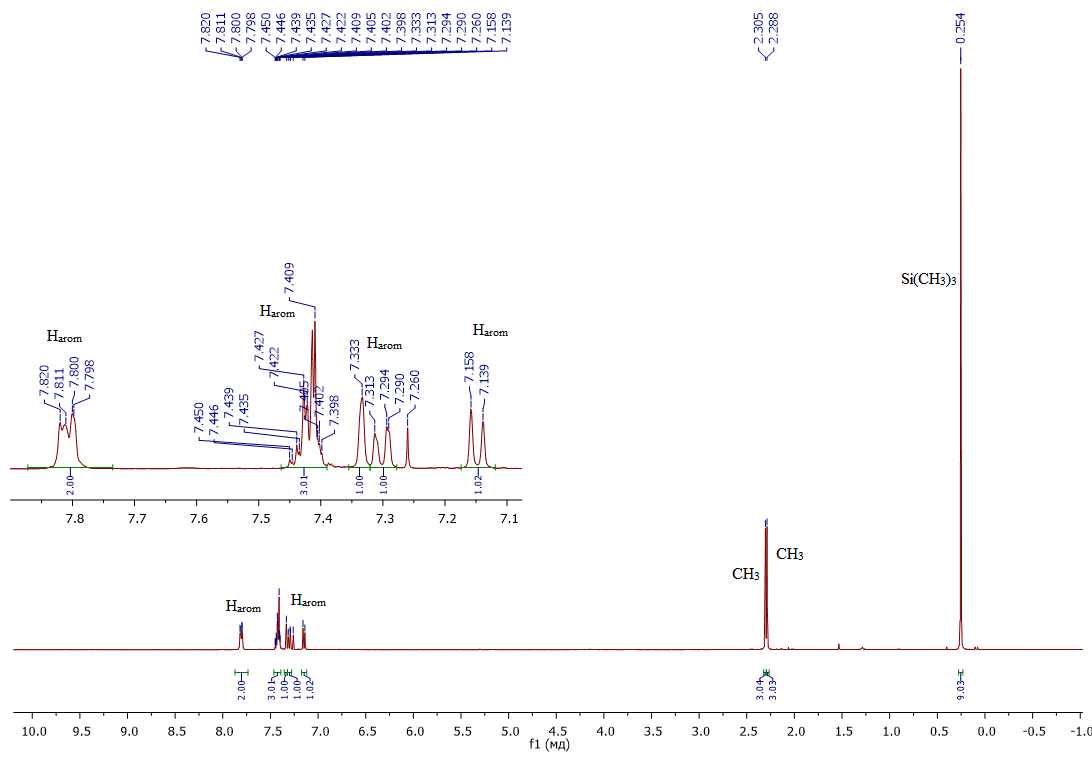


Figure S32. ^1^Н-NMR spectrum of the compound **Ik** (CDCl_3_, 400 MHz).


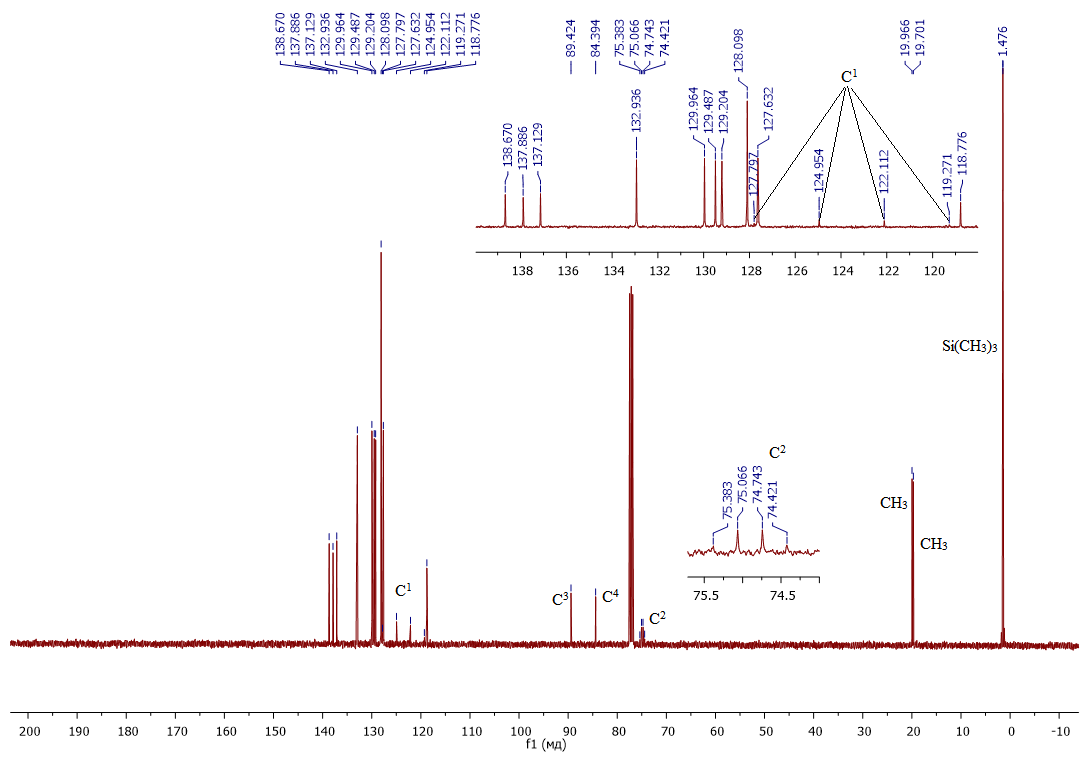


Figure S33. ^13^C-NMR spectrum of the compound **Ik** (CDCl_3_, 100 MHz).


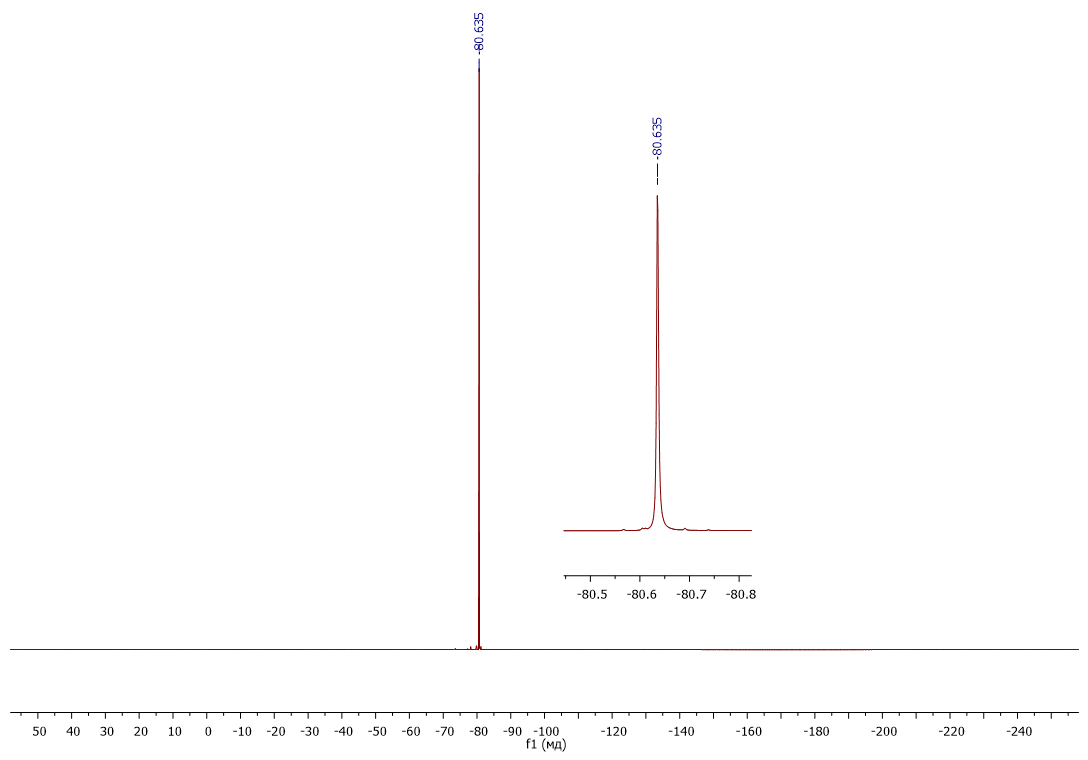


Figure S34. ^19^F-NMR spectrum of the compound **Ik** (CDCl_3_, 376 MHz).


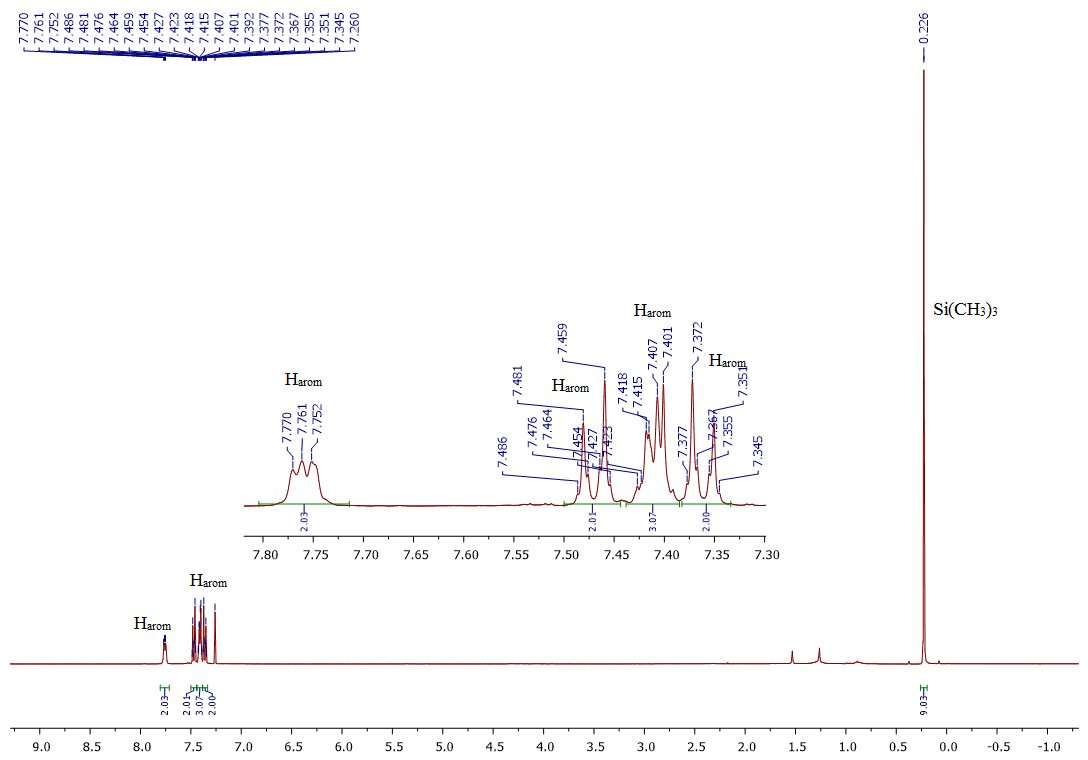


Figure S35. ^1^Н-NMR spectrum of the compound **Il** (CDCl_3_, 400 MHz).


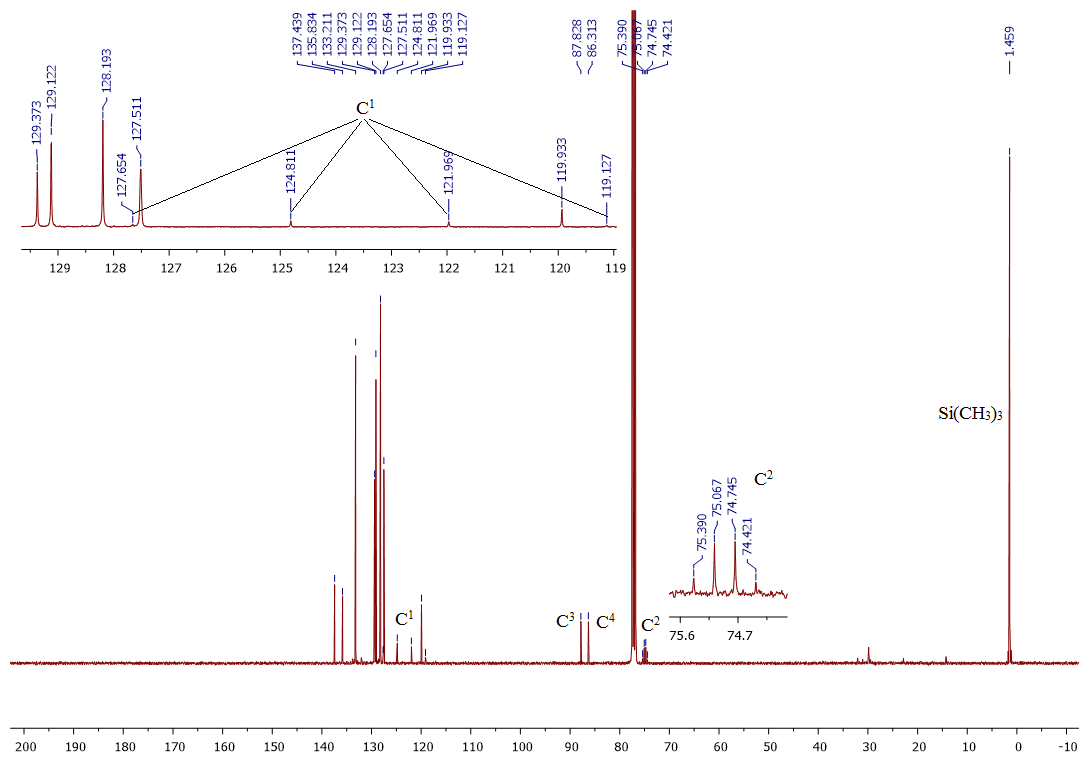


Figure S36. ^13^C-NMR spectrum of the compound **Il** (CDCl_3_, 100 MHz).


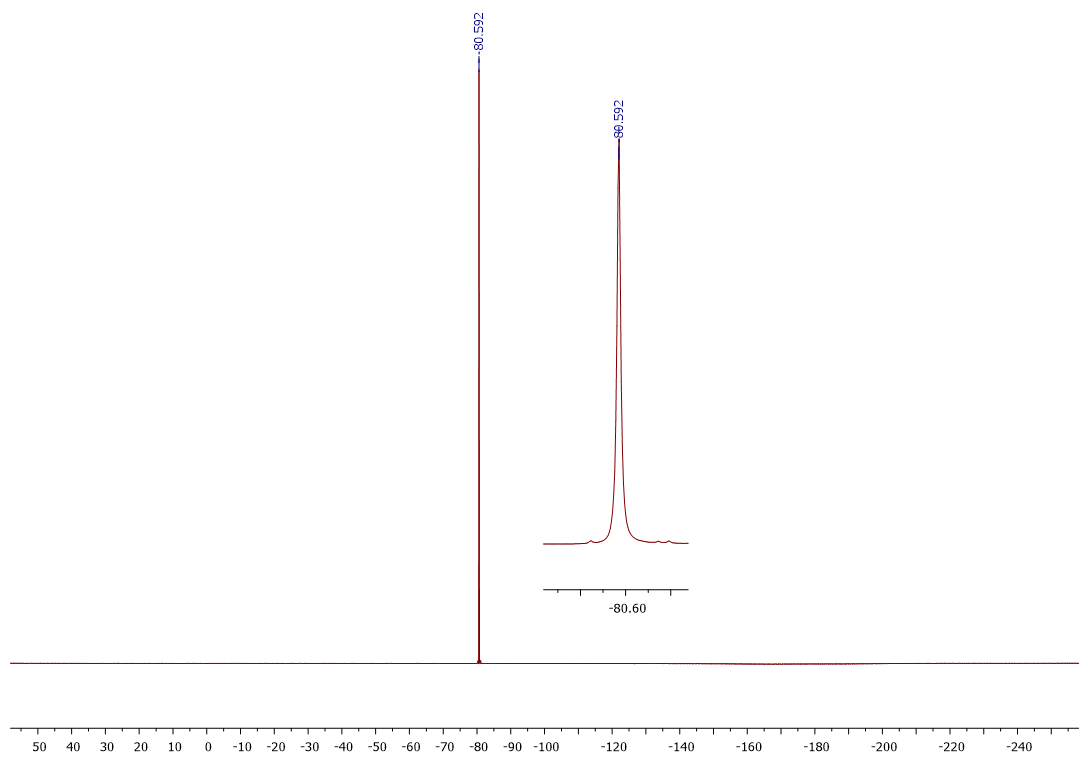


Figure S37. ^19^F-NMR spectrum of the compound **Il** (CDCl_3_, 376 MHz).


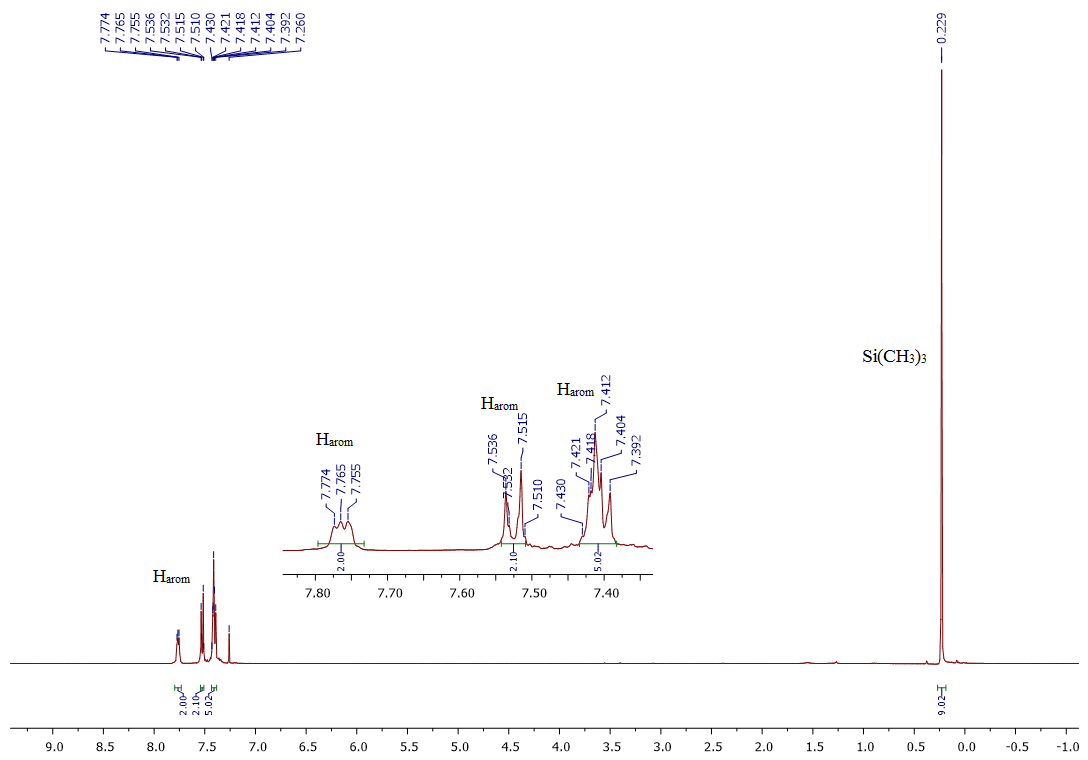


Figure S38. ^1^Н-NMR spectrum of the compound **Im** (CDCl_3_, 400 MHz).


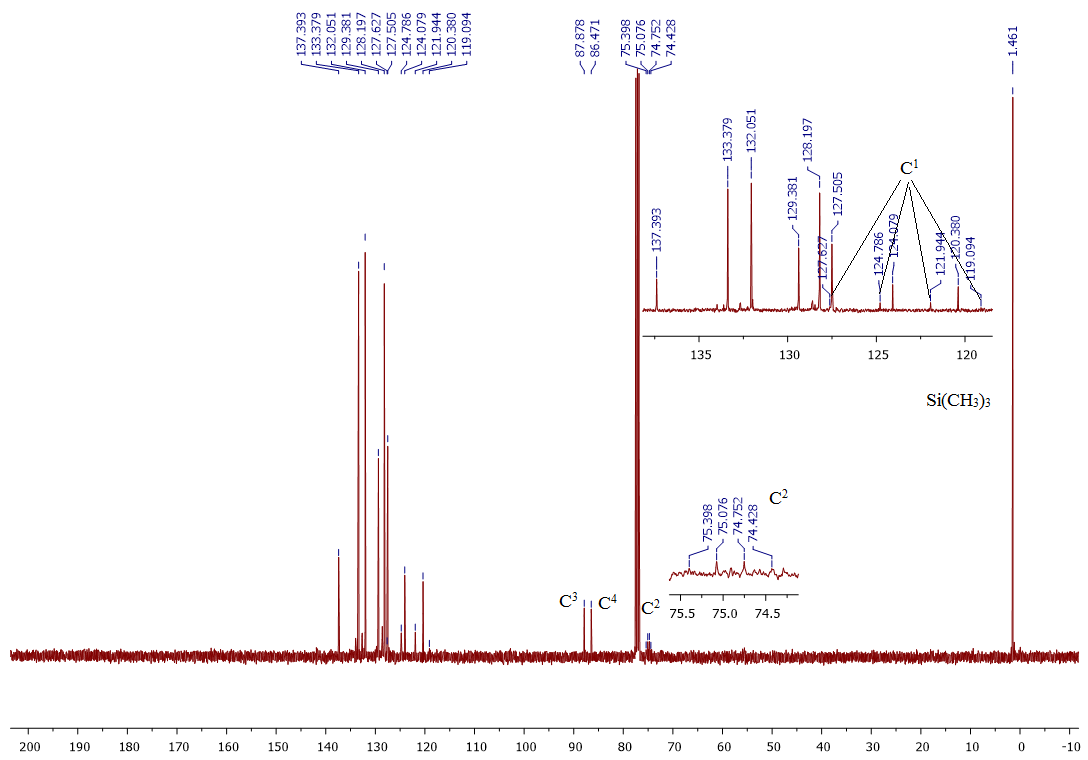


Figure S39. ^13^C-NMR spectrum of the compound **Im** (CDCl_3_, 100 MHz).


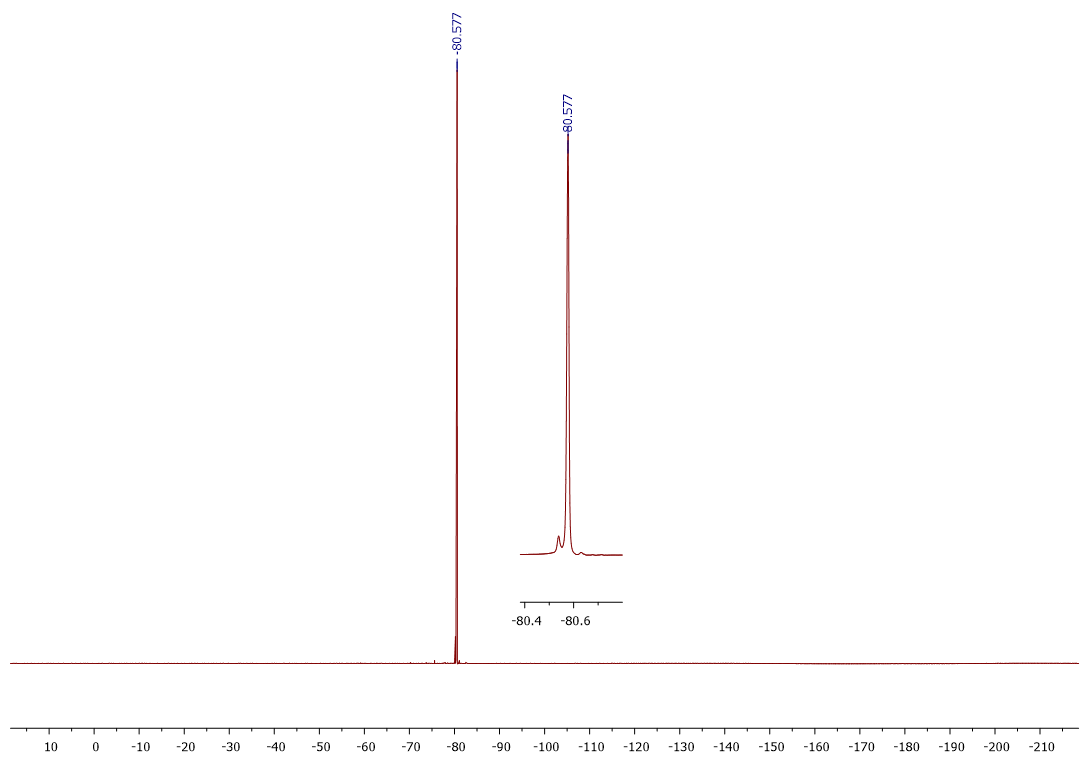


Figure S40. ^19^F-NMR spectrum of the compound **Im** (CDCl_3_, 376 MHz).


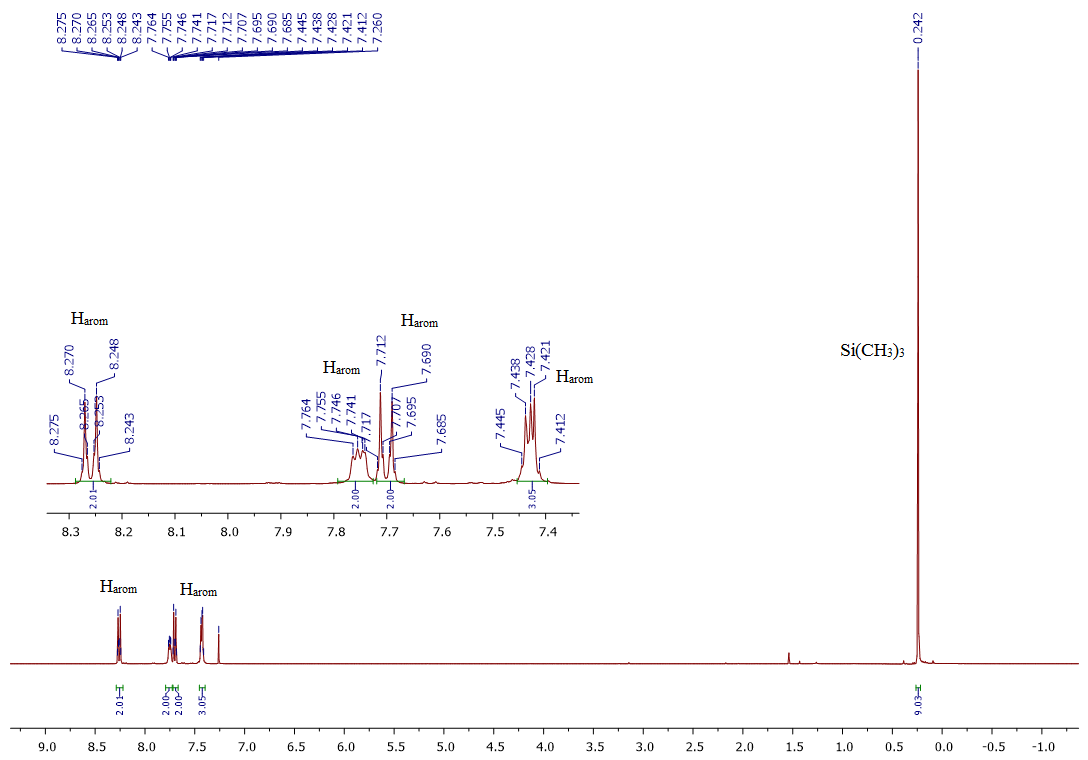


Figure S41. ^1^Н-NMR spectrum of the compound **In** (CDCl_3_, 400 MHz).


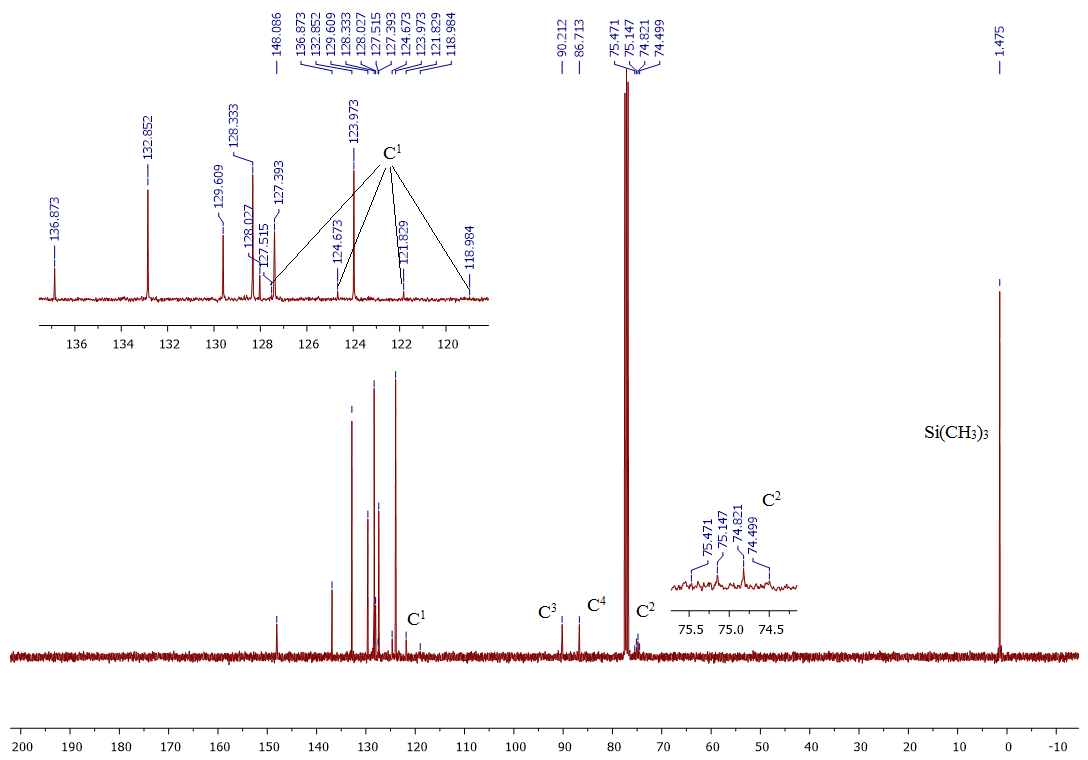


Figure S42. ^13^C-NMR spectrum of the compound **In** (CDCl_3_, 100 MHz).


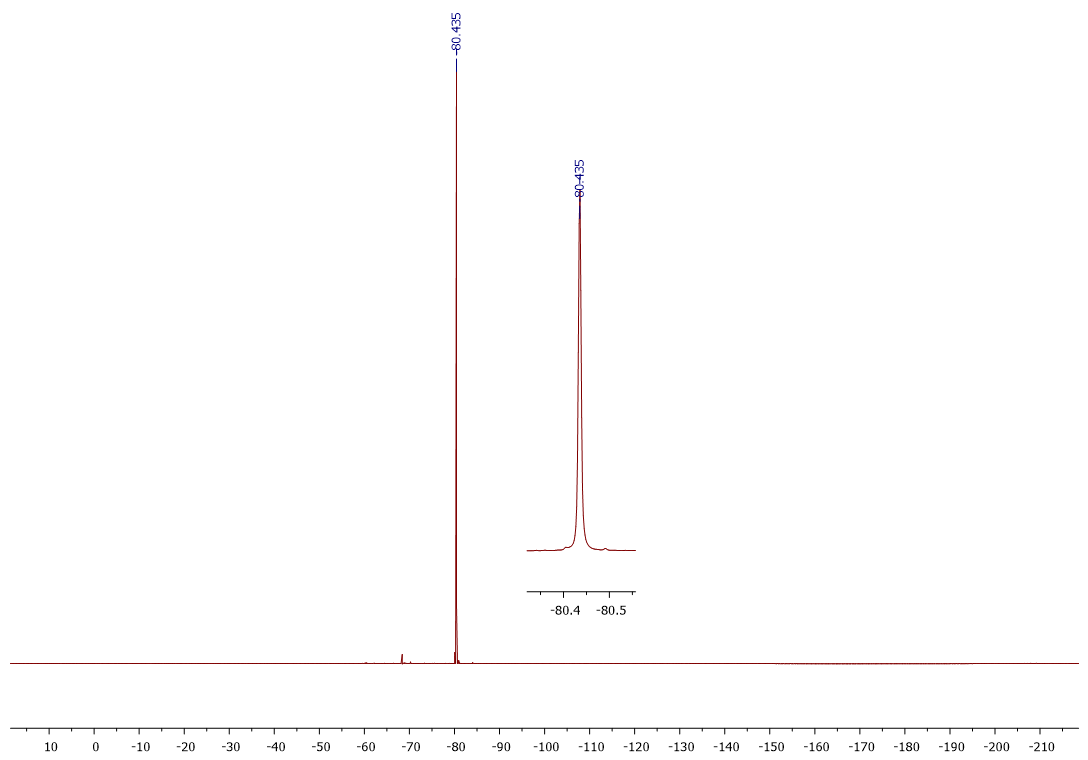


Figure S43. ^19^F-NMR spectrum of the compound **In** (CDCl_3_, 376 MHz).


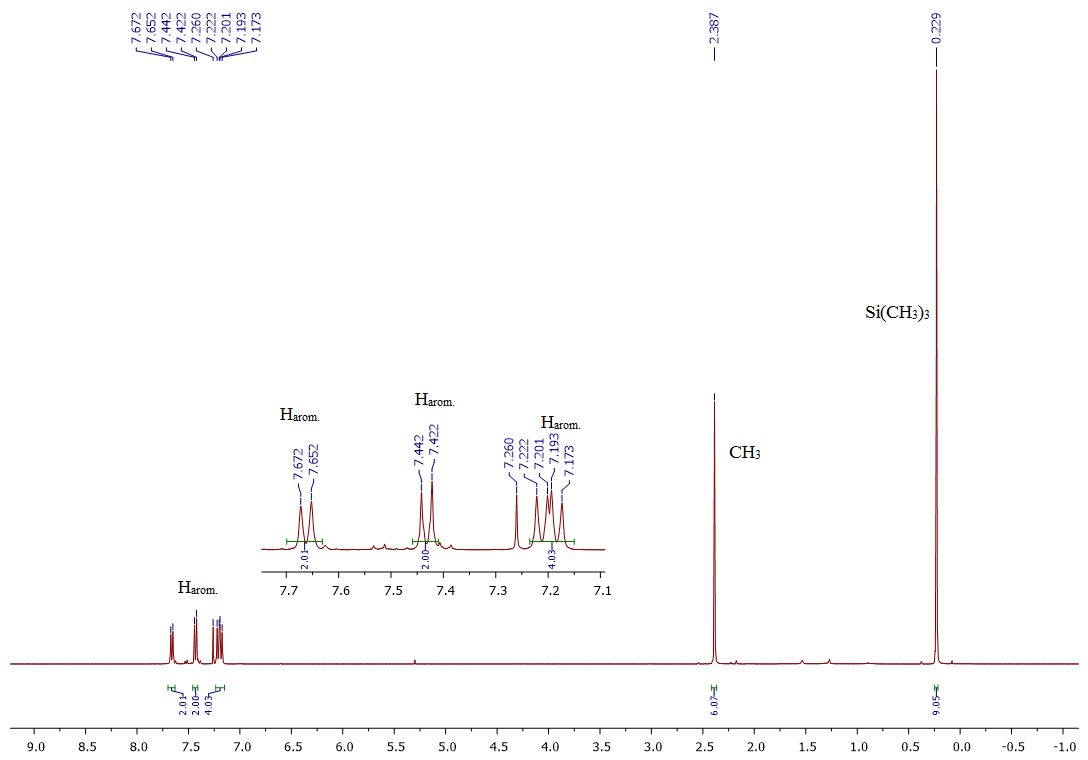


Figure S44. ^1^Н-NMR spectrum of the compound **Io** (CDCl_3_, 400 MHz).


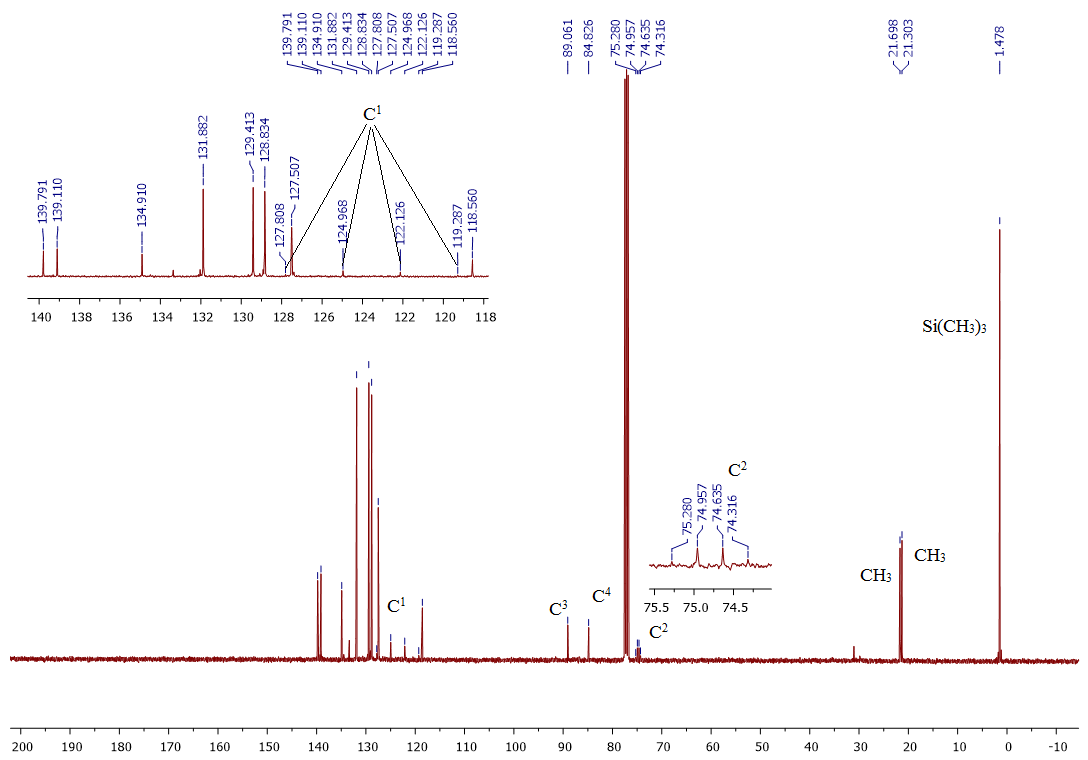


Figure S45. ^13^C-NMR spectrum of the compound **Io** (CDCl_3_, 100 MHz).


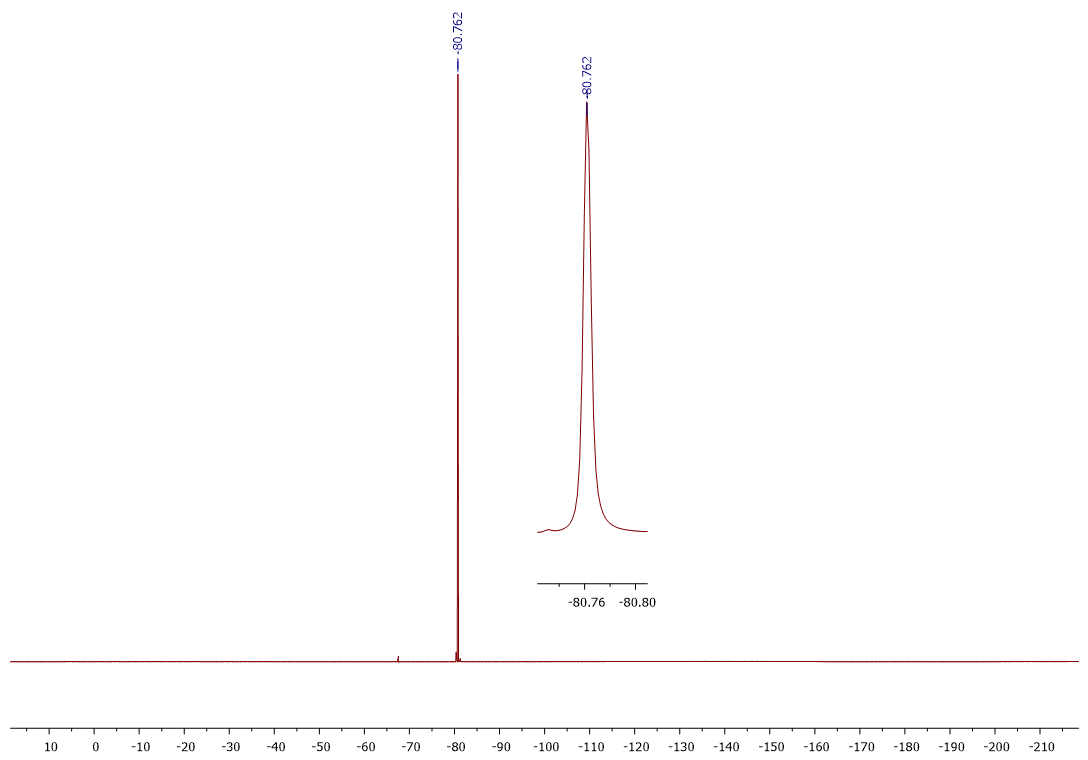


Figure S46. ^19^F-NMR spectrum of the compound **Io** (CDCl_3_, 376 MHz).


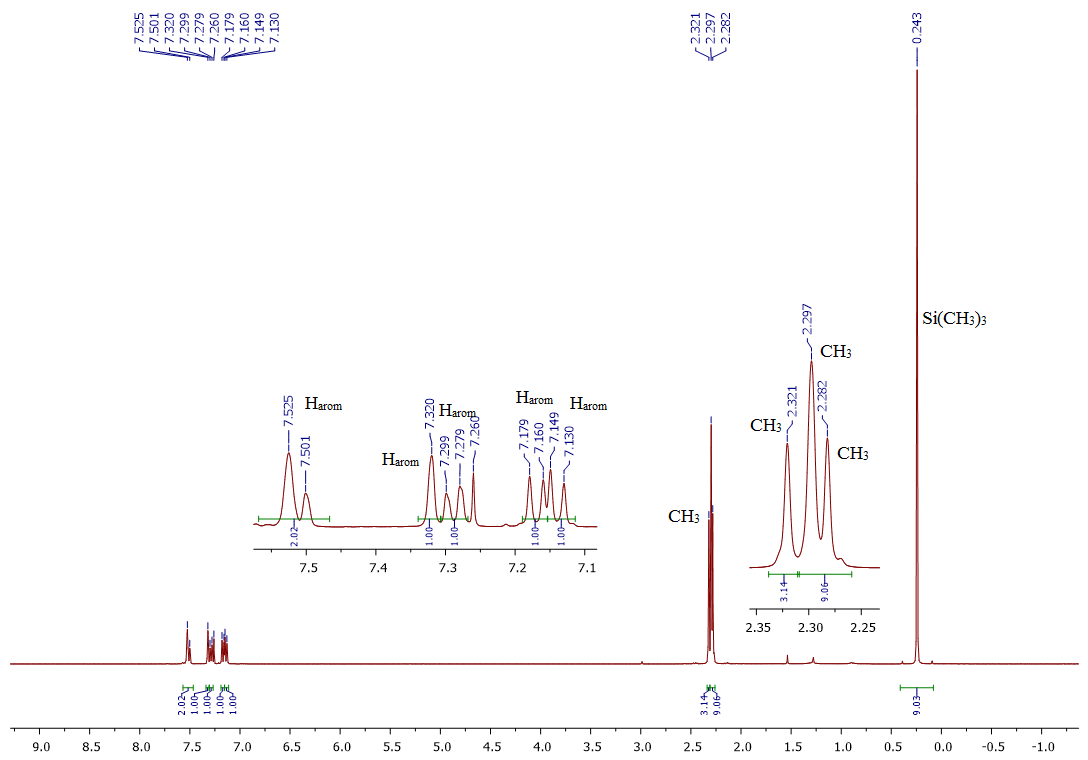


Figure S47. ^1^Н-NMR spectrum of the compound **Ip** (CDCl_3_, 400 MHz).


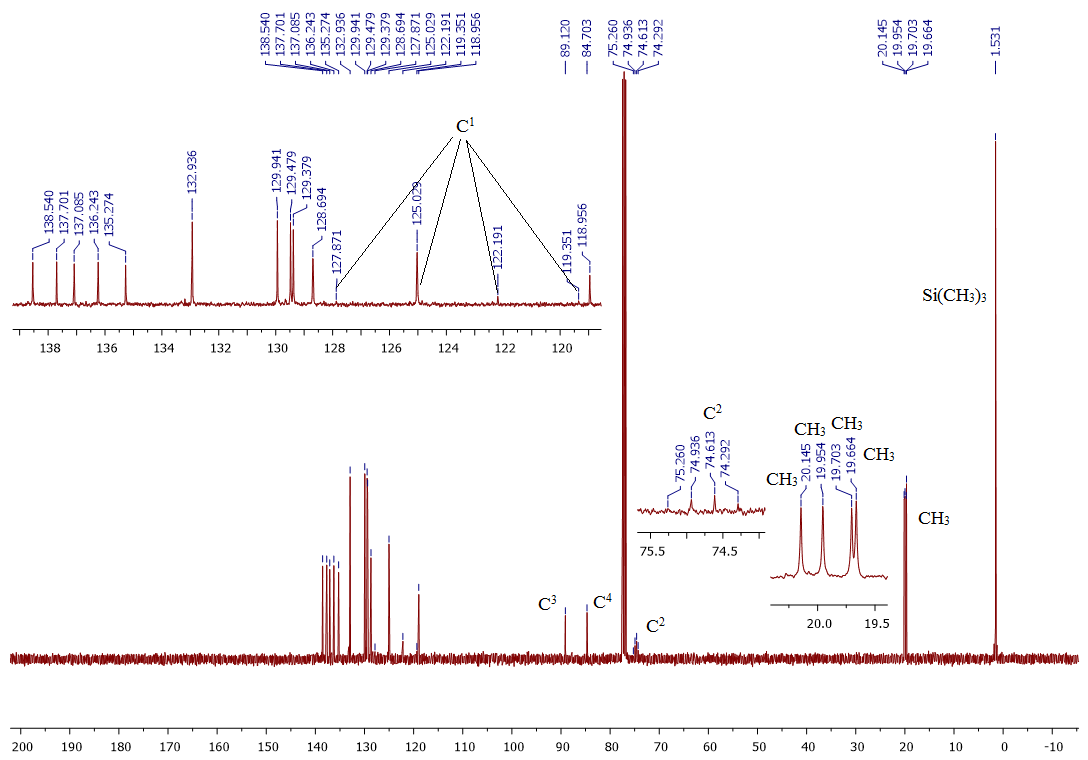


Figure S48. ^13^C-NMR spectrum of the compound **Ip** (CDCl_3_, 100 MHz).


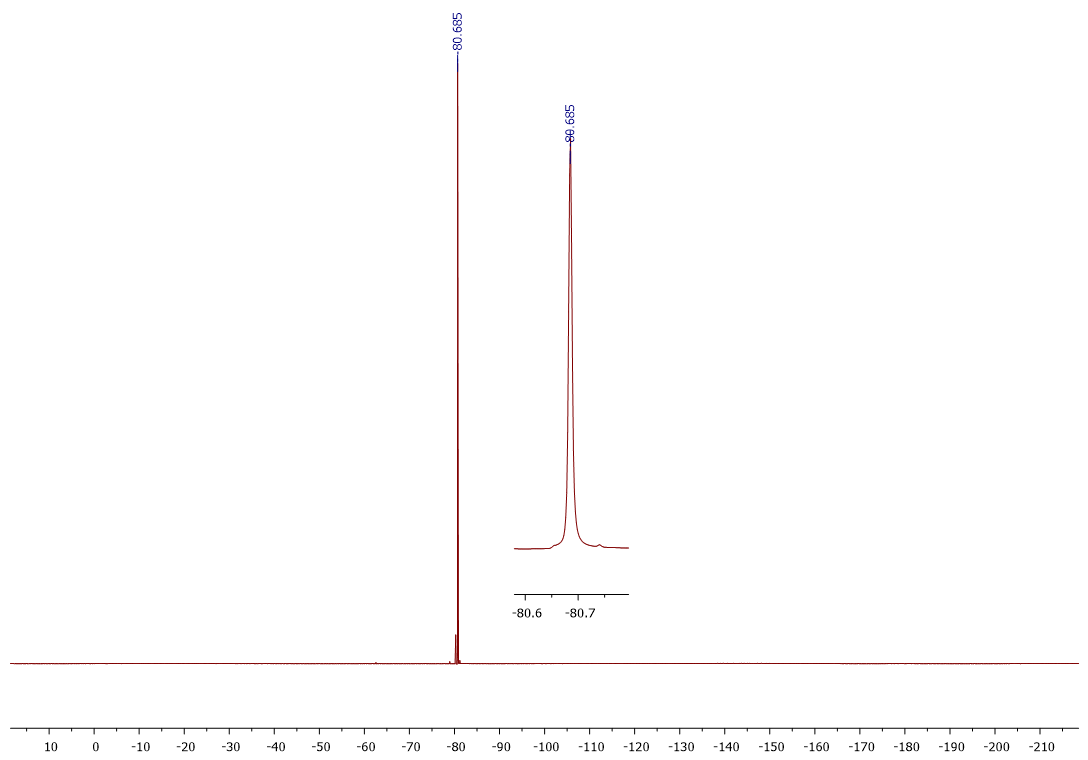


Figure S49. ^19^F-NMR spectrum of the compound **Ip** (CDCl_3_, 376 MHz).


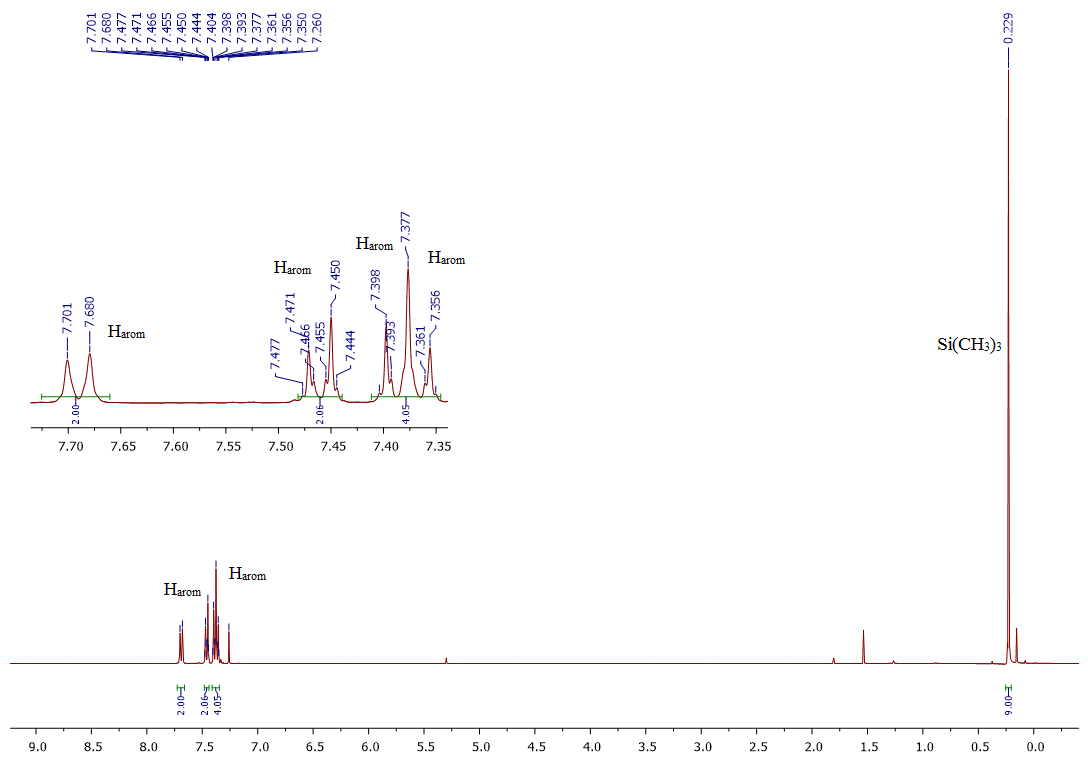


Figure S50. ^1^Н-NMR spectrum of the compound **Iq** (CDCl_3_, 400 MHz).


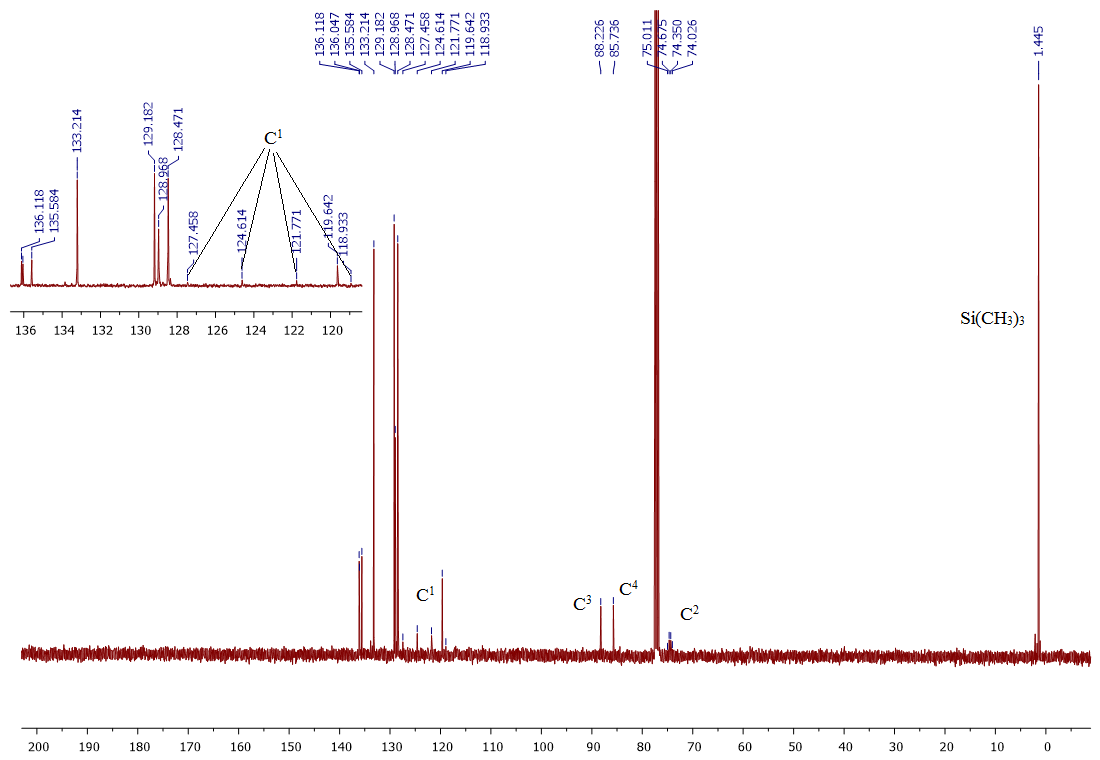


Figure S51. ^13^C-NMR spectrum of the compound **Iq** (CDCl_3_, 100 MHz).


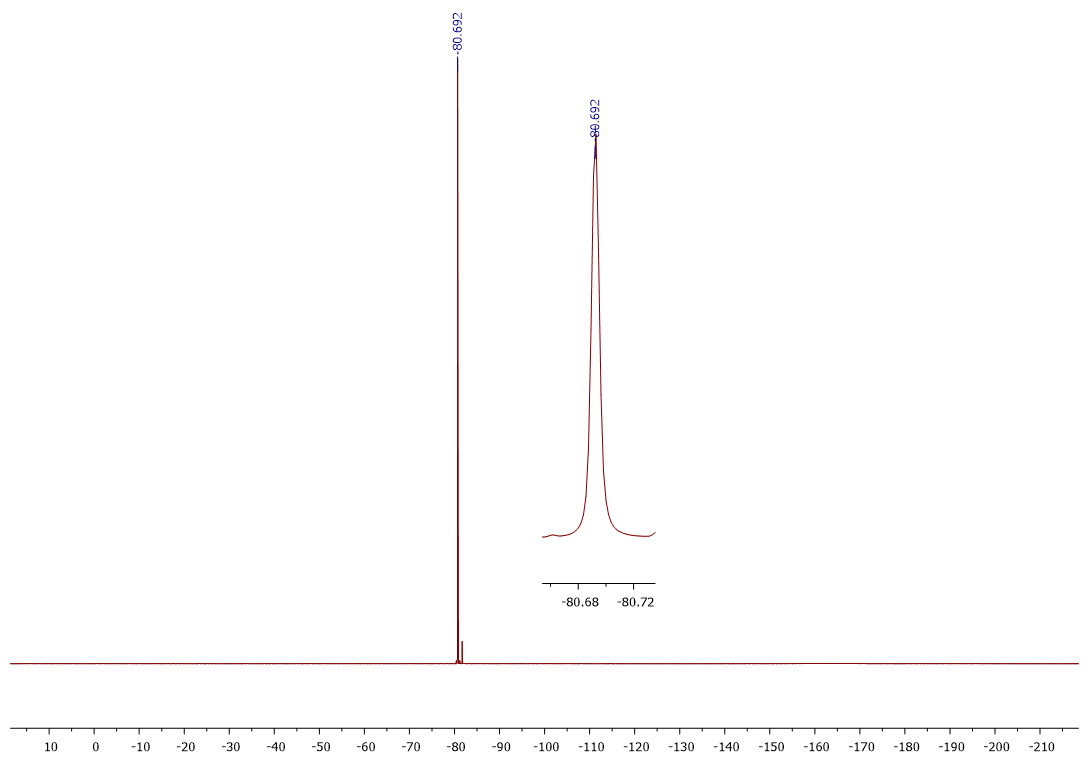


Figure S52. ^19^F-NMR spectrum of the compound **Iq** (CDCl_3_, 376 MHz).


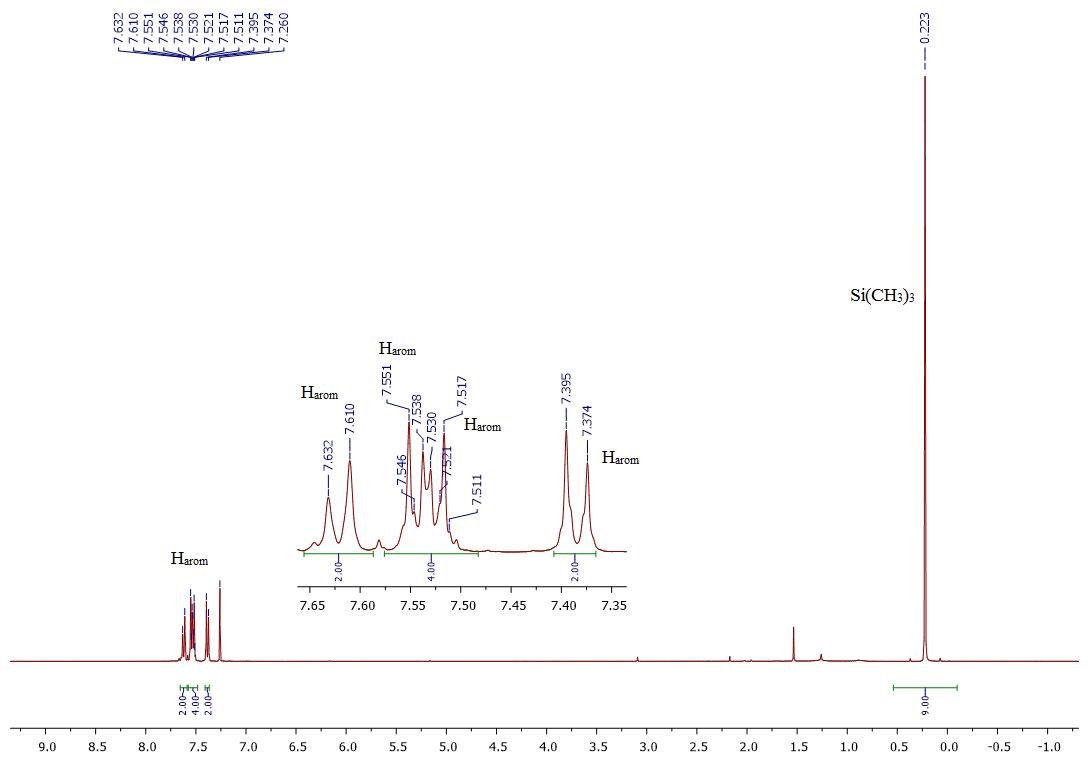


Figure S53. ^1^Н-NMR spectrum of the compound **Ir** (CDCl_3_, 400 MHz).


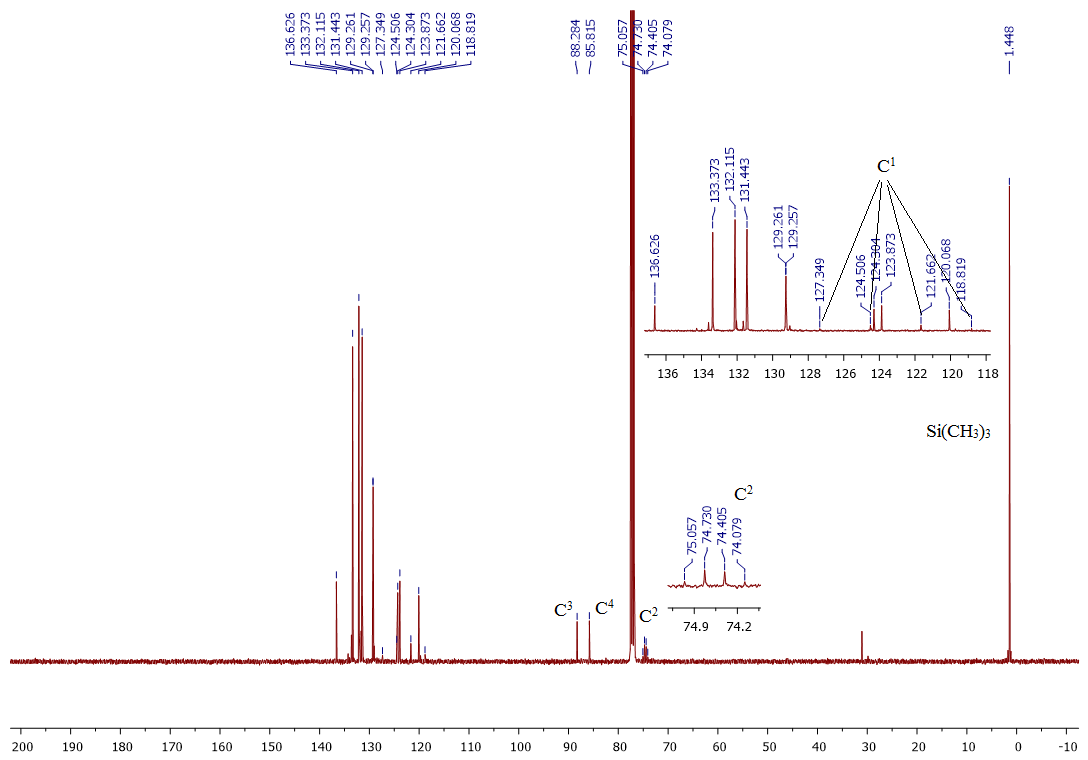


Figure S54. ^13^C-NMR spectrum of the compound **Ir** (CDCl_3_, 100 MHz).


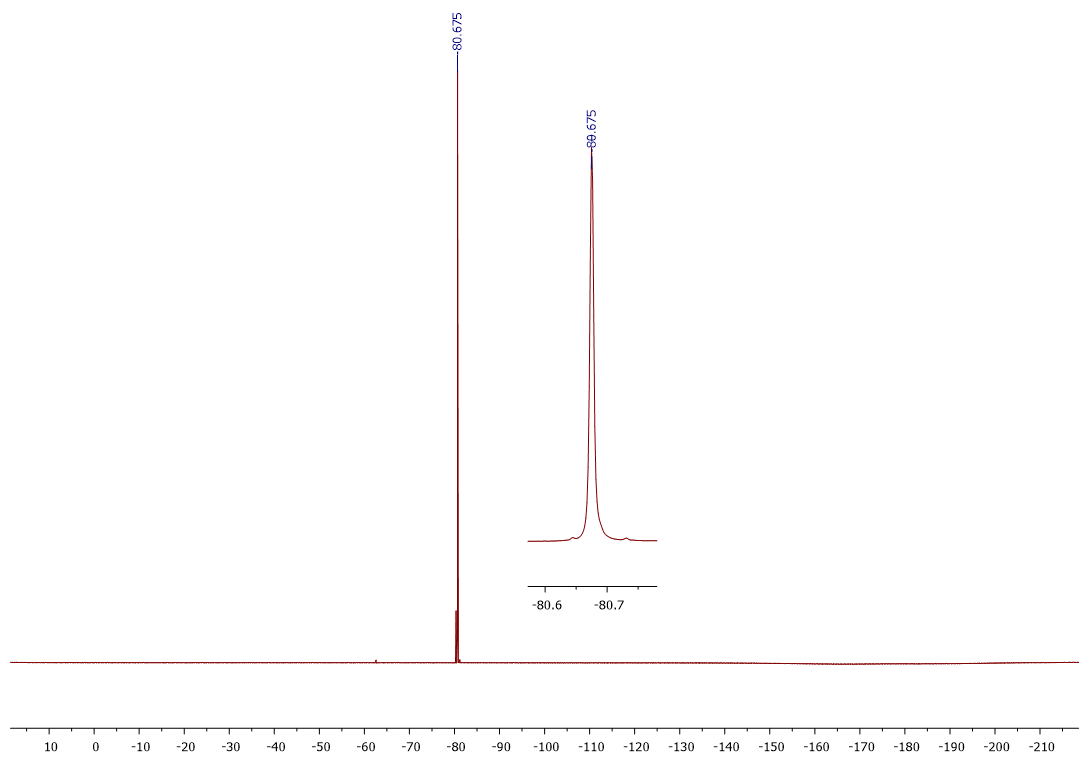


Figure S55. ^19^F-NMR spectrum of the compound **Ir** (CDCl_3_, 376 MHz).


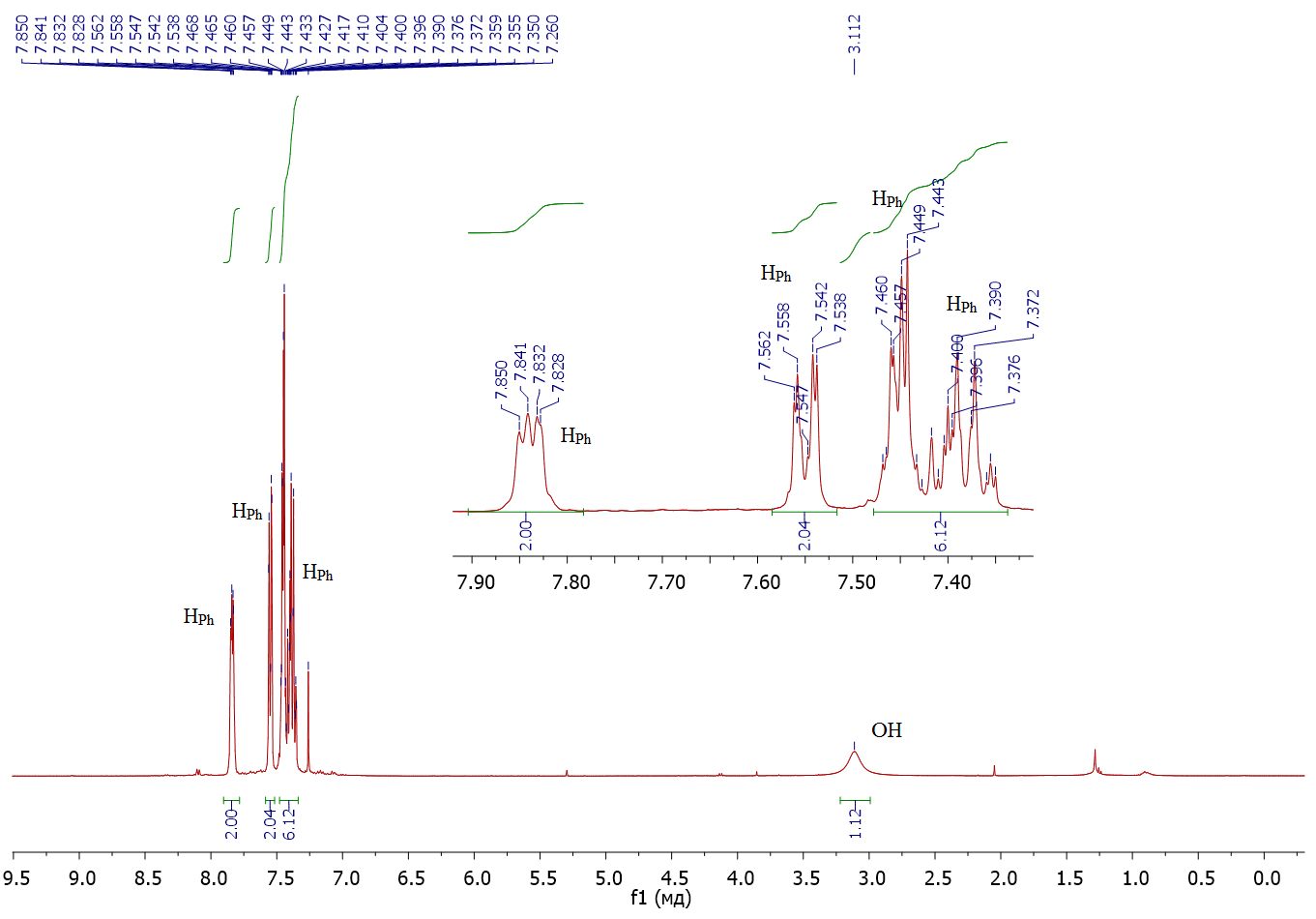


Figure S56. ^1^Н-NMR spectrum of the compound **1a** (CDCl_3_, 400 MHz).


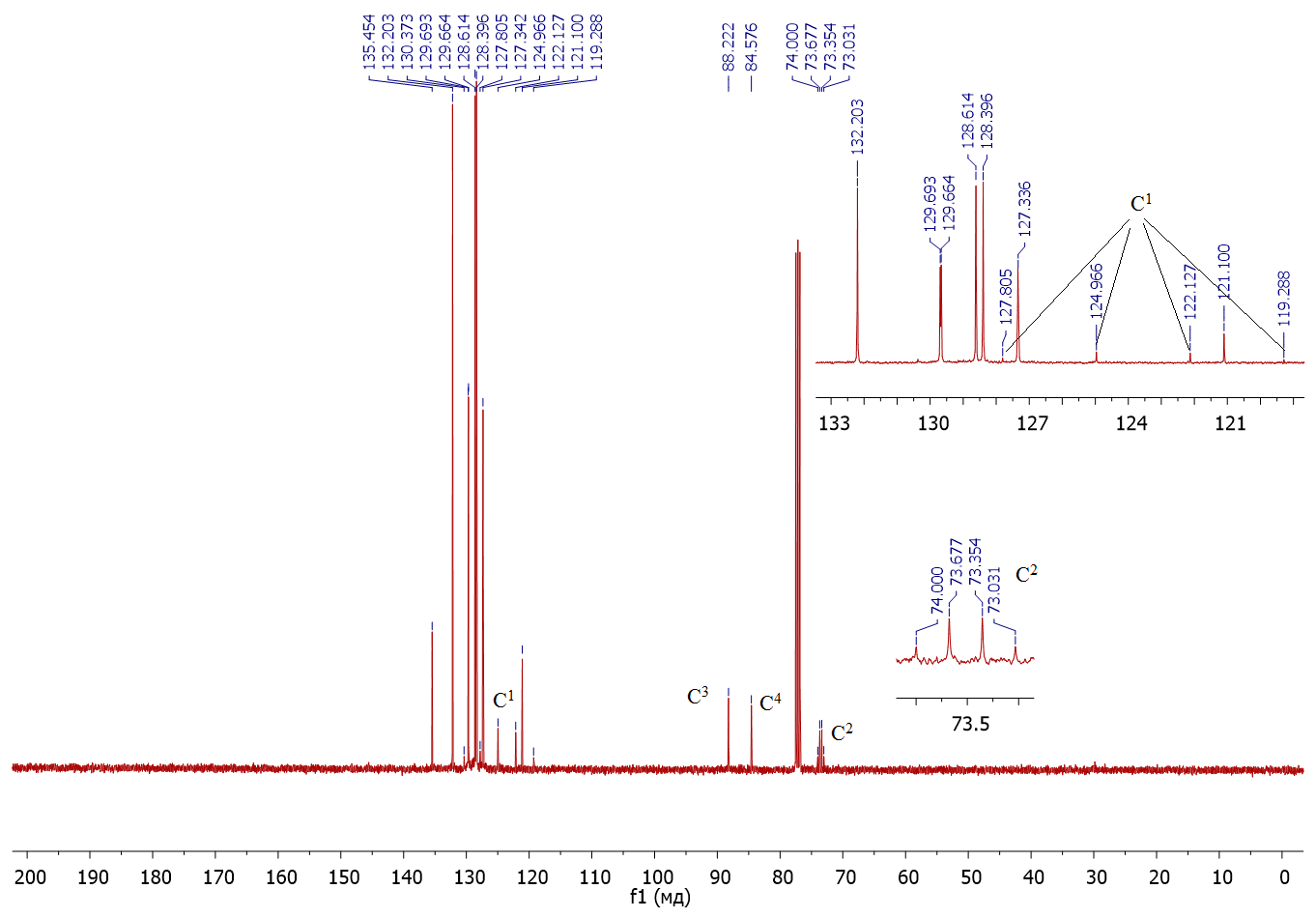


Figure S57. ^13^C-NMR spectrum of the compound **1a** (CDCl_3_, 100 MHz).


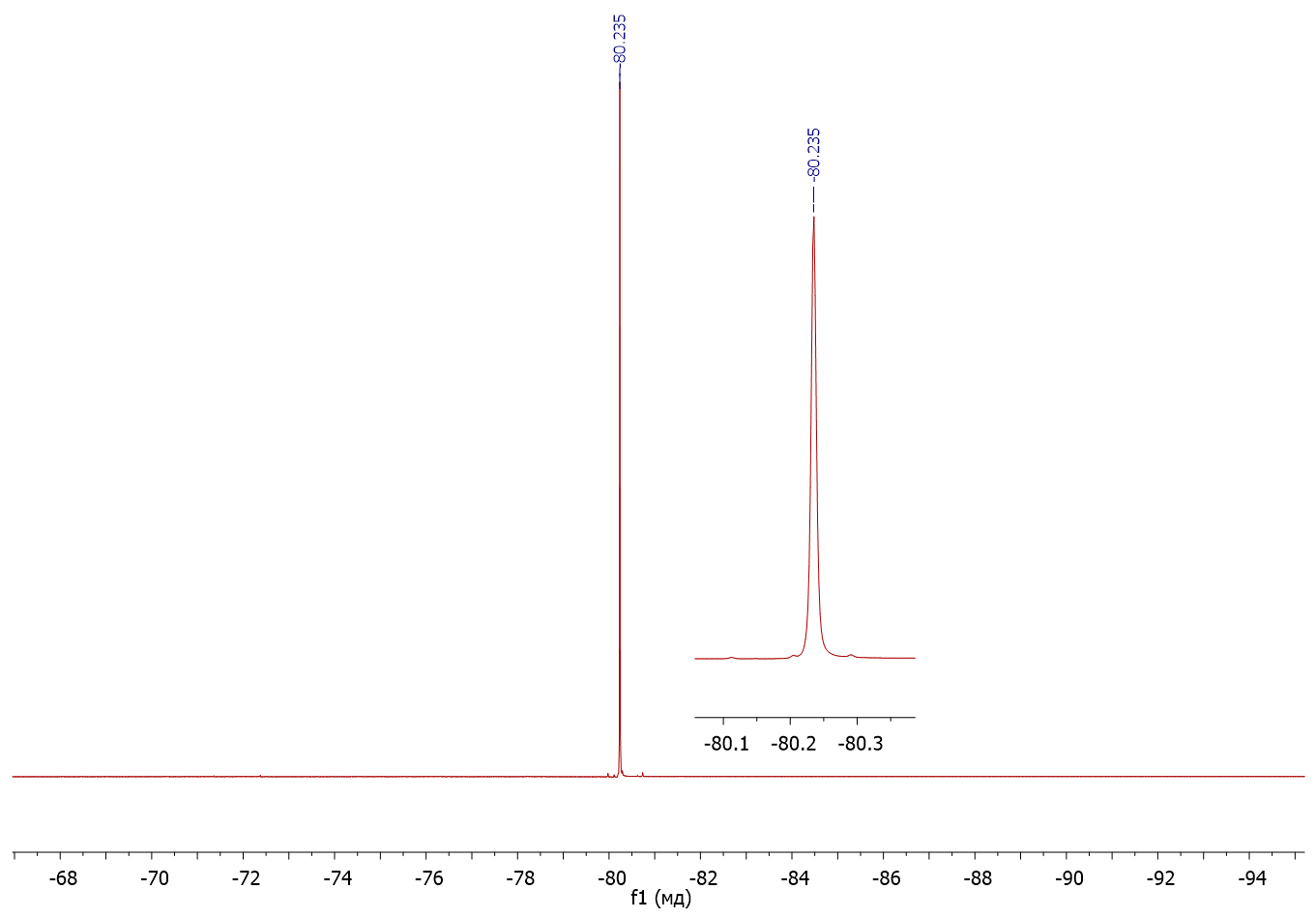


Figure S58. ^19^F-NMR spectrum of the compound **1a** (CDCl_3_, 376 MHz).


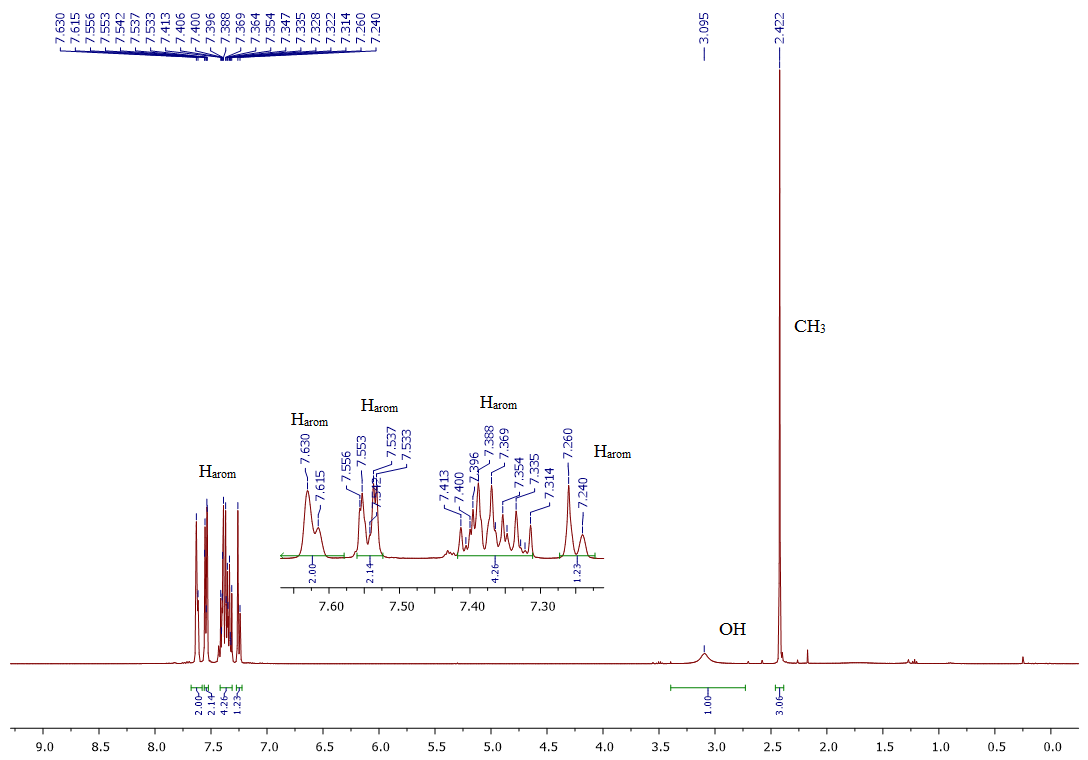


Figure S59. ^1^Н-NMR spectrum of the compound **1b** (CDCl_3_, 400 MHz).


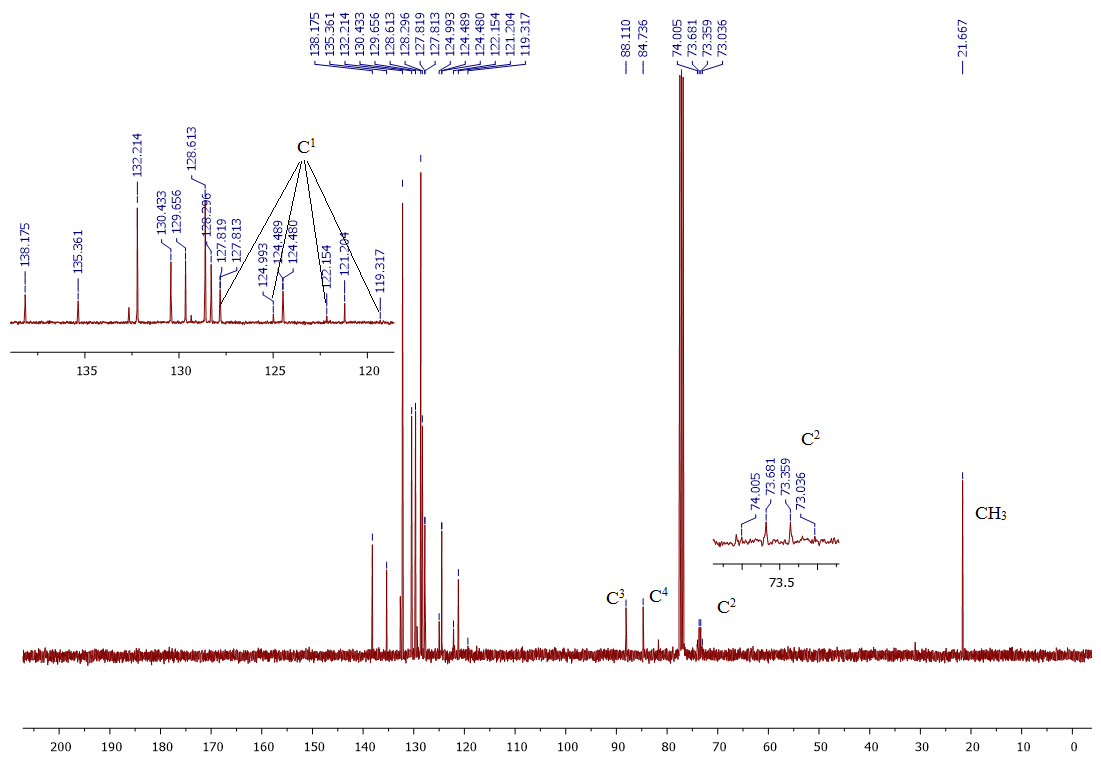


Figure S60. ^13^C-NMR spectrum of the compound **1b** (CDCl_3_, 100 MHz).


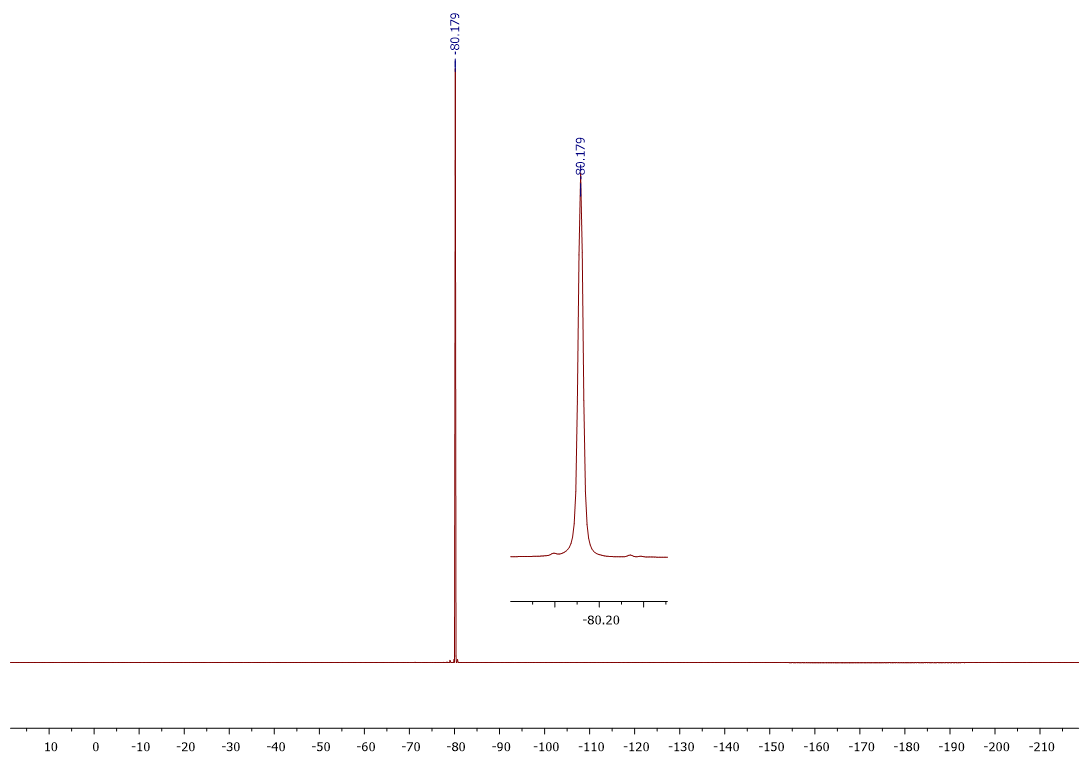


Figure S61. ^19^F-NMR spectrum of the compound **1b** (CDCl_3_, 376 MHz).


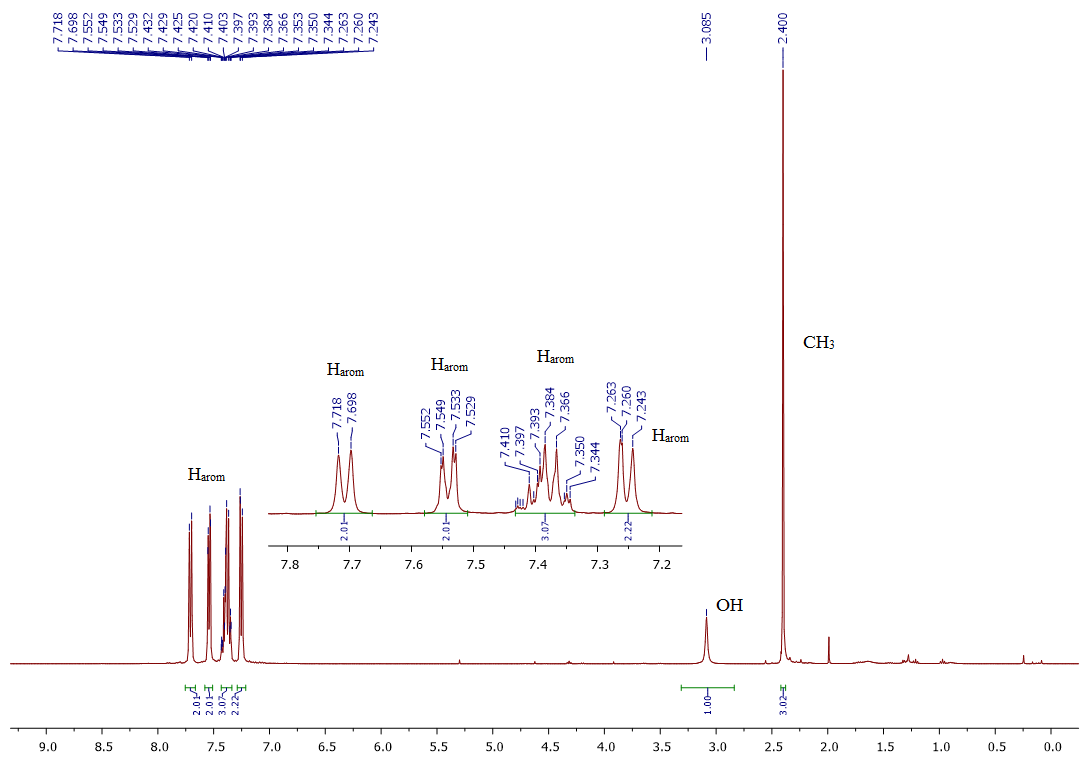


Figure S62. ^1^Н-NMR spectrum of the compound **1c** (CDCl_3_, 400 MHz).


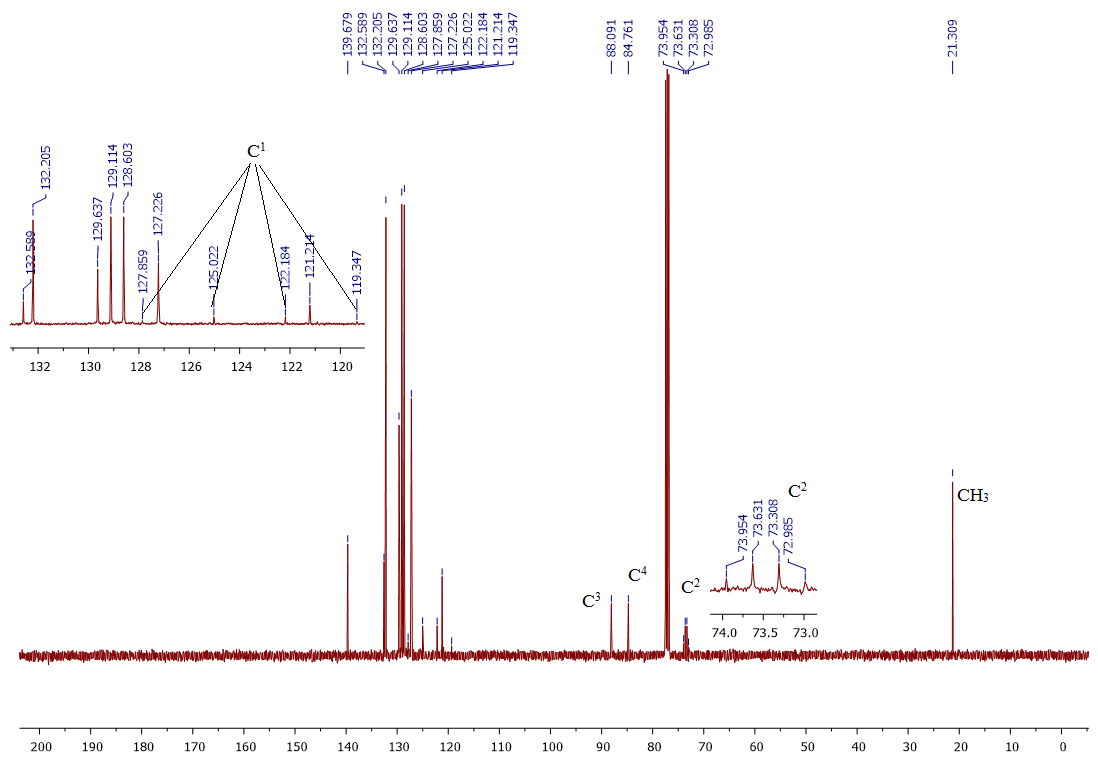


Figure S63. ^13^C-NMR spectrum of the compound **1c** (CDCl_3_, 100 MHz).


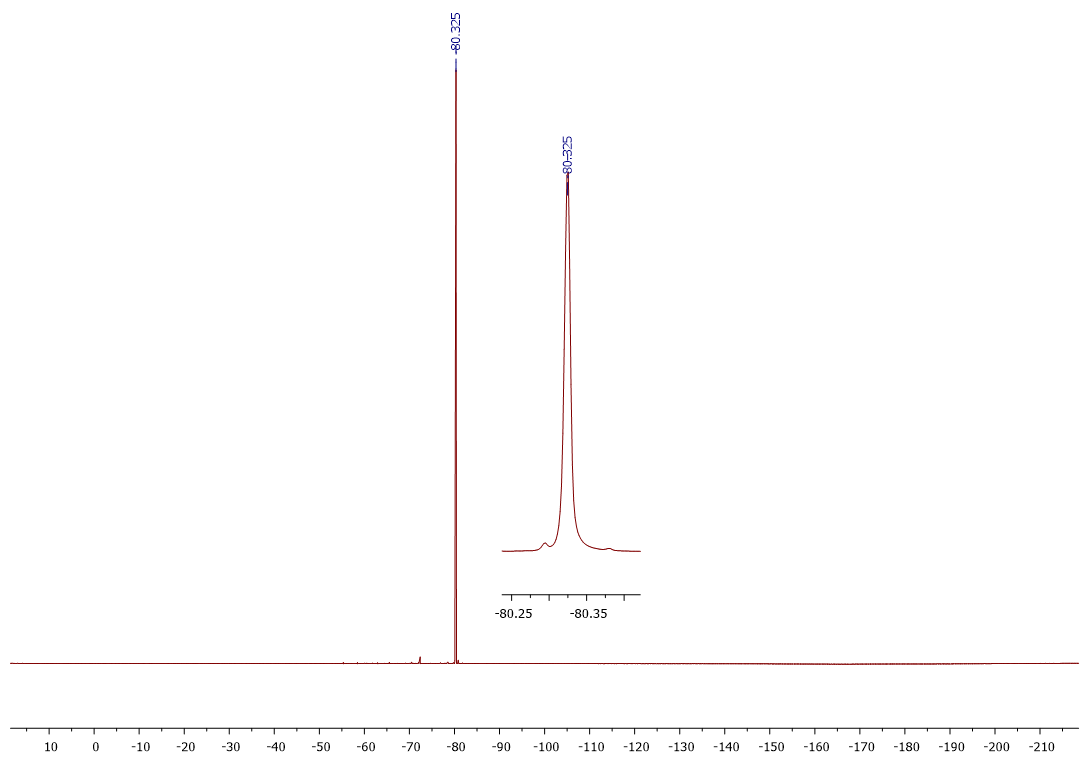


Figure S64. ^19^F-NMR spectrum of the compound **1c** (CDCl_3_, 376 MHz).


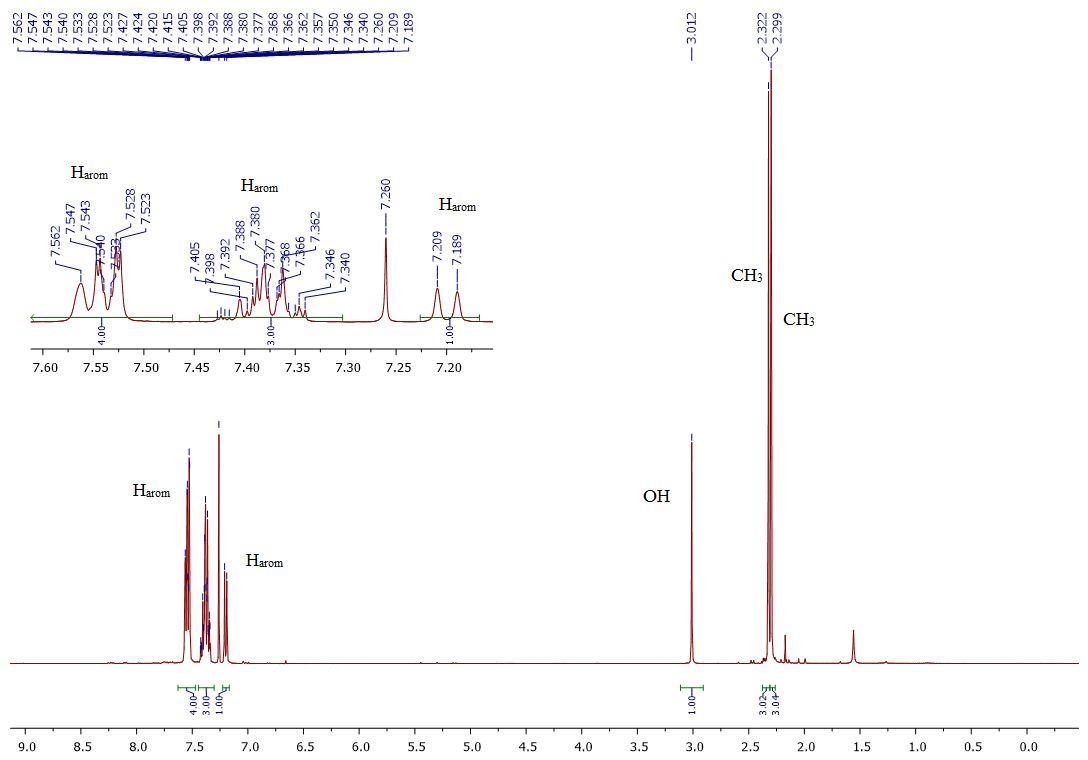


Figure S65. ^1^Н-NMR spectrum of the compound **1d** (CDCl_3_, 400 MHz).


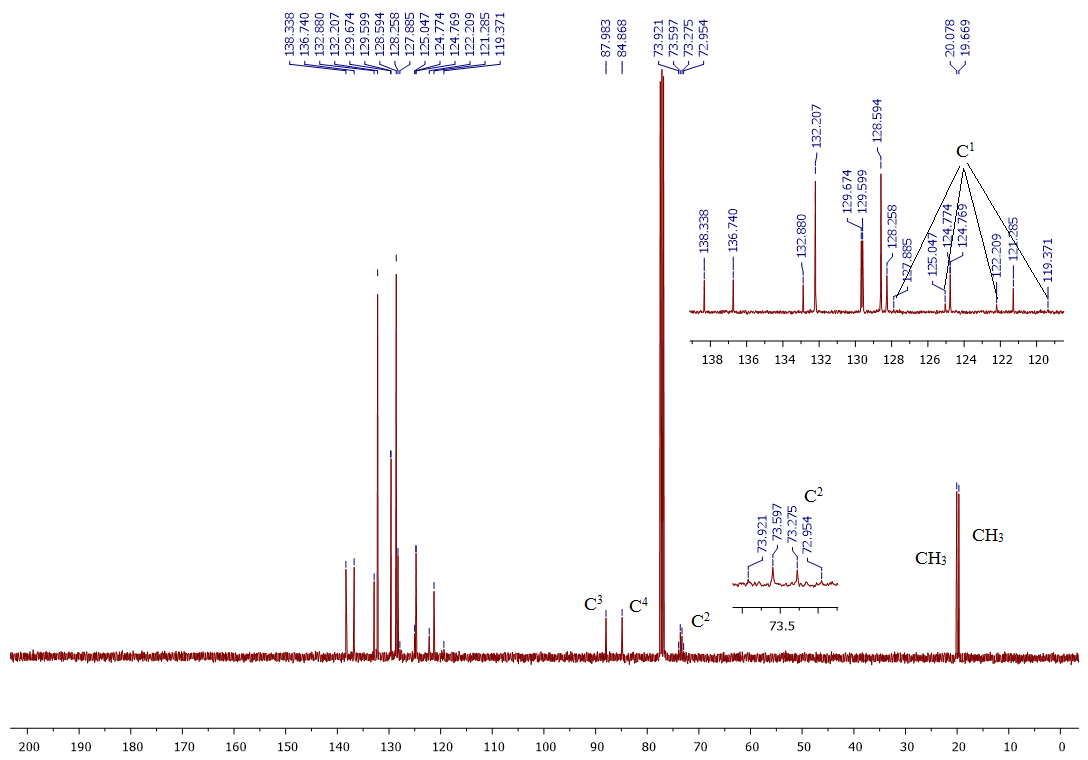


Figure S66. ^13^C-NMR spectrum of the compound **1d** (CDCl_3_, 100 MHz).


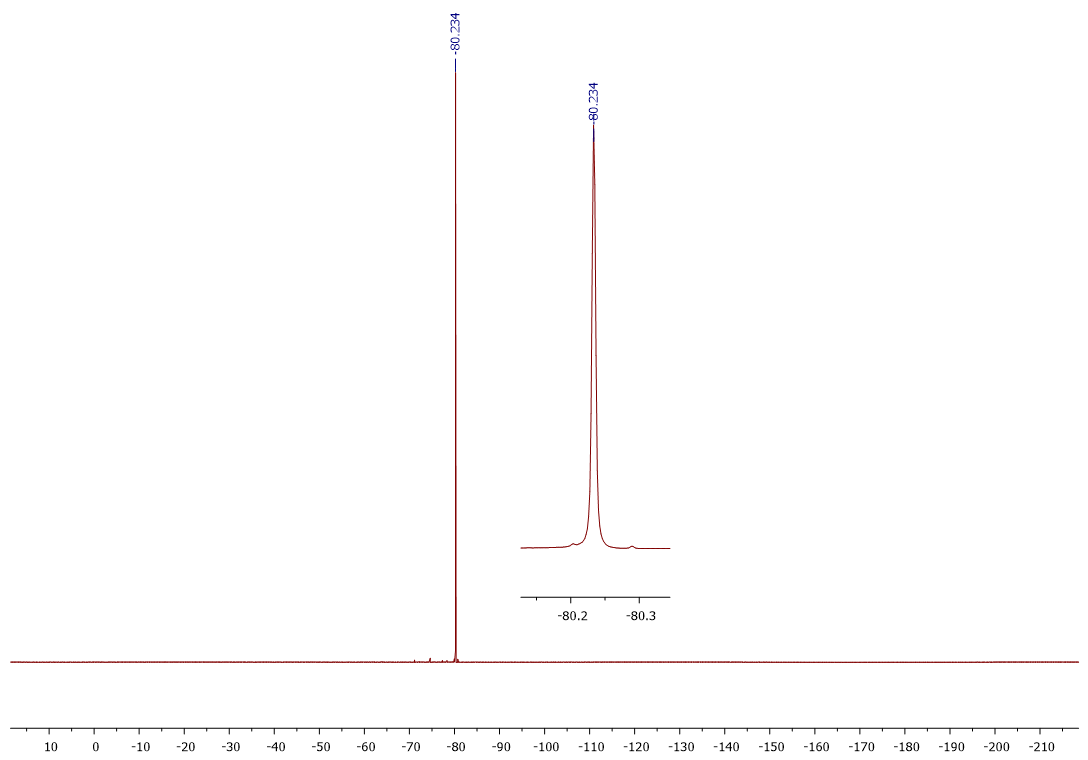


Figure S67. ^19^F-NMR spectrum of the compound **1d** (CDCl_3_, 376 MHz).


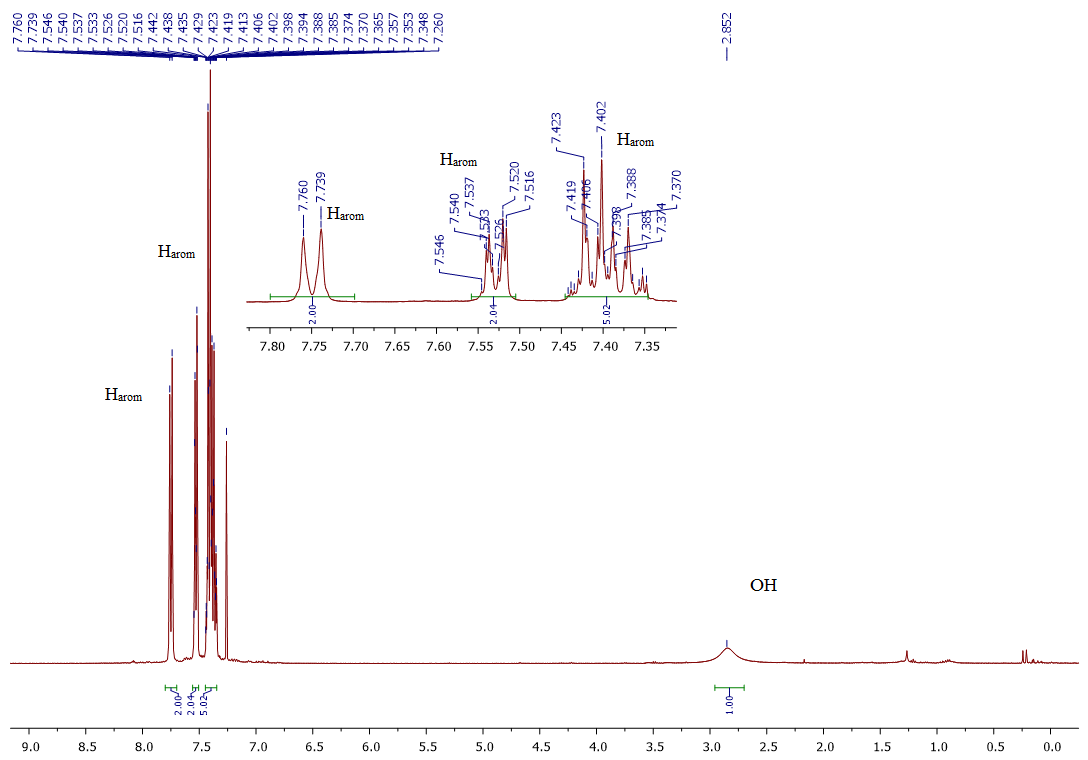


Figure S68. ^1^Н-NMR spectrum of the compound **1e** (CDCl_3_, 400 MHz).


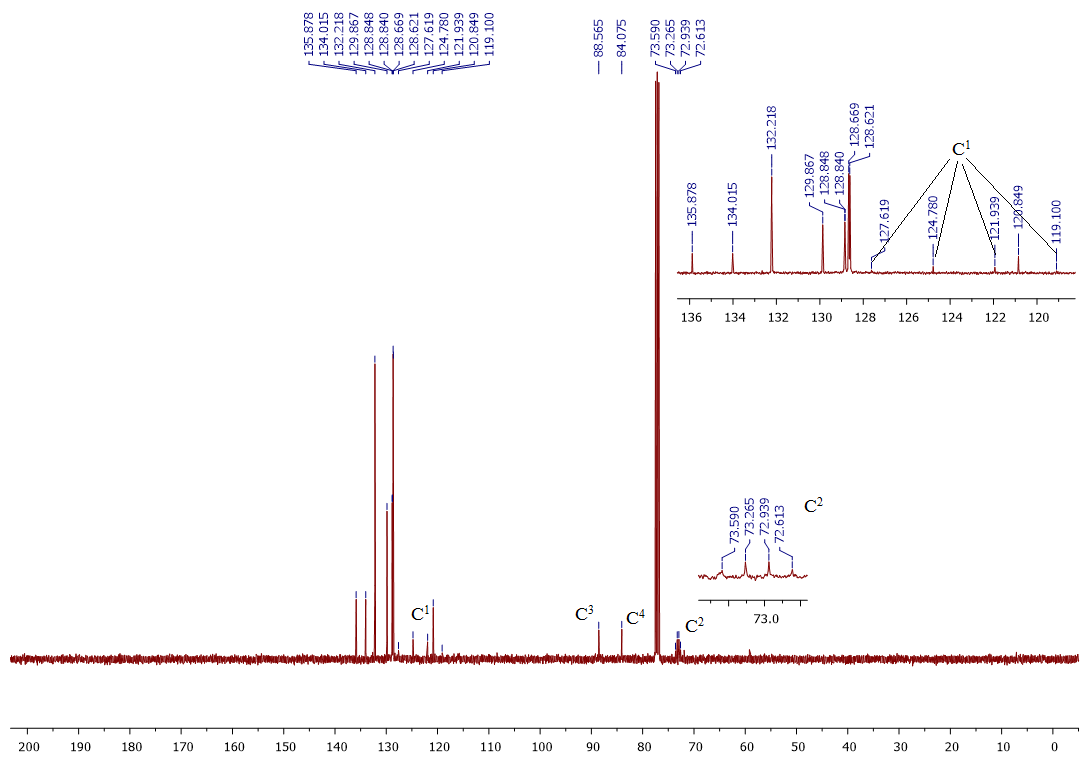


Figure S69. ^13^C-NMR spectrum of the compound **1e** (CDCl_3_, 100 MHz).


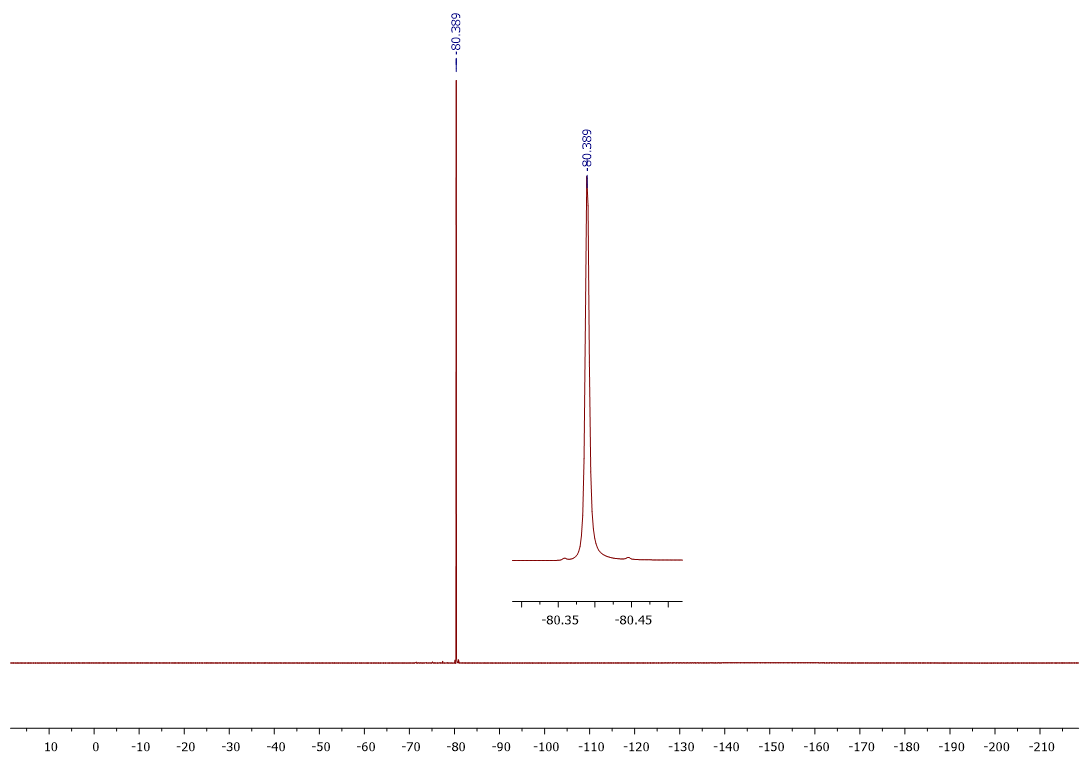


Figure S70. ^19^F-NMR spectrum of the compound **1e** (CDCl_3_, 376 MHz).


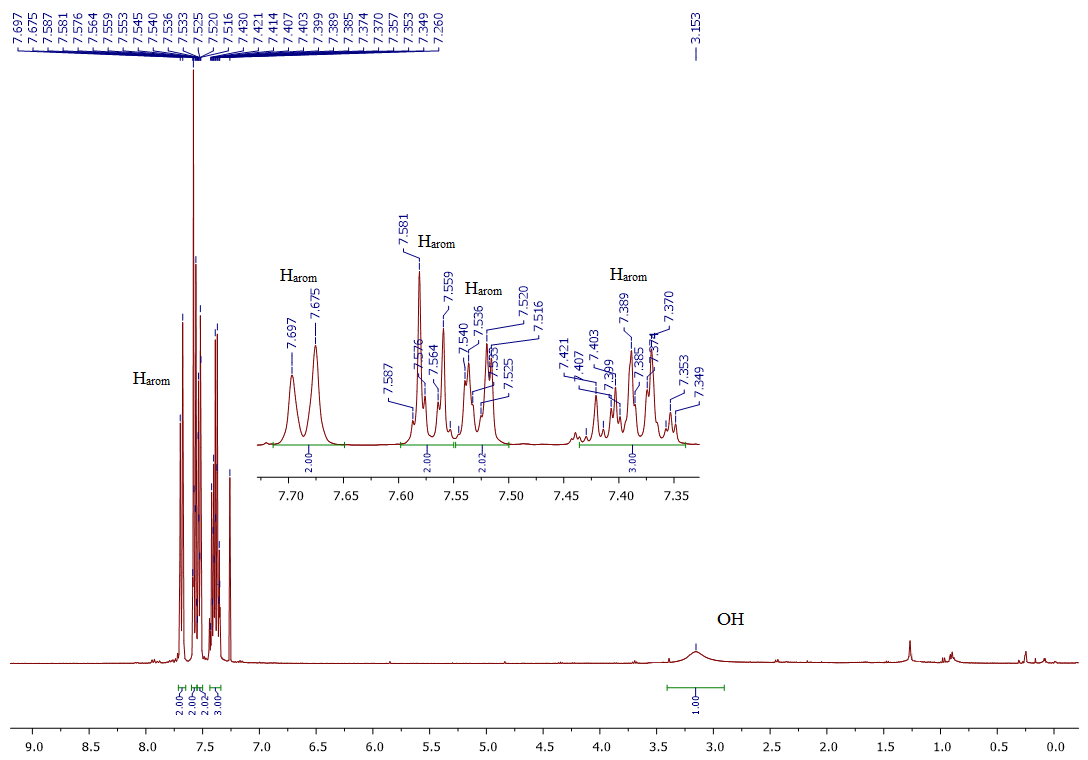


Figure S71. ^1^Н-NMR spectrum of the compound **1f** (CDCl_3_, 400 MHz).


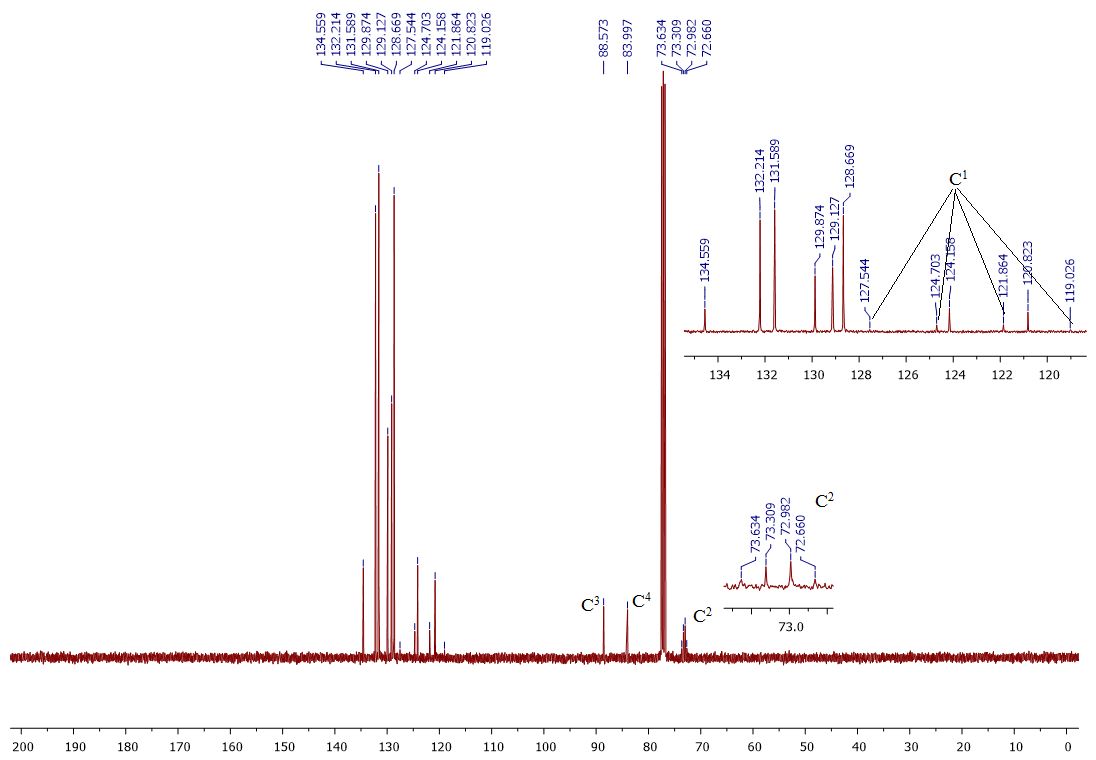


Figure S72. ^13^C-NMR spectrum of the compound **1f** (CDCl_3_, 100 MHz).


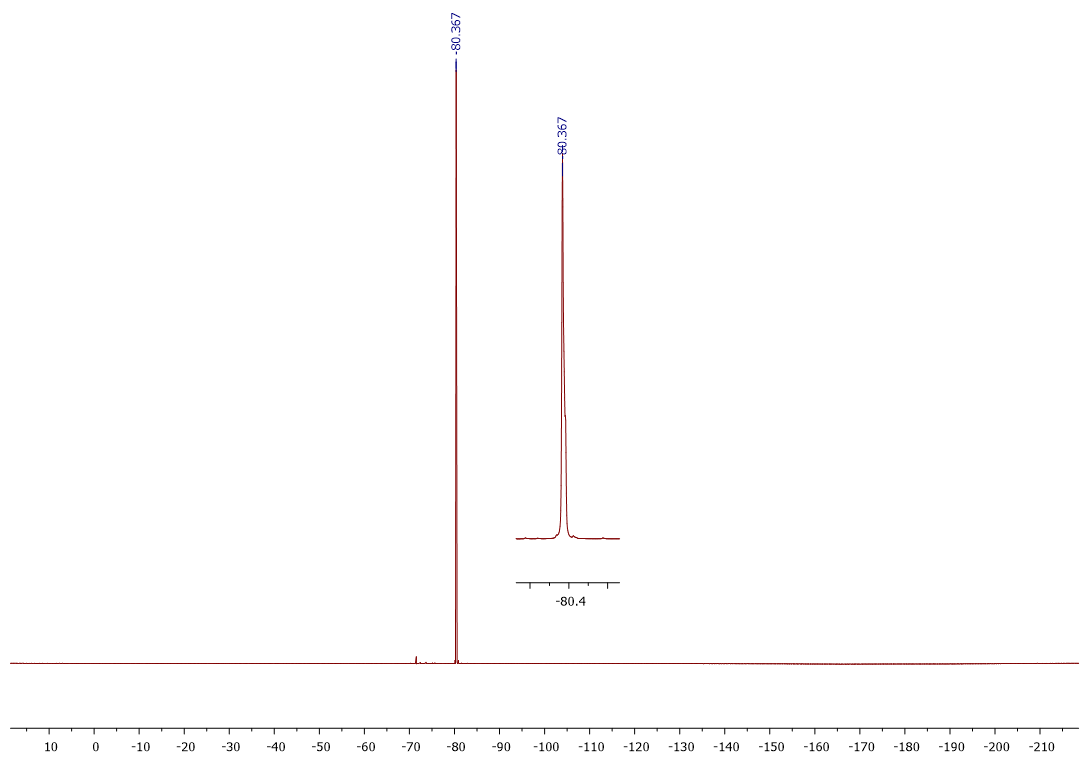


Figure S73. ^19^F-NMR spectrum of the compound **1f** (CDCl_3_, 376 MHz).


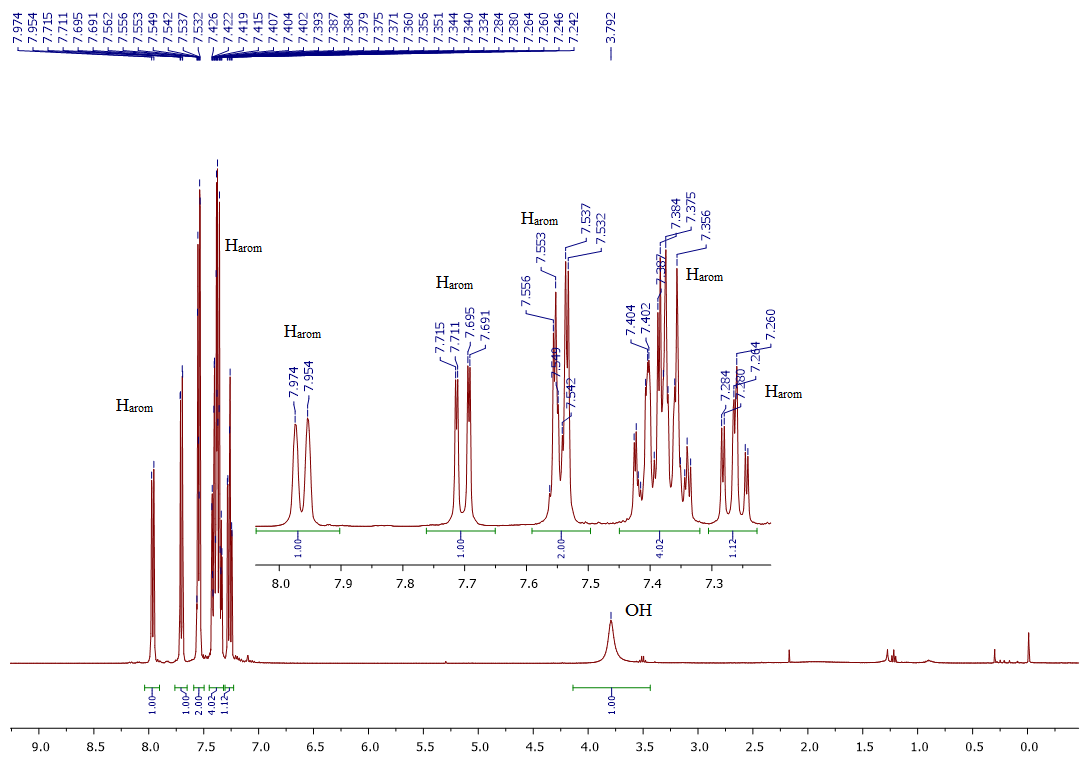


Figure S74. ^1^Н-NMR spectrum of the compound **1g** (CDCl_3_, 400 MHz).


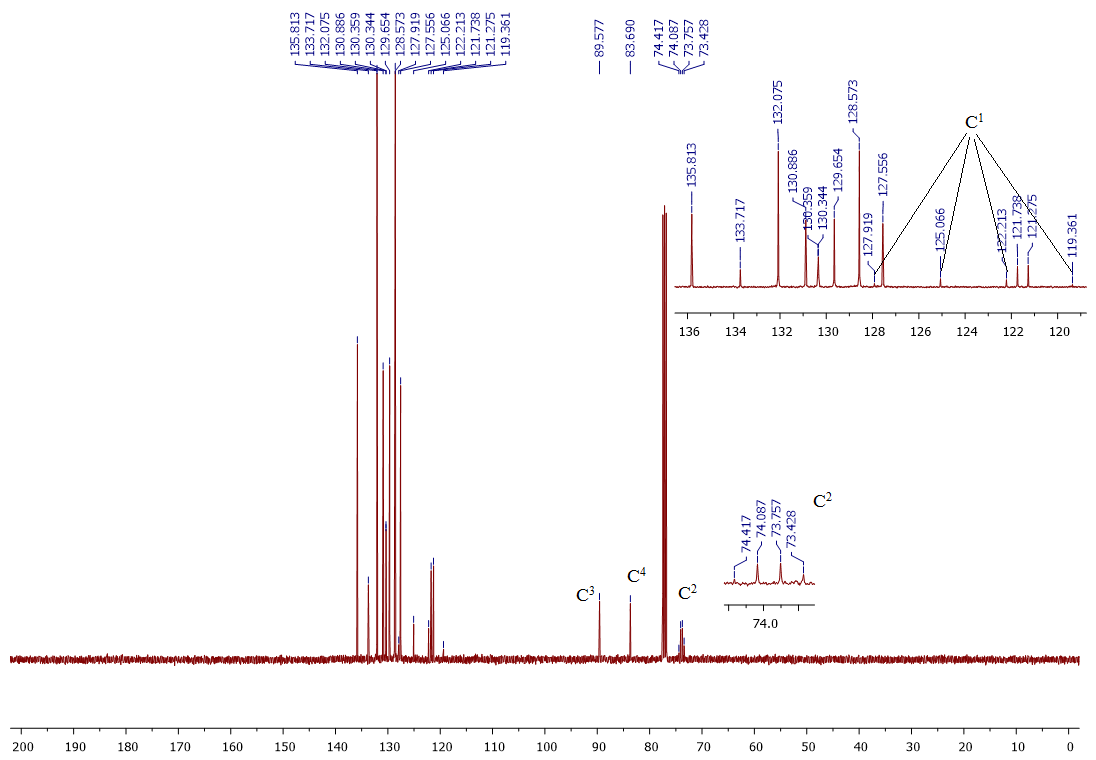


Figure S75. ^13^C-NMR spectrum of the compound **1g** (CDCl_3_, 100 MHz).


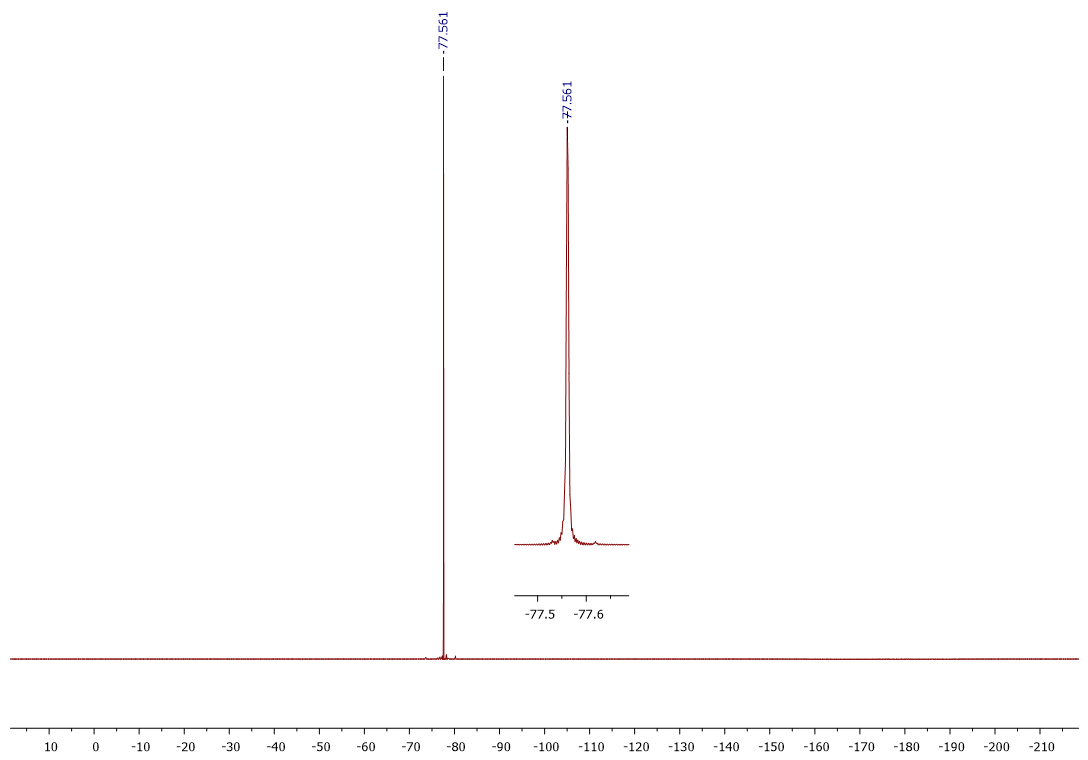


Figure S76. ^19^F-NMR spectrum of the compound **1g** (CDCl_3_, 376 MHz).


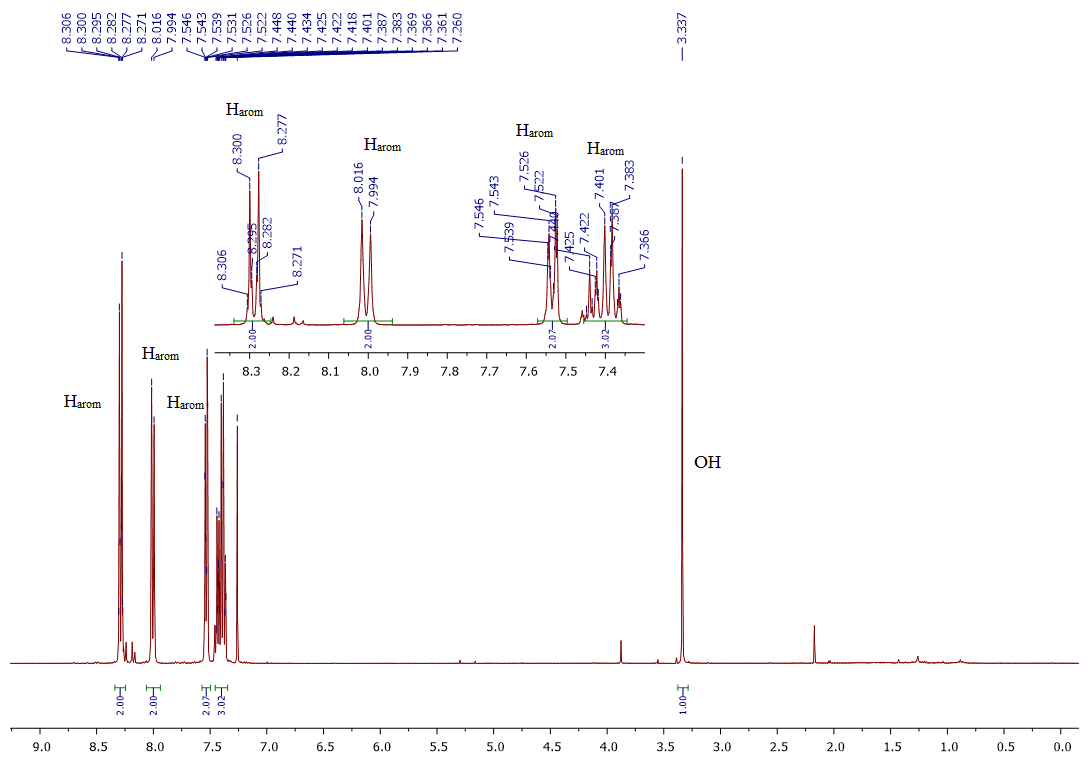


Figure S77. ^1^Н-NMR spectrum of the compound **1h** (CDCl_3_, 400 MHz).


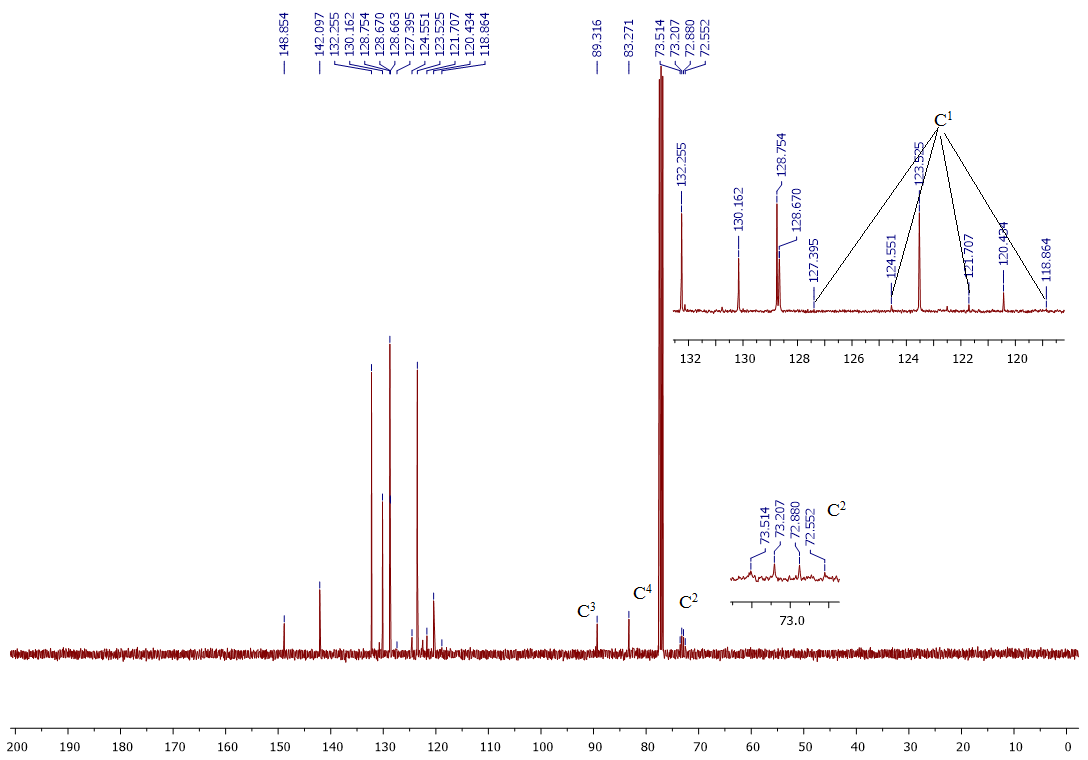


Figure S78. ^13^C-NMR spectrum of the compound **1h** (CDCl_3_, 100 MHz).


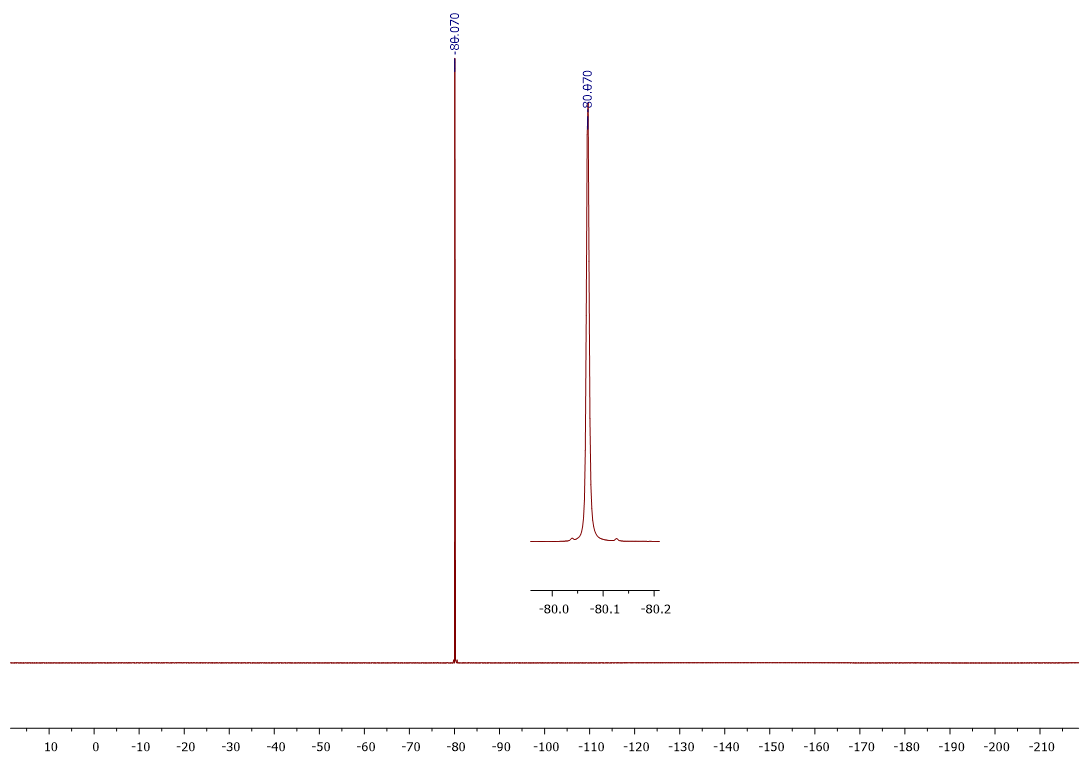


Figure S79. ^19^F-NMR spectrum of the compound **1h** (CDCl_3_, 376 MHz).


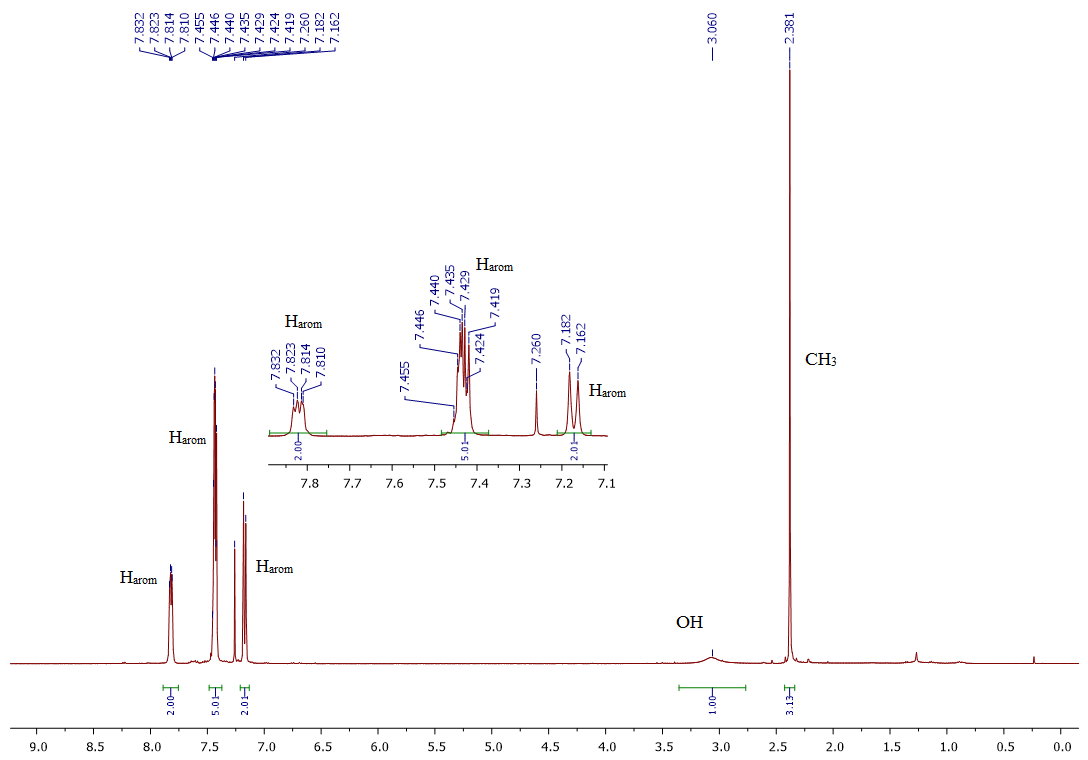


Figure S80. ^1^Н-NMR spectrum of the compound **1i** (CDCl_3_, 400 MHz).


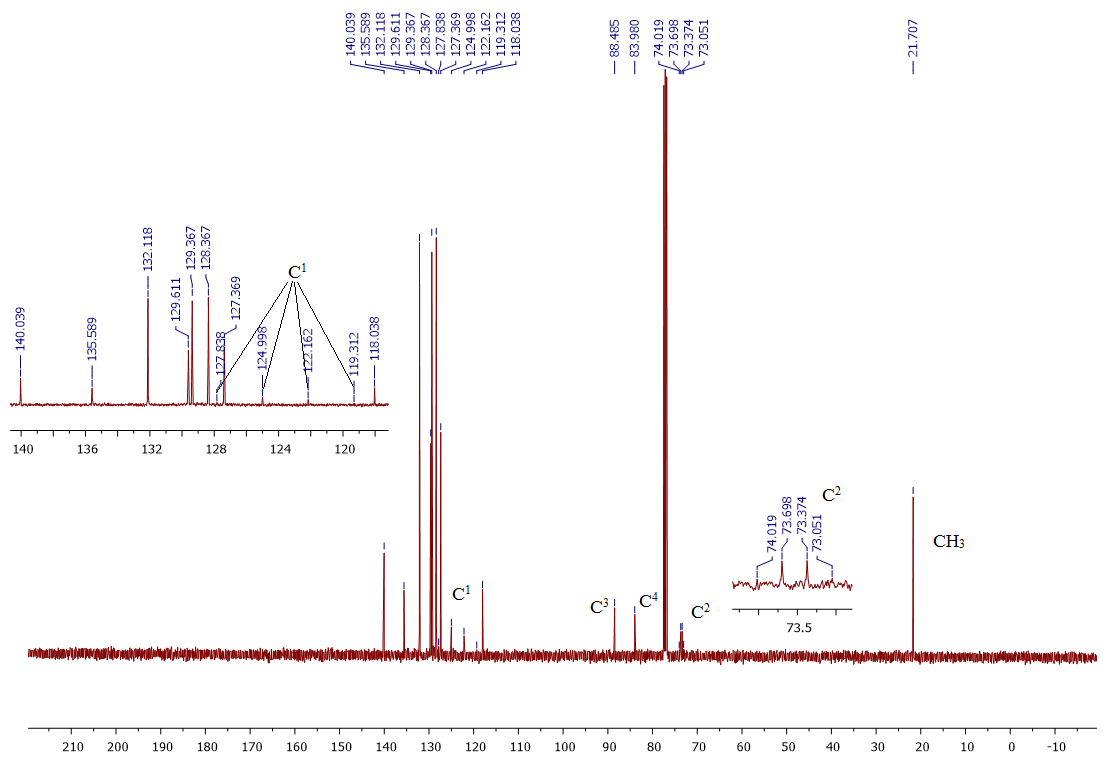


Figure S81. ^13^C-NMR spectrum of the compound **1i** (CDCl_3_, 100 MHz).


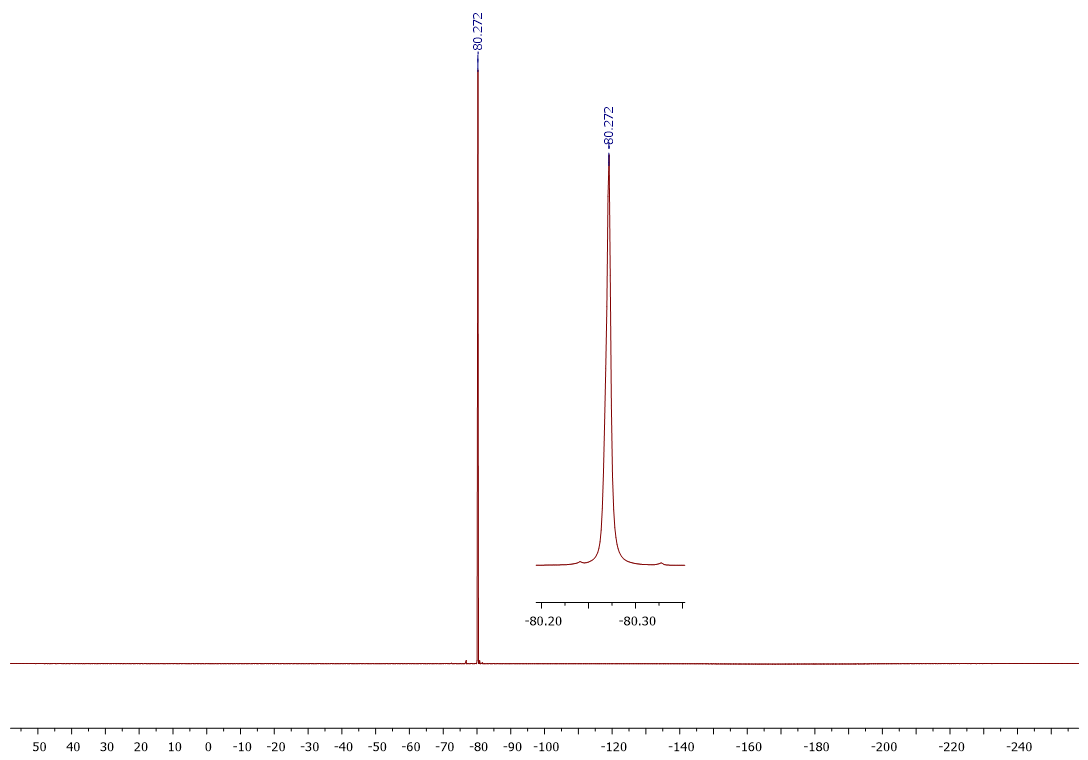


Figure S82. ^19^F-NMR spectrum of the compound **1i** (CDCl_3_, 376 MHz).


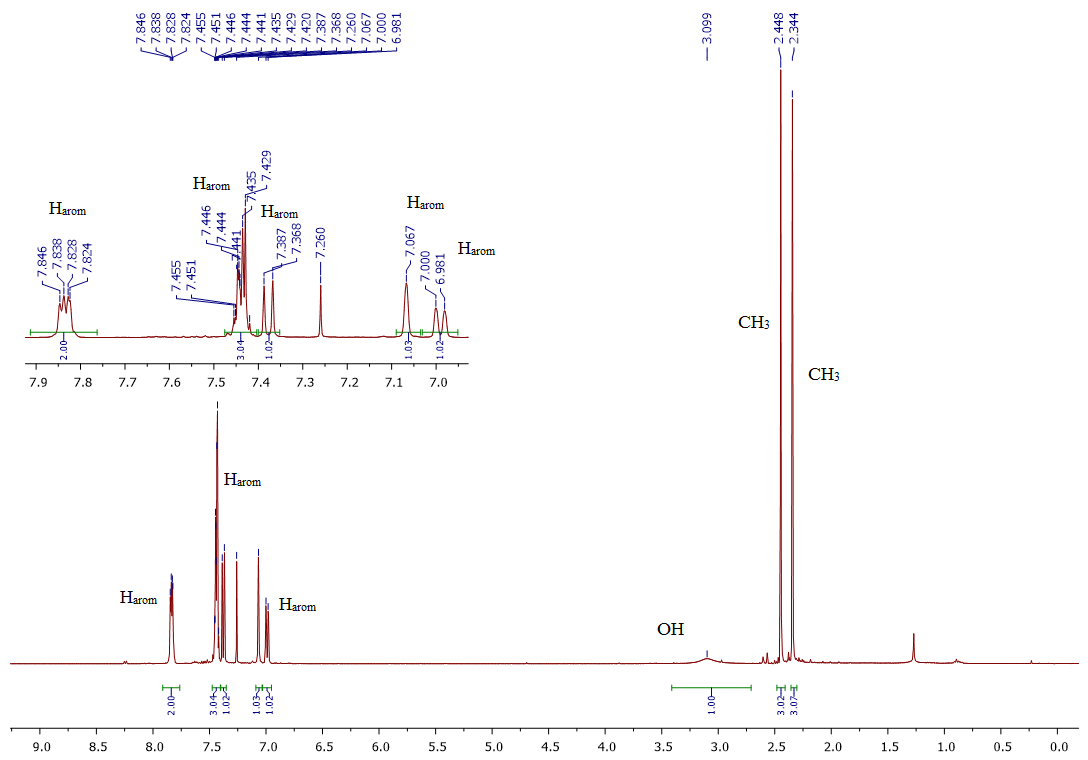


Figure S83. ^1^Н-NMR spectrum of the compound **1j** (CDCl_3_, 400 MHz).


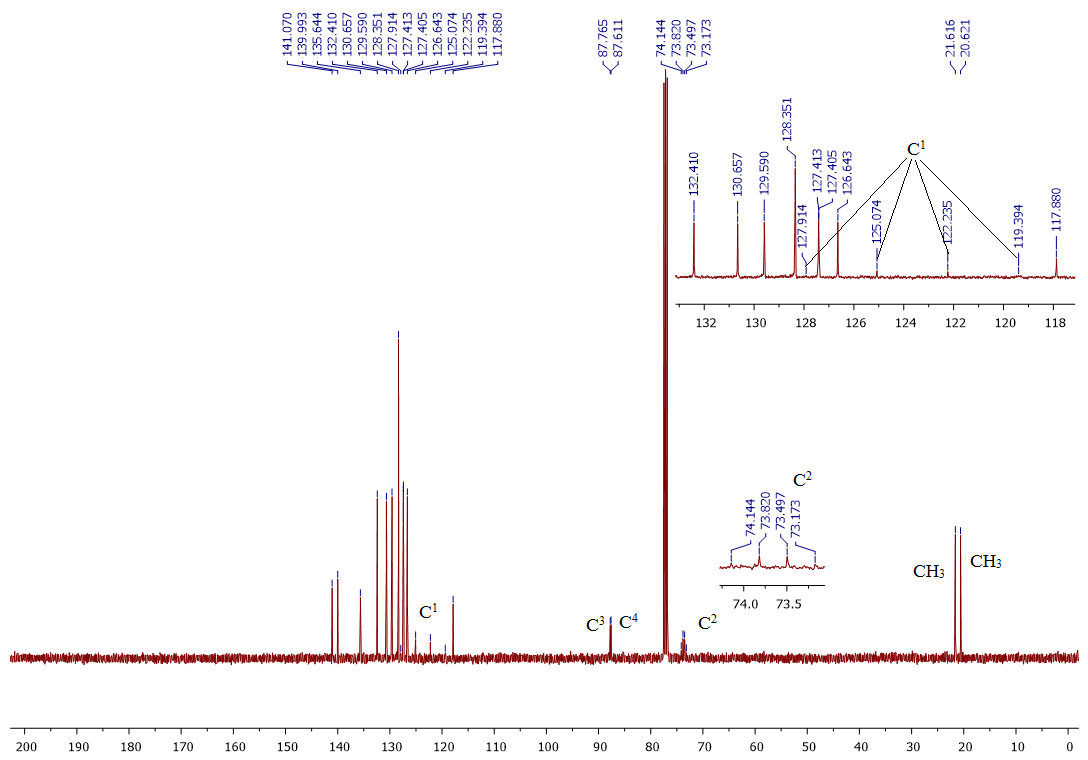


Figure S84. ^13^C-NMR spectrum of the compound **1j** (CDCl_3_, 100 MHz).


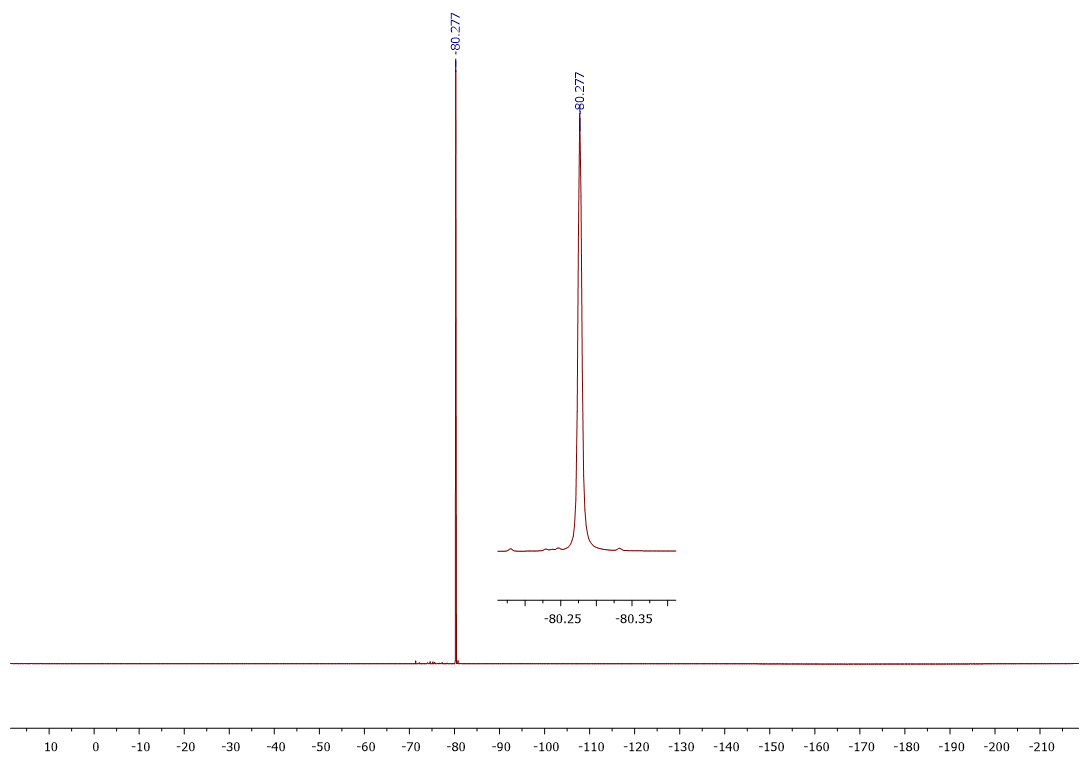


Figure S85. ^19^F-NMR spectrum of the compound **1j** (CDCl_3_, 376 MHz).


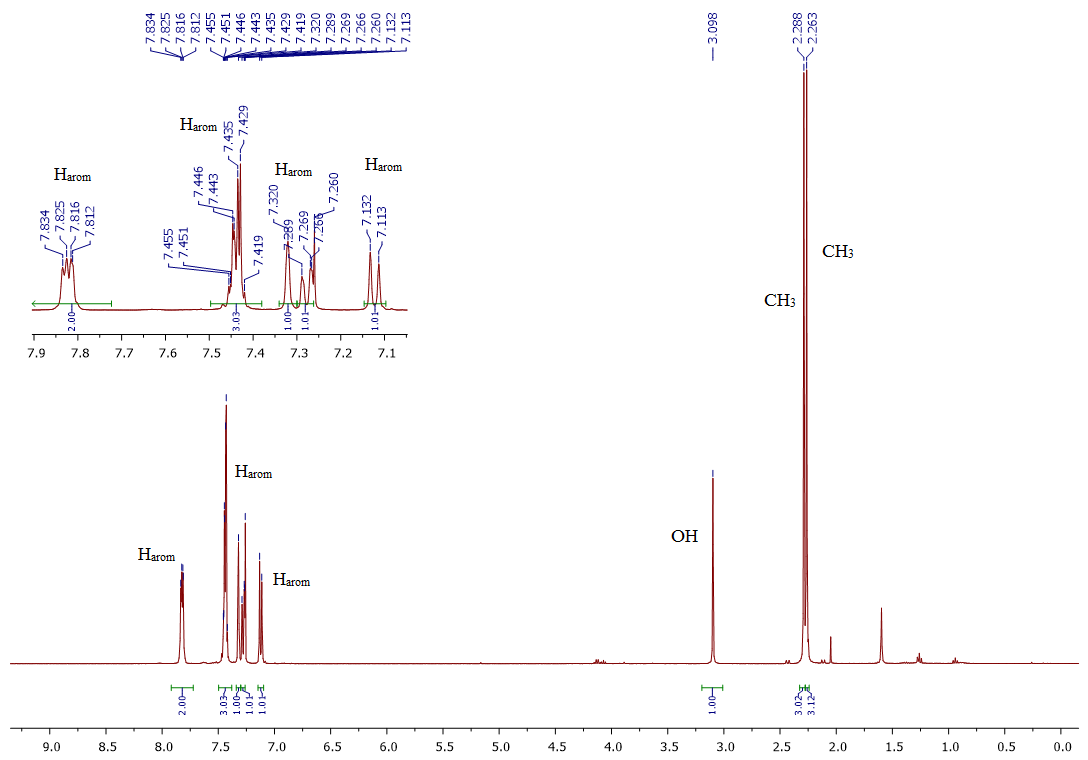


Figure S86. ^1^Н-NMR spectrum of the compound **1k** (CDCl_3_, 400 MHz).


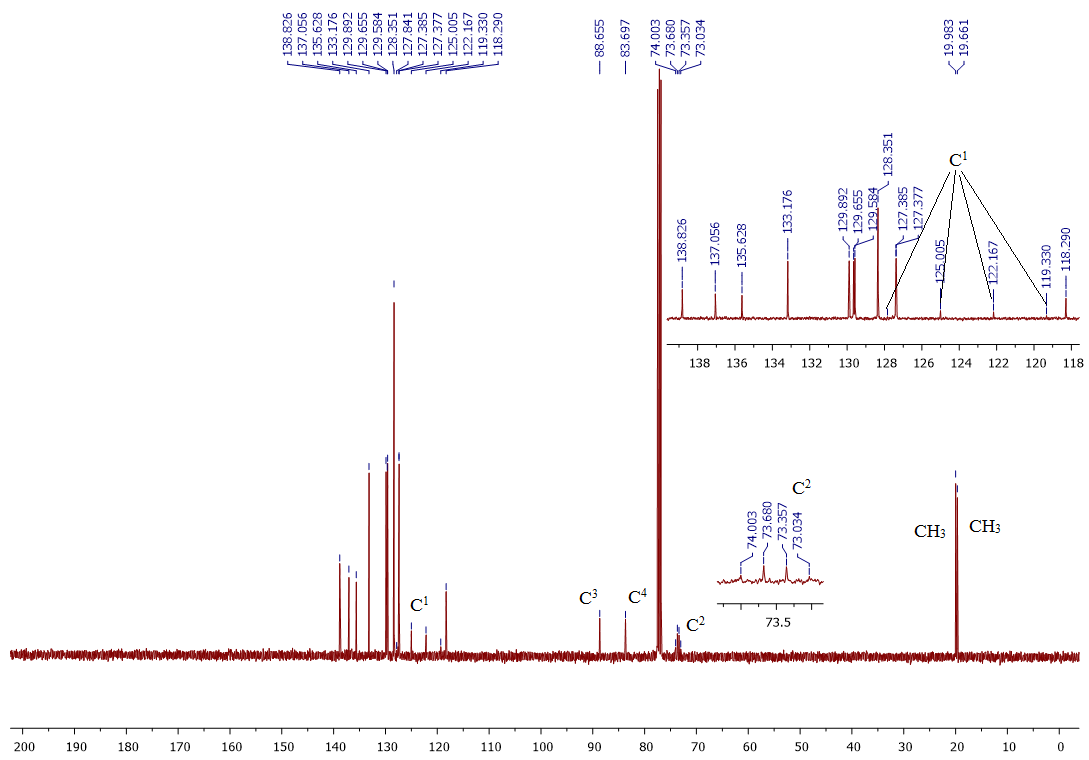


Figure S87. ^13^C-NMR spectrum of the compound **1k** (CDCl_3_, 100 MHz).


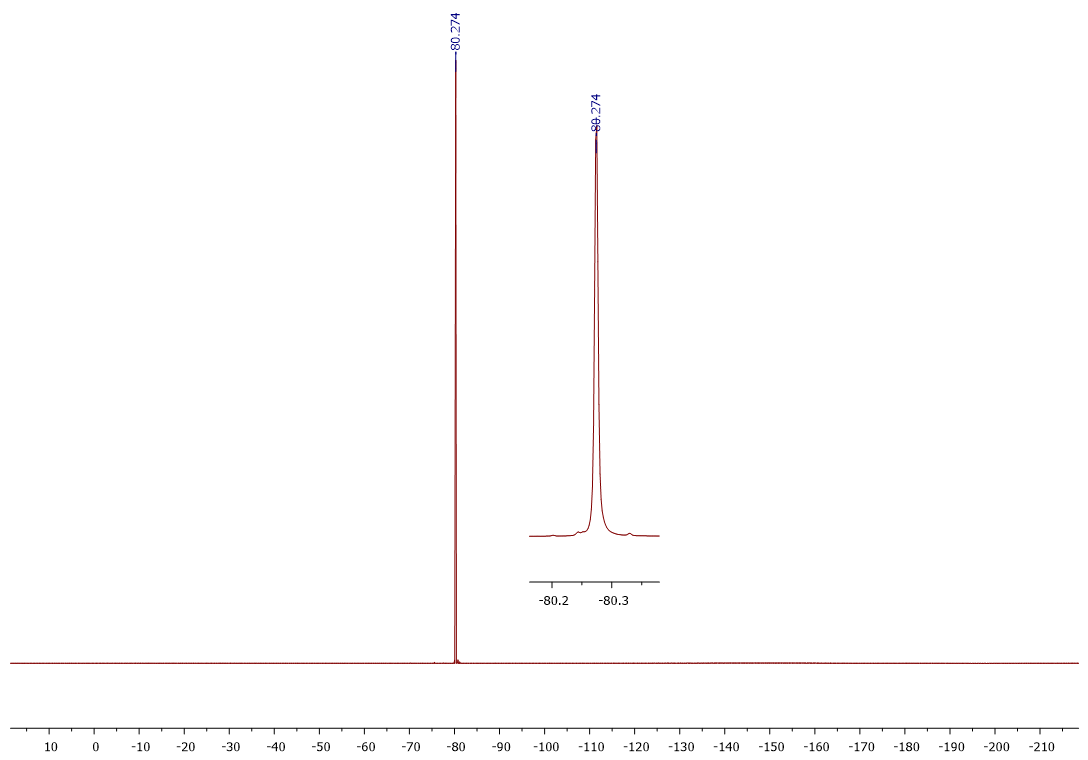


Figure S88. ^19^F-NMR spectrum of the compound **1k** (CDCl_3_, 376 MHz).


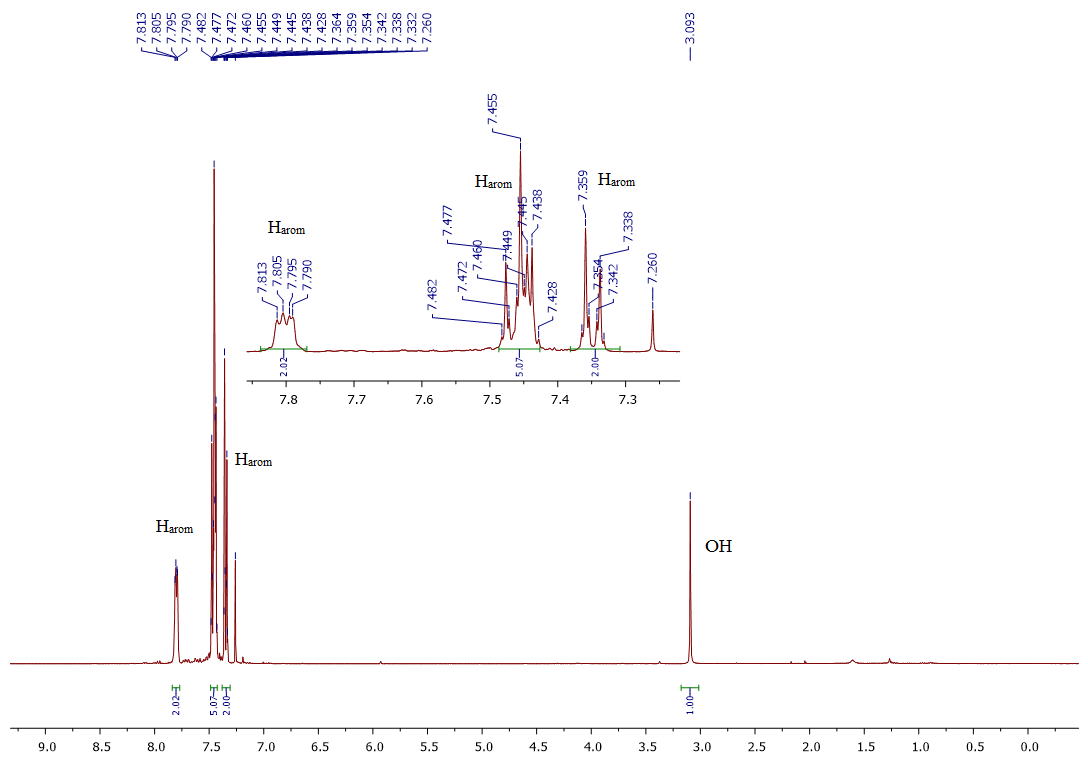


Figure S89. ^1^Н-NMR spectrum of the compound **1l** (CDCl_3_, 400 MHz).


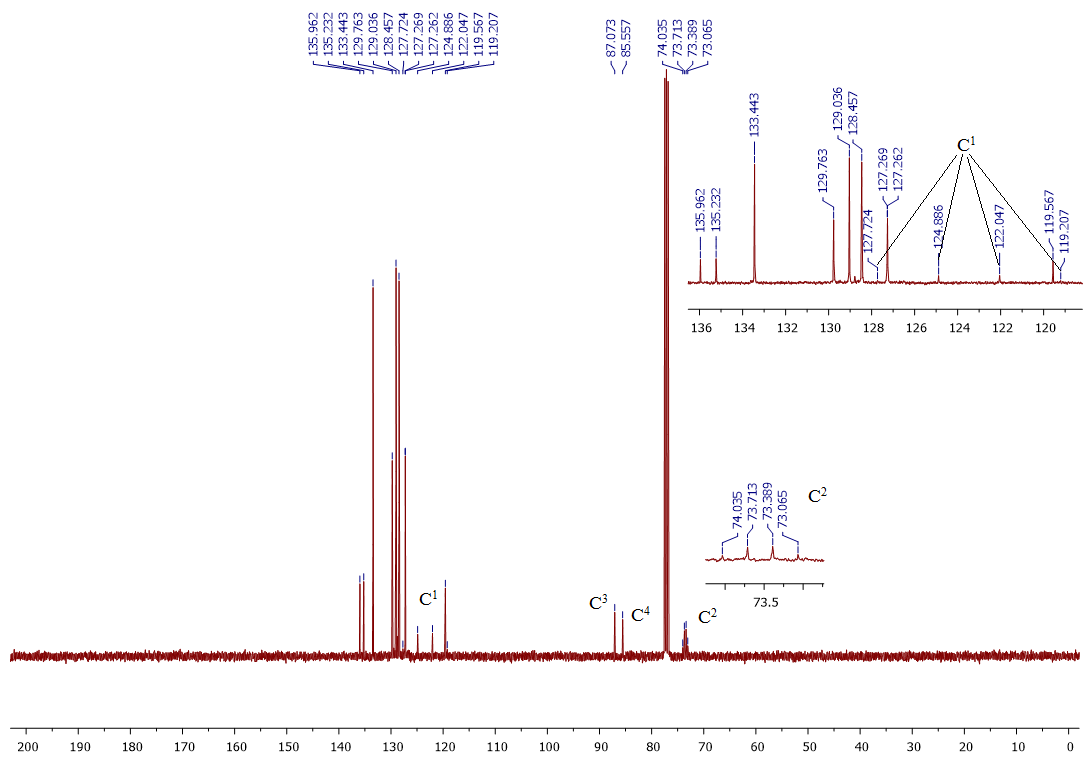


Figure S90. ^13^C-NMR spectrum of the compound **1l** (CDCl_3_, 100 MHz).


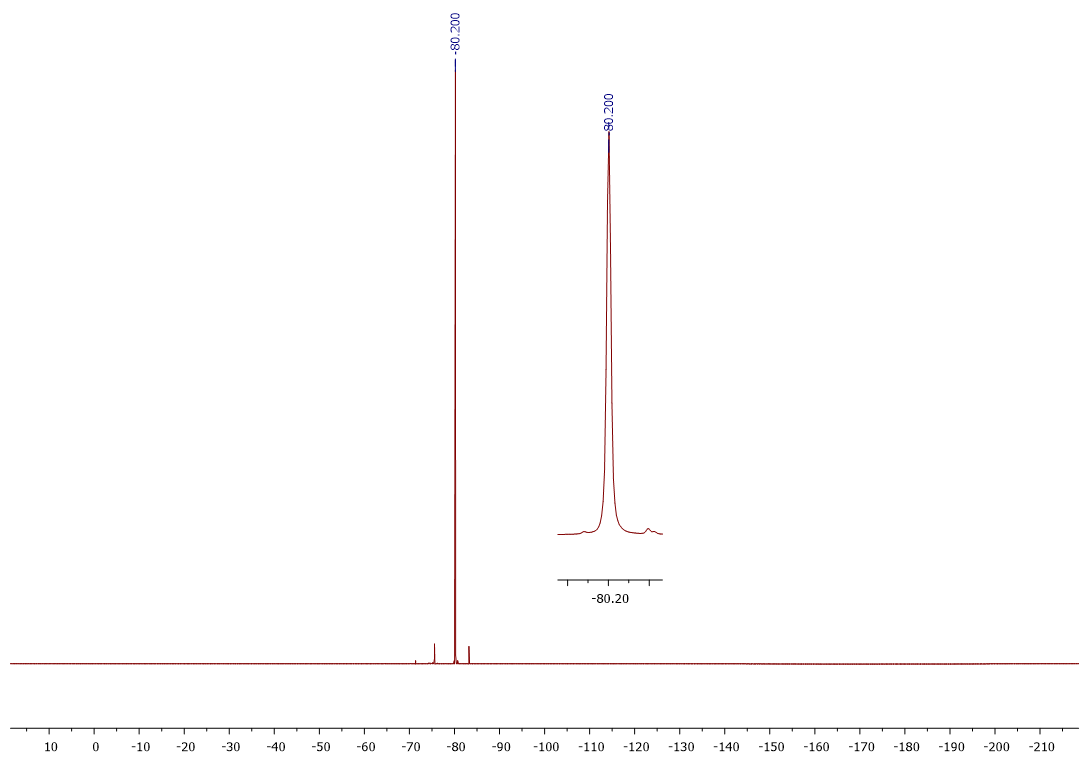


Figure S91. ^19^F-NMR spectrum of the compound **1l** (CDCl_3_, 376 MHz).


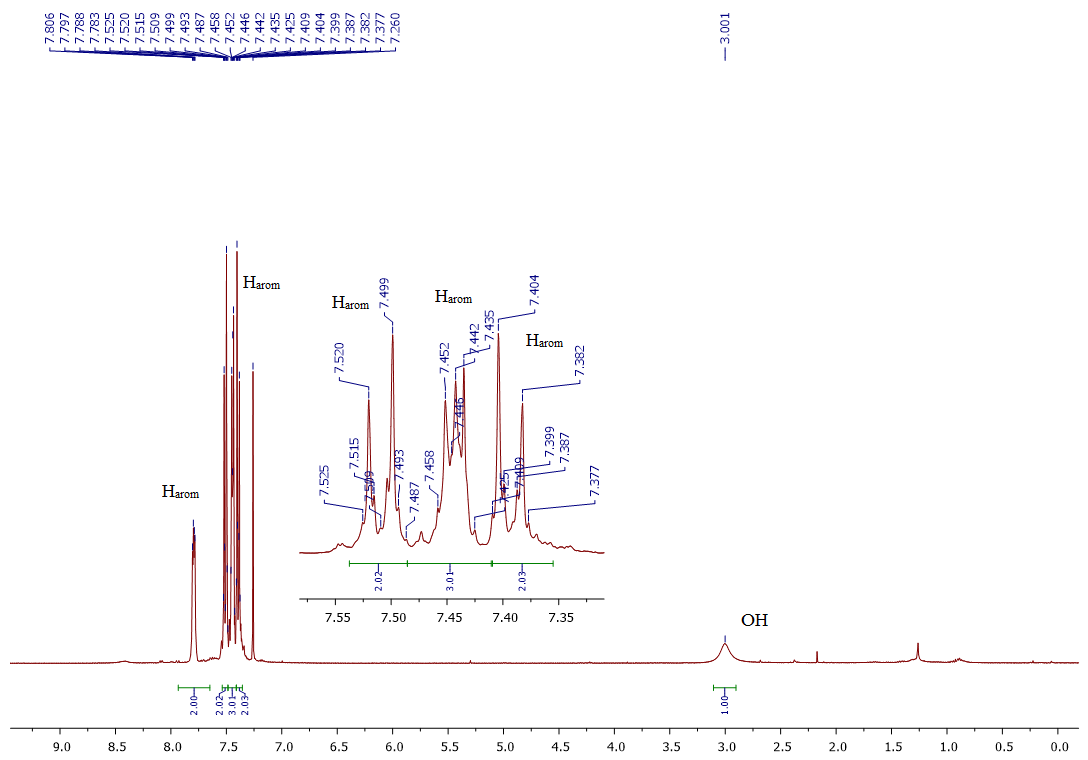


Figure S92. ^1^Н-NMR spectrum of the compound **1m** (CDCl_3_, 400 MHz).


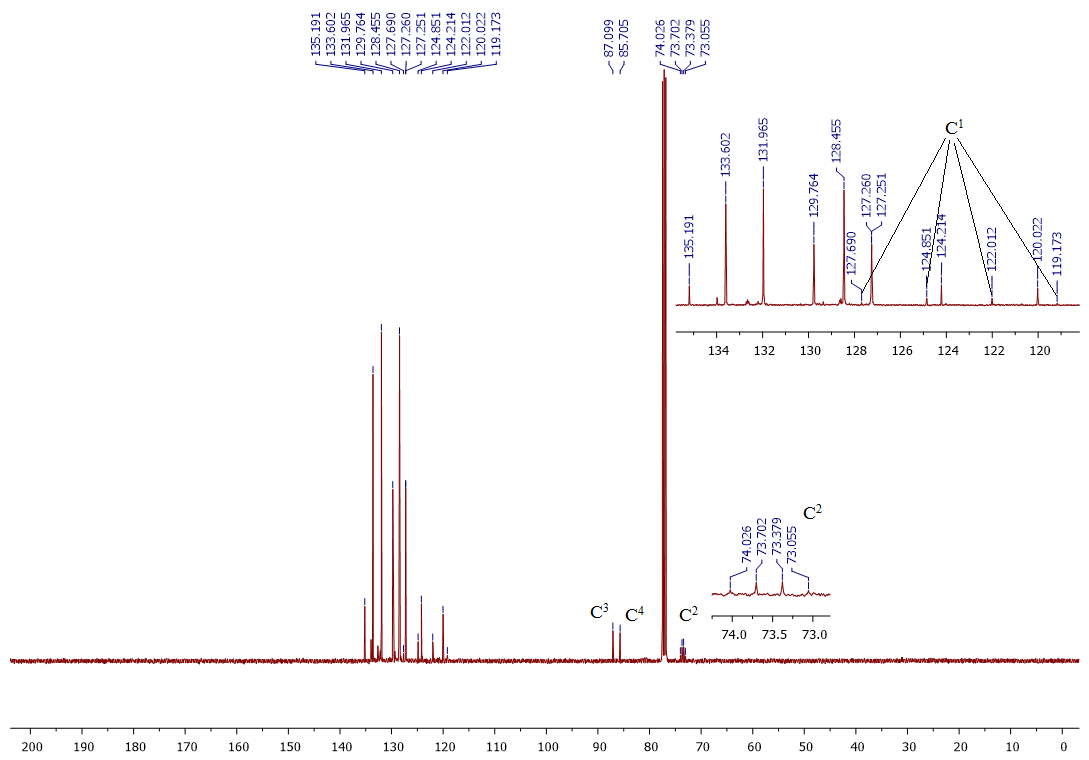


Figure S93. ^13^C-NMR spectrum of the compound **1m** (CDCl_3_, 100 MHz).


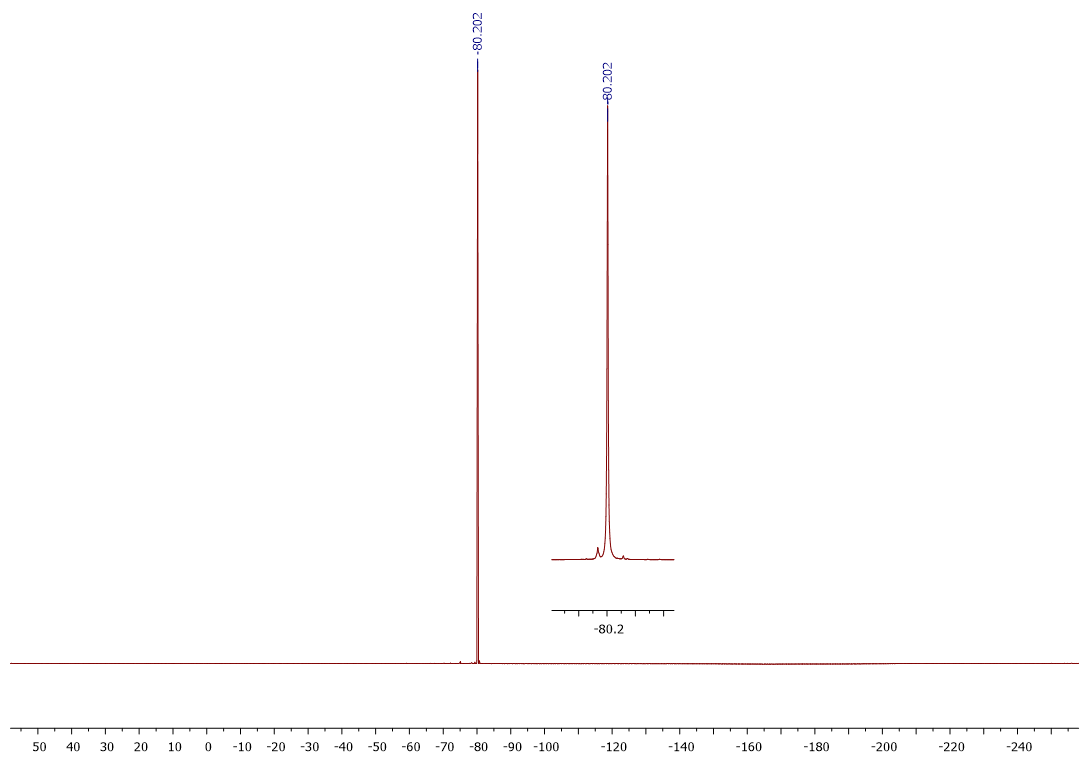


Figure S94. ^19^F-NMR spectrum of the compound **1m** (CDCl_3_, 376 MHz).


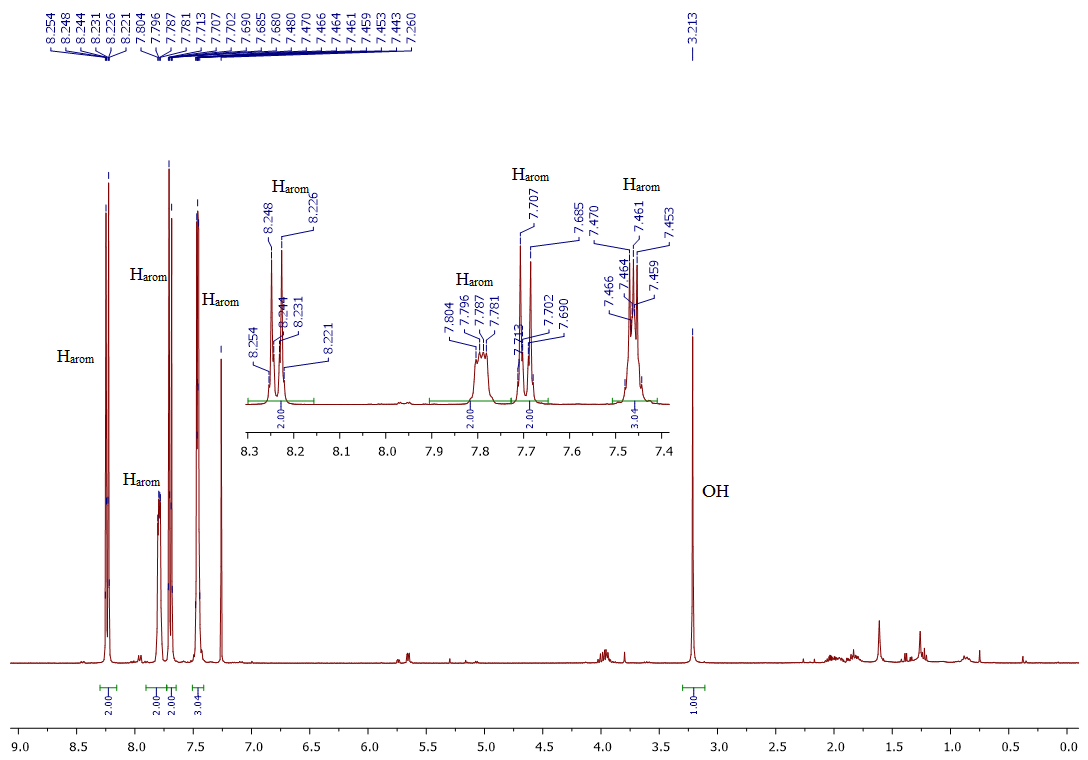


Figure S95. ^1^Н-NMR spectrum of the compound **1n** (CDCl_3_, 400 MHz).


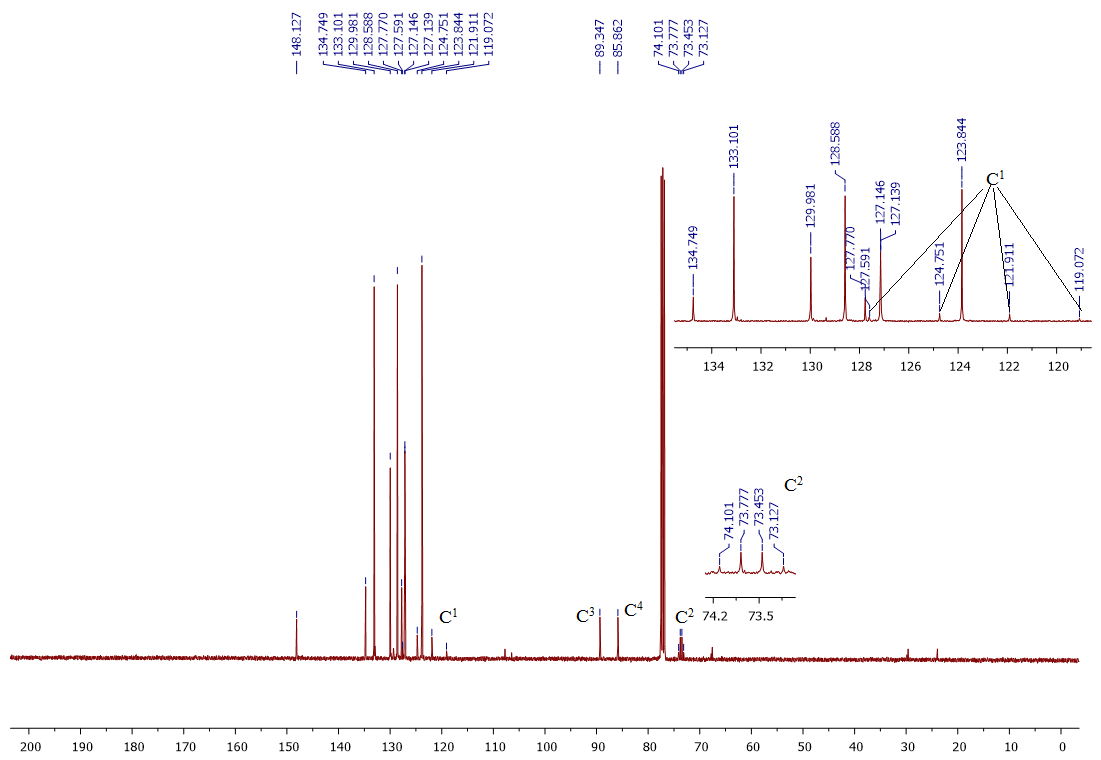


Figure S96. ^13^C-NMR spectrum of the compound **1n** (CDCl_3_, 100 MHz).


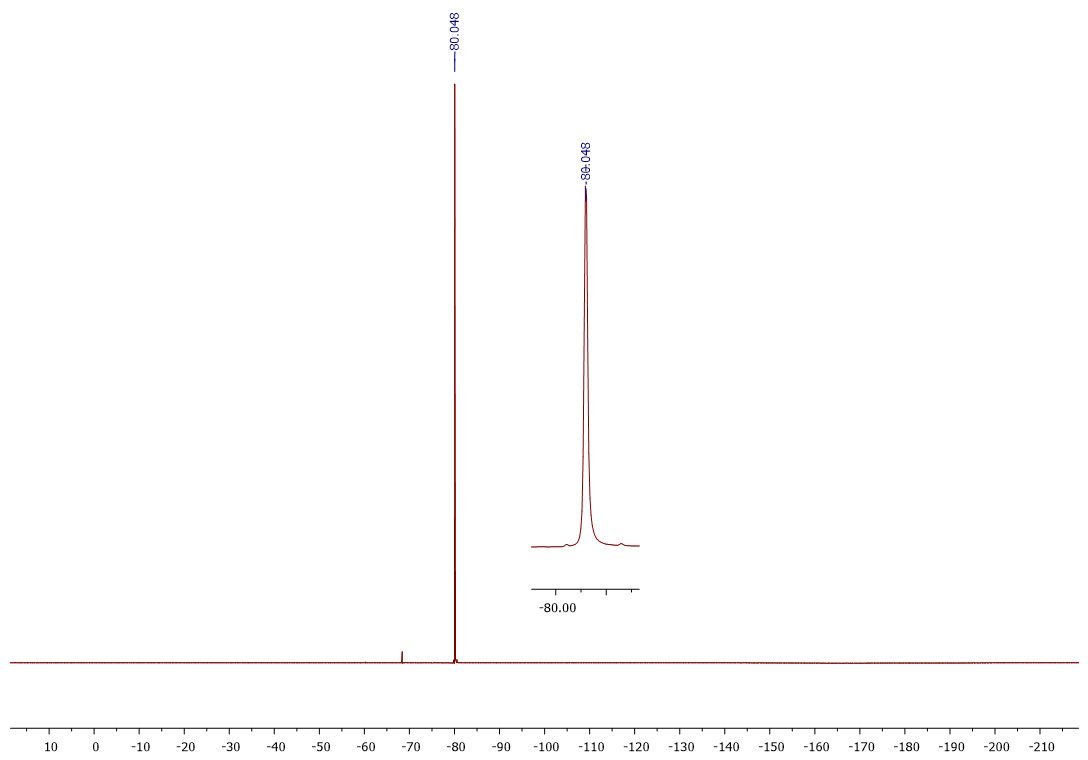


Figure S97. ^19^F-NMR spectrum of the compound **1n** (CDCl_3_, 376 MHz).


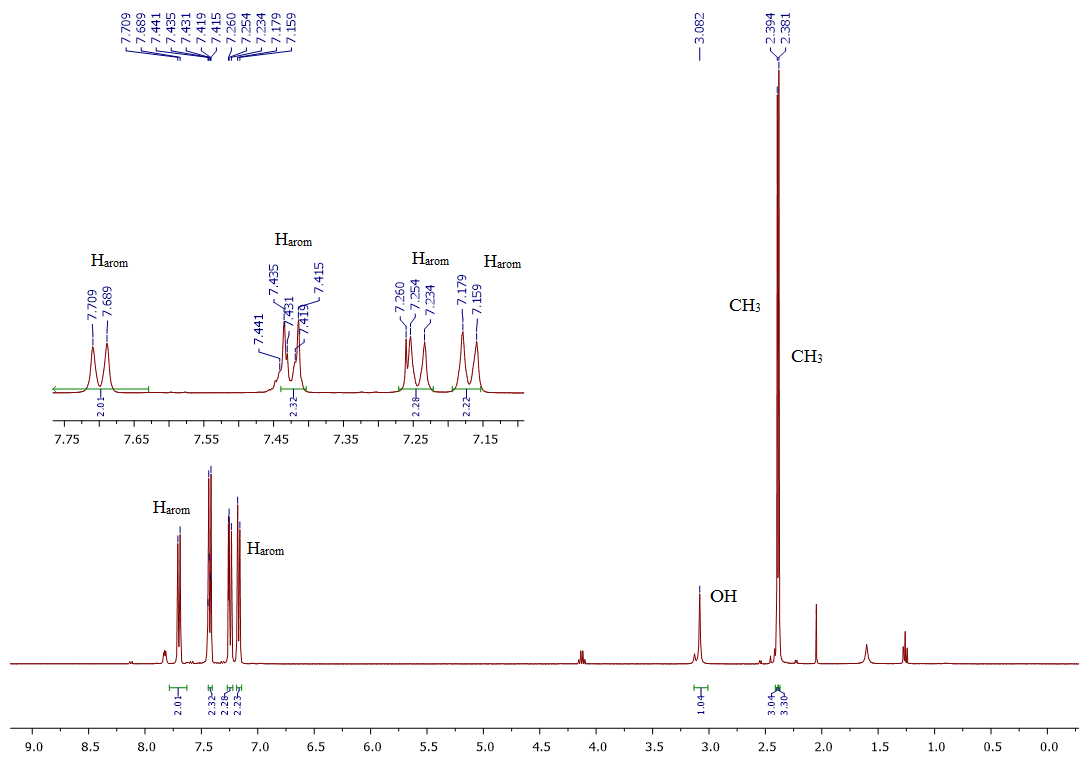


Figure S98. ^1^Н-NMR spectrum of the compound **1o** (CDCl_3_, 400 MHz).


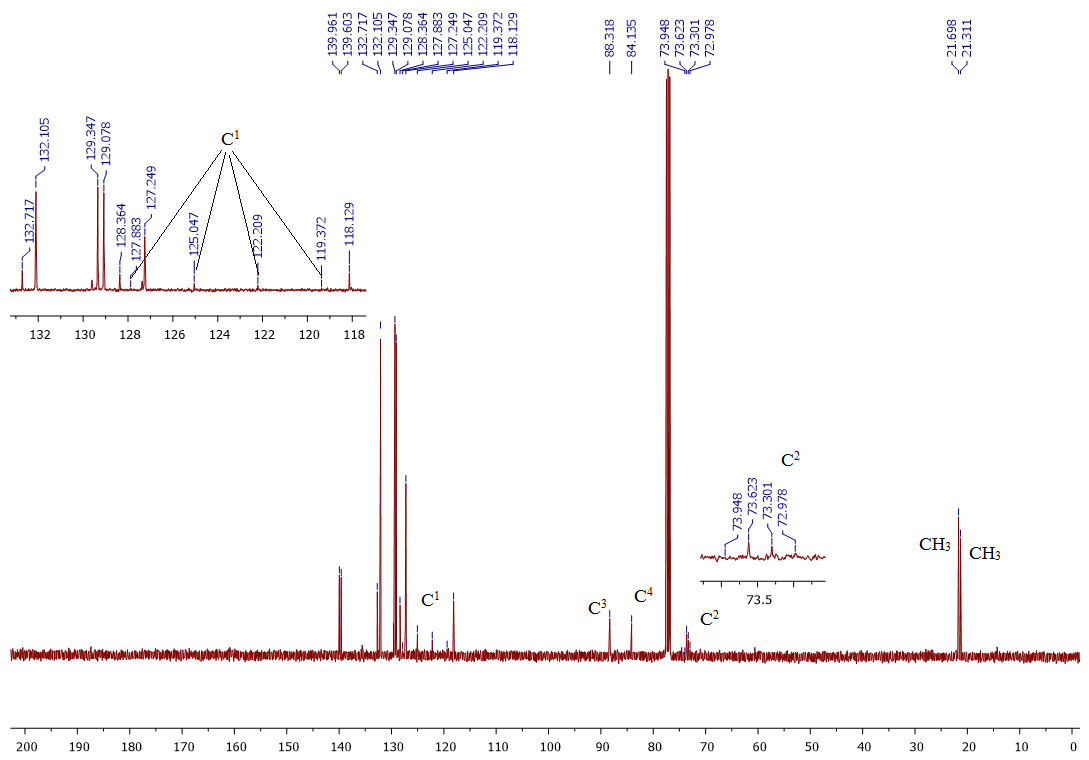


Figure S99. ^13^C-NMR spectrum of the compound **1o** (CDCl_3_, 100 MHz).


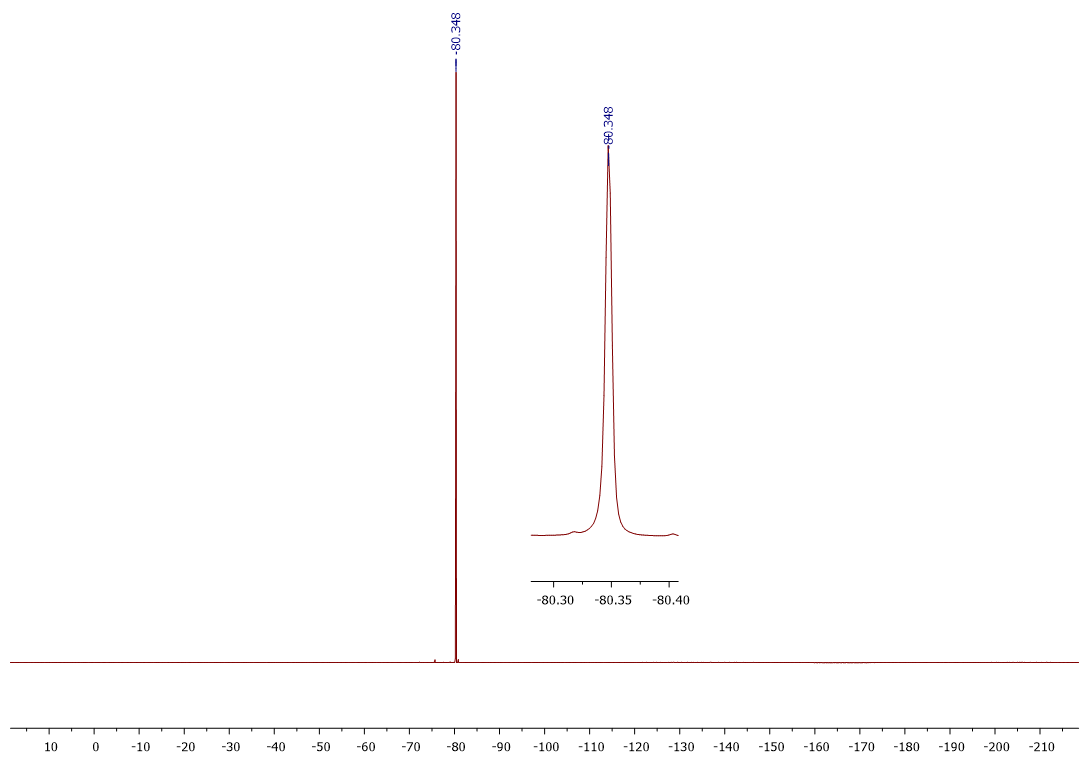


Figure S100. ^19^F-NMR spectrum of the compound **1o** (CDCl_3_, 376 MHz).

Figure S101. ^1^Н-NMR spectrum of the compound **1p** (CDCl_3_, 400 MHz).

Figure S102. ^13^C-NMR spectrum of the compound **1p** (CDCl_3_, 100 MHz).

Figure S103. ^19^F-NMR spectrum of the compound **1p** (CDCl_3_, 376 MHz).

Figure S104. ^1^Н-NMR spectrum of the compound **1q** (CDCl_3_, 400 MHz).

Figure S105. ^13^C-NMR spectrum of the compound **1q** (CDCl_3_, 100 MHz).

Figure S106. ^19^F-NMR spectrum of the compound **1q** (CDCl_3_, 376 MHz).

Figure S107. ^1^Н-NMR spectrum of the compound **1r** (CDCl_3_, 400 MHz).

Figure S108. ^13^C-NMR spectrum of the compound **1r** (CDCl_3_, 100 MHz).

Figure S109. ^19^F-NMR spectrum of the compound **1r** (CDCl_3_, 376 MHz).

Figure S110. ^1^Н-NMR spectrum of the compound **2a** (CDCl_3_, 400 MHz).

Figure S111. ^13^C-NMR spectrum of the compound **2a** (CDCl_3_, 100 MHz).

Figure S112. ^19^F-NMR spectrum of the compound **2a** (CDCl_3_, 376 MHz).

H_arom._

H_arom._

H_arom._

H_arom._

C^2^H

Figure S113. ^1^Н-NMR spectrum of the compound **4aa** (CDCl_3_, 400 MHz).

C^1^

C^1^

CF_3_

Figure S114. ^13^C-NMR spectrum of the compound **4aa** (CDCl_3_, 100 MHz).

Figure S115. DEPT spectrum of the compound **4aa** (CDCl_3_, 100 MHz).

Figure S116. ^19^F-NMR spectrum of the compound **4aa** (CDCl_3_, 376 MHz).

H_arom._

H_arom._

H_arom._

H_arom._

H_arom._

H_arom._

C^2^H

CH_3_

Figure S117. ^1^Н-NMR spectrum of the compound **4ab** (CDCl_3_, 400 MHz).

CF_3_

CH_3_

C^1^

C^1^

Figure S118. ^13^C-NMR spectrum of the compound **4ab** (CDCl_3_, 100 MHz).

CH_3_

Figure S119. DEPT spectrum of the compound **4ab** (CDCl_3_, 100 MHz).

Figure S120. ^19^F-NMR spectrum of the compound **4ab** (CDCl_3_, 376 MHz).

H_arom_

H_arom_

H_arom_

H_arom_

H_arom_

C^2^H

CH_3_

Figure S121. ^1^Н-NMR spectrum of the compound **4ac** (CDCl_3_, 400 MHz).

CF_3_

C^1^

C^1^

CH_3_

Figure S122. ^13^C-NMR spectrum of the compound **4ac** (CDCl_3_, 100 MHz).

Figure S123. ^19^F-NMR spectrum of the compound **4ac** (CDCl_3_, 376 MHz).

H_arom._

C^2^H

H_arom._

H_arom._

CH_3_

H_arom._

H_arom._

H_arom._

H_arom._

H_arom._

H_arom._

C^2^H

CH_3_

Figure S124. ^1^Н-NMR spectrum of the compounds **4ad** and **6a** (CDCl_3_, 400 MHz).

CF_3_

C^1^

C^1^

CH_3_

Figure S125. ^13^C-NMR spectrum of the compound **4ad** (CDCl_3_, 100 MHz).

CH_3_

Figure S126. DEPT spectrum of the compound **4ad** (CDCl_3_, 100 MHz).

CF_3_

CF_3_

CF_3_

CF_3_

Figure S127. ^19^F-NMR spectrum of the compounds **4ad** and **6a** (CDCl_3_, 376 MHz).

Figure S128. NOESY H-F spectrum of the compound **4ad** (CDCl_3_, 400 MHz).

H_arom._

H_arom._

H_arom._

C^2^H

H_arom._

H_arom._

Figure S129. ^1^Н-NMR spectrum of the compound **4ae** (CDCl_3_, 400 MHz).

C^1^

C^1^

CF_3_

Figure S130. ^13^C-NMR spectrum of the compound **4ae** (CDCl_3_, 100 MHz).

Figure S131. DEPT spectrum of the compound **4ae** (CDCl_3_, 100 MHz).

Figure S132. ^19^F-NMR spectrum of the compound **4ae** (CDCl_3_, 376 MHz).

H_arom._

H_arom._

H_arom._

H_arom._

C^2^H

Figure S133. ^1^Н-NMR spectrum of the compound **4af** (CDCl_3_, 400 MHz).

C^1^

C^1^

CF_3_

Figure S134. ^13^C-NMR spectrum of the compound **4af** (CDCl_3_, 100 MHz).

Figure S135. ^19^F-NMR spectrum of the compound **4af** (CDCl_3_, 376 MHz).

H_arom._

H_arom._

H_arom._

H_arom._

H_arom._

H_arom._

C^2^H

Figure S136. ^1^Н-NMR spectrum of the compound **4ag** (CDCl_3_, 400 MHz).

C^1^

C^1^

CF_3_

Figure S137. ^13^C-NMR spectrum of the compound **4ag** (CDCl_3_, 100 MHz).

Figure S138. DEPT spectrum of the compound **4ag** (CDCl_3_, 100 MHz).

Figure S139. ^19^F-NMR spectrum of the compound **4ag** (CDCl_3_, 376 MHz).

C^2^H

H_arom._

H_arom._

H_arom._

H_arom._

H_arom._

H_arom._

Figure S140. ^1^Н-NMR spectrum of the compound **4ah** (CDCl_3_, 400 MHz).

C^1^

C^1^

CF_3_

Figure S141. ^13^C-NMR spectrum of the compound **4ah** (CDCl_3_, 100 MHz).

Figure S142. DEPT spectrum of the compound **4ah** (CDCl_3_, 100 MHz).

Figure S143. ^19^F-NMR spectrum of the compound **4ah** (CDCl_3_, 376 MHz).

H_arom._

H_arom._

H_arom._

H_arom._

H_arom._

H_arom._

H_arom._

H_arom._

H_arom._

H_arom._

H_arom._

H_arom._

H_arom._

CH_3_

CH_3_

C^2^H

C^2^H

Figure S144. ^1^Н-NMR spectrum of the compounds **4ai** and **5aa** (CDCl_3_, 400 MHz).

CF_3_

CF_3_

C^1^

C^1^

C^1^

C^1^

CH_3_

CH_3_

CH_3_

Figure S145. ^13^C-NMR spectrum of the compounds **4ai** and **5aa** (CDCl_3_, 100 MHz).

CH_3_

CH_3_

CH_3_

CH_3_

Figure S146. DEPT spectrum of the compounds **4ai** and **5aa** (CDCl_3_, 100 MHz).

CF_3_

CF_3_

CF_3_

CF_3_

Figure S147. ^19^F-NMR spectrum of the compounds **4ai** and **5aa** (CDCl_3_, 376 MHz).

Figure S148. COSY H-H spectrum of the compounds**4ai** and **5aa** (CDCl_3_, 400 MHz).

Figure S149. NOESY H-H spectrum of the compounds**4ai**, **5aa** (CDCl_3_, 400 MHz).

Figure S150. NOESY H-F spectrum of the compounds **4ai**, **5aa** (CDCl_3_, 400 MHz).

H_arom._

H_arom._

H_arom._

H_arom._

C^7^H

H_arom._

H_arom._

C^5^H

C^5^H

H_arom._

H_arom._

C^2^H

C^2^H

CH_3_

CH_3_

CH_3_

CH_3_

Figure S151. ^1^Н-NMR spectrum of the compounds **4aj** and **5ab** (CDCl_3_, 400 MHz).

CF_3_

CF_3_

C^1^

C^1^

C^1^

CH_3_

CH_3_

CH_3_

CH_3_

CH_3_

C^1^

CH_3_

Figure S152. ^13^C-NMR spectrum of the compounds **4aj** and **5ab** (CDCl_3_, 100 MHz).

CH_3_

CH_3_

CH_3_

CH_3_

CH_3_

CH_3_

Figure S153. DEPT spectrum of the compounds **4aj** and **5ab** (CDCl_3_, 100 MHz).

CF_3_

CF_3_

CF_3_

CF_3_

Figure S154. ^19^F-NMR spectrum of the compounds **4aj** and **5ab** (CDCl_3_, 376 MHz).

Figure S155. NOESY H-H spectrum of the compounds **4aj**, **5ab** (CDCl_3_, 400 MHz).

Figure S156. NOESY H-F spectrum of the compounds **4aj**, **5ab** (CDCl_3_, 400 MHz).

C^2^H

H_arom._

H_arom._

H_arom._

H_arom._

Figure S157. ^1^Н-NMR spectrum of the compound **4ak** (CDCl_3_, 400 MHz).

C^1^

CF_3_

C^1^

Figure S158. ^13^C-NMR spectrum of the compound **4ak** (CDCl_3_, 100 MHz).

Figure S159. DEPT spectrum of the compound **4ak** (CDCl_3_, 100 MHz).

Figure S160. ^19^F-NMR spectrum of the compound **4ak** (CDCl_3_, 376 MHz).

H_arom._

H_arom._

H_arom._

H_arom._

C^2^H

H_arom._

Figure S161. ^1^Н-NMR spectrum of the compound **4al** (CDCl_3_, 400 MHz).

CF_3_

C^1^

C^1^

Figure S162. ^13^C-NMR spectrum of the compound **4al** (CDCl_3_, 100 MHz).

Figure S163. DEPT spectrum of the compound **4al** (CDCl_3_, 100 MHz).

Figure S164. ^19^F-NMR spectrum of the compound **4al** (CDCl_3_, 376 MHz).

H_arom._

H_arom._

H_arom._

H_arom._

H_arom._

H_arom._

C^2^H

Figure S165. ^1^Н-NMR spectrum of the compound **4am** (CDCl_3_, 400 MHz).

C^1^

C^1^

CF_3_

Figure S166. ^13^C-NMR spectrum of the compound **4am** (CDCl_3_, 100 MHz).

Figure S167. DEPT spectrum of the compound **4am** (CDCl_3_, 100 MHz).

Figure S168. ^19^F-NMR spectrum of the compound **4am** (CDCl_3_, 376 MHz).

H_arom._

C^7^H

H_arom._

H_arom._

H_arom._

C^7^H

H_arom._

H_arom._

C^2^H

Figure S169. ^1^Н-NMR spectrum of the compound **4an** (CDCl_3_, 400 MHz).

CF_3_

C^1^

C^1^

Figure S170. ^13^C-NMR spectrum of the compound **4an** (CDCl_3_, 100 MHz).

Figure S171. DEPT spectrum of the compound **4an** (CDCl_3_, 100 MHz).

Figure S172. ^19^F-NMR spectrum of the compound **4an** (CDCl_3_, 376 MHz).

Figure S173. NOESY H-H spectrum of the compound **4an** (CDCl_3_, 400 MHz).

Figure S174. NOESY H-F spectrum of the compound **4an** (CDCl_3_, 400 MHz).

H_arom._

C^2^H

H_arom._

H_arom._

H_arom._

Figure S175. ^1^Н-NMR spectrum of the compound **4ao** (CDCl_3_, 400 MHz).

CF_3_

C^1^

C^1^

Figure S176. ^13^C-NMR spectrum of the compound **4ao** (CDCl_3_, 100 MHz).

Figure S177. DEPT spectrum of the compound **4ao** (CDCl_3_, 100 MHz).

Figure S178. ^19^F-NMR spectrum of the compound **4ao** (CDCl_3_, 376 MHz).

C^6^H

C^5^H

H_arom._

C^6^H

C^5^H

H_arom._

C^2^H

CH_3_

CH_3_

Figure S179. ^1^Н-NMR spectrum of the compound **4ba** (CDCl_3_, 400 MHz).

CF_3_

C^1^

CH_3_

C^1^

CH_3_

CH_3_

Figure S180. ^13^C-NMR spectrum of the compound **4ba** (CDCl_3_, 100 MHz).

Figure S181. ^19^F-NMR spectrum of the compound **4ba** (CDCl_3_, 376 MHz).

Figure S182. NOESY H-H spectrum of the compound **4ba** (CDCl_3_, 400 MHz).

Figure S183. NOESY H-F spectrum of the compound **4ba** (CDCl_3_, 400 MHz).

H_arom._

H_arom._

H_arom._

C^5^H

C^6^H

CH_3_

C^6^H

C^5^H

C^2^H

CH_3_

Figure S184. ^1^Н-NMR spectrum of the compound **4bb** (CDCl_3_, 400 MHz).

C^1^

C^1^

CH_3_

CH_3_

CH_3_

CF_3_

Figure S185. ^13^C-NMR spectrum of the compound **4bb** (CDCl_3_, 100 MHz).

CH_3_

CH_3_

CH_3_

Figure S186. DEPT spectrum of the compound **4bb** (CDCl_3_, 100 MHz).

Figure S187. ^19^F-NMR spectrum of the compound **4bb** (CDCl_3_, 376 MHz).

H_arom._

C^5^H

H_arom._

C^6^H

H_arom._

C^6^H

C^5^H

C^2^H

CH_3_

CH_3_

CH_3_

Figure S188. ^1^Н-NMR spectrum of the compound **4bc** (CDCl_3_, 400 MHz).

CF_3_

C^1^

C^1^

CH_3_

CH_3_

CH_3_

Figure S189. ^13^C-NMR spectrum of the compound **4bc** (CDCl_3_, 100 MHz).

CH_3_

CH_3_

CH_3_

Figure S190. DEPT spectrum of the compound **4bc** (CDCl_3_, 100 MHz).

Figure S191. ^19^F-NMR spectrum of the compound **4bc** (CDCl_3_, 376 MHz).

Figure S192. NOESY H-H spectrum of the compound **4bc** (CDCl_3_, 400 MHz).

Figure S193. NOESY H-F spectrum of the compound **4bc** (CDCl_3_, 400 MHz).

C^6^H

H_arom._

H_arom._

C^6^H

C^2^H

CH_3_

H_arom._

CH_3_

Figure S194. ^1^Н-NMR spectrum of the compound **4bd** (CDCl_3_, 400 MHz).

CF_3_

CH_3_

CH_3_

C^1^

C^1^

CH_3_

CH_3_

Figure S195. ^13^C-NMR spectrum of the compound **4bd** (CDCl_3_, 100 MHz).

CH_3_

CH_3_

CH_3_

CH_3_

Figure S196. DEPT spectrum of the compound **4bd** (CDCl_3_, 100 MHz).

Figure S197. ^19^F-NMR spectrum of the compound **4bd** (CDCl_3_, 376 MHz).

C^2^H

C^6^H

C^5^H

H_arom._

C^6^H

C^5^H

H_arom._

CH_3_

CH_3_

Figure S198. ^1^Н-NMR spectrum of the compound **4be** (CDCl_3_, 400 MHz).

CF_3_

CH_3_

CH_3_

C^1^

C^1^

CH_3_

Figure S199. ^13^C-NMR spectrum of the compound **4be** (CDCl_3_, 100 MHz).

CH_3_

CH_3_

CH_3_

Figure S200. DEPT spectrum of the compound **4be** (CDCl_3_, 100 MHz).

Figure S201. ^19^F-NMR spectrum of the compound **4be** (CDCl_3_, 376 MHz).

Figure S202. NOESY H-H spectrum of the compound **4be** (CDCl_3_, 400 MHz).

Figure S203. NOESY H-F spectrum of the compound **4be** (CDCl_3_, 400 MHz).

C^6^H

C^5^H

C^2^H

H_arom._

C^6^H

CH_3_

CH_3_

H_arom._

C^5^H

H_arom._

Figure S204. ^1^Н-NMR spectrum of the compound **4bf** (CDCl_3_, 400 MHz).

CH_3_

CH_3_

CH_3_

C^1^

CF_3_

C^1^

Figure S205. ^13^C-NMR spectrum of the compound **4bf** (CDCl_3_, 100 MHz).

CH_3_

CH_3_

CH_3_

Figure S206. DEPT spectrum of the compound **4bf** (CDCl_3_, 100 MHz).

Figure S207. ^19^F-NMR spectrum of the compound **4bf** (CDCl_3_, 376 MHz).

Figure S208. NOESY H-H spectrum of the compound **4bf** (CDCl_3_, 400 MHz).

Fig S209. NOESY H-F spectrum of the compound **4bf** (CDCl_3_, 400 MHz).

H_arom._

H_arom._

H_arom._

H_arom._

H_arom._

C^6^H

C^5^H

C^5^H

C^6^H

C^2^H

CH_3_

CH_3_

Figure S210. ^1^Н-NMR spectrum of the compound **4bg** (CDCl_3_, 400 MHz).

CF_3_

CH_3_

CH_3_

CH_3_

C^1^

C^1^

Figure S211. ^13^C-NMR spectrum of the compound **4bg** (CDCl_3_, 100 MHz).

CH_3_

CH_3_

Figure S212. DEPT spectrum of the compound **4bg** (CDCl_3_, 100 MHz).

Figure S213. ^19^F-NMR spectrum of the compound **4bg** (CDCl_3_, 376 MHz).

C^6^H

C^5^H

H_arom._

H_arom._

H_arom._

H_arom._

C^5^H

C^6^H

C^2^H

CH_3_

CH_3_

Figure S214. ^1^Н-NMR spectrum of the compound **4bh** (CDCl_3_, 400 MHz).

CH_3_

CH_3_

C^1^

C^1^

CH_3_

CF_3_

Figure S215. ^13^C-NMR spectrum of the compound **4bh** (CDCl_3_, 100 MHz).

CH_3_

CH_3_

CH_3_

Figure S216. DEPT spectrum of the compound **4bh** (CDCl_3_, 100 MHz).

Figure S217. ^19^F-NMR spectrum of the compound **4bh** (CDCl_3_, 376 MHz).

C^2^H

H_arom._

C^6^H

C^5^H

C^6^H

C^5^H

C^2^H

CH_3_

CH_3_

H_arom._

Figure S218. ^1^Н-NMR spectrum of the compound **4bi** (CDCl_3_, 400 MHz).

CF_3_

C^1^

C^1^

CH_3_

CH_3_

CH_3_

Figure S219. ^13^C-NMR spectrum of the compound **4bi** (CDCl_3_, 100 MHz).

CH_3_

CH_3_

CH_3_

Figure S220. DEPT spectrum of the compound **4bi** (CDCl_3_, 100 MHz).

Figure S221. ^19^F-NMR spectrum of the compound **4bi** (CDCl_3_, 376 MHz).

Figure S222. NOESY H-H spectrum of the compound **4bi** (CDCl_3_, 400 MHz).

Figure S223. NOESY H-F spectrum of the compound **4bi** (CDCl_3_, 400 MHz).

H_arom._

H_arom._

H_arom._

H_arom._

H_arom._

H_arom._

H_arom._

C^5^H

C^6^H

C^2^H

C^2^H

CH_3_

CH_3_

CH_3_

CH_3_

CH_3_

CH_3_

CH_3_

CH_3_

Figure S224. ^1^Н-NMR spectrum of the compounds **4bj** and **5ba** (CDCl_3_, 400 MHz).

CF_3_

CF_3_

C^1^

C^1^

C^1^

C^1^

CH_3_

CH_3_

CH_3_

CH_3_

CH_3_

CH_3_

CH_3_

CH_3_

CH_3_

Figure S225. ^13^C-NMR spectrum of the compounds **4bj** and **5ba** (CDCl_3_, 100 MHz).

Figure S226. DEPT spectrum of the compounds **4bj** and **5ba** (CDCl_3_, 100 MHz).

CH_3_

CH_3_

CH_3_

CH_3_

CH_3_

CH_3_

CH_3_

CH_3_

CH_3_

CF_3_

CF_3_

CF_3_

CF_3_

Figure S227. ^19^F-NMR spectrum of the compounds **4bj** and **5ba** (CDCl_3_, 376 MHz).

Figure S228. NOESY H-H spectrum of the compounds **4bj**, **5ba** (CDCl_3_, 400 MHz).

Figure S229. NOESY H-F spectrum of the compounds **4bj**, **5ba** (CDCl_3_, 400 MHz).

C^6^H

C^5^H

C^6^H

C^5^H

H_arom._

C^2^H

H_arom._

CH_3_

CH_3_

Figure S230. ^1^Н-NMR spectrum of the compound **4bk** (CDCl_3_, 400 MHz).

CF_3_

C^1^

C^1^

CH_3_

CH_3_

CH_3_

Figure S231. ^13^C-NMR spectrum of the compound **4bk** (CDCl_3_, 100 MHz).

CH_3_

CH_3_

CH_3_

Figure S232. DEPT spectrum of the compound **4bk** (CDCl_3_, 100 MHz).

Figure S233. ^19^F-NMR spectrum of the compound **4bk** (CDCl_3_, 376 MHz).

Figure S234. NOESY H-H spectrum of the compound **4bk** (CDCl_3_, 400 MHz).

Figure S235. NOESY H-F spectrum of the compound **4bk** (CDCl_3_, 400 MHz).

H_arom._

H_arom._

C^5^H

C^6^H

C^2^H

CH_3_

CH_3_

C^6^H

C^5^H

H_arom._

Figure S236. ^1^Н-NMR spectrum of the compound **4bl** (CDCl_3_, 400 MHz).

CF_3_

C^1^

C^1^

CH_3_

CH_3_

CH_3_

Figure S237. ^13^C-NMR spectrum of the compound **4bl** (CDCl_3_, 100 MHz).

CH_3_

CH_3_

CH_3_

Figure S238. DEPT spectrum of the compound **4bl** (CDCl_3_, 100 MHz).

Figure S239. ^19^F-NMR spectrum of the compound **4bl** (CDCl_3_, 376 MHz).

Figure S240. NOESY H-H spectrum of the compound **4bl** (CDCl_3_, 400 MHz).

Figure S241. NOESY H-F spectrum of the compound **4bl** (CDCl_3_, 400 MHz).

H_arom._

H_arom._

H_arom._

H_arom._

H_arom._

H_arom._

H_arom._

C^2^H

CH_3_

CH_3_

Figure S242. ^1^Н-NMR spectrum of the compound **4bm** (CDCl_3_, 400 MHz).

CF_3_

CH_3_

CH_3_

CH_3_

C^1^

C^1^

Figure S243. ^13^C-NMR spectrum of the compound **4bm** (CDCl_3_, 100 MHz).

CH_3_

CH_3_

CH_3_

Figure S244. DEPT spectrum of the compound **4bm** (CDCl_3_, 100 MHz).

Figure S245. ^19^F-NMR spectrum of the compound **4bm** (CDCl_3_, 376 MHz).

CH_3_

CH_3_

C^6^H

C^5^H

H_arom._

H_arom._

H_arom._

C^6^H

C^5^H

C^2^H

Figure S246. ^1^Н-NMR spectrum of the compound **4bn** (CDCl_3_, 400 MHz).

CH_3_

CH_3_

C^1^

CH_3_

C^1^

CF_3_

Figure S247. ^13^C-NMR spectrum of the compound **4bn** (CDCl_3_, 100 MHz).

Figure S248. ^19^F-NMR spectrum of the compound **4bn** (CDCl_3_, 376 MHz).

Figure S249. NOESY H-H spectrum of the compound **4bn** (CDCl_3_, 400 MHz).

Figure S250. NOESY H-F spectrum of the compound **4bn** (CDCl_3_, 400 MHz).

H_arom._

H_arom._

H_arom._

H_arom._

C^6^H

H_arom._

H_arom._

C^6^H

H_arom._

C^2^H

C^2^H

CH_3_

CH_3_

CH_3_

CH_3_

CH_3_

CH_3_

CH_3_

CH_3_

CH_3_

CH_3_

CH_3_

Figure S251. ^1^Н-NMR spectrum of the compounds **4bo** and **5bc** (CDCl_3_, 400 MHz).

CF_3_

C^1^

C^1^

CH_3_

CH_3_

CH_3_

CF_3_

CH_3_

CH_3_

CH_3_

CH_3_

CH_3_

C^1^

C^1^

CH_3_

Figure S252. ^13^C-NMR spectrum of the compounds **4bo** and **5bc** (CDCl_3_, 100 MHz).

CH_3_

CH_3_

CH_3_

CH_3_

CH_3_

CH_3_

CH_3_

CH_3_

CH_3_

CH_3_

Figure S253. DEPT spectrum of the compounds **4bo** and **5bc** (CDCl_3_, 100 MHz).

CF_3_

CF_3_

CF_3_

CF_3_

Figure S254. ^19^F-NMR spectrum of the compounds **4bo** and **5bc** (CDCl_3_, 376 MHz).

Figure S255. NOESY H-H spectrum of the compounds **4bo**, **5bc** (CDCl_3_, 400 MHz).

Figure S256. NOESY H-F spectrum of the compounds **4bo**, **5bc** (CDCl_3_, 400 MHz).

C^6^H

C^5^H

H_arom._

H_arom._

H_arom._

H_arom._

C^6^H

C^5^H

C^2^H

CH_3_

CH_3_

Figure S257. ^1^Н-NMR spectrum of the compound **4bp** (CDCl_3_, 400 MHz).

C^1^

CH_3_

CH_3_

CH_3_

C^1^

CF_3_

Figure S258. ^13^C-NMR spectrum of the compound **4bp** (CDCl_3_, 100 MHz).

CH_3_

CH_3_

CH_3_

Figure S259. DEPT spectrum of the compound **4bp** (CDCl_3_, 100 MHz).

Figure S260. ^19^F-NMR spectrum of the compound **4bp** (CDCl_3_, 376 MHz).

Figure S261. NOESY H-H spectrum of the compound **4bp** (CDCl_3_, 400 MHz).

Figure S262. NOESY H-F spectrum of the compound **4bp** (CDCl_3_, 400 MHz).

H_arom._

H_arom._

H_arom._

C^5^H

C^6^H

C^2^H

CH_3_

CH_3_

C^6^H

C^5^H

Figure S263. ^1^Н-NMR spectrum of the compound **4bq** (CDCl_3_, 400 MHz).

C^1^

CH_3_

CH_3_

CH_3_

CF_3_

C^1^

Figure S264. ^13^C-NMR spectrum of the compound **4bq** (CDCl_3_, 100 MHz).

CH_3_

CH_3_

CH_3_

Figure S265. DEPT spectrum of the compound **4bq** (CDCl_3_, 100 MHz).

Figure S266. ^19^F-NMR spectrum of the compound **4bq** (CDCl_3_, 376 MHz).

Figure S267. NOESY H-H spectrum of the compound **4bq** (CDCl_3_, 400 MHz).

Figure S268. NOESY H-F spectrum of the compound **4bq** (CDCl_3_, 400 MHz).

H_arom._

H_arom._

H_arom._

H_arom._

H_arom._

H_arom._

H_arom._

H_arom._

H_arom._

C^2^H

C^2^H

C^5^H

CH_3_

CH_3_

CH_3_

CH_3_

Figure S269. ^1^Н-NMR spectrum of the compounds **4ca** and **5ca** (CDCl_3_, 400 MHz).

CF_3_

CF_3_

C^1^

C^1^

CH_3_

CH_3_

CH_3_

CH_3_

CH_3_

CH_3_

CH_3_

C^1^

C^1^

Figure S270. ^13^C-NMR spectrum of the compounds **4ca** and **5ca** (CDCl_3_, 100 MHz).

CH_3_

CH_3_

CH_3_

CH_3_

CH_3_

CH_3_

CH_3_

CH_3_

Figure S271. DEPT spectrum of the compounds **4ca** and **5ca** (CDCl_3_, 100 MHz).

CF_3_

CF_3_

Figure S272. ^19^F-NMR spectrum of the compounds **4ca** and **5ca** (CDCl_3_, 376 MHz).

Figure S273. NOESY H-H spectrum of the compounds **4ca**, **5ca** (CDCl_3_, 400 MHz).

Figure S274. NOESY H-F spectrum of the compounds **4ca**, **5ca** (CDCl_3_, 400 MHz).

H_arom._

H_arom._

H_arom._

H_arom._

H_arom._

C^7^H

H_arom._

H_arom._

H_arom._

C^5^H

H_arom._

H_arom._

C^5^H

C^2^H

C^2^H

CH_3_

CH_3_

CH_3_

CH_3_

Figure S275. ^1^Н-NMR spectrum of the compounds **4cb** and **5cb** (CDCl_3_, 400 MHz).

CF_3_

CF_3_

C^1^

C^1^

C^1^

C^1^

CH_3_

CH_3_

CH_3_

CH_3_

CH_3_

CH_3_

Figure S276. ^13^C-NMR spectrum of the compounds **4cb** and **5cb** (CDCl_3_, 100 MHz).

CH_3_

CH_3_

CH_3_

CH_3_

CH_3_

CH_3_

CH_3_

Figure S277. DEPT spectrum of the compounds **4cb** and **5cb** (CDCl_3_, 100 MHz).

CH_3_

CH_3_

CH_3_

CH_3_

Figure S278. ^19^F-NMR spectrum of the compounds **4cb** and **5cb** (CDCl_3_, 376 MHz).

Figure S279. COSY H-H spectrum of the compounds **4cb** and **5cb** (CDCl_3_, 400 MHz).

Figure S280. NOESY H-H spectrum of the compounds **4cb**, **5cb** (CDCl_3_, 400 MHz).

Figure S281. NOESY H-F spectrum of the compounds **4cb**, **5cb** (CDCl_3_, 400 MHz).

H_arom._

H_arom._

H_arom._

H_arom._

H_arom._

H_arom._

H_arom._

C^5^H

C^5^H

C^2^H

C^2^H

CH_3_

CH_3_

CH_3_

CH_3_

H_arom._

H_arom._

Figure S282. ^1^Н-NMR spectrum of the compounds **4cc** and **5cc** (CDCl_3_, 400 MHz).

CF_3_

CF_3_

CH_3_

CH_3_

CH_3_

CH_3_

CH_3_

CH_3_

CH_3_

C^1^

C^1^

C^1^

C^1^

Figure S283. ^13^C-NMR spectrum of the compounds **4cc** and **5cc** (CDCl_3_, 100 MHz).

CH_3_

CH_3_

CH_3_

CH_3_

CH_3_

CH_3_

CH_3_

CH_3_

Figure S284. DEPT spectrum of the compounds **4cc** and **5cc** (CDCl_3_, 100 MHz).

CF_3_

CF_3_

Figure S285. ^19^F-NMR spectrum of the compounds **4cc** and **5cc** (CDCl_3_, 376 MHz).

Figure S286. NOESY H-H spectrum of the compounds **4cc**, **5cc** (CDCl_3_, 400 MHz).

Figure S287. NOESY H-F spectrum of the compounds **4cc**, **5cc** (CDCl_3_, 400 MHz).

H_arom._

H_arom._

H_arom._

H_arom._

H_arom._

H_arom._

H_arom._

C^7^H

H_arom._

H_arom._

C^5^H

H_arom._

C^2^H

C^5^H

C^2^H

CH_3_

CH_3_

CH_3_

CH_3_

H_arom._

Figure S288. ^1^Н-NMR spectrum of the compounds **4cd** and **5cd** (CDCl_3_, 400 MHz).

CF_3_

CF_3_

C^1^

C^1^

C^1^

CH_3_

CH_3_

CH_3_

CH_3_

CH_3_

Figure S289. ^13^C-NMR spectrum of the compounds **4cd** and **5cd** (CDCl_3_, 100 MHz).

CF_3_

CF_3_

Figure S290. ^19^F-NMR spectrum of the compounds **4cd** and **5cd** (CDCl_3_, 376 MHz).

Figure S291. NOESY H-H spectrum of the compounds **4cd**, **5cd** (CDCl_3_, 400 MHz).

Figure S292. NOESY H-F spectrum of the compounds **4cd**, **5cd** (CDCl_3_, 400 MHz).

H_arom._

H_arom._

H_arom._

H_arom._

H_arom._

H_arom._

C^7^H

C^5^H

H_arom._

H_arom._

C^5^H

C^2^H

C^2^H

CH_3_

CH_3_

CH_3_

CH_3_

Figure S293. ^1^Н-NMR spectrum of the compounds **4ce** and **5ce** (CDCl_3_, 400 MHz).

CF_3_

CF_3_

C^1^

C^1^

C^1^

C^1^

CH_3_

CH_3_

CH_3_

CH_3_

Figure S294. ^13^C-NMR spectrum of the compounds **4ce** and **5ce** (CDCl_3_, 100 MHz).

CH_3_

CH_3_

CH_3_

CH_3_

Figure S295. DEPT spectrum of the compounds **4ce** and **5ce** (CDCl_3_, 100 MHz).

CF_3_

CF_3_

Figure S296. ^19^F-NMR spectrum of the compounds **4ce** and **5ce** (CDCl_3_, 376 MHz).

C^7^H

CH_3_

C^7^H

C^5^H

C^5^H

H_arom._

H_arom._

H_arom._

H_arom._

H_arom._

H_arom._

C^2^H

CH_3_

Figure S297. ^1^Н-NMR spectrum of the compound **4cf** (CDCl_3_, 400 MHz).

CF_3_

C^1^

C^1^

CH_3_

CH_3_

Figure S298. ^13^C-NMR spectrum of the compound **4cf** (CDCl_3_, 100 MHz).

CH_3_

CH_3_

Figure S299. DEPT spectrum of the compound **4cf** (CDCl_3_, 100 MHz).

Figure S300. ^19^F-NMR spectrum of the compound **4cf** (CDCl_3_, 376 MHz).

H_arom._

H_arom._

C^2^H

H_arom._

H_arom._

H_arom._

H_arom._

CH_3_

CH_3_

H_arom._

Figure S301. ^1^Н-NMR spectrum of the compound **4cg** (CDCl_3_, 400 MHz).

CH_3_

CH_3_

C^1^

C^1^

CF_3_

Figure S302. ^13^C-NMR spectrum of the compound **4cg** (CDCl_3_, 100 MHz).

CH_3_

CH_3_

Figure S303. DEPT spectrum of the compound **4cg** (CDCl_3_, 100 MHz).

Figure S304. ^19^F-NMR spectrum of the compound **4cg** (CDCl_3_, 376 MHz).

H_arom._

H_arom._

H_arom._

H_arom._

H_arom._

H_arom._

H_arom._

H_arom._

H_arom._

H_arom._

H_arom._

H_arom._

C^5^H

C^5^H

CH_3_

CH_3_

CH_3_

CH_3_

C^2^H

C^2^H

Figure S305. ^1^Н-NMR spectrum of the compounds **4ch** and **5cf** (CDCl_3_, 400 MHz).

CF_3_

CF_3_

CH_3_

CH_3_

CH_3_

CH_3_

CH_3_

C^1^

C^1^

C^1^

C^1^

CH_3_

CH_3_

Figure S306. ^13^C-NMR spectrum of the compounds **4ch** and **5cf** (CDCl_3_, 100 MHz).

CH_3_

CH_3_

CH_3_

CH_3_

Figure S307. DEPT spectrum of the compounds **4ch** and **5cf** (CDCl_3_, 100 MHz).

CH_3_

CH_3_

CH_3_

CH_3_

Figure S308. ^19^F-NMR spectrum of the compounds **4ch** and **5cf** (CDCl_3_, 376 MHz).

Figure S309. NOESY H-H spectrum of the compounds **4ch**, **5cf** (CDCl_3_, 400 MHz).

Figure S310. NOESY H-F spectrum of the compounds **4ch**, **5cf** (CDCl_3_, 400 MHz).

H_arom._

H_arom._

C^7^H

H_arom._

H_arom._

C^2^H

CH_3_

CH_3_

Figure S311. ^1^Н-NMR spectrum of the compound **4ci** (CDCl_3_, 400 MHz).

CF_3_

CH_3_

CH_3_

C^1^

C^1^

Figure S312. ^13^C-NMR spectrum of the compound **4ci** (CDCl_3_, 100 MHz).

CH_3_

CH_3_

Figure S313. DEPT spectrum of the compound **4ci** (CDCl_3_, 100 MHz).

Figure S314. ^19^F-NMR spectrum of the compound **4ci** (CDCl_3_, 376 MHz).

Figure S315. NOESY H-H spectrum of the compound **4ci** (CDCl_3_, 400 MHz).

Figure S316. NOESY H-F spectrum of the compound **4ci** (CDCl_3_, 400 MHz).

H_arom._

H_arom._

H_arom._

H_arom._

C^7^H

C^5^H

H_arom._

C^2^H

CH_3_

CH_3_

H_arom._

Figure S317. ^1^Н-NMR spectrum of the compound **4cj** (CDCl_3_, 400 MHz).

CF_3_

C^1^

C^1^

CH_3_

CH_3_

Figure S318. ^13^C-NMR spectrum of the compound **4cj** (CDCl_3_, 100 MHz).

CH_3_

CH_3_

Figure S319. DEPT spectrum of the compound **4cj** (CDCl_3_, 100 MHz).

Figure S320. ^19^F-NMR spectrum of the compound **4cj** (CDCl_3_, 376 MHz).

Figure S321. NOESY H-H spectrum of the compound **4cj** (CDCl_3_, 400 MHz).

Figure S322. NOESY H-F spectrum of the compound **4cj** (CDCl_3_, 400 MHz).

H_arom._

H_arom._

H_arom._

H_arom._

C^7^H

C^5^H

C^2^H

CH_3_

CH_3_

Figure S323. ^1^Н-NMR spectrum of the compound **4ck** (CDCl_3_, 400 MHz).

CF_3_

C^1^

C^1^

CH_3_

CH_3_

Figure S324. ^13^C-NMR spectrum of the compound **4ck** (CDCl_3_, 100 MHz).

CH_3_

CH_3_

Figure S325. DEPT spectrum of the compound **4ck** (CDCl_3_, 100 MHz).

Figure S326. ^19^F-NMR spectrum of the compound **4ck** (CDCl_3_, 376 MHz)

H_arom._

H_arom._

H_arom._

H_arom._

H_arom._

C^2^H

CH_3_

CH_3_

Figure S327. ^1^Н-NMR spectrum of the compound **4cl** (CDCl_3_, 400 MHz).

CF_3_

CH_3_

CH_3_

C^1^

C^1^

Figure S328. ^13^C-NMR spectrum of the compound **4cl** (CDCl_3_, 100 MHz).

CH_3_

CH_3_

Figure S329. DEPT spectrum of the compound **4cl** (CDCl_3_, 100 MHz).

Figure S330. ^19^F-NMR spectrum of the compound **4cl** (CDCl_3_, 376 MHz).

H_arom._

H_arom._

C^2^H

H_arom._

H_arom._

H_arom._

H_arom._

CH_3_

CH_3_

Figure S331. ^1^Н-NMR spectrum of the compound **4cm** (CDCl_3_, 400 MHz).

CF_3_

CH_3_

CH_3_

C^1^

C^1^

Figure S332. ^13^C-NMR spectrum of the compound **4cm** (CDCl_3_, 100 MHz).

Figure S333. ^19^F-NMR spectrum of the compound **4cm** (CDCl_3_, 376 MHz).

H_arom_

H_arom_

H_arom_

H_arom_

H_arom_

C^2^H

H_arom_

CH_3_

CH_3_

CH_3_

CH_3_

CH_3_

C^2^H

H_arom_

H_arom_

Figure S334. ^1^Н-NMR spectrum of the compounds **4da** and **4ea** (CDCl_3_, 400 MHz).

C^1^

CH_3_

CH_3_

CH_3_

CF_3_

C^1^

Figure S335. ^13^C-NMR spectrum of the compound **4da** (CDCl_3_, 100 MHz).

CH_3_

CH_3_

CH_3_

Figure S336. DEPT spectrum of the compound **4da** (CDCl_3_, 100 MHz).

CF_3_

CF_3_

CF_3_

CF_3_

Figure S337. ^19^F-NMR spectrum of the compounds **4da** and **4ea** (CDCl_3_, 376 MHz).

Figure S338. NOESY H-H spectrum of the compounds **4da** and **4ea** (CDCl_3_, 400 MHz).

Figure S339. NOESY H-F spectrum of the compounds **4da**, **4ea** (CDCl_3_, 400 MHz).

CH_3_

CH_3_

CH_3_

CH_3_

CH_3_

CH_3_

CH_3_

CH_3_

CH_3_

C^6^H

C^5^H

H_arom_

H_arom_

H_arom_

C^2^H

C^6^H

C^2^H

H_arom_

C^5^H

H_arom_

H_arom_

Figure S340. ^1^Н-NMR spectrum of the compounds **4db** and **4eb** (CDCl_3_, 400 MHz).

CF_3_

C^1^

CH_3_

CH_3_

CH_3_

C^1^

Figure S341. ^13^C-NMR spectrum of the compound **4db** (CDCl_3_, 100 MHz).

CH_3_

CH_3_

CH_3_

Figure S342. DEPT spectrum of the compound **4db** (CDCl_3_, 100 MHz).

CF_3_

CF_3_

CF_3_

CF_3_

Figure S343. ^19^F-NMR spectrum of the compounds **4db** and **4eb** (CDCl_3_, 376 MHz).

Figure S344. NOESY H-H spectrum of the compounds **4db**, **4eb** (CDCl_3_, 400 MHz).

Figure S345. NOESY H-F spectrum of the compounds **4db**, **4eb** (CDCl_3_, 400 MHz).

CH_3_

CH_3_

CH_3_

CH_3_

CH_3_

CH_3_

CH_3_

CH_3_

CH_3_

H_arom_

H_arom_

H_arom_

H_arom_

H_arom_

H_arom_

H_arom_

H_arom_

C^2^H

C^2^H

H_arom_

H_arom_

H_arom_

H_arom_

Figure S346. ^1^Н-NMR spectrum of the compounds **4dc** and **4ec** (CDCl_3_, 400 MHz).

CF_3_

CH_3_

CH_3_

CH_3_

C^1^

C^1^

Figure S347. ^13^C-NMR spectrum of the compound **4dc** (CDCl_3_, 100 MHz).

CH_3_

CH_3_

CH_3_

Figure S348. DEPT spectrum of the compound **4dc** (CDCl_3_, 100 MHz).

CF_3_

CF_3_

CF_3_

CF_3_

Figure S349. ^19^F-NMR spectrum of the compounds **4dc** and **4ec** (CDCl_3_, 376 MHz).

Figure S350. NOESY H-H spectrum of the compounds **4dc**, **4ec** (CDCl_3_, 400 MHz).

Figure S351. NOESY H-F spectrum of the compounds **4dc**, **4ec** (CDCl_3_, 400 MHz).

H_arom_

H_arom_

H_arom_

H_arom_

H_arom_

H_arom_

C^6^H

C^5^H

C^2^H

C^6^H

C^5^H

C^2^H

CH_3_

CH_3_

CH_3_

CH_3_

Figure S352. ^1^Н-NMR spectrum of the compounds **4dd** and **4ed** (CDCl_3_, 400 MHz).

CF_3_

CH_3_

CH_3_

CH_3_

CH_3_

CH_3_

C^1^

C^1^

Figure S353. ^13^C-NMR spectrum of the compound **4dd** (CDCl_3_, 100 MHz).

CH_3_

CH_3_

CH_3_

CH_3_

CH_3_

Figure S354. DEPT spectrum of the compound **4dd** (CDCl_3_, 100 MHz).

CF_3_

CF_3_

CF_3_

CF_3_

Figure S355. ^19^F-NMR spectrum of the compounds **4dd** and **4ed** (CDCl_3_, 376 MHz).

Figure S356. NOESY H-H spectrum of the compounds **4dd**, **4ed** (CDCl_3_, 400 MHz).

Figure S357. NOESY H-F spectrum of the compounds **4dd**, **4ed** (CDCl_3_, 400 MHz).

H_arom_

H_arom_

H_arom_

H_arom_

C^6^H

C^6^H

C^5^H

C^5^H

C^2^H

CH_3_

CH_3_

CH_3_

CH_3_

CH_3_

H_arom_

H_arom_

C^2^H

Figure S358. ^1^Н-NMR spectrum of the compounds **4de** and **4ee** (CDCl_3_, 400 MHz).

CF_3_

CH_3_

CH_3_

CH_3_

C^1^

C^1^

Figure S359. ^13^C-NMR spectrum of the compound **4de** (CDCl_3_, 100 MHz).

CH_3_

CH_3_

CH_3_

Figure S360. DEPT spectrum of the compound **4de** (CDCl_3_, 100 MHz).

CF_3_

CF_3_

CF_3_

CF_3_

Figure S361. ^19^F-NMR spectrum of the compounds **4de** and **4ee** (CDCl_3_, 376 MHz).

Figure S362. NOESY H-H spectrum of the compounds **4de**, **4ee** (CDCl_3_, 400 MHz).

Figure S363. NOESY H-F spectrum of the compounds **4de**, **4ee** (CDCl_3_, 400 MHz).

H_arom_

H_arom_

H_arom_

H_arom_

H_arom_

H_arom_

H_arom_

H_arom_

H_arom_

C^2^H

H_arom_

C^2^H

CH_3_

CH_3_

CH_3_

CH_3_

CH_3_

CH_3_

Figure S364. ^1^Н-NMR spectrum of the compounds **4df**, **4ef** (CDCl_3_, 400 MHz).

C^1^

C^1^

CH_3_

CH_3_

CH_3_

CF_3_

Figure S365. ^13^C-NMR spectrum of the compound **4df** (CDCl_3_, 100 MHz).

CH_3_

CH_3_

CH_3_

Figure S366. DEPT spectrum of the compound **4df** (CDCl_3_, 100 MHz).

CF_3_

CF_3_

CF_3_

CF_3_

Figure S367. ^19^F-NMR spectrum of the compounds **4df** and **4ef** (CDCl_3_, 376 MHz).

Figure S368. NOESY H-H spectrum of the compounds **4df**, **4ef** (CDCl_3_, 400 MHz).

Figure S369. NOESY H-F spectrum of the compounds **4df** and **4ef** (CDCl_3_, 400 MHz).

H_arom_

H_arom_

H_arom_

H_arom_

H_arom_

H_arom_

H_arom_

H_arom_

H_arom_

H_arom_

H_arom_

C^2^H

C^2^H

CH_3_

CH_3_

CH_3_

CH_3_

CH_3_

CH_3_

CH_3_

CH_3_

H_arom_

Figure S370. ^1^Н-NMR spectrum of the compounds **4dg** and **4eg** (CDCl_3_, 400 MHz).

CF_3_

CF_3_

CH_3_

CH_3_

CH_3_

CH_3_

CH_3_

CH_3_

CH_3_

C^1^

C^1^

C^1^

C^1^

Figure S371. ^13^C-NMR spectrum of the compounds **4dg** and **4eg** (CDCl_3_, 100 MHz).

CH_3_

CH_3_

CH_3_

CH_3_

Figure S372. DEPT spectrum of the compounds **4dg** and **4eg** (CDCl_3_, 100 MHz).

CF_3_

CF_3_

CF_3_

CF_3_

Fig. S373. ^19^F-NMR spectrum of the compounds **4dg**, **4eg** (DMSO-d_6_, 376 MHz, 100 °C).

H_arom._

H_arom._

H_arom._

H_arom._

H_arom._

H_arom._

H_arom._

H_arom._

H_arom._

H_arom._

H_arom._

H_arom._

C^2^H

C^2^H

CH_3_

CH_3_

CH_3_

CH_3_

CH_3_

CH_3_

CH_3_

CH_3_

Figure S374. ^1^Н-NMR spectrum of the compounds **4dh** and **4eh** (CDCl_3_, 400 MHz).

CF_3_

CF_3_

CH_3_

CH_3_

CH_3_

CH_3_

CH_3_

C^1^

C^1^

C^1^

CH_3_

CH_3_

CH_3_

Figure S375. ^13^C-NMR spectrum of the compounds **4dh** and **4eh** (CDCl_3_, 100 MHz).

CH_3_

CH_3_

CH_3_

CH_3_

CH_3_

CH_3_

CH_3_

Figure S376. DEPT spectrum of the compounds **4dh** and **4eh** (CDCl_3_, 100 MHz).

CF_3_

CF_3_

CF_3_

CF_3_

Figure S377. ^19^F-NMR spectrum of the compounds **4dh** and **4eh** (CDCl_3_, 376 MHz).

H_arom_

H_arom_

C^2^H

C^2^H

H_arom_

H_arom_

H_arom_

H_arom_

CH_3_

CH_3_

CH_3_

CH_3_

CH_3_

CH_3_

CH_3_

CH_3_

H_arom_

H_arom_

Figure S378. ^1^Н-NMR spectrum of the compounds **4di** and **4ei** (CDCl_3_, 400 MHz).

CF_3_

C^1^

CH_3_

CH_3_

CH_3_

C^1^

Figure S379. ^13^C-NMR spectrum of the compound **4di** (CDCl_3_, 100 MHz).

CH_3_

CH_3_

CH_3_

Figure S380. DEPT spectrum of the compound **4di** (CDCl_3_, 100 MHz).

CF_3_

CF_3_

CF_3_

CF_3_

Figure S381. ^19^F-NMR spectrum of the compounds **4di** and **4ei** (CDCl_3_, 376 MHz).

Figure S382. NOESY H-H spectrum of the compounds **4di**, **4ei** (CDCl_3_, 400 MHz).

Figure S383. NOESY H-F spectrum of the compounds **4di** and **4ei** (CDCl_3_, 400 MHz).

H_arom_

H_arom_

H_arom_

H_arom_

H_arom_

H_arom_

C^5^H

C^2^H

C^6^H

C^2^H

CH_3_

CH_3_

CH_3_

CH_3_

CH_3_

CH_3_

Figure S384. ^1^Н-NMR spectrum of the compounds **4dj** and **4ej** (CDCl_3_, 400 MHz).

CF_3_

CH_3_

CH_3_

CH_3_

C^1^

C^1^

Figure S385. ^13^C-NMR spectrum of the compound **4dj** (CDCl_3_, 100 MHz).

CH_3_

CH_3_

CH_3_

Figure S386. DEPT spectrum of the compound **4dj** (CDCl_3_, 100 MHz).

CF_3_

CF_3_

CF_3_

CF_3_

Figure S387. ^19^F-NMR spectrum of the compounds **4dj** and **4ej** (CDCl_3_, 376 MHz).

H_arom_

CH_3_

CH_3_

CH_3_

CH_3_

CH_3_

CH_3_

C^2^H

C^2^H

C^6^H

C^5^H

H_arom_

H_arom_

H_arom_

H_arom_

H_arom_

H_arom_

H_arom_

Figure S388. ^1^Н-NMR spectrum of the compounds **4dk** and **4ek** (CDCl_3_, 400 MHz).

CF_3_

C^1^

C^1^

CH_3_

CH_3_

CH_3_

Figure S389. ^13^C-NMR spectrum of the compound **4dk** (CDCl_3_, 100 MHz).

CH_3_

CH_3_

CH_3_

Figure S390. DEPT spectrum of the compound **4dk** (CDCl_3_, 100 MHz).

CF_3_

CF_3_

CF_3_

CF_3_

Figure S391. ^19^F-NMR spectrum of the compounds **4dk** and **4ek** (CDCl_3_, 376 MHz).

Figure S392. NOESY H-H spectrum of the compounds **4dk**, **4ek** (CDCl_3_, 400 MHz).

H_arom_

H_arom_

H_arom_

H_arom_

H_arom_

H_arom_

H_arom_

H_arom_

H_arom_

H_arom_

C^5^H

C^6^H

C^2^H

C^2^H

C^6^H

C^2^H

C^5^H

C^2^H

CH_3_

CH_3_

CH_3_

CH_3_

CH_3_

CH_3_

CH_3_

CH_3_

CH_3_

CH_3_

Figure S393. ^1^Н-NMR spectrum of the compounds **4dl** and **4el** (CDCl_3_, 400 MHz).

CF_3_

CH_3_

CH_3_

CH_3_

C^1^

C^1^

Figure S394. ^13^C-NMR spectrum of the compound **4dl** (CDCl_3_, 100 MHz).

CH_3_

CH_3_

CH_3_

Figure S395. DEPT spectrum of the compound **4dl** (CDCl_3_, 100 MHz).

CF_3_

CF_3_

CF_3_

CF_3_

Figure S396. ^19^F-NMR spectrum of the compounds **4dl** and **4el** (CDCl_3_, 376 MHz).

C^2^H

C^2^H

C^6^H

C^5^H

H_arom_

H_arom_

CH_3_

CH_3_

CH_3_

CH_3_

CH_3_

C^5^H

C^6^H

H_arom_

H_arom_

H_arom_

H_arom_

H_arom_

Figure S397. ^1^Н-NMR spectrum of the compounds **4dm** and **4em** (CDCl_3_, 400 MHz).

CF_3_

CF_3_

C^1^

C^1^

CH_3_

CH_3_

CH_3_

CH_3_

CH_3_

C^1^

Figure S398. ^13^C-NMR spectrum of the compounds **4dm** and **4em** (CDCl_3_, 100 MHz).

CH_3_

CH_3_

CH_3_

CH_3_

CH_3_

Figure S399. DEPT spectrum of the compounds **4dm** and **4em** (CDCl_3_, 100 MHz).

CF_3_

CF_3_

CF_3_

CF_3_

Figure S400. ^19^F-NMR spectrum of the compounds **4dm** and **4em** (CDCl_3_, 376 MHz).

Figure S401. NOESY H-H spectrum of the compounds **4dm**, **4em** (CDCl_3_, 400 MHz).

Figure S402. NOESY H-F spectrum of the compounds **4dm**, **4em** (CDCl_3_, 400 MHz).

C^2^H

H_arom_

H_arom_

H_arom_

H_arom_

H_arom_

H_arom_

H_arom_

H_arom_

H_arom_

H_arom_

C^2^H

CH_3_

CH_3_

CH_3_

CH_3_

Figure S403. ^1^Н-NMR spectrum of the compounds **4dn** and **4en** (CDCl_3_, 400 MHz).

CF_3_

C^1^

C^1^

CH_3_

CH_3_

CH_3_

Figure S404. ^13^C-NMR spectrum of the compound **4dn** (CDCl_3_, 100 MHz).

CH_3_

CH_3_

CH_3_

Figure S405. DEPT spectrum of the compound **4dn** (CDCl_3_, 100 MHz).

CF_3_

CF_3_

CF_3_

CF_3_

Figure S406. ^19^F-NMR spectrum of the compounds **4dn** and **4en** (CDCl_3_, 376 MHz).

CH_3_

H_arom_

H_arom_

H_arom_

H_arom_

H_arom_

H_arom_

H_arom_

H_arom_

H_arom_

H_arom_

H_arom_

H_arom_

C^2^H

C^2^H

CH_3_

CH_3_

CH_3_

CH_3_

Figure S407. ^1^Н-NMR spectrum of the compounds **4do** and **4eo** (CDCl_3_, 400 MHz).

CF_3_

C^1^

C^1^

CH_3_

CH_3_

CH_3_

Figure S408. ^13^C-NMR spectrum of the compound **4do** (CDCl_3_, 100 MHz).

CH_3_

CH_3_

CH_3_

Figure S409. DEPT spectrum of the compound **4do** (CDCl_3_, 100 MHz).

CF_3_

CF_3_

CF_3_

CF_3_

Figure S410. ^19^F-NMR spectrum of the compounds **4do** and **4eo** (CDCl_3_, 376 MHz).
