# Supplementary material for: TfOH-Promoted Reaction of 2,4-Diaryl-1,1,1-Trifluorobut-3-yn-2-oles with Arenes: Synthesis of 1,3-Diaryl-1-CF3-Indenes and Versatility of the Reaction Mechanisms
Source: Molecules. 2018 Nov 25;23(12):3079. doi: 10.3390/molecules23123079 (PMC6321356; doi:10.3390/molecules23123079)
Supplement: Supplementary file 1 [file molecules-23-03079-s001.zip › supplyment/SI part 1-edited.doc]

*Supporting Information*

**TfOH-Promoted Reaction of 2,4-Diaryl-1,1,1-Trifluorobut-3-yn-2-oles with Arenes: Synthesis of 1,3-Diaryl-1-CF_3_-Indenes and Versatility of the Reaction Mechanisms**

Aleksey V. Zerov ^1^, Anna N. Kazakova ^1^, Irina A. Boyarskaya ^1^, Taras L. Panikorovsky ^2^, Vitalii V. Suslonov ^2^, Olesya V. Khoroshilova ^2^ and Aleksander V. Vasilyev ^1,3,^*

^1^	Department of Organic Chemistry, Institute of Chemistry, Saint Petersburg State University, Universitetskaya nab., 7/9, Saint Petersburg 199034, Russia; [lfdse@mail.ru](mailto:lfdse@mail.ru) (A.V.Z.); [a.kazakova0@gmail.com](mailto:a.kazakova0@gmail.com) (A.N.K.); [iralbo@yahoo.com](mailto:iralbo@yahoo.com) (I.A.B.)

^2^	Center for X-ray Diffraction Studies, Research park, St. Petersburg State University, Universitetskiy pr. 26, Saint Petersburg, Petrodvoretz 198504, Russia; [taras.panikorovsky@spbu.ru](mailto:taras.panikorovsky@spbu.ru) (T.L.P.); [v.suslonov@spbu.ru](mailto:v.suslonov@spbu.ru) (V.V.S.); [o_khorosh@mail.ru](mailto:o_khorosh@mail.ru) (O.V.K.)

^3^	Department of Chemistry, Saint Petersburg State Forest Technical University, Institutsky per., 5, Saint Petersburg 194021, Russia.

*****	Correspondence: [aleksvasil@mail.ru](mailto:aleksvasil@mail.ru); Tel.: +07-812–670-9352; Fax: +07-812–670-9390

**Table of Contents:**

General Information……………………………………………………………..S2 (SI part 1)

Preparation and Characterization of Starting Materials…………………………S3 (SI part 1)

General Procedure and Characterization Data.………………………………….S12 (SI part 1)

References………..……………………………………………………………...S52 (SI part 1)

Original-NMR Spectra (^1^H, ^13^C, DEPT, ^19^F, NOESY-HH and NOESY-HF)…..S53 (SI part 2,3)

X-Ray Data……………. ………………………………………...……………..S314 (SI part 3)

DFT Calculations of Cations **Aa**, **Ba**…………………........................................S327 (SI part 3)

**General Information**

The-NMR spectra of solutions of compounds in CDCl_3_ were recorded on a Bruker AVANCE III 400 (at 400, 376, and 100 MHz for ^1^H, ^19^F and, ^13^C-NMR spectra, respectively) spectrometer at 25 °C. The residual proton-solvent peak CDCl_3_ (δ 7.26 ppm) for ^1^H-NMR spectra and the carbon signal of CDCl_3_ (δ 77.0 ppm) for ^13^C-NMR spectra were used as references. ^19^F-NMR spectra were indirectly referred to the signal of CFCl_3_ (δ 0.0 ppm). HRMS was carried out with the instruments Bruker maXis HRMS-ESI-QTOF and Varian 902-MS MALDI Mass Spectrometer. Chromato-mass-spectrometry data were obtained with a Shimadzu QP-2010 Ultra with a SPB-1 SULFUR capillary column (30 m × 0.32 mm), with thickness of the stationary phase being 1.25 μm. The preparative reactions were monitored by thin-layer chromatography carried out on silica gel plates (Alugram SIL G/UV-254), using UV light for detection. Preparative TLC was performed on silica gel Chemapol L 5/40 with petroleum ether–ethyl acetate mixture elution.

Single crystal X-ray analysis was performed with a single crystal diffractometer Agilent Technologies (Oxford Diffraction) «Supernova». A suitable crystal was selected and studied on the diffractometer. The crystal was kept at 100(2) K during data collection. Using Olex2^1^ the structure was solved with the ShelXS^2^ structure solution program using Direct Methods and refined with the ShelXL refinement package using Least Squares minimisation. CCDC 1568593 —(**4bg**), CCDC 1578216—(**4ci**), CCDC 1568602—(**4dc**), CCDC 1568599—(**4de**), CCDC 1568594—(**4dg**), CCDC 1563374—(**4di**), CCDC 1568596—(**4dk**), CCDC 1568600—(**4dm**), CCDC 1568595—(**4fb**), CCDC 1568597—(**4fc**), CCDC 1568603—(**4fh**), CCDC 1568598—(**4fk**), CCDC 1568601—(**5ac**), contain the supplementary crystallographic data, which can be obtained free of charge at www.ccdc.cam.ac.uk/conts/retrieving.html or from the Cambridge Crystallographic Data Centre, 12 Union Road, Cambridge CB2 1EZ, UK; Fax: (internat.) + 44-1223-336-033; E-mail: [deposit@ccdc.cam.ac.uk](mailto:deposit@ccdc.cam.ac.uk).

**DFT calculations.** All computations were carried out at the DFT/HF hybrid level of theory using Becke’s three-parameter hybrid exchange functional in combination with the gradient-corrected correlation functional of Lee, Yang, and Parr (B3LYP) by using GAUSSIAN 2003 program packages.^3^ The geometries optimization were performed using the 6-311+G(2d,2p) basis set (standard 6-311 basis set added with polarization (d, p) and diffuse functions). Optimizations were performed on all degrees of freedom and solvent-phase optimized structures were verified as true minima with no imaginary frequencies. The Hessian matrix was calculated analytically for the optimized structures in order to prove the location of correct minima and to estimate the thermodynamic parameters. Gibbs free energies were calculated for 25°С. Solvent-phase calculations used the Polarizable Continuum Model (PCM).

**Preparation and Characterization of Starting Materials**

The CF_3_-propargyl alcohols were synthesized via a three-step procedure involving: 1) a Sonogashira cross-coupling of acetylenes with acid chlorides^4^; 2) Trifluoromethylation of 1,3-diarylpropynones with CF_3_SiMe_3_^5,6^; 3) Detrimethylsilylation of the corresponding trimethylsilyl ether of 2,4-diaryl-1,1,1-trifluorobut-3-yn-2-ols under aqueous HCl^5^ or with SnCl_2_^7^.

**Trimethylsilyl ether of 2,4-diphenyl-1,1,1-trifluorobut-3-yn-2-ol (Ia)**: Yellow oil. The reaction scale is 500 mg (3.73 mmol) 1,3-diphenylprop-2-yn-1-one, isolated amount is 781 mg, 60% yield. ^1^Н-NMR (CDCl_3_, 400 MHz) δ, ppm: 0.25 s (9H, CH_3_), 7.36–7.43 m (6H_Ph_), 7.54–7.56 m (2H_Ph_), 7.79–7.81 m (2H_Ph_). ^13^C-NMR (CDCl_3_, 100 MHz) δ, ppm: 1.5 (CH_3_), 74.7 (C^2^, *J*_C-F_ 32.2 Hz), 85.3 (C^4^), 89.0 (C^3^), 121.5, 123.5 q (C^1^, *J*_C-F_ 286.0 Hz), 127.6 q (*J*_C-F_ 0.8 Hz), 128.1, 128.7, 129.3, 129.6, 132.0, 137.7. ^19^F MNR (CDCl_3_, 376 MHz) δ, ppm: −80.63 q (CF_3_). HRMS (ESI): С_19_H_19_F_3_OSiAg found 455.0208 [M + Ag]^+^; calcd. 455.0203.

**Trimethylsilyl ether of 2-(3-methylphenyl)-4-phenyl-1,1,1-trifluorobut-3-yn-2-ol (Ib):** Yellow oil. The reaction scale is 394 mg (1.8 mmol) 1-(3-methylphenyl)-3-phenylprop-2-yn-1-one, isolated amount is 550 mg, 84% yield. ^1^Н-NMR (CDCl_3_, 400 MHz) δ, ppm: 0.25 s (9H, Si(CH_3_)_3_), 2.42 s (3H, CH_3_), 7.21 d (1H_arom._, ^3^*J* 7.5 Hz), 7.30 t (1H_arom._, ^3^*J* 7.5 Hz), 7.36–7.42 m (3H_arom._), 7.53–7.57 m (2H_arom._), 7.58 s (1H_arom._), 7.58–7.60 m (1H_arom._). ^13^C-NMR (CDCl_3_, 100 MHz) δ, ppm: 1.5 (Si(CH_3_)_3_), 21.7 (CH_3_), 74.9 q (C^2^, *J*_C-F_ 32.9 Hz), 85.5 (C^4^), 88.9 (C^3^), 121.6, 123.5 q (C^1^, *J*_C-F_ 285.9 Hz), 124.7, 127.8, 128.2 q (*J*_C-F_ 0.6 Hz), 128.7, 129.5, 130.0, 132.0, 137.6, 137.8. ^19^F MNR (CDCl_3_, 376 MHz) δ, ppm: −80.57 s (CF_3_). HRMS (ESI): С_20_H_21_F_3_OSiAg found 469.0371 [M + Ag]^+^; calcd. 469.0360.

**Trimethylsilyl ether of 2-(4-methylphenyl)-4-phenyl-1,1,1-trifluorobut-3-yn-2-ol (Ic):** Pale yellow oil. The reaction scale is 213 mg (0.97 mmol) 1-(4-methylphenyl)-3-phenylprop-2-yn-1-one, isolated amount is 332 mg, 95% yield. ^1^Н-NMR (CDCl_3_, 400 MHz) δ, ppm: 0.25 s (9H, Si(CH_3_)_3_), 2.40 (3H, CH_3_), 7.23 d (2H_arom._, ^3^*J* 8.0 Hz), 7.36–7.42 m (3H_arom._), 7.55 m (2H_arom._), 7.68 d (2H_arom._, ^3^*J* 8.0 Hz). ^13^C-NMR (CDCl_3_, 100 MHz) δ, ppm: 1.5 (Si(CH_3_)_3_), 21.3 (CH_3_), 74.8 q (C^2^, *J*_C-F_ 32.9 Hz), 85.5 (C^4^), 88.9 (C^3^), 121.6, 123.5 q (C^1^, *J*_C-F_ 285.9 Hz), 127.5, 128.7, 128.9, 129.5, 132.0, 134.8, 139.2. ^19^F MNR (CDCl_3_, 376 MHz) δ, ppm: −80.74 s (CF_3_). HRMS (ESI): С_20_H_21_F_3_OSiAg found 469.0369 [M + Ag]^+^; calcd. 469.0360.

**Trimethylsilyl ether of 2-(3,4-dimethylphenyl)-4-phenyl-1,1,1-trifluorobut-3-yn-2-ol (Id):** Pale yellow oil. The reaction scale is 355 mg (1.52 mmol) 1-(3,4-dimethylphenyl)-3-phenylprop-2-yn-1-one, isolated amount is 524 mg, 92% yield. ^1^Н-NMR (CDCl_3_, 400 MHz) δ, ppm: 0.24 s (9H, Si(CH_3_)_3_), 2.30 (3H, CH_3_), 2.32 (3H, CH_3_), 7.17 d (1H_arom._, ^3^*J* 7.8 Hz), 7.36-7.43 m (3H_arom._), 7.50–7.56 m (4H_arom._). ^13^C-NMR (CDCl_3_, 100 MHz) δ, ppm: 1.5 (Si(CH_3_)_3_), 19.7 (CH_3_), 20.2 (CH_3_), 74.8 q (C^2^, *J*_C-F_ 32.4 Hz), 85.6 (C^4^), 88.7 (C^3^), 121.7, 123.6 q (C^1^, *J*_C-F_ 286.0 Hz), 125.0, 128.6, 128.7, 129.4, 129.4, 132.0, 135.1, 136.3, 137.8. ^19^F MNR (CDCl_3_, 376 MHz) δ, ppm: −80.67 s (CF_3_). HRMS (ESI): С_21_H_23_F_3_OSiAg found 483.0512 [M + Ag]^+^; calcd. 483.0516.

**Trimethylsilyl ether of 2-(4-chlorophenyl)-4-phenyl-1,1,1-trifluorobut-3-yn-2-ol (Ie):** Yellow oil. The reaction scale is 330 mg (1.37 mmol) 1-(4-chlorophenyl)-3-phenylprop-2-yn-1-one, isolated amount is 488 mg, 93% yield. ^1^Н-NMR (CDCl_3_, 400 MHz) δ, ppm: 0.24 s (9H, Si(CH_3_)_3_), 7.36–7.43 m (5H_arom._), 7.53–7.55 m (2H_arom._), 7.72 d (2H_arom_, *^3^J* 8.5 Hz). ^13^C-NMR (CDCl_3_, 100 MHz) δ, ppm: 1.5 (Si(CH_3_)_3_), 74.7 q (C^2^, *J*_C-F_ 32.7 Hz), 84.7 (C^4^), 89.4 (C^3^), 121.2, 123.3 q (C^1^, *J*_C-F_ 285.8 Hz), 128.4, 128.7, 129.1 d (*J*_C-F_ 0.5 Hz), 129.7, 132.0, 135.5, 136.4. ^19^F MNR (CDCl_3_, 376 MHz) δ, ppm: −80.74 s (CF_3_). HRMS (ESI): С_19_H_18_ClF_3_OSiAg found 488.9800 [M + Ag]^+^; calcd. 488.9813.

**Trimethylsilyl ether of 2-(4-bromophenyl)-4-phenyl-1,1,1-trifluorobut-3-yn-2-ol (If):** Yellow oil. The reaction scale is 466 mg (1.64 mmol) 1-(4-bromophenyl)-3-phenylprop-2-yn-1-one, isolated amount is 677 mg, 97% yield. ^1^Н-NMR (CDCl_3_, 400 MHz) δ, ppm: 0.25 s (9H, Si(CH_3_)_3_), 7.36–7.43 m (3H_arom._), 7.53–7.56 m (4H_arom._), 7.66 d (2H_arom._, ^3^*J* 8.5 Hz). ^13^C-NMR (CDCl_3_, 100 MHz) δ, ppm: 1.5 (Si(CH_3_)_3_), 74.6 q (C^2^, *J*_C-F_ 32.5 Hz), 84.7 (C^4^), 89.4 (C^3^), 121.2, 123.3 q (C^1^, *J*_C-F_ 285.8 Hz), 123.8, 128.7, 129.4, 129.7, 131.4, 132.0, 136.9. ^19^F MNR (CDCl_3_, 376 MHz) δ, ppm: −80.72 s (CF_3_). HRMS (ESI): С_19_H_18_BrF_3_OSiAg found 532.9287 [M + Ag]^+^; calcd. 532.9308.

**Trimethylsilyl ether of 2-(2-bromophenyl)-4-phenyl-1,1,1-trifluorobut-3-yn-2-ol (Ig):** Pale yellow oil. The reaction scale is 560 mg (1.96 mmol) 1-(2-bromophenyl)-3-phenylprop-2-yn-1-one, isolated amount is 750 mg, 90% yield. ^1^Н-NMR (CDCl_3_, 400 MHz) δ, ppm: 0.29 s (9H, Si(CH_3_)_3_), 7.22 dt (1H_arom._, ^3^*J* 7.9 Hz, ^4^*J* 1.3 Hz), 7.36–7.42 m (4H_arom._), 7.55–7.57 m (2H_arom._), 7.69 dd (1H_arom._, ^3^*J* 7.9 Hz, ^4^*J* 1.3 Hz), 7.98 dd (1H_arom._, ^3^*J* 7.9 Hz, ^4^*J* 1.3 Hz). ^13^C-NMR (CDCl_3_, 100 MHz) δ, ppm: 1.6 (Si(CH_3_)_3_), 75.1 q (C^2^, *J*_C-F_ 32.9 Hz), 84.5 (C^4^), 90.8 (C^3^), 121.6, 122.0, 123.6 q (C^1^, *J*_C-F_ 287.4 Hz), 127.2, 128.7, 129.6, 130.6, 131.1, 131.8, 135.4, 136.2. ^19^F MNR (CDCl_3_, 376 MHz) δ, ppm: −78.56 s (CF_3_). HRMS (ESI): С_19_H_18_BrF_3_OSiAg found 532.9299 [M + Ag]^+^; calcd. 532.9308.

**Trimethylsilyl ether of 2-(4-nitrophenyl)-4-phenyl-1,1,1-trifluorobut-3-yn-2-ol (Ih):** Orange solid, mp 70°С. The reaction scale is 500 mg (2 mmol) 1-(4-nitrophenyl)-3-phenylprop-2-yn-1-one, isolated amount is 407 mg, 52% yield. ^1^Н-NMR (CDCl_3_, 400 MHz) δ, ppm: 0.27 s (9H, Si(CH_3_)_3_), 7.38–7.45 m (3H_arom._), 7.53–7.56 m (2H_arom_), 7.98 d (2H_arom._, ^3^*J* 8.8 Hz), 8.28 d (2H_arom._, ^3^*J* 8.8 Hz). ^13^C-NMR (CDCl_3_, 100 MHz) δ, ppm: 1.4 (Si(CH_3_)_3_), 74.5 q (C^2^, *J*_C-F_ 32.9 Hz), 83.9 (C^4^), 90.2 (C^3^), 120.8, 123.0 q (C^1^, *J*_C-F_ 286.4 Hz), 123.4, 128.8, 128.8, 130.0, 132.0, 144.7, 148.7. ^19^F MNR (CDCl_3_, 376 MHz) δ, ppm: −80.31 s (CF_3_). HRMS (ESI): С_19_H_18_F_3_NO_3_SiAg found 500.0040 [M + Ag]^+^; calcd. 500.0054.

**Trimethylsilyl ether of 4-(4-methylphenyl)-2-phenyl-1,1,1-trifluorobut-3-yn-2-ol (Ii):** Orange solid, mp 52°С. The reaction scale is 200 mg (0.91 mmol) 3-(4-methylphenyl)-1-phenylprop-2-yn-1-one, isolated amount is 257 mg, 78% yield. ^1^Н-NMR (CDCl_3_, 400 MHz) δ, ppm: 0.24 s (9H, Si(CH_3_)_3_), 2.39 (3H, CH_3_), 7.19 d (2H_arom._, ^3^*J* 8.0 Hz), 7.40–7.42 m (3H_arom._), 7.45 d (2H_arom._, ^3^*J* 8.0 Hz), 7.79–7.81 m (2H_arom._). ^13^C-NMR (CDCl_3_, 100 MHz) δ, ppm: 1.5 (Si(CH_3_)_3_), 21.7 (CH_3_), 74.9 q (C^2^, *J*_C-F_ 32.4 Hz), 84.7 (C^4^), 89.2 (C^3^), 118.5, 123.5 q (C^1^, *J*_C-F_ 285.7 Hz), 127.6, 128.1, 129.2, 129.4, 131.9, 137.8, 139.9. ^19^F MNR (CDCl_3_, 376 MHz) δ, ppm: −80.64 s (CF_3_). HRMS (ESI): С_20_H_21_F_3_OSiAg found 469.0366 [M + Ag]^+^; calcd. 469.0360.

**Trimethylsilyl ether of 4-(2,4-dimethylphenyl)-2-phenyl-1,1,1-trifluorobut-3-yn-2-ol (Ij):** Yellow oil. The reaction scale is 244 mg (1.04 mmol) 3-(2,4-dimethylphenyl)-1-phenylprop-2-yn-1-one, isolated amount is 317 mg, 81% yield. ^1^Н-NMR (CDCl_3_, 400 MHz) δ, ppm: 0.23 s (9H, Si(CH_3_)_3_), 2.36 s (3H, CH_3_), 2.48 s (3H, CH_3_), 7.01 d (1H_arom._, ^3^*J* 7.8 Hz), 7.09 s (1H_arom._), 7.38–7.44 m (4H_arom._), 7.80–7.82 m (2H_arom._). ^13^C-NMR (CDCl_3_, 100 MHz) δ, ppm: 1.5 (Si(CH_3_)_3_), 20.7 (CH_3_), 21.6 (CH_3_), 75.0 q (C^2^, *J*_C-F_ 32.3 Hz), 88.3 (C^4^), 88.5 (C^3^), 118.3, 123.6 q (C^1^, *J*_C-F_ 285.9 Hz), 126.7, 127.7, 128.1, 129.2, 130.7, 132.3, 137.9, 139.8, 141.1. ^19^F MNR (CDCl_3_, 376 MHz) δ, ppm: −80.67 s (CF_3_). HRMS (ESI): С_21_H_23_F_3_OSiAg found 483.0511 [M + Ag]^+^, calcd. 483.0516.

**Trimethylsilyl ether of 4-(3,4-dimethylphenyl)-2-phenyl-1,1,1-trifluorobut-3-yn-2-ol (Ik):** Colorless solid, mp 92°С. The reaction scale is 244 mg (1.04 mmol) 3-(3,4-dimethylphenyl)-1-phenylprop-2-yn-1-one, isolated amount is 317 mg, 94% yield. ^1^Н-NMR (CDCl_3_, 400 MHz) δ, ppm: 0.25 s (9H, Si(CH_3_)_3_), 2.29 s (3H, CH_3_), 2.31 s (3H, CH_3_), 7.15 d (1H_arom._, ^3^*J* 7.7 Hz), 7.30 dd (1H_arom._, ^3^*J* 7.7 Hz, ^4^*J* 1.3 Hz), 7.33 s (1H_arom._), 7.38–7.46 m (3H_arom._), 7.77–7.84 m (2H_arom._). ^13^C-NMR (CDCl_3_, 100 MHz) δ, ppm: 1.5 (Si(CH_3_)_3_), 19.7 (CH_3_), 20.0 (CH_3_), 74.9 q (C^2^, *J*_C-F_ 36.2 Hz), 84.4 (C^4^), 89.4 (C^3^), 118.8, 123.5 q (C^1^, *J*_C-F_ 285.9 Hz), 127.6, 128.1, 129.2, 129.5, 130.0, 132.9, 137.1, 137.9, 138.7. ^19^F MNR (CDCl_3_, 376 MHz) δ, ppm: −80.64 s (CF_3_). HRMS (ESI): С_21_H_23_F_3_OSiAg found 483.0507 [M + Ag]^+^; calcd. 483.0516.

**Trimethylsilyl ether of 4-(4-chlorophenyl)-2-phenyl-1,1,1-trifluorobut-3-yn-2-ol (Il):** Pale yellow oil. The reaction scale is 114 mg (0.47 mmol) 3-(4-chlorophenyl)-1-phenylprop-2-yn-1-one, isolated amount is 155 mg, 86% yield. ^1^Н-NMR (CDCl_3_, 400 MHz) δ, ppm: 0.23 s (9H, Si(CH_3_)_3_), 7.36 d (2H_arom._, ^3^*J* 8.6 Hz), 7.39-7.43 m (3H_arom._), 7.47 d (2H_arom._, ^3^*J* 8.6 Hz), 7.74–7.77 m (2H_arom_). ^13^C-NMR (CDCl_3_, 100 MHz) δ, ppm: 1.5 (Si(CH_3_)_3_), 74.9 q (C^2^, *J*_C-F_ 32.5 Hz), 86.3 (C^4^), 87.8 (C^3^), 119.9, 123.4 q (C^1^, *J*_C-F_ 286.0 Hz), 127.5, 128.2, 129.1, 129.4, 133.2, 135.8, 137.4. ^19^F MNR (CDCl_3_, 376 MHz) δ, ppm: −80.59 s (CF_3_). HRMS (ESI): С_19_H_18_ClF_3_OSiAg found 488.9804 [M + Ag]^+^; calcd. 488.9813.

**Trimethylsilyl ether of 4-(4-bromophenyl)-2-phenyl-1,1,1-trifluorobut-3-yn-2-ol (Im):** Yellow oil. The reaction scale is 168 mg (0.6 mmol) 3-(4-bromophenyl)-1-phenylprop-2-yn-1-one, isolated amount is 189 mg, 75% yield. ^1^Н-NMR (CDCl_3_, 400 MHz) δ, ppm: 0.23 s (9H, Si(CH_3_)_3_), 7.39–7.43 m (5H_arom._), 7.53 d (2H_arom._, ^3^*J* 8.5 Hz), 7.76–7.77 m (2H_arom_). ^13^C-NMR (CDCl_3_, 100 MHz) δ, ppm: 1.5 (Si(CH_3_)_3_), 74.9 q (C^2^, *J*_C-F_ 32.6 Hz), 86.5 (C^4^), 87.9 (C^3^), 120.4, 123.4 q (C^1^, *J*_C-F_ 285.9 Hz), 124.1, 127.5, 128.2, 129.4, 132.1, 133.4, 137.4. ^19^F MNR (CDCl_3_, 376 MHz) δ, ppm: −80.58 s (CF_3_). HRMS (ESI): С_19_H_18_BrF_3_OSiAg found 532.9276 [M + Ag]^+^; calcd. 532.9308.

**Trimethylsilyl ether of 4-(4-nitrophenyl)-2-phenyl-1,1,1-trifluorobut-3-yn-2-ol (In):** Colorless oil. The reaction scale is 300 mg (1.2 mmol) 3-(4-nitrophenyl)-1-phenylprop-2-yn-1-one, isolated amount is 189 mg, 40% yield. ^1^Н-NMR (CDCl_3_, 400 MHz) δ, ppm: 0.24 s (9H, Si(CH_3_)_3_), 7.41–7.45 m (3H_arom._), 7.70 d (2H_arom._, ^3^*J* 8.9 Hz), 7.74–7.76 m (2H_arom._), 8.26 d (2H_arom._, ^3^*J* 8.9 Hz). ^13^C-NMR (CDCl_3_, 100 MHz) δ, ppm: 1.5 (Si(CH_3_)_3_), 75.0 q (C^2^, *J*_C-F_ 32.5 Hz), 86.7 (C^4^), 90.2 (C^3^), 123.3 q (C^1^, *J*_C-F_ 286.1 Hz), 124.0, 127.4, 128.0, 128.3, 129.6, 132.9, 136.9, 148.1. ^19^F MNR (CDCl_3_, 376 MHz) δ, ppm: −80.44 s (CF_3_). HRMS (ESI): С_19_H_18_F_3_NO_3_SiAg found 500.0057 [M + Ag]^+^; calcd. 500.0054.

**Trimethylsilyl ether of 2,4-bis(4-methylphenyl)-1,1,1-trifluorobut-3-yn-2-ol (Io):** Pale yellow oil. The reaction scale is 78 mg (0.33 mmol) 1,3-bis(4-metylphenyl)prop-2-yn-1-one, isolated amount is 103 mg, 83% yield. ^1^Н-NMR (CDCl_3_, 400 MHz) δ, ppm: 0.23 s (9H, Si(CH_3_)_3_), 2.39 s (6H, CH_3_), 7.18 d (2H_arom._, ^3^*J* 8.1 Hz), 7.21 d (2H_arom._, ^3^*J* 8.1 Hz), 7.43 d (2H_arom._, ^3^*J* 8.1 Hz), 7.66 d (2H_arom._, ^3^*J* 8.1 Hz). ^13^C-NMR (CDCl_3_, 100 MHz) δ, ppm: 1.5 (Si(CH_3_)_3_), 21.3 (CH_3_), 21.7 (CH_3_), 74.8 q (C^2^, *J*_C-F_ 32.3 Hz), 84.8 (C^4^), 89.1 (C^3^), 118.6, 123.6 q (C^1^, *J*_C-F_ 285.7 Hz), 127.5, 128.8, 129.4, 131.9, 134.9, 139.1, 139.8. ^19^F MNR (CDCl_3_, 376 MHz) δ, ppm: −80.76 s (CF_3_). HRMS (ESI): С_21_H_23_F_3_OSiAg found 483.0516 [M + Ag]^+^; calcd. 483.0516.

**Trimethylsilyl ether of 2,4-bis(3,4-dimethylphenyl)-1,1,1-trifluorobut-3-yn-2-ol (Ip):** Pale yellow oil. The reaction scale is 99 mg (0.38 mmol) 1,3-bis(3,4-dimetylphenyl)prop-2-yn-1-one, isolated amount is 115 mg, 75% yield. ^1^Н-NMR (CDCl_3_, 400 MHz) δ, ppm: 0.24 s (9H, Si(CH_3_)_3_), 2.28 (3H, CH_3_), 2.30 (6H, CH_3_), 2.32 (3H, CH_3_), 7.14 d (1H_arom._, ^3^*J* 7.8 Hz), 7.17 d (1H_arom._, ^3^*J* 7.8 Hz), 7.29 d (2H_arom._, ^3^*J* 7.8 Hz), 7.32 s (1H_arom._), 7.50–7.53 m (1H_arom._), 7.53 s (1H_arom._). ^13^C-NMR (CDCl_3_, 100 MHz) δ, ppm: 1.5 (Si(CH_3_)_3_), 19.7 (CH_3_), 19.7 (CH_3_), 20.0 (CH_3_), 20.1 (CH_3_), 74.8 q (C^2^, *J*_C-F_ 32.4 Hz), 84.7 (C^4^), 89.1 (C^3^), 119.0, 123.6 q (C^1^, *J*_C-F_ 285.7 Hz), 125.0, 128.7, 129.4, 129.5, 129.9, 132.9, 135.3, 136.2, 137.1, 137.7, 138.5. ^19^F MNR (CDCl_3_, 376 MHz) δ, ppm: −80.69 s (CF_3_). HRMS (ESI): С_23_H_27_F_3_OSiAg found 511.0817 [M + Ag]^+^; calcd. 511.0829.

**Trimethylsilyl ether of 2,4-bis(4-chlorophenyl)-1,1,1-trifluorobut-3-yn-2-ol (Iq):** Colorless oil. The reaction scale is 700 mg (2.55 mmol) 1,3-bis(4-chlorophenyl)prop-2-yn-1-one, isolated amount is 750 mg, 71% yield. ^1^Н-NMR (CDCl_3_, 400 MHz) δ, ppm: 0.23 s (9H, Si(CH_3_)_3_), 7.37 d (2H_arom._, ^3^*J* 8.3 Hz), 7.39 d (2H_arom._, ^3^*J* 8.3 Hz), 7.46 d (2H, ^3^*J* 8.5 Hz), 7.69 d (2H, ^3^*J* 8.5 Hz). ^13^C-NMR (CDCl_3_, 100 MHz) δ, ppm: 1.4 (Si(CH_3_)_3_), 74.5 q (C^2^, *J*_C-F_ 32.7 Hz), 85.7 (C^4^), 88.2 (C^3^), 119.6, 123.2 q (C^1^, *J*_C-F_ 286.1 Hz), 128.5, 129.0, 129.2, 133.2, 135.6, 136.0, 136.1. ^19^F MNR (CDCl_3_, 376 MHz) δ, ppm: −80.69 s (CF_3_). HRMS (ESI): С_19_H_17_Cl_2_F_3_OSiAg found 522.9402 [M + Ag]^+^; calcd. 522.9424.

**Trimethylsilyl ether of 2,4-bis(4-bromophenyl)-1,1,1-trifluorobut-3-yn-2-ol (Ir):** Colorless solid, mp 63°С. The reaction scale is 235 mg (0.65 mmol) 1,3-bis(4-bromophenyl)prop-2-yn-1-one, isolated amount is 271 mg, 83% yield. ^1^Н-NMR (CDCl_3_, 400 MHz) δ, ppm: 0.22 s (9H, Si(CH_3_)_3_), 7.38 d (2H_arom._, ^3^*J* 8.4 Hz), 7.53 d (2H_arom._, ^3^*J* 8.4 Hz), 7.54 d (2H_arom._, ^3^*J* 8.5 Hz), 7.62 d (2H_arom._, ^3^*J* 8.5 Hz). ^13^C-NMR (CDCl_3_, 100 MHz) δ, ppm: 1.4 (Si(CH_3_)_3_), 74.6 q (C^2^, *J*_C-F_ 32.9 Hz), 85.8 (C^4^), 88.3 (C^3^), 120.1, 123.1 q (C^1^, *J*_C-F_ 286.1 Hz), 123.9, 124.3, 129.3 q (*J*_C-F_ 0.4 Hz), 131.4, 132.1, 133.4, 136.6. ^19^F MNR (CDCl_3_, 376 MHz) δ, ppm: −80.68 s (CF_3_). HRMS (ESI): С_19_H_17_Br_2_F_3_OSiAg found 610.8390 [M + Ag]^+^; calcd. 610.8413.

**2,4-Diphenyl-1,1,1-trifluorobut-3-yn-2-ol (1a):**^8^ Yellow oil. The reaction scale is 700 mg (2 mmol) trimethylsilyl ether of 2,4-diphenyl-1,1,1-trifluorobut-3-yn-2-ol, isolated amount is 541 mg, 98% yield. ^1^Н-NMR (CDCl_3_, 400 MHz) δ, ppm: 3.11 s (1H, OH), 7.35–7.47 m (6H_Ph_), 7.54-7.56 m (2H_Ph_), 7.83-7.85 m (2H_Ph_). ^13^C-NMR (CDCl_3_, 100 MHz) δ, ppm: 73.5 (C^2^, *J*_C-F_ 32.4 Hz), 84.6 (C^4^), 88.2 (C^3^), 121.1, 123.6 q (C^1^, *J*_C-F_ 285.6 Hz), 127.3, 128.4, 128.6, 129.66, 129.69, 132.2, 135.5. ^19^F-NMR (CDCl_3_, 376 MHz) δ, ppm: −80.24 s (CF_3_).

**2-(3-Methylphenyl)-1,1,1-trifluoro-4-phenylbut-3-yn-2-ol (1b):**^9^ Yellow oil. The reaction scale is 565 mg (1.6 mmol) trimethylsilyl ether of 2-(3-methylphenyl)-1,1,1-trifluoro-4-phenyl-but-3-yn-2-ol, isolated amount is 303 mg, 67% yield. ^1^Н-NMR (CDCl_3_, 400 MHz) δ, ppm: 2.42 s (3H, CH_3_), 3.10 s (1H, OH), 7.25 d (1H_arom._, ^3^*J* 7.9 Hz), 7.31-7.41 m (4H_arom._), 7.53-7.56 m (2H_arom._), 7.62-7.63 m (1H_arom_), 7.63 s (1H_arom._). ^13^C-NMR (CDCl_3_, 100 MHz) δ, ppm: 21.7 (CH_3_), 73.5 q (C^2^, *J*_C-F_ 32.6 Hz), 84.7 (C^4^), 88.1 (C^3^), 121.2, 123.6 q (C^1^, *J*_C-F_ 285.6 Hz), 124.5 q (*J*_C-F_ 0.8 Hz), 127.8 q (*J*_C-F_ 0.6 Hz), 128.3, 128.6, 129.7, 130.4, 132.2, 135.4, 138.2. ^19^F-NMR (CDCl_3_, 376 MHz) δ, ppm: −80.18 s (CF_3_).

**2-(4-Methylphenyl)-1,1,1-trifluoro-4-phenylbut-3-yn-2-ol (1c)**:^9^ Yellow oil. The reaction scale is 346 mg (0.96 mmol) trimethylsilyl ether of 2-(4-methylphenyl)-1,1,1-trifluoro-4-phenyl-but-3-yn-2-ol, isolated amount is 268 mg, 96% yield. ^1^Н-NMR (CDCl_3_, 400 MHz) δ, ppm: 2.40 (3H, CH_3_), 3.09 s (1H, OH), 7.25 d (2H_arom._, ^3^*J* 8.0 Hz), 7.34–7.43 m (3H_arom_), 7.54 dd (2H_arom._, ^3^*J* 8.0 Hz, ^3^*J* 1.5 Hz), 7.71 d (2H_arom._, ^3^*J* 8.0 Hz). ^13^C-NMR (CDCl_3_, 100 MHz) δ, ppm: 21.3 (CH_3_), 73.5 q (C^2^, *J*_C-F_ 32.5 Hz), 84.8 (C^4^), 88.1 (C^3^), 121.2, 123.6 q (C^1^, *J*_C-F_ 285.5 Hz), 127.2, 128.6, 129.1, 129.6, 132.2, 132.6, 139.7. ^19^F-NMR (CDCl_3_, 376 MHz) δ, ppm: −80.33 s (CF_3_).

**2-(3,4-Dimethylphenyl)-1,1,1-trifluoro-4-phenylbut-3-yn-2-ol (1d):** Pale yellow solid, mp 81°С. The reaction scale is 458 mg (1.22 mmol) trimethylsilyl ether of 2-(3,4-dimethylphenyl)-1,1,1-trifluoro-4-phenyl-but-3-yn-2-ol, isolated amount is 282 mg, 76% yield. ^1^Н-NMR (CDCl_3_, 400 MHz) δ, ppm: 2.30 s (3H, CH_3_), 2.32 s (3H, CH_3_), 3.01 s (1H, OH), 7.20 d (1H_arom._, ^3^*J* 7.9 Hz), 7.34–7.43 m (3H_arom_), 7.52–7.56 m (4H_arom._). ^13^C-NMR (CDCl_3_, 100 MHz) δ, ppm: 19.7 (CH_3_), 20.1 (CH_3_), 73.4 q (C^2^, *J*_C-F_ 32.4 Hz), 84.9 (C^4^), 88.0 (C^3^), 121.3, 123.6 q (C^1^, *J*_C-F_ 285.6 Hz), 124.8 q (*J*_C-F_ 0.6 Hz), 128.3, 128.6, 129.6, 129.7, 132.2, 132.9, 136.7, 138.3. ^19^F-NMR (CDCl_3_, 376 MHz) δ, ppm: −80.23 s (CF_3_). HRMS (ESI): С_18_H_15_F_3_OAg found 411.0118 [M + Ag]^+^; calcd. 411.0121.

**2-(4-Chlorophenyl)-1,1,1-trifluoro-4-phenylbut-3-yn-2-ol (1e):**^10^ Pale yellow oil. The reaction scale is 465 mg (1.22 mmol) trimethylsilyl ether of 2-(4-chlorophenyl)-1,1,1-trifluoro-4-phenyl-but-3-yn-2-ol, isolated amount is 350 mg, 93% yield. ^1^Н-NMR (CDCl_3_, 400 MHz) δ, ppm: 2.85 s (1H, OH), 7.35–7.44 m (5H_arom._), 7.52–7.55 m (2H_arom._), 7.75 d (2H_arom._, ^3^*J* 8.4 Hz). ^13^C-NMR (CDCl_3_, 100 MHz) δ, ppm: 73.1 q (C^2^, *J*_C-F_ 32.7 Hz), 84.1 (C^4^), 88.6 (C^3^), 120.8, 123.4 q (C^1^, *J*_C-F_ 285.8 Hz), 128.6, 128.7, 128.8 d (*J*_C-F_ 0.8 Hz), 129.9, 132.2, 134.0, 135.9. ^19^F-NMR (CDCl_3_, 376 MHz) δ, ppm: −80.39 s (CF_3_).

**2-(4-Bromophenyl)-1,1,1-trifluoro-4-phenylbut-3-yn-2-ol (1f):**^10^ Yellow oil. The reaction scale is 648 mg (1.52 mmol) trimethylsilyl ether of 2-(4-bromophenyl)-1,1,1-trifluoro-4-phenyl-but-3-yn-2-ol, isolated amount is 498 mg, 92% yield. ^1^Н-NMR (CDCl_3_, 400 MHz) δ, ppm: 3.15 s (1H, OH), 7.35–7.42 m (3H_arom_), 7.52–7.55 m (2H_arom._), 7.57 d (2H_arom._, ^3^*J* 8.6 Hz), 7.69 d (2H_arom._, ^3^*J* 8.6 Hz). ^13^C-NMR (CDCl_3_, 100 MHz) δ, ppm: 73.2 q (C^2^, *J*_C-F_ 32.6 Hz), 84.0 (C^4^), 88.6 (C^3^), 120.8, 123.3 q (C^1^, *J*_C-F_ 285.7 Hz), 124.2, 128.7, 129.1, 129.9, 131.6, 132.2, 134.6. ^19^F-NMR (CDCl_3_, 376 MHz) δ, ppm: −80.37 s (CF_3_).

**2-(2-Bromophenyl)-1,1,1-trifluoro-4-phenylbut-3-yn-2-ol (1g):** Yellow oil. The reaction scale is 765 mg (1.8 mmol) trimethylsilyl ether of 2-(2-bromophenyl)-1,1,1-trifluoro-4-phenyl-but-3-yn-2-ol, isolated amount is 608 mg, 95% yield. ^1^Н-NMR (CDCl_3_, 400 MHz) δ, ppm: 3.79 s (1H, OH), 7.26 dt (1H_arom._, ^3^*J* 8.0 Hz, ^4^*J* 1.7 Hz), 7.33–7.43 m (4H_arom_), 7.53–7.56 m (2H_arom._), 7.70 dd (1H_arom._, ^3^*J* 8.0 Hz, ^4^*J* 1.3 Hz), 7.96 d (1H_arom._, ^3^*J* 8.0 Hz). ^13^C-NMR (CDCl_3_, 100 MHz) δ, ppm: 73.9 q (C^2^, *J*_C-F_ 33.1 Hz), 83.7 (C^4^), 89.6 (C^3^), 121.3, 121.7, 123.6 q (C^1^, *J*_C-F_ 287.0 Hz), 127.6, 128.6, 129.7, 130.4 d (*J*_C-F_ 1.5 Hz), 130.9, 132.1, 133.7, 135.8. ^19^F-NMR (CDCl_3_, 376 MHz) δ, ppm: -77.56 s (CF_3_). HRMS (ESI): С_16_H_10_BrF_3_OAg found 460.8933 [M + Ag]^+^; calcd. 460.8913.

**2-(4-Nitrophenyl)-1,1,1-trifluoro-4-phenylbut-3-yn-2-ol (1h):** Yellow solid, mp 115°С. The reaction scale is 386 mg (1 mmol) trimethylsilyl ether of 2-(4-nitrophenyl)-1,1,1-trifluoro-4-phenyl-but-3-yn-2-ol, isolated amount is 202 mg, 63% yield. ^1^Н-NMR (CDCl_3_, 400 MHz) δ, ppm: 3.34 s (1H, OH), 7.36–7.45 m (3H_arom._), 7.52–7.55 m (2H_arom._), 8.01 d (2H_arom._, ^3^*J* 8.8 Hz), 8.29 d (2H_arom._, ^3^*J* 8.8 Hz). ^13^C-NMR (CDCl_3_, 100 MHz) δ, ppm: 73.0 q (C^2^, *J*_C-F_ 32.8 Hz), 83.3 (C^4^), 89.3 (C^3^), 120.4, 123.1 q (C^1^, *J*_C-F_ 286.1 Hz), 123.5, 128.7, 128.8, 130.2, 132.3, 142.1, 148.9. ^19^F-NMR (CDCl_3_, 376 MHz) δ, ppm: −80.07 s (CF_3_). HRMS (ESI): С_16_H_10_F_3_NO_3_Ag found 427.9645 [M + Ag]^+^; calcd. 427.9659.

**4-(4-Methylphenyl)-1,1,1-trifluoro-2-phenylbut-3-yn-2-ol (1i):**^10^ Yellow oil. The reaction scale is 257 mg (0.7 mmol) trimethylsilyl ether of 4-(4-methylphenyl)-1,1,1-trifluoro-2-phenyl-but-3-yn-2-ol, isolated amount is 162 mg, 79% yield. ^1^Н-NMR (CDCl_3_, 400 MHz) δ, ppm: 2.38 s (3H, CH_3_), 3.06 s (1H, OH), 7.17 d (2H_arom._, ^3^*J* 7.9 Hz), 7.42–7.46 m (5H_arom._), 7.81–7.7.84 m (2H_arom._). ^13^C-NMR (CDCl_3_, 100 MHz) δ, ppm: 21.7 (CH_3_), 73.5 q (C^2^, *J*_C-F_ 32.4 Hz), 84.0 (C^4^), 88.5 (C^3^), 118.0, 123.6 q (C^1^, *J*_C-F_ 285.4 Hz), 127.4, 128.4, 129.4, 129.6, 132.1, 135.6, 140.0. ^19^F-NMR (CDCl_3_, 376 MHz) δ, ppm: −80.27 s (CF_3_).

**4-(2,4-Dimethylphenyl)-1,1,1-trifluoro-2-phenylbut-3-yn-2-ol (1j):** Yellow oil. The reaction scale is 287 mg (0.8 mmol) trimethylsilyl ether of 4-(2,4-dimethylphenyl)-1,1,1-trifluoro-2-phenyl-but-3-yn-2-ol, isolated amount is 187 mg, 81% yield. ^1^Н-NMR (CDCl_3_, 400 MHz) δ, ppm: 2.34 s (3H, CH_3_), 2.45 s (3H, CH_3_), 3.10 s (1H, OH), 7.00 d (1H_arom._, ^3^*J* 7.8 Hz), 7.07 s (1H_arom._), 7.38 d (1H_arom._, ^3^*J* 7.8 Hz), 7.41–7.46 m (3H_arom._), 7.82–7.85 m (2H_arom._). ^13^C-NMR (CDCl_3_, 100 MHz) δ, ppm: 20.6 (CH_3_), 21.6 (CH_3_), 73.7 q (C^2^, *J*_C-F_ 32.6 Hz), 87.6 (C^4^), 87.8 (C^3^), 117.9, 123.7 q (C^1^, *J*_C-F_ 285.8 Hz), 126.6, 127.4 d (*J*_C-F_ 0.9 Hz), 128.4, 129.6, 130.7, 132.4, 135.6, 140.0, 141.1. ^19^F-NMR (CDCl_3_, 376 MHz) δ, ppm: −80.28 s (CF_3_). HRMS (ESI): С_18_H_15_F_3_OAg found 411.0123 [M + Ag]^+^; calcd. 411.0121.

**4-(3,4-Dimethylphenyl)-1,1,1-trifluoro-2-phenylbut-3-yn-2-ol (1k):** Yellow oil. The reaction scale is 371 mg (1.0 mmol) trimethylsilyl ether of 4-(3,4-dimethylphenyl)-1,1,1-trifluoro-2-phenyl-but-3-yn-2-ol, isolated amount is 222 mg, 73% yield. ^1^Н-NMR (CDCl_3_, 400 MHz) δ, ppm: 2.26 (3H, CH_3_), 2.29 (3H, CH_3_), 3.10 s (1H, OH), 7.12 d (1H_arom._, ^3^*J* 7.8 Hz), 7.28 dd (1H_arom._, ^3^*J* 7.8 Hz, ^4^*J* 1.2 Hz), 7.32 s (1H_arom._), 7.42–7.46 m (3H_arom._), 7.81–7.83 m (2H_arom._). ^13^C-NMR (CDCl_3_, 100 MHz) δ, ppm: 19.7 (CH_3_), 20.0 (CH_3_), 73.5 q (C^2^, *J*_C-F_ 32.5 Hz), 83.7 (C^4^), 88.7 (C^3^), 118.3, 123.6 q (C^1^, *J*_C-F_ 285.4 Hz), 127.4 q (*J*_C-F_ 0.8 Гц), 128.4, 129.6, 129.7, 129.9, 133.2, 135.6, 137.1, 138.8. ^19^F-NMR (CDCl_3_, 376 MHz) δ, ppm: −80.27 s (CF_3_). HRMS (ESI): С_18_H_15_F_3_OAg found 411.0112 [M + Ag]^+^; calcd. 411.0121.

**4-(4-Chlorophenyl)-1,1,1-trifluoro-2-phenylbut-3-yn-2-ol (1l):**^11^ Pale yellow oil. The reaction scale is 371 mg (0.96 mmol) trimethylsilyl ether of 4-(4-chlorophenyl)-1,1,1-trifluoro-2-phenyl-but-3-yn-2-ol, isolated amount is 232 mg, 78% yield. ^1^Н-NMR (CDCl_3_, 400 MHz) δ, ppm: 3.09 s (1H, OH), 7.35 d (2H_arom._, ^3^*J* 8.6 Hz), 7.43–7.48 m (5H_arom_), 7.79–7.81 m (2H_arom._). ^13^C-NMR (CDCl_3_, 100 MHz) δ, ppm: 73.6 q (C^2^, *J*_C-F_ 32.5 Hz), 85.6 (C^4^), 87.1 (C^3^), 119.6, 123.5 q (C^1^, *J*_C-F_ 285.6 Hz), 127.3 d (*J*_C-F_ 0.7 Hz), 128.5, 129.0, 129.8, 133.4, 135.2, 134.9. ^19^F-NMR (CDCl_3_, 376 MHz) δ, ppm: −80.20 s (CF_3_).

**4-(4-Bromophenyl)-1,1,1-trifluoro-2-phenylbut-3-yn-2-ol (1m):**^11^ Yellow oil. The reaction scale is 189 mg (0.44 mmol) trimethylsilyl ether of 4-(4-bromophenyl)-1,1,1-trifluoro-2-phenyl-but-3-yn-2-ol, isolated amount is 144 mg, 92% yield. ^1^Н-NMR (CDCl_3_, 400 MHz) δ, ppm: 3.00 s (1H, OH), 7.39 d (2H_arom._, ^3^*J* 8.6 Hz), 7.43–7.46 m (3H_arom._), 7.51 d (2H_arom._, ^3^*J* 8.6 Hz), 7.78–7.81 m (2H_arom._). ^13^C-NMR (CDCl_3_, 100 MHz) δ, ppm: 73.5 q (C^2^, *J*_C-F_ 32.6 Hz), 85.7 (C^4^), 87.1 (C^3^), 120.0, 123.4 q (C^1^, *J*_C-F_ 285.6 Hz), 124.2, 127.3 q (*J*_C-F_ 0.8 Hz), 128.5, 129.8, 132.0, 133.6, 135.2. ^19^F-NMR (CDCl_3_, 376 MHz) δ, ppm: −80.20 s (CF_3_).

**4-(4-Nitrophenyl)-1,1,1-trifluoro-2-phenylbut-3-yn-2-ol (1n):** Colorless solid, mp 102°С. The reaction scale is 164 mg (0.42 mmol) trimethylsilyl ether of 4-(4-nitrophenyl)-1,1,1-trifluoro-2-phenyl-but-3-yn-2-ol, isolated amount is 131 mg, 97% yield. ^1^Н-NMR (CDCl_3_, 400 MHz) δ, ppm: 3.21 s (1H, OH), 7.44–7.48 m (3H_arom._), 7.70 d (2H_arom._, ^3^*J* 8.9 Hz), 7.78–7.80 m (2H_arom._), 8.24 d (2H_arom._, ^3^*J* 8.9 Hz). ^13^C-NMR (CDCl_3_, 100 MHz) δ, ppm: 73.6 q (C^2^, *J*_C-F_ 32.7 Hz), 85.9 (C^4^), 89.3 (C^3^), 123.3 q (C^1^, *J*_C-F_ 286.8 Hz), 123.8, 127.1 q (*J*_C-F_ 0.7 Hz), 127.8, 128.6, 130.0, 133.1, 134.7, 148.1. ^19^F-NMR (CDCl_3_, 376 MHz) δ, ppm: −80.05 s (CF_3_). HRMS (ESI): С_16_H_10_F_3_NO_3_Ag found 427.9646 [M + Ag]^+^; calcd. 427.9659.

**2,4-Bis(4-methylphenyl)-1,1,1-trifluorobut-3-yn-2-ol (1o):**^12^ Yellow oil. The reaction scale is 200 mg (0.5 mmol) trimethylsilyl ether of 2,4-bis(4-methylphenyl)-1,1,1-trifluorobut-3-yn-2-ol, isolated amount is 149 mg, 92% yield. ^1^Н-NMR (CDCl_3_, 400 MHz) δ, ppm: 2.38 s (3H, CH_3_), 2.39 s (3H, CH_3_), 3.08 s (1H, OH), 7.17 d (2H_arom._, ^3^*J* 8.0 Hz), 7.24 d (2H_arom._, ^3^*J* 8.0 Hz), 7.42 d (2H_arom._, ^3^*J* 8.0 Hz), 7.70 d (2H_arom._, ^3^*J* 8.0 Hz). ^13^C-NMR (CDCl_3_, 100 MHz) δ, ppm: 21.3 (CH_3_), 21.7 (CH_3_), 73.5 q (C^2^, *J*_C-F_ 32.4 Hz), 84.1 (C^4^), 88.3 (C^3^), 118.1, 123.6 q (C^1^, *J*_C-F_ 285.4 Hz), 127.2, 128.4, 129.1, 129.3, 132.1, 132.7, 139.6, 140.0. ^19^F-NMR (CDCl_3_, 376 MHz) δ, ppm: −80.35 s (CF_3_).

**2,4-Bis(3,4-dimethylphenyl)-1,1,1-trifluorobut-3-yn-2-ol (1p):** Yellow oil. The reaction scale is 724 mg (1.8 mmol) trimethylsilyl ether of 2,4-bis(3,4-dimethylphenyl)-1,1,1-trifluorobut-3-yn-2-ol, isolated amount is 390 mg, 68% yield. ^1^Н-NMR (CDCl_3_, 400 MHz) δ, ppm: 2.26 s (3H, CH_3_), 2.29 s (3H, CH_3_), 2.30 s (3H, CH_3_), 2.33 s (3H, CH_3_), 3.08 s (1H, OH), 7.12 d (1H_arom._, ^3^*J* 7.8 Hz), 7.20 d (1H_arom._, ^3^*J* 7.8 Hz), 7.28 d (1H_arom._, ^3^*J* 8.1 Hz), 7.32 s (1H_arom_), 7.55 d (1H_arom._, ^3^*J* 8.1 Hz), 7.58 s (1H_arom._). ^13^C-NMR (CDCl_3_, 100 MHz) δ, ppm: 19.6 (CH_3_), 19.7 (CH_3_), 19.9 (CH_3_), 20.1 (CH_3_), 73.4 q (C^2^, *J*_C-F_ 32.4 Hz), 84.0 (C^4^), 88.4 (C^3^), 118.5, 123.7 q (C^1^, *J*_C-F_ 285.5 Hz), 124.8, 128.3, 129.6, 129.6, 129.9, 133.1, 133.2, 136.7, 137.0, 138.2, 138.7. ^19^F-NMR (CDCl_3_, 376 MHz) δ, ppm: −80.25 s (CF_3_). HRMS (ESI): С_20_H_19_F_3_OAg found 439.0430 [M + Ag]^+^; calcd. 439.0434.

**2,4-Bis(4-Chlorophenyl)-1,1,1-trifluorobut-3-yn-2-ol (1q):**^12^ Pale yellow oil. The reaction scale is 720 mg (1.74 mmol) trimethylsilyl ether of 2,4-bis(4-chlorophenyl)-1,1,1-trifluorobut-3-yn-2-ol, isolated amount is 592 mg, 99% yield. ^1^Н-NMR (CDCl_3_, 400 MHz) δ, ppm: 3.12 s (1H, OH), 7.35 d (2H_arom._, ^3^*J* 8.7 Hz), 7.41 d (2H_arom._, ^3^*J* 8.7 Hz), 7.45 d (2H_arom._, ^3^*J* 8.7 Hz), 7.73 d (2H_arom._, ^3^*J* 8.7 Hz). ^13^C-NMR (CDCl_3_, 100 MHz) δ, ppm: 73.1 q (C^2^, *J*_C-F_ 32.7 Hz), 85.0 (C^4^), 87.4 (C^3^), 119.3, 123.3 q (C^1^, *J*_C-F_ 285.8 Hz), 128.7, 128.8 q (*J*_C-F_ 0.6 Hz), 129.1, 133.4, 133.8, 136.0, 136.2. ^19^F-NMR (CDCl_3_, 376 MHz) δ, ppm: −80.34 s (CF_3_).

**2,4-Bis(4-bromophenyl)-1,1,1-trifluorobut-3-yn-2-ol (1r):** Pale yellow oil. The reaction scale is 138 mg (0.28 mmol) trimethylsilyl ether of 2,4-bis(4-bromophenyl)-1,1,1-trifluorobut-3-yn-2-ol, isolated amount is 68 mg, 58% yield. ^1^Н-NMR (CDCl_3_, 400 MHz) δ, ppm: 3.15 s (1H, OH), 7.38 d (2H_arom._, ^3^*J* 8.5 Hz), 7.51 d (2H_arom._, ^3^*J* 8.5 Hz), 7.57 d (2H_arom._, ^3^*J* 8.6 Hz), 7.66 d (2H_arom._, ^3^*J* 8.6 Hz). ^13^C-NMR (CDCl_3_, 100 MHz) δ, ppm: 73.2 q (C^2^, *J*_C-F_ 32.6 Hz), 85.1 (C^4^), 87.5 (C^3^), 119.7, 123.2 q (C^1^, *J*_C-F_ 285.8 Hz), 124.3, 124.4, 129.0 q (*J*_C-F_ 0.5 Hz), 131.7, 132.0, 133.6, 134.3. ^19^F-NMR (CDCl_3_, 376 MHz) δ, ppm: −80.32 s (CF_3_). HRMS (ESI): С_16_H_9_Br_2_F_3_OAg found 538.8039 [M + Ag]^+^; calcd. 538.8018.

**General procedure for reaction of compounds 1a–r with arenes in superacid TfOH. Synthesis of compounds 2-5.**

CF_3_-alcohol **1** (0.1 mmol) was added to mixture of TfOH (0.15 mmol or 0.25 mmol) with benzene (5 mmol, 0.4 mL) or another arene (0.11 mmol) in CH_2_Cl_2_ (1 mL) at 20 °C. Reaction mixture was magnetically stirred for 1 h. Then the mixture was poured into ice water (30 mL) and extracted with chloroform (2 × 40 mL). The extracts were combined, washed with water, a saturated aqueous solution of NaHCO_3_, water again, and dried over Na_2_SO_4_. The solvent was distilled off under reduced pressure. The crude mixture was purified by preparative TLC on silica gel, using petroleum ether or petroleum ether-EtOAc mixtures (9:1) as an eluent.

**1,1,1-Trifluoro-4-(2,5-dimethylphenyl)-4-(4-nitrophenyl)-buta-2,3-diene (2a):** Colorless oil. The reaction scale is 30 mg (0.1 mmol) alcohol **1n** and 12 mg (0.11 mmol) *p*-xylene,isolated amount is 22 mg **2a** (yield 71%). ^1^Н-NMR (CDCl_3_, 400 MHz) δ, ppm: 2.14 s (3H, CH_3_), 2.37 s (3H, CH_3_), 7.12 s (1H_arom_), 7.18 dd (1H_arom._, ^3^*J* 7.9 Hz, ^4^*J* 1.3 Hz), 7.21 d (1H_arom._, ^3^*J* 7.9 Hz), 7.35-7.44 m (3H_arom._), 7.40 d (2H_arom._, ^3^*J* 8.9 Hz), 7.51 d (2H_arom._, ^3^*J* 7.7 Hz), 8.19 d (2H_arom._, ^3^*J* 8.9 Hz). ^13^C-NMR (CDCl_3_, 100 MHz) δ, ppm: 19.6 (CH_3_), 21.1 (CH_3_), 105.1 q (C^2^, *J*_C-F_ 34.6 Hz), 115.3, 123.3 q (C^1^, *J*_C-F_ 275.4 Hz), 124.3, 127.4 q (*J*_C-F_ 1.1 Hz), 127.9, 129.0, 129.1, 129.2, 130.2, 130.6, 131.0, 132.3, 133.7, 136.4, 141.1, 147.7, 206.5 q (C^3^, *J*_C-F_ 3.9 Hz). ^19^F-NMR (CDCl_3_, 376 MHz) δ, ppm: −59.65 s (CF_3_). HRМS (МALDI): C_24_H_19_F_3_NO_2_ найдено 410.1374 [М + H]^+^; вычислено 410.1365.

**1,3-Diphenyl-1-(trifluoromethyl)-*1****H***-indene (4aa):** Pale yellow solid, mp 95°С (МеОН). The reaction scale is 30 mg (0.1 mmol) alcohol **1a** and 0.4 mL benzene, isolated amount is 17 mg **4aa** (yield 51%). ^1^Н-NMR (CDCl_3_, 400 MHz) δ, ppm: 6.68 s (1H, C^2^H), 7.32-7.38 m (4H_arom._), 7.41-7.50 m (4H_arom._), 7.57-7.59 m (3H_arom._), 7.62-7.64 m (2H_arom._), 7.68 d (1H_arom._, ^3^*J* 7.4 Hz). ^13^C-NMR (CDCl_3_, 100 MHz) δ, ppm: 64.3 q (C^1^, *J*_C-F_ 27.0 Hz), 121.8 (C^2^), 125.9, 126.7, 126.8 q (CF_3_, *J*_C-F_ 282.5 Hz), 127.9, 128.0, 128.2, 128.7, 128.8, 128.9, 132.2 q (*J*_C-F_ 1.7 Hz), 134.4, 134.6, 143.7 (C^3^), 144.0 d (*J*_C-F_ 0.6 Hz), 147.4. ^19^F-NMR (CDCl_3_, 376 MHz) δ, ppm: –67.45 s (CF_3_). HRMS (MALDI-TOF): C_22_H_16_F_3_ found 337.1208 [M + H]^+^; calcd. 337.1199.

**1-(3-Methylphenyl)-3-phenyl-1-(trifluoromethyl)-1H-indene (4ab):** Pale yellow oil. The reaction scale is 30 mg (0.1 mmol) alcohol **1b** and 0.4 mL benzene, isolated amount is 18 mg **4ab** (yield 48%). ^1^Н-NMR (CDCl_3_, 400 MHz) δ, ppm: 2.34 s (CH_3_), 6.67 s (1H, C^2^H), 7.13 d (1H_arom._, ^3^*J* 7.4 Hz), 7.21-7.25 m (1H_arom._), 7.34-7.40 m (3H_arom._), 7.41-7.50 m (4H_arom._), 7.57 d (1H_arom._, ^3^*J* 7.4 Hz), 7.62-7.65 m (2H_arom._), 7.69 d (1H_arom._, ^3^*J* 7.5 Hz). ^13^C-NMR (CDCl_3_, 100 MHz) δ, ppm: 21.8 (CH_3_), 64.2 q (C^1^, *J*_C-F_ 26.8 Hz), 121.8, 125.1 q (*J*_C-F_ 1.8 Hz), 125.9 q (*J*_C-F_ 0.7 Hz), 126.7, 126.8 q (CF_3_, *J*_C-F_ 282.6 Hz), 127.9, 128.6, 128.69, 128.7 q (*J*_C-F_ 1.9 Hz), 128.73, 128.9 (2С), 129.0, 132.3 q (*J*_C-F_ 1.8 Hz), 134.5, 138.4, 143.6, 144.0 q (*J*_C-F_ 1.1 Hz), 147.3. ^19^F-NMR (CDCl_3_, 376 MHz) δ, ppm: −67.41 s (CF_3_). HRMS (MALDI-TOF): C_23_H_18_F_3_ found 351.1343 [M + H]^+^; calcd. 351.1356.

**1-(4-Methylphenyl)-3-phenyl-1-(trifluoromethyl)-1H-indene (4ac):** Colorless oil. The reaction scale is 30 mg (0.1 mmol) alcohol **1c** and 0.4 mL benzene, isolated amount is 24 mg **4ac** (yield 69%). ^1^Н-NMR (CDCl_3_, 400 MHz) δ, ppm: 2.34 s (3H, CH_3_), 6.68 s (1H, C^2^H), 7.15 d (2H_arom._, ^3^*J* 8.1 Hz), 7.36 td (1H_arom._, ^3^*J* 7.4 Hz, ^4^*J* 1.1 Hz), 7.41-7.50 m (6H_arom._), 7.57 d (1H_arom._, ^3^*J* 7.4 Hz), 7.61-7.64 m (2H_arom._), 7.69 d (1H_arom._, ^3^*J* 7.4 Hz). ^13^C-NMR (CDCl_3_, 100 MHz) δ, ppm: 21.1 (CH_3_), 64.0 q (C^1^, *J*_C-F_ 27.0 Hz), 121.8, 125.8 q (*J*_C-F_ 0.6 Hz), 126.6, 126.8 q (CF_3_, *J*_C-F_ 282.5 Hz), 127.90 q (*J*_C-F_ 1.7 Hz), 127.94, 128.66, 128.71, 128.8, 129.5, 131.6, 132.3 q (*J*_C-F_ 1.8 Hz), 134.5, 138.0, 143.6, 144.0 q (*J*_C-F_ 1.0 Hz), 147.3. ^19^F-NMR (CDCl_3_, 376 MHz) δ, ppm: −67.66 s (CF_3_). HRMS (MALDI-TOF): C_23_H_18_F_3_ found 351.1361 [M + H]^+^; calcd. 351.1356.

**1-(3,4-Dimethylphenyl)-3-phenyl-1-(trifluoromethyl)-*1H*-indene (4ad):** The reaction scale is 30 mg (0.1 mmol) alcohol **1d** and 0.4 mL benzene, isolated amount is 17 mg mixture of **4ad**, **6a** and **6b** (total yield 47%, ratio **4ad**:**6a**:**6b** = 5:2:1, respectively). ^1^Н-NMR (CDCl_3_, 400 MHz, measured in a mixture with **6a**) δ, ppm: 2.236 s (3H, CH_3_), 2.244 s (3H, CH_3_), 6.67 s (1H, C^2^H), 7.09 d (1H_arom._, ^3^*J* 7.7 Hz), 7.30-7.34 m (2H_arom._), 7.35 td (1H_arom._, ^3^*J* 7.5 Hz, ^4^*J* 1.0 Hz), 7.40-7.49 m (4H_arom._), 7.56 d (1H_arom._, ^3^*J* 7.5 Hz), 7.60-7.63 m (2H_arom._), 7.69 d (1H_arom._, ^3^*J* 7.5 Hz). ^13^C-NMR (CDCl_3_, 100 MHz) δ, ppm: 19.5 (CH_3_), 20.2 (CH_3_), 63.9 q (C^1^, *J*_C-F_ 26.8 Hz), 121.7, 125.5 q (*J*_C-F_ 1.7 Hz), 125.9 q (*J*_C-F_ 0.8 Hz), 126.6, 126.8 q (CF_3_, *J*_C-F_ 282.5 Hz), 127.9, 128.6, 128.7, 128.8, 129.2 q (*J*_C-F_ 1.7 Hz), 130.0, 132.3 q (*J*_C-F_ 1.8 Hz), 134.5, 136.8, 137.0, 143.6, 143.97 q (*J*_C-F_ 1.2 Hz), 144.0 q (*J*_C-F_ 1.2 Hz), 147.1. ^19^F-NMR (CDCl_3_, 376 MHz, measured in a mixture with **6a**) δ, ppm: −67.62 s (CF_3_). HRMS (MALDI-TOF): C_24_H_20_F_3_ found 365.1519 [M + H]^+^; calcd. 365.1512.

**1-(4-Chlorophenyl)-3-phenyl-1-(trifluoromethyl)-1***H***-indene (4ae):** Pale yellow oil. The reaction scale is 30 mg (0.1 mmol) alcohol **1e** and 0.4 mL benzene, isolated amount is 27 mg **4ae** (yield 73%). ^1^Н-NMR (CDCl_3_, 400 MHz) δ, ppm: 6.62 s (1H, C^2^H), 7.30 d (2H_arom._, ^3^*J* 8.8 Hz), 7.37 td (1H_arom._, ^3^*J* 7.5 Hz, ^4^*J* 1.0 Hz), 7.42-7.51 m (6H_arom_), 7.58 d (1H_arom._, ^3^*J* 7.5 Hz), 7.60–7.65 m (3H_arom._). ^13^C-NMR (CDCl_3_, 100 MHz) δ, ppm: 63.8 q (C^1^, *J*_C-F_ 27.0 Hz), 122.0, 125.7, 126.6 q (CF_3_, *J*_C-F_ 282.4 Hz), 126.9, 127.9, 128.87, 128.92, 128.95, 129.0, 129.4 q (*J*_C-F_ 1.8 Hz), 131.6 q (*J*_C-F_ 1.6 Hz), 133.2, 134.2, 134.3, 143.56, 143.63, 147.8. ^19^F-NMR (CDCl_3_, 376 MHz) δ, ppm: −67.56 s (CF_3_). HRMS (MALDI-TOF): C_22_H_15_F_3_Cl found 371.0812 [M + H]^+^; calcd. 371.0809.

**1-(4-Bromophenyl)-3-phenyl-1-(trifluoromethyl)-1H-indene (4af):** Colorless oil. The reaction scale is 40 mg (0.12 mmol) alcohol **1f** and 0.6 mL benzene, isolated amount is 56 mg **4af** (yield 80%). ^1^Н-NMR (CDCl_3_, 400 MHz) δ, ppm: 6.61 s (1H, C^2^H), 7.51 td (1H_arom._, ^3^*J* 7.5 Hz, ^4^*J* 0.9 Hz), 7.41–7.51 m (8H_arom._), 7.58 d (1H_arom._, ^3^*J* 7.5 Hz), 7.60–7.64 m (3H_arom._). ^13^C-NMR (CDCl_3_, 100 MHz) δ, ppm: 63.9 q (C^1^, *J*_C-F_ 26.7 Hz), 122.0, 122.4, 125.7 q (*J*_C-F_ 0.7 Hz), 126.5 q (CF_3_, *J*_C-F_ 282.4 Hz), 126.9, 127.9, 128.87, 128.92, 129.0, 129.7 q (*J*_C-F_ 1.7 Hz), 131.6 q (*J*_C-F_ 1.8 Hz), 131.9, 133.8, 134.2, 143.57, 143.60 q (*J*_C-F_ 1.1 Hz), 147.9. ^19^F-NMR (CDCl_3_, 376 MHz) δ, ppm: –67.51 s (CF_3_). HRMS (MALDI-TOF): C_22_H_15_F_3_Br found 415.0310 [M + H]^+^; calcd. 415.0304.

**1-(2-Bromophenyl)-1-(trifluoromethyl)-3-phenyl-1*H*-indene (4ag):** Pale yellow oil. The reaction scale is 30 mg (0.09 mmol) alcohol **1g** and 0.4 mL benzene, isolated amount is 35 mg **4ae** (yield 66%). ^1^Н-NMR (CDCl_3_, 400 MHz) δ, ppm: 6.42 s (1H, C^2^H), 7.17 td (1H_arom._, ^3^*J* 7.7 Hz, ^4^*J* 1.5 Hz), 7.26-7.31 m (3H_arom._), 7.41-7.46 m (3H_arom._), 7.48-7.53 m (2H_arom._), 7.56 d (1H_arom._, ^3^*J* 7.7 Hz), 7.65–7.67 m (2H_arom._), 7.99−8.02 m (1H_arom._). ^13^C-NMR (CDCl_3_, 100 MHz) δ, ppm: 65.9 q (C^1^, *J*_C-F_ 25.4 Hz), 121.7, 122.8, 125.0, 126.4 q (CF_3_, *J*_C-F_ 284.1 Hz), 126.8, 127.6, 127.8, 128.7, 128.8, 128.9, 129.3, 129.5, 130.7 q (*J*_C-F_ 4.3 Hz), 133.5, 134.7, 136.4, 144.2, 146.1, 151.1. ^19^F-NMR (CDCl_3_, 376 MHz) δ, ppm: −65.17 s (CF_3_). HRMS (MALDI-TOF): C_22_H_15_F_3_Br found 415.0308 [M + H]^+^; calcd. 415.0304.

**1-(4-Nitrophenyl)-3-phenyl-1-(trifluoromethyl)-1*H*-indene (4ah):** Pale yellow oil. The reaction scale is 30 mg (0.1 mmol) alcohol **1h**, 38 mg (0.25 mmol) TfOH and 0.4 mL benzene, isolated amount is 25 mg **4ah** (yield 70%). ^1^Н-NMR (CDCl_3_, 400 MHz) δ, ppm: 6.60 s (1H, C^2^H), 7.39 td (1H_arom._, ^3^*J* 7.5, ^4^*J* 0.9 Hz), 7.43–7.52 m (4H_arom_), 7.58–7.64 m (4H_arom_), 7.69 d (2H_arom._, ^3^*J* 9.0 Hz), 8.17 d (2H_arom._, ^3^*J* 9.0 Hz). ^13^C-NMR (CDCl_3_, 100 MHz) δ, ppm: 64.3 q (C^1^, *J*_C-F_ 27.4 Hz), 122.3, 123.9, 125.6, 126.3 q (CF_3_, *J*_C-F_ 282.6 Hz), 127.3, 127.9, 128.9 q (*J*_C-F_ 1.9 Hz), 129.0, 129.1, 129.4, 130.8 q (*J*_C-F_ 1.6 Hz), 133.8, 142.3, 143.3 q (*J*_C-F_ 0.8 Hz), 143.5, 147.7, 148.8. ^19^F-NMR (CDCl_3_, 376 MHz) δ, ppm: –66.98 s (CF_3_). HRMS (MALDI-TOF): C_22_H_15_F_3_NO_2_ found 382.1049 [M + H]^+^; calcd. 382.1049.

**3-(4-Methylphenyl)-1-phenyl-1-(trifluoromethyl)-*1H*-indene (4ai)** and **1,3-diphenyl–6-methyl-1-(trifluoromethyl)-*1H*-indene (5aa):** The reaction scale is 30 mg (0.1 mmol) alcohol **1i** and 0.4 mL benzene, isolated amount is 25 mg mixture of **4ai** and **5aa** (total yield 72%, ratio **4ai** : **5aa** = 1:3.8, respectively). Compound **4ai**: ^1^Н-NMR (CDCl_3_, 400 MHz, measured in a mixture with **5aa**) δ, ppm: 2.43 s (3H, CH_3_), 6.64 s (1H, C^2^H), 7.29 d (2H_arom._, ^3^*J* 7.9 Hz), 7.31-7.38 m (5H_arom._), 7.40-7.42 m (1H_arom._), 7.53 d (2H_arom._, ^3^*J* 7.9 Hz), 7.56-7.58 m (2H_arom._), 7.68 d (1H_arom._, ^3^*J* 7.4 Hz). ^13^C-NMR (CDCl_3_, 100 MHz, measured in a mixture with **5aa**) δ, ppm: 21.5 (CH_3_), 64.2 q (C^1^, *J*_C-F_ 26.3 Hz), 121.8, 125.8, 126.6, 126.8 q (CF_3_, *J*_C-F_ 282.5 Hz), 127.8, 127.9, 128.0 q (*J*_C-F_ 2.0 Hz), 128.1, 129.46, 129.54, 131.5, 131.6 q (*J*_C-F_ 1.7 Hz), 134.8, 138.6, 143.8, 144.0 q (*J*_C-F_ 1.1 Hz), 147.3. ^19^F-NMR (CDCl_3_, 376 MHz, measured in a mixture with **5aa**) δ, ppm: –67.45 s (CF_3_). HRMS (MALDI-TOF): C_23_H_18_F_3_ found 351.1361 [M + H]^+^; calcd. 351.1356. Compound **5aa:** ^1^Н-NMR (CDCl_3_, 400 MHz, measured in a mixture with **4ai**) δ, ppm: 2.45 s (3H, CH_3_), 6.61 s (1H, C^2^H), 7.24 d (1H_arom._, ^3^*J* 7.9 Hz), 7.31-7.38 m (3H_arom._), 7.40–7.49 m (5H_arom._), 7.56–7.58 m (2H_arom._), 7.62-7.65 m (2H_arom._). ^13^C-NMR (CDCl_3_, 100 MHz, measured in a mixture with **4ai**) δ, ppm: 21.8 (CH_3_), 64.1 q (C^1^, *J*_C-F_ 26.9 Hz), 121.5, 126.7 q (*J*_C-F_ 0.8 Hz), 126.8 q (CF_3_, *J*_C-F_ 282.5 Hz), 127.89, 127.94 q (*J*_C-F_ 1.8 Hz), 128.1, 128.6, 128.76, 128.83, 129.4, 131.3 q (*J*_C-F_ 1.8 Hz), 134.6, 135.0, 136.7, 141.0, 144.3 (*J*_C-F_ 1.0 Hz), 147.3. ^19^F-NMR (CDCl_3_, 376 MHz, measured in a mixture with **4ai**) δ, ppm: –67.33 s (CF_3_). HRMS (MALDI-TOF): C_23_H_18_F_3_ found 351.1361 [M + H]^+^; calcd. 351.1356.

**3-(2,4-Dimethylphenyl)-1-phenyl-1-(trifluoromethyl)-*1H*-indene (4aj)** and **4,6-dimethyl-1,3-diphenyl-1-(trifluoromethyl)-*1H*-indene (5ab):** Isolated from the reaction of alcohol **1j** (30 mg, 0.11 mmol) and benzene (0.4 mL), yield 47% (19 mg, mixture of **4aj** and **5ab** 1:7, respectively) or from the reaction of alcohol **1a** (30 mg, 0.11 mmol) and *m*-xylene (12 mg, 0.12 mmol), yield 63% (25 mg mixture of **4aj** and **5ab** 1:6, respectively). Compound **4aj**: ^1^Н-NMR (CDCl_3_, 400 MHz, measured in a mixture with **5ab**) δ, ppm: 2.25 s (3H, CH_3_), 2.40 s (3H, CH_3_), 6.52 s (1H, C^2^H), 7.08–7.13 m (2H_arom._), 7.15 m (1H_arom._), 7.21 d (1H_arom._, ^3^*J* 7.5 Hz), 7.30–7.43 m (5H_arom._), 7.56–7.61 m (2H_arom._), 7.67 d (1H_arom._, ^3^*J* 7.5 Hz). ^13^C-NMR (CDCl_3_, 100 MHz, measured in a mixture with **5ab**) δ, ppm: 20.1 (CH_3_), 21.3 (CH_3_), 64.6 q (C^1^, *J*_C-F_ 26.9 Hz), 121.9, 125.5 q (*J*_C-F_ 0.6 Hz), 126.5, 126.6, 126.8 q (CF_3_, *J*_C-F_ 284.1 Hz), 127.9, 128.1, 128.8, 129.2, 129.2, 131.0, 131.1, 133.2 q (*J*_C-F_ 1.7 Hz), 134.8, 136.3, 138.1, 143.4 q (*J*_C-F_ 1.1 Hz), 145.1, 147.7. ^19^F-NMR (CDCl_3_, 376 MHz, measured in a mixture with **5ab**) δ, ppm: –67.50 s (CF_3_). HRMS (MALDI-TOF): C_24_H_20_F_3_ found 365.1497 [M + H]^+^; calcd. 365.1512. Compound **5ab**: ^1^Н-NMR (CDCl_3_, 400 MHz, measured in a mixture with **4aj**) δ, ppm: 1.97 s (3H, CH_3_), 2.39 (3H, CH_3_), 6.39 s (1H, C^2^H), 6.95 d (1H, C^5^H, ^4^*J* 0.6 Hz), 7.30 s (1H, C^7^H), 7.32-7.43 m (8H_arom._), 7.57 d (2H_arom._, ^3^*J* 7.5 Hz). ^13^C-NMR (CDCl_3_, 100 MHz, measured in a mixture with **4aj**) δ, ppm: 20.2 (CH_3_), 21.5 (CH_3_), 63.7 q (C^1^, *J*_C-F_ 26.6 Hz), 124.3 q (*J*_C-F_ 0.7 Hz), 126.9 q (CF_3_, *J*_C-F_ 282.5 Hz), 127.9 q (*J*_C-F_ 1.8 Hz), 127.9, 128.0, 128.2, 128.6, 128.7, 132.5, 132.6, 133.2 q (*J*_C-F_ 1.8 Hz), 135.2, 136.6, 137.5, 139.2, 144.5 q (*J*_C-F_ 0.9 Hz), 149.3. ^19^F-NMR (CDCl_3_, 376 MHz, measured in a mixture with **4aj**) δ, ppm: –67.36 s (CF_3_). HRMS (MALDI-TOF): C_24_H_20_F_3_ found 365.1497 [M + H]^+^; calcd. 365.1512.

**3-(4-Chlorophenyl)-1-phenyl-1-(trifluoromethyl)-*1H*-indene (4ak):** Pale yellow oil. The reaction scale is 30 mg (0.1 mmol) alcohol **1l** and 0.4 mL benzene, isolated amount is 22 mg **4ak** (yield 59%). ^1^Н-NMR (CDCl_3_, 400 MHz) δ, ppm: 6.67 s (1H, C^2^H), 7.30–7.37 m (3H_arom_), 7.38 td (1H_arom._, ^3^*J* 7.4 Hz, ^4^*J* 1.1 Hz), 7.42–7.47 m (1H_arom._), 7.45 d (2H_arom._, ^3^*J* 8.5 Hz), 7.51 d (1H_arom._, ^3^*J* 7.4 Hz), 7.55-7.59 m (2H_arom_), 7.56 d (2H_arom._, ^3^*J* 8.5 Hz), 7.69 d (1H_arom._, ^3^*J* 7.4 Hz). ^13^C-NMR (CDCl_3_, 100 MHz) δ, ppm: 64.4 q (C^1^, *J*_C-F_ 26.9 Hz), 121.6, 126.0 q (*J*_C-F_ 0.8 Hz), 126.7 q (CF_3_, *J*_C-F_ 282.6 Hz), 126.9, 128.0 q (*J*_C-F_ 1.6 Hz), 128.3, 128.8, 128.9, 129.1, 129.2, 132.6 q (*J*_C-F_ 1.8 Hz), 132.8, 134.4, 134.7, 143.3, 143.8 q (*J*_C-F_ 0.8 Hz), 146.3. ^19^F-NMR (CDCl_3_, 376 MHz) δ, ppm: −67.46 s (CF_3_). HRMS (MALDI-TOF): C_22_H_15_F_3_Cl found 371.0798 [M + H]^+^; calcd. 371.0809.

**3-(4-Bromophenyl)-1-phenyl-1-(trifluoromethyl)-*1H*-indene (4al):** Colorless oil. The reaction scale is 30 mg (0.09 mmol) alcohol **1m** and 0.4 mL benzene, isolated amount is 19 mg **4al** (yield 51%). ^1^Н-NMR (CDCl_3_, 400 MHz) δ, ppm: 6.67 s (1H, C^2^H), 7.32-7.36 m (3H_arom._), 7.37 td (1H_arom._, ^3^*J* 7.5 Hz, ^4^*J* 1.2 Hz), 7.44 td (1H_arom._, ^3^*J* 7.5 Hz, ^4^*J* 1.2 Hz), 7.49 d (2H_arom._, ^3^*J* 8.5 Hz), 7.50-7.52 m (1H_arom._), 7.54-7.56 m (2H_arom_), 7.61 d (2H_arom._, ^3^*J* 8.5 Hz), 7.68 d (1H_arom._, ^3^*J* 7.5 Hz). ^13^C-NMR (CDCl_3_, 100 MHz) δ, ppm: 64.4 q (C^1^, *J*_C-F_ 26.9 Hz), 121.6, 122.8, 126.0, 126.6 q (CF_3_, *J*_C-F_ 282.4 Hz), 126.9, 128.0 q (*J*_C-F_ 1.6 Hz), 128.3, 128.8, 128.9, 129.5, 132.1, 132.6 q (*J*_C-F_ 1.6 Hz), 133.3, 134.3, 143.2, 143.8, 146.4. ^19^F-NMR (CDCl_3_, 376 MHz) δ, ppm: –67.46 s (CF_3_). HRMS (MALDI-TOF): C_22_H_15_F_3_Br found 415.0298 [M + H]^+^; calcd. 415.0304.

**3-(4-Nitrophenyl)-1-phenyl-1-(trifluoromethyl)-*1H*-indene (4am):** Pale yellow oil. The reaction scale is 30 mg (0.1 mmol) alcohol **1n**, 38 mg (0.25 mmol) TfOH and 0.4 mL benzene, isolated amount is 22 mg **4am** (yield 62%). ^1^Н-NMR (CDCl_3_, 400 MHz) δ, ppm: 6.80 s (1H, C^2^H), 7.31-7.38 m (3H_arom_), 7.42 td (1H_arom._, ^3^*J* 7.4 Hz, ^4^*J* 1.5 Hz), 7.47 td (1H_arom._, ^3^*J* 7.4 Hz, ^4^*J* 1.1 Hz), 7.49-7.55 m (3H_arom._), 7.71 d (1H_arom._, ^3^*J* 7.4 Hz), 7.78 d (2H_arom._, ^3^*J* 8.8 Hz), 8.34 d (2H_arom._, ^3^*J* 8.8 Hz). ^13^C-NMR (CDCl_3_, 100 MHz) δ, ppm: 64.7 q (C^1^, *J*_C-F_ 27.0 Hz), 121.5, 124.2, 126.3 q (*J*_C-F_ 0.5 Hz), 126.5 q (CF_3_, *J*_C-F_ 282.7 Hz), 127.4, 128.0 q (*J*_C-F_ 1.7 Hz), 128.5, 128.8, 129.0, 129.1, 133.8, 134.7 q (*J*_C-F_ 1.8 Hz), 140.9, 142.5, 143.7, 145.6, 148.0. ^19^F-NMR (CDCl_3_, 376 MHz) δ, ppm: –67.40 s (CF_3_). HRMS (MALDI-TOF): C_22_H_15_F_3_NO_2_ found 382.1051 [M + H]^+^; calcd. 382.1049.

**1,3-Bis(4-chlorophenyl)-1-(trifluoromethyl)-*1H*-indene (4an):** Colorless solid, mp 110°С (МеОН-CH_2_Cl_2_). The reaction scale is 30 mg (0.09 mmol) alcohol **1q** and 0.4 mL benzene, isolated amount is 27 mg **4an** (yield 70%). ^1^Н-NMR (CDCl_3_, 400 MHz) δ, ppm: 6.61 s (1H, C^2^H), 7.30 d (2H_arom._, ^3^*J* 8.6 Hz), 7.38 td (1H_arom._, ^3^*J* 7.4 Hz, ^4^*J* 1.0 Hz), 7.43-7.48 m (5H_arom._), 7.52 d (1H_arom._, ^3^*J* 7.4 Hz), 7.55 d (2H_arom._, ^3^*J* 8.6 Hz), 7.64 d (1H, C^7^H, ^3^*J* 8.6 Hz). ^13^C-NMR (CDCl_3_, 100 MHz) δ, ppm: 63.9 q (C^1^, *J*_C-F_ 27.2 Hz), 121.8, 125.8 q (C^7^, *J*_C-F_ 0.7 Hz), 126.5 q (CF_3_, *J*_C-F_ 282.6 Hz), 127.1, 129.0, 129.1, 129.17, 129.22, 129.4 q (*J*_C-F_ 1.8 Hz), 132.0 q (C^2^, *J*_C-F_ 1.7 Hz), 132.6, 133.0, 134.4, 134.8, 143.2, 143.5 q (*J*_C-F_ 0.6 Hz), 146.7. ^19^F-NMR (CDCl_3_, 376 MHz) δ, ppm: –67.57 s (CF_3_). HRMS (MALDI-TOF): C_22_H_14_F_3_Cl_2_ found 405.0431 [M + H]^+^; calcd. 405.0419.

**1,3-Bis(4-bromophenyl)-4,7-dimethyl-1-(trifluoromethyl)-*1H*-indene (4ao):** Yellow oil. The reaction scale is 40 mg (0.1 mmol) alcohol **1r** and 0.6 mL benzene, isolated amount is 36 mg **4ao** (yield 80%). ^1^Н-NMR (CDCl_3_, 400 MHz) δ, ppm: 6.60 s (1H, C^2^H), 7.38 td (1H_arom._, ^3^*J* 7.5 Hz, ^4^*J* 1.3 Hz), 7.39 d (2H_arom._, ^3^*J* 8.6 Hz), 7.43-7.46 m (1H_arom._), 7.45 d (2H_arom._, ^3^*J* 8.6 Hz), 7.47 d (2H_arom._, ^3^*J* 8.5 Hz), 7.51 d (1H_arom._, ^3^*J* 7.5 Hz), 7.61 d (2H_arom._, ^3^*J* 8.5 Hz), 7.62-7.64 m (1H_arom._). ^13^C-NMR (CDCl_3_, 100 MHz) δ, ppm: 64.0 q (C^1^, *J*_C-F_ 27.2 Hz), 121.8, 122.6, 123.0, 125.8, 126.4 q (CF_3_, *J*_C-F_ 282.6 Hz), 127.1, 129.1, 129.5, 129.7 q (*J*_C-F_ 1.7 Hz), 132.0 (2С), 132.1, 133.0, 133.5, 143.1, 143.5, 146.8. ^19^F-NMR (CDCl_3_, 376 MHz) δ, ppm: –67.54 s (CF_3_). HRMS (MALDI-TOF): C_22_H_14_F_3_Br_2_ found 492.9397 [M + H]^+^; calcd. 492.9409.

**4,7-Dimethyl-1,3-diphenyl-1-(trifluoromethyl)-*1H*-indene (4ba):** Colorless solid, mp 106°С (МеОН-CH_2_Cl_2_). The reaction scale is 30 mg (0.1 mmol) alcohol **1a** and 12 mg (0.11 mmol) *p*-xylene, isolated amount is 30 mg **4ba** (yield 75%). ^1^Н-NMR (CDCl_3_, 400 MHz) δ, ppm: 1.97 s (3H, CH_3_), 2.05 s (3H, CH_3_), 6.25 s (1H, C^2^H), 6.97 d (1H, C^6^H, ^3^*J* 7.8 Hz), 7.05 d (1H, C^5^H, ^3^*J* 7.8 Hz), 7.28-7.34 m (5H_arom._), 7.37-7.43 m (5H_arom._). ^13^C-NMR (CDCl_3_, 100 MHz) δ, ppm: 19.2 q (CH_3_, *J*_C-F_ 3.8 Hz), 20.1 (CH_3_), 65.3 q (C^1^, *J*_C-F_ 26.5 Hz), 127.0 q (*J*_C-F_ 1.9 Hz), 127.3 q (CF_3_, *J*_C-F_ 283.5 Hz), 127.6, 127.9, 128.2, 128.7, 128.9, 129.9, 130.2,132.4, 133.3, 134.3, 136.4 q (*J*_C-F_ 2.1 Hz), 137.6, 143.0, 143.2, 148.3. ^19^F-NMR (CDCl_3_, 376 MHz) δ, ppm: –62.43 s (CF_3_). HRMS (MALDI-TOF): C_24_H_20_F_3_ found 365.1504 [M + H]^+^; calcd. 365.1512.

**1-(Trifluoromethyl)-4,7-dimethyl-1-(3-methylphenyl)-3-phenyl-*1H*-indene (4bb):** Pale yellow solid, mp 145°С (МеОН-CH_2_Cl_2_). The reaction scale is 30 mg (0.1 mmol) alcohol **1b** and 12 mg (0.11 mmol) *p*-xylene, isolated amount is 24 mg **4bb** (yield 66%). ^1^Н-NMR (CDCl_3_, 400 MHz) δ, ppm: 1.96 s (3H, CH_3_), 2.06 s (3H, CH_3_), 2.33 s (3H, CH_3_), 6.22 s (1H, C^2^H), 6.96 d (1H, C^6^H, ^3^*J* 7.8 Hz), 7.04 d (1H, C^5^H, ^3^*J* 7.8 Hz), 7.10 d (2H_arom._, ^3^*J* 7.6 Hz), 7.14 s (1H_arom._), 7.17-7.21 m (1H_arom._), 7.35-7.42 m (5H_arom._). ^13^C-NMR (CDCl_3_, 100 MHz) δ, ppm: 19.3 q (CH_3_, *J*_C-F_ 3.8 Hz), 20.1 (CH_3_), 21.8 (CH_3_), 65.2 q (C^1^, *J*_C-F_ 26.3 Hz), 124.0 q (*J*_C-F_ 2.0 Hz), 127.3 q (CF_3_, *J*_C-F_ 283.8 Hz), 127.6 q (*J*_C-F_ 2.0 Hz), 127.8, 128.2, 128.4, 128.70, 128.72, 129.8, 130.1, 132.3, 133.3, 134.1, 136.5 q (*J*_C-F_ 2.0 Hz), 137.7, 138.5, 143.0, 143.1 q (*J*_C-F_ 1.0 Hz), 148.2. ^19^F-NMR (CDCl_3_, 376 MHz) δ, ppm: –62.33 s (CF_3_). HRMS (MALDI-TOF): C_25_H_22_F_3_ found 379.1680 [M + H]^+^; calcd. 379.1669.

**4,7-Dimethyl-1-(4-methylphenyl)-3-phenyl-1-(trifluoromethyl)-*1H*-indene (4bc):** Colorless solid, mp 148°С (МеОН-CH_2_Cl_2_). The reaction scale is 30 mg (0.1 mmol) alcohol **1c** and 12 mg (0.11 mmol) *p*-xylene, isolated amount is 18 mg **4bc** (yield 44%). ^1^Н-NMR (CDCl_3_, 400 MHz) δ, ppm: 1.95 s (3H, CH_3_), 2.06 s (3H, CH_3_), 2.34 s (3H, CH_3_), 6.21 s (1H, C^2^H), 6.95 d (1H, C^6^H, ^3^*J* 7.8 Hz), 7.03 d (1H, C^5^H, ^3^*J* 7.8 Hz), 7.11 d (2H_arom._, ^3^*J* 8.1 Hz), 7.20 d (2H_arom._, ^3^*J* 8.1 Hz), 7.33-7.41 m (5H_arom._). ^13^C-NMR (CDCl_3_, 100 MHz) δ, ppm: 19.3 q (CH_3_, *J*_C-F_ 3.8 Hz), 20.1 (CH_3_), 21.1 (CH_3_), 65.0 q (C^1^, *J*_C-F_ 26.4 Hz), 126.9 q (*J*_C-F_ 1.9 Hz), 127.4 q (CF_3_, *J*_C-F_ 283.6 Hz), 127.8, 128.2, 128.7, 129.6, 129.8, 130.1, 131.1, 132.3, 133.3, 136.6 q (*J*_C-F_ 2.0 Hz), 137.4, 137.7, 143.0, 143.1 q (*J*_C-F_ 1.1 Hz), 148.1. ^19^F-NMR (CDCl_3_, 376 MHz) δ, ppm: –62.53 s (CF_3_). HRMS (MALDI-TOF): C_25_H_22_F_3_ found 379.1674 [M + H]^+^; calcd. 379.1669.

**1-(3,4-Dimethylphenyl)-4,7-dimethyl-3-phenyl-1-(trifluoromethyl)-*1H*-indene (4bd):** Colorless solid, mp 181°С (МеОН-CH_2_Cl_2_). The reaction scale is 30 mg (0.1 mmol) alcohol **1d** and 12 mg (0.11 mmol) *p*-xylene, isolated amount is 28 mg **4bd** (yield 72%). ^1^Н-NMR (CDCl_3_, 400 MHz) δ, ppm: 1.96 s (3H, CH_3_), 2.08 s (3H, CH_3_), 2.24 s (3H, CH_3_), 2.25 s (3H, CH_3_), 6.21 s (1H, C^2^H), 6.96 d (1H, C^6^H, ^3^*J* 7.8 Hz), 7.03-7.07 m (3H_arom_), 7.09 s (1H_arom._), 7.34-7.42 m (5H_arom._). ^13^C-NMR (CDCl_3_, 100 MHz) δ, ppm: 19.4 q (CH_3_, *J*_C-F_ 3.8 Hz), 19.5 (CH_3_), 20.2 (CH_3_), 20.2 (CH_3_), 65.0 q (C^1^, *J*_C-F_ 26.2 Hz), 124.4 q (*J*_C-F_ 1.8 Hz), 127.4 q (CF_3_, *J*_C-F_ 283.8 Hz), 127.8, 128.1 q (*J*_C-F_ 1.8 Hz), 128.1, 128.7, 129.8, 130.06, 130.08, 131.3, 132.2, 133.3 136.0, 136.6 q (*J*_C-F_ 2.0 Hz), 137.0, 137.7, 142.98, 143.0 q (*J*_C-F_ 0.8 Hz), 147.9. ^19^F-NMR (CDCl_3_, 376 MHz) δ, ppm: –62.37 s (CF_3_). HRMS (MALDI-TOF): C_26_H_24_F_3_ found 393.1813 [M + H]^+^; calcd. 393.1825.

**1-(4-Chlorophenyl)-4,7-dimethyl-3-phenyl-1-(trifluoromethyl)-*1H*-indene (4be):** Colorless solid, mp 138°С (МеОН-CH_2_Cl_2_). The reaction scale is 30 mg (0.1 mmol) alcohol **1e** and 12 mg (0.11 mmol) *p*-xylene, isolated amount is 24 mg **4be** (yield 60%). ^1^Н-NMR (CDCl_3_, 400 MHz) δ, ppm: 1.95 s (3H, CH_3_), 2.04 s (3H, CH_3_), 6.18 s (1H, C^2^H), 6.96 d (1H, C^6^H, ^3^*J* 7.8 Hz), 7.04 d (1H, C^5^H, ^3^*J* 7.8 Hz), 7.23-7.28 m (4H_arom._), 7.33-7.42 m (5H_arom._). ^13^C-NMR (CDCl_3_, 100 MHz) δ, ppm: 19.3 q (CH_3_, *J*_C-F_ 3.8 Hz), 20.1 (CH_3_), 64.8 q (C^1^, *J*_C-F_ 26.8 Hz), 127.1 q (CF_3_, *J*_C-F_ 283.6 Hz), 128.0, 128.2, 128.5 q (*J*_C-F_ 2.0 Hz), 128.6, 129.1, 130.0, 130.4, 132.6, 133.0, 133.2, 133.6, 135.9 q (*J*_C-F_ 2.1 Hz), 137.4, 142.7 q (*J*_C-F_ 2.1 Hz), 142.8, 148.7. ^19^F-NMR (CDCl_3_, 376 MHz) δ, ppm: –62.63 s (CF_3_). HRMS (MALDI-TOF): C_24_H_19_F_3_Cl found 399.1123 [M + H]^+^; calcd. 399.1122.

**1-(4-Bromophenyl)-4,7-dimethyl-3-phenyl-1-(trifluoromethyl)-*1H*-indene (4bf):** Pale yellow solid, mp 136°С (МеОН-CH_2_Cl_2_). The reaction scale is 30 mg (0.09 mmol) alcohol **1f** and 12 mg (0.11 mmol) *p*-xylene, isolated amount is 28 mg **4bf** (yield 74%). ^1^Н-NMR (CDCl_3_, 400 MHz) δ, ppm: 1.95 s (3H, CH_3_), 2.04 s (3H, CH_3_), 6.18 s (1H, C^2^H), 6.97 d (1H, C^6^H, ^3^*J* 7.8 Hz), 7.05 d (1H, C^5^H, ^3^*J* 7.8 Hz), 7.18 d (2H_arom._, ^3^*J* 8.6 Hz), 7.34-7.42 m (5H_arom._), 7.43 d (2H_arom._, ^3^*J* 8.6 Hz). ^13^C-NMR (CDCl_3_, 100 MHz) δ, ppm: 19.3 q (CH_3_, *J*_C-F_ 3.8 Hz), 20.1 (CH_3_), 64.9 q (C^1^, *J*_C-F_ 26.7 Hz), 121.6, 127.1 q (CF_3_, *J*_C-F_ 283.7 Hz), 128.0, 128.2, 128.6, 128.8 q (*J*_C-F_ 2.0 Hz), 130.0, 130.4, 132.0, 132.6, 133.2, 133.6, 135.8 q (*J*_C-F_ 2.1 Hz), 137.3, 142.7 q (*J*_C-F_ 2.1 Hz), 142.9, 148.8. ^19^F-NMR (CDCl_3_, 376 MHz) δ, ppm: –62.61 s (CF_3_). HRMS (MALDI-TOF): C_24_H_19_F_3_Br found 443.0619 [M + H]^+^; calcd. 443.0617.

**1-(2-Bromophenyl)-4,7-dimethyl-3-phenyl-1-(trifluoromethyl)-*1H*-indene (4bg):** Colorless solid, mp 103°С (МеОН-CH_2_Cl_2_). The reaction scale is 30 mg (0.09 mmol) alcohol **1g** and 12 mg (0.11 mmol) *p*-xylene, isolated amount is 17 mg **4bg** (yield 45%). ^1^Н-NMR (CDCl_3_, 400 MHz) δ, ppm: 1.89 s (3H, CH_3_), 1.93 s (3H, CH_3_), 6.06 s (1H, C^2^H), 6.90 d (1H, C^6^H, ^3^*J* 7.8 Hz), 7.02 d (1H, C^5^H, ^3^*J* 7.8 Hz), 7.17 td (1H_arom._, ^3^*J* 7.9 Hz, ^4^*J* 1.3 Hz), 7.35-7.45 m (6H_arom._), 7.53 dd (1H_arom._, ^3^*J* 7.9 Hz, ^4^*J* 1.3 Hz), 7.94-7.98 m (1H_arom._). ^13^C-NMR (CDCl_3_, 100 MHz) δ, ppm: 18.5 q (CH_3_, *J*_C-F_ 3.1 Hz), 20.0 (CH_3_), 66.1 q (C^1^, *J*_C-F_ 25.1 Hz), 121.9 q (*J*_C-F_ 0.9 Hz), 127.1 q (CF_3_, *J*_C-F_ 285.2 Hz), 127.8, 127.9, 128.2, 128.3, 129.0, 129.3, 130.5 q (*J*_C-F_ 4.2 Hz), 130.6, 131.3 q (*J*_C-F_ 1.2 Hz), 132.4, 132.9, 133.3, 136.1, 138.0, 141.4 q (*J*_C-F_ 0.4 Hz), 144.8, 152.3. ^19^F-NMR (CDCl_3_, 376 MHz) δ, ppm: –65.08 s (CF_3_). HRMS (MALDI-TOF): C_24_H_19_F_3_Br found 443.0618 [M + H]^+^; calcd. 443.0617.

**4,7-Dimethyl-1-(4-nitrophenyl)-3-phenyl-1-(trifluoromethyl)-*1H*-indene (4bh):** Yellow oil. The reaction scale is 30 mg (0.09 mmol) alcohol **1h**, 35 mg (0.23 mmol) TfOH and 12 mg (0.11 mmol) *p*-xylene, isolated amount is 31 mg **4bh** (yield 84%). ^1^Н-NMR (CDCl_3_, 400 MHz) δ, ppm: 1.97 s (3H, CH_3_), 2.00 s (3H, CH_3_), 6.19 s (1H, C^2^H), 6.99 d (1H, C^6^H, ^3^*J* 7.8 Hz), 7.08 d (1H, C^5^H, ^3^*J* 7.8 Hz), 7.35-7.43 m (5H_arom._), 7.48 d (2H_arom._, ^3^*J* 8.8 Hz), 8.16 d (2H_arom._, ^3^*J* 8.8 Hz). ^13^C-NMR (CDCl_3_, 100 MHz) δ, ppm: 19.3 q (CH_3_, *J*_C-F_ 3.6 Hz), 20.0 (CH_3_), 65.1 q (C^1^, *J*_C-F_ 27.0 Hz), 124.1, 126.8 q (CF_3_, *J*_C-F_ 283.6 Hz), 128.1 q (*J*_C-F_ 2.1 Hz), 128.2, 128.3, 128.5, 130.3 (2C), 130.8, 133.0, 134.9 q (*J*_C-F_ 1.9 Hz), 136.9, 142.3, 142.5, 142.8, 147.3, 149.9. ^19^F-NMR (CDCl_3_, 376 MHz) δ, ppm: –62.53 s (CF_3_). HRMS (MALDI-TOF): C_24_H_19_F_3_NO_2_ found 410.1374 [M + H]^+^; calcd. 410.1363.

**3-(4-Methylphenyl)-4,7-dimethyl-1-phenyl-1-(trifluoromethyl)-*1H*-indene (4bi):** Colorless solid, mp 136°С (МеОН-CH_2_Cl_2_). The reaction scale is 30 mg (0.1 mmol) alcohol **1i** and 12 mg (0.11 mmol) *p*-xylene, isolated amount is 27 mg **4bi** (yield 71%). ^1^Н-NMR (CDCl_3_, 400 MHz) δ, ppm: 1.98 s (3H, CH_3_), 2.04 s (3H, CH_3_), 2.41 s (3H, CH_3_), 6.21 s (1H, C^2^H), 6.95 d (1H, C^6^H, ^3^*J* 7.8 Hz), 7.04 d (1H, C^5^H ^3^*J* 7.8 Hz), 7.20 d (2H_arom._, ^3^*J* 7.3 Hz), 7.25-7.35 m (7H_arom._). ^13^C-NMR (CDCl_3_, 100 MHz) δ, ppm: 19.2 q (CH_3_, *J*_C-F_ 3.7 Hz), 20.2 (CH_3_), 21.4 (CH_3_), 65.2 q (C^1^, *J*_C-F_ 26.3 Hz), 127.0 q (*J*_C-F_ 1.9 Hz), 127.3 q (CF_3_, *J*_C-F_ 283.8 Hz), 127.6, 128.6, 128.8, 128.9, 129.8, 130.2, 132.3, 133.2, 134.4, 134.6, 136.3 q (*J*_C-F_ 2.0 Hz), 137.6, 143.1, 143.2, 148.3. ^19^F-NMR (CDCl_3_, 376 MHz) δ, ppm: –62.46 s (CF_3_). HRMS (MALDI-TOF): C_25_H_22_F_3_ found 379.1652 [M + H]^+^; calcd. 379.1669.

 **1-Phenyl-4,7-dimethyl-3-(3,4-dimethylphenyl)-1-(trifluoromethyl)-*1H*-indene (4bj)** and **3-(2,5-dimethylphenyl)–6,7-dimethyl-1-phenyl-1-(trifluoromethyl)-*1H*-indene (5ba):** The reaction scale is 30 mg (0.1 mmol) alcohol **1k** and 12 mg (0.11 mmol) *p*-xylene, isolated amount is 11 mg mixture of **4bj**, **5ba** (total yield 28%, ratio **4bj** : **5ba** =1.5:1, respectively) and 13 mg **5bb**. Compound **4bj**: ^1^Н-NMR (CDCl_3_, 400 MHz, measured in a mixture with **5ba**) δ, ppm: 1.99 s (3H, CH_3_), 2.03 s (3H, CH_3_), 2.29 s (3H, CH_3_), 2.31 s (3H, CH_3_), 6.19 s (1H, C^2^H), 6.94 d (1H, C^6^H, ^3^*J* 7.8 Hz), 7.03 d (1H, C^5^H, ^3^*J* 7.8 Hz), 7.08-7.10 m (2H_arom._), 7.14 s (1H_arom._), 7.27-7.34 m (5H_arom._). ^13^C-NMR (CDCl_3_, 100 MHz, measured in a mixture with **5ba**) δ, ppm: 19.2 q (CH_3_, *J*_C-F_ 3.6 Hz), 19.7 (CH_3_), 19.8 (CH_3_), 20.2 (CH_3_), 65.2 q (C^1^, *J*_C-F_ 26.4 Hz), 126.0, 127.0 q (*J*_C-F_ 1.7 Hz), 127.3 q (CF_3_, *J*_C-F_ 280.9 Hz), 127.5, 128.8, 128.9, 129.8, 130.5, 132.3, 133.2, 134.5, 135.0, 135.3, 136.2, 136.4, 143.1, 144.4, 146.8, 148.4. ^19^F-NMR (CDCl_3_, 376 MHz, measured in a mixture with **5ba**) δ, ppm: –62.47 s (CF_3_). HRMS (MALDI-TOF): C_26_H_24_F_3_ found 393.1815 [M + H]^+^; calcd. 393.1825. Compound **5ba**: ^1^Н-NMR (CDCl_3_, 400 MHz, measured in a mixture with **4bj**) δ, ppm: 1.99 s (3H, CH_3_), 2.21 s (3H, CH_3_), 2.28 s (3H, CH_3_), 2.34 s (3H, CH_3_), 6.20 s (1H, C^2^H), 6.83 d (1H_arom._, ^3^*J* 7.6 Hz), 7.12-7.19 m (4H_arom._), 7.27-7.34 m (5H_arom._). ^13^C-NMR (CDCl_3_, 100 MHz, measured in a mixture with **4bj**) δ, ppm: 16.4 q (CH_3_, *J*_C-F_ 3.6 Hz), 19.6 (CH_3_), 20.4 (CH_3_), 21.0 (CH_3_), 66.0 q (C^1^, *J*_C-F_ 26.1 Hz), 119.0, 127.0 q (*J*_C-F_ 1.9 Hz), 127.4 q (CF_3_, *J*_C-F_ 279.4 Hz), 127.6, 128.8, 129.0, 129.4, 129.7, 130.2, 130.3, 133.3, 134.1, 134.6, 134.7 q (*J*_C-F_ 1.6 Hz), 134.9, 136.5, 143.0, 143.2, 144.4. ^19^F-NMR (CDCl_3_, 376 MHz, measured in a mixture with **4bj**) δ, ppm: –62.92 s (CF_3_). HRMS (MALDI-TOF): C_26_H_24_F_3_ found 393.1815 [M + H]^+^; calcd. 393.1825.

**3-(4-Chlorophenyl)-4,7-dimethyl-1-phenyl-1-(trifluoromethyl)-*1H*-indene (4bk):** Colorless solid, mp 138°С (МеОН-CH_2_Cl_2_). The reaction scale is 30 mg (0.1 mmol) alcohol **1l** and 12 mg (0.11 mmol) *p*-xylene, isolated amount is 27 mg **4bk** (yield 68%). ^1^Н-NMR (CDCl_3_, 400 MHz) δ, ppm: 1.97 s (3H, CH_3_), 2.04 s (3H, CH_3_), 6.23 s (1H, C^2^H), 6.97 d (1H, C^6^H, ^3^*J* 7.8 Hz), 7.04 d (1H, C^5^H, ^3^*J* 7.8 Hz), 7.26-7.32 m (7H_arom._), 7.38 d (2H_arom._, ^3^*J* 8.6 Hz). ^13^C-NMR (CDCl_3_, 100 MHz) δ, ppm: 19.2 q (CH_3_, *J*_C-F_ 3.7 Hz), 20.3 (CH_3_), 65.3 q (C^1^, *J*_C-F_ 26.4 Hz), 127.0 q (*J*_C-F_ 1.9 Hz), 127.2 q (CF_3_, *J*_C-F_ 283.7 Hz), 127.7, 128.5, 128.9, 130.0, 130.06, 130.09, 132.4, 133.4, 133.9, 134.0, 136.0, 136.9 q (*J*_C-F_ 2.1 Hz), 142.6, 143.1 q (*J*_C-F_ 1.1 Hz), 147.2. ^19^F-NMR (CDCl_3_, 376 MHz) δ, ppm: –62.41 s (CF_3_). HRMS (MALDI-TOF): C_24_H_19_F_3_Cl found 399.1126 [M + H]^+^; calcd. 399.1122.

**3-(4-Bromophenyl)-1-(trifluoromethyl)-4,7-dimethyl-1-phenyl-*1H*-indene (4bl):** Colorless solid, mp 161°С (МеОН-CH_2_Cl_2_). The reaction scale is 30 mg (0.09 mmol) alcohol **1m** and 11 mg (0.1 mmol) *p*-xylene, isolated amount is 24 mg **4bl** (yield 58%). ^1^Н-NMR (CDCl_3_, 400 MHz) δ, ppm: 1.97 s (3H, CH_3_), 2.03 s (3H, CH_3_), 6.23 s (1H, C^2^H), 6.97 d (1H, C^6^H, ^3^*J* 7.8 Hz), 7.04 d (1H, C^5^H, ^3^*J* 7.8 Hz), 7.25 d (2H_arom._, ^3^*J* 8.4 Hz), 7.27-7.32 m (5H_arom._), 7.53 d (2H_arom._, ^3^*J* 8.4 Hz). ^13^C-NMR (CDCl_3_, 100 MHz) δ, ppm: 19.2 q (CH_3_, *J*_C-F_ 3.8 Hz), 20.3 (CH_3_), 65.3 q (C^1^, *J*_C-F_ 26.4 Hz), 122.0, 127.0 q (*J*_C-F_ 2.0 Hz), 127.2 q (CF_3_, *J*_C-F_ 283.7 Hz), 127.7, 128.9, 130.0, 130.1, 130.4, 131.4, 132.4, 133.4, 134.0, 136.5, 136.8 q (*J*_C-F_ 2.1 Hz), 142.5, 143.1 q (*J*_C-F_ 1.1 Hz), 147.2. ^19^F-NMR (CDCl_3_, 376 MHz) δ, ppm: –62.41 s (CF_3_). HRMS (MALDI-TOF): C_24_H_19_F_3_Br found 443.0617 [M + H]^+^; calcd. 443.0617.

**4,7-Dimethyl-3-(4-nitrophenyl)-1-phenyl-1-(trifluoromethyl)-*1H*-indene (4bm):** Colorless solid, mp 176°С (МеОН-CH_2_Cl_2_). The reaction scale is 30 mg (0.1 mmol) alcohol **1n**, 35 mg (0.23 mmol) TfOH and 12 mg (0.11 mmol) *p*-xylene, isolated amount is 29 mg **4bm** (yield 70%). ^1^Н-NMR (CDCl_3_, 400 MHz) δ, ppm: 1.95 s (3H, CH_3_), 2.04 s (3H, CH_3_), 6.30 s (1H, C^2^H), 7.00 d (1H_arom._, ^3^*J* 7.8 Hz), 7.06 d (1H_arom._, ^3^*J* 7.8 Hz), 7.28-7.33 m (5H_arom._), 7.55 d (2H_arom._, ^3^*J* 8.7 Hz), 8.27 d (2H_arom._, ^3^*J* 8.7 Hz). ^13^C-NMR (CDCl_3_, 100 MHz) δ, ppm: 19.3 q (CH_3_, *J*_C-F_ 3.6 Hz), 20.5 (CH_3_), 65.7 q (C^1^, *J*_C-F_ 26.7 Hz), 123.6, 126.9 q (*J*_C-F_ 1.9 Hz), 127.0 q (CF_3_, *J*_C-F_ 283.8 Hz), 128.0, 129.1, 129.7, 129.8, 130.5, 132.5, 133.5, 133.7, 137.6 q (*J*_C-F_ 2.0 Hz), 142.0, 143.0 q (*J*_C-F_ 0.6 Hz), 144.6, 146.4, 147.7. ^19^F-NMR (CDCl_3_, 376 MHz) δ, ppm: –62.32 s (CF_3_). HRMS (MALDI-TOF): C_24_H_19_F_3_NO_2_ found 410.1373 [M + H]^+^; calcd. 410.1363.

**1,3-Di(4-methylphenyl)-4,7-dimethyl-1-(trifluoromethyl)-*1H*-indene (4bn):** Colorless oil. The reaction scale is 30 mg (0.1 mmol) alcohol **1o** and 12 mg (0.11 mmol) *p*-xylene, isolated amount is 23 mg **4bn** (yield 59%). ^1^Н-NMR (CDCl_3_, 400 MHz) δ, ppm: 1.98 s (3H, CH_3_), 2.05 s (3H, CH_3_), 2.34 s (3H, CH_3_), 2.40 s (3H, CH_3_), 6.19 s (1H, C^2^H), 6.95 d (1H, C^6^H, ^3^*J* 7.8 Hz), 7.03 d (1H, C^5^H, ^3^*J* 7.8 Hz), 7.10 d (2H_arom._, ^3^*J* 8.2 Hz), 7.19-7.22 m (4H_arom._), 7.25 d (2H_arom._, ^3^*J* 8.2 Hz). ^13^C-NMR (CDCl_3_, 100 MHz) δ, ppm: 19.3 q (CH_3_, *J*_C-F_ 3.8 Hz), 20.2 (CH_3_), 20.1 (CH_3_), 21.4 (CH_3_), 64.9 q (C^1^, *J*_C-F_ 26.4 Hz), 126.9 q (*J*_C-F_ 1.9 Hz), 127.4 q (CF_3_, *J*_C-F_ 283.7 Hz), 128.6, 128.8, 129.6, 129.7, 130.1, 131.2, 132.3, 133.2, 134.7, 136.5 q (*J*_C-F_ 2.1 Hz), 137.3, 137.5, 143.1, 143.2 q (*J*_C-F_ 0.9 Hz), 148.1. ^19^F-NMR (CDCl_3_, 376 MHz) δ, ppm: –62.53 s (CF_3_). HRMS (MALDI-TOF): C_26_H_24_F_3_ found 393.1840 [M + H]^+^; calcd. 393.1825.

 **1,3-Di(3,4-dimethylphenyl)-4,7-dimethyl-1-(trifluoromethyl)-*1H*-indene (4bo)** and **1-(3,4-dimethylphenyl)-3-(2,5-dimethylphenyl)–6,7-dimethyl-1-(trifluoromethyl)-*1H*-indene (5bc):** The reaction scale is 50 mg (0.16 mmol) alcohol **1p** and 20 mg (0.18 mmol) *p*-xylene, isolated amount is 16 mg mixture of **4bo** and **5bc** (total yield 25%, ratio **4bo** : **5bc** =1.4:1, respectively) and 16 mg **5bd**. Compound **4bo**: ^1^Н-NMR (CDCl_3_, 400 MHz, measured in a mixture with **5bc**) δ, ppm: 1.98 s (3H, CH_3_), 2.06 s (3H, CH_3_), 2.22 s (3H, CH_3_), 2.24 s (3H, CH_3_), 2.28 s (3H, CH_3_), 2.30 s (3H, CH_3_), 6.16 s (1H, C^2^H), 6.94 d (1H, C^6^H, ^3^*J* 7.8 Hz), 7.01-7.12 m (6H_arom._), 7.17 d (1H_arom._, ^3^*J* 7.4 Hz). ^13^C-NMR (CDCl_3_, 100 MHz, measured in a mixture with **5bc**) δ, ppm: 19.4 q (CH_3_, *J*_C-F_ 3.8 Hz), 19.5 (CH_3_), 19.7 (CH_3_), 19.8 (CH_3_), 20.19 (CH_3_), 20.23 (CH_3_), 64.9 q (C^1^, *J*_C-F_ 26.3 Hz), 118.9, 124.4 q (*J*_C-F_ 2.0 Hz), 126.1, 127.4 q (CF_3_, *J*_C-F_ 283.8 Hz), 128.1 q (*J*_C-F_ 2.0 Hz), 129.4, 129.6, 129.9, 132.2, 133.2, 134.6, 136.1, 135.3, 136.0, 136.3, 136.3 q (*J*_C-F_ 2.1 Hz), 137.0, 143.08 q (*J*_C-F_ 1.1 Hz), 143.15, 146.4, 148.0. ^19^F-NMR (CDCl_3_, 376 MHz, measured in a mixture with **5bc**) δ, ppm: –62.40 s (CF_3_). HRMS (MALDI-TOF): C_28_H_28_F_3_ found 421.2124 [M + H]^+^; calcd. 421.2138. Compound **5bc**: ^1^Н-NMR (CDCl_3_, 400 MHz, measured in a mixture with **4bo**) δ, ppm: 2.02 s (3H, CH_3_), 2.21 s (3H, CH_3_), 2.23 s (3H, CH_3_), 2.25 s (3H, CH_3_), 2.28 s (3H, CH_3_), 2.34 s (3H, CH_3_), 6.17 s (1H, C^2^H), 6.82 d (1H_arom._, ^3^*J* 7.6 Hz), 7.01-7.12 m (6H_arom._), 7.15-7.18 m (1H_arom._). ^13^C-NMR (CDCl_3_, 100 MHz, measured in a mixture with **4bo**) δ, ppm: 16.5 q (CH_3_, *J*_C-F_ 3.6 Hz), 19.5 (CH_3_), 19.6 (CH_3_), 20.2 (CH_3_), 20.4 (CH_3_), 21.0 (CH_3_), 65.8 q (C^1^, *J*_C-F_ 25.5 Hz), 124.4 q (*J*_C-F_ 2.0 Hz), 127.3 q (CF_3_, *J*_C-F_ 279.1 Hz), 128.1 q (*J*_C-F_ 2.0 Hz), 128.9, 129.9, 130.0, 130.11, 130.12, 130.3, 130.4, 131.5, 131.9, 132.2, 133.3, 134.2, 134.9 q (*J*_C-F_ 2.0 Hz), 136.0, 136.4, 137.1, 142.9 q (*J*_C-F_ 1.1 Hz), 144.4. ^19^F-NMR (CDCl_3_, 376 MHz, measured in a mixture with **4bo**) δ, ppm: –62.89 s (CF_3_). HRMS (MALDI-TOF): C_28_H_28_F_3_ found 421.2124 [M + H]^+^; calcd. 421.2138.

**1,3-Bis(4-chlorophenyl)-1-(trifluoromethyl)-4,7-dimethyl-*1H*-indene (4bp):** Colorless solid, mp 167°С (МеОН-CH_2_Cl_2_). The reaction scale is 30 mg (0.09 mmol) alcohol **1q** and 11 mg (0.1 mmol) *p*-xylene, isolated amount is 28 mg **4bp** (yield 74%). ^1^Н-NMR (CDCl_3_, 400 MHz) δ, ppm: 1.96 s (3H, CH_3_), 2.03 s (3H, CH_3_), 6.18 s (1H, C^2^H), 6.98 d (1H, C^6^H, ^3^*J* 7.8 Hz), 7.05 d (1H, C^5^H, ^3^*J* 7.8 Hz), 7.22 d (2H_arom._, ^3^*J* 8.6 Hz), 7.26-7.30 m (4H_arom._), 7.38 d (2H_arom._, ^3^*J* 8.6 Hz). ^13^C-NMR (CDCl_3_, 100 MHz) δ, ppm: 19.3 q (CH_3_, *J*_C-F_ 3.7 Hz), 20.2 (CH_3_), 64.9 q (C^1^, *J*_C-F_ 26.7 Hz), 128.3 q (CF_3_, *J*_C-F_ 283.6 Hz), 128.4 q (*J*_C-F_ 2.0 Hz), 128.5, 129.1, 130.0, 130.3, 132.7 (2С), 133.3, 133.7, 134.1, 135.8, 135.8, 136.3 q (*J*_C-F_ 2.0 Hz), 142.5, 142.6 q (*J*_C-F_ 0.9 Hz), 147.6. ^19^F-NMR (CDCl_3_, 376 MHz) δ, ppm: –62.59 s (CF_3_). HRMS (MALDI-TOF): C_24_H_18_F_3_Cl_2_ found 433.0733 [M + H]^+^; calcd. 433.0733.

**1,3-Bis(4-bromophenyl)-1-(trifluoromethyl)-4,7-dimethyl-*1H*-indene (4bq):** Colorless solid, mp 176°С (МеОН-CH_2_Cl_2_). The reaction scale is 30 mg (0.07 mmol) alcohol **1r** and 9 mg (0.08 mmol) *p*-xylene, isolated amount is 24 mg **4bq** (yield 78%). ^1^Н-NMR (CDCl_3_, 400 MHz) δ, ppm: 1.96 s (3H, CH_3_), 2.03 s (3H, CH_3_), 6.18 s (1H, C^2^H), 6.97 d (1H, C^6^H, ^3^*J* 7.8 Hz), 7.05 d (1H, C^5^H, ^3^*J* 7.8 Hz), 7.15 d (2H_arom._, ^3^*J* 8.3 Hz), 7.23 d (2H_arom._, ^3^*J* 8.3 Hz) 7.42 d (2H_arom._, ^3^*J* 8.6 Hz), 7.53 d (2H_arom._, ^3^*J* 8.6 Hz). ^13^C-NMR (CDCl_3_, 100 MHz) δ, ppm: 19.3 q (CH_3_, *J*_C-F_ 3.7 Hz), 20.3 (CH_3_), 65.0 q (C^1^, *J*_C-F_ 26.7 Hz), 121.8, 122.2, 127.0 q (CF_3_, *J*_C-F_ 283.6 Hz), 128.8 q (*J*_C-F_ 2.0 Hz), 130.3 (2С), 131.5, 132.1, 132.7, 133.26, 133.31, 136.2 q (*J*_C-F_ 1.9 Hz), 136.3 (2С), 142.4, 142.6, 147.7. ^19^F-NMR (CDCl_3_, 376 MHz) δ, ppm: –62.57 s (CF_3_). HRMS (MALDI-TOF): C_24_H_18_F_3_Br_2_ found 520.9722 [M + H]^+^; calcd. 520.9722.

 **1-(3-Methylphenyl)-4,6-dimethyl-3-phenyl-1-(trifluoromethyl)-*1H*-indene (4ca)** and **3-(2,4-dimethylphenyl)-1-(3-methylphenyl)-1-(trifluoromethyl)-*1H*-indene (5ca):** The reaction scale is 30 mg (0.1 mmol) alcohol **1b** and 12 mg (0.11 mmol) *m*-xylene, isolated amount is 25 mg mixture of **4ca** and **5ca** (total yield 66%, ratio **4ca** : **5ca** =3:1, respectively). Compound **4ca**: ^1^Н-NMR (CDCl_3_, 400 MHz, measured in a mixture with **5ca**) δ, ppm: 1.96 s (3H, CH_3_), 2.35 s (3H, CH_3_), 2.38 s (3H, CH_3_), 6.37 s (1H, C^2^H), 6.94 s (1H, C^5^H), 7.11-7.16 m (2H_arom._), 7.28 s (1H_arom._), 7.33-7.43 m (7H_arom._). ^13^C-NMR (CDCl_3_, 100 MHz, measured in a mixture with **5ca**) δ, ppm: 20.2 (CH_3_), 21.5 (CH_3_), 21.8 (CH_3_), 63.6 q (C^1^, *J*_C-F_ 26.6 Hz), 124.3, 125.0 q (*J*_C-F_ 1.8 Hz), 126.9 q (CF_3_, *J*_C-F_ 282.5 Hz), 127.9, 128.6 q (*J*_C-F_ 1.8 Hz), 128.2, 128.6, 128.7, 128.8, 132.5, 132.6, 133.3 q (*J*_C-F_ 1.7 Hz), 135.0, 136.5, 137.6, 138.3, 139.2, 144.5, 149.2. ^19^F-NMR (CDCl_3_, 376 MHz, measured in a mixture with **5ca**) δ, ppm: –67.31 s (CF_3_). HRMS (MALDI-TOF): C_25_H_22_F_3_ found 379.1648 [M + H]^+^; calcd. 379.1669. Compound **5ca:** ^1^Н-NMR (CDCl_3_, 400 MHz, measured in a mixture with **4ca**) δ, ppm: 2.24 s (3H, CH_3_), 2.35 s (3H, CH_3_), 2.39 s (3H, CH_3_), 6.50 s (1H, C^2^H), 7.07-7.11 m (1H_arom._), 7.19-7.25 m (5H_arom._), 7.29-7.43 m (4H_arom._), 7.66 d (1H_arom._, ^3^*J* 7.1 Hz). ^13^C-NMR (CDCl_3_, 100 MHz, measured in a mixture with **4ca**) δ, ppm: 20.1 (CH_3_), 21.3 (CH_3_), 21.8 (CH_3_), 64.5 q (C^1^, *J*_C-F_ 26.5 Hz), 121.9, 125.0 (*J*_C-F_ 1.8 Hz), 125.5, 126.5, 126.6, 126.8 q (CF_3_, *J*_C-F_ 282.1 Hz), 127.4, 127.6, 128.6, 128.7, 129.2, 131.0, 131.4, 133.3 q (*J*_C-F_ 1.6 Hz), 134.7, 136.3, 138.1, 138.4, 143.4 q (*J*_C-F_ 2.1 Hz), 145.1, 147.5. ^19^F-NMR (CDCl_3_, 376 MHz, measured in a mixture with **4ca**) δ, ppm: –67.46 s (CF_3_). HRMS (MALDI-TOF): C_25_H_22_F_3_ found 379.1648 [M + H]^+^; calcd. 379.1669.

**1-(4-Methylphenyl)-4,6-dimethyl-3-phenyl-1-(trifluoromethyl)-*1H*-indene (4cb)** and **3-(2,4-dimethylphenyl)-1-(4-methylphenyl)-1-(trifluoromethyl)-*1H*-indene (5cb):** The reaction scale is 30 mg (0.1 mmol) alcohol **1c** and 12 mg (0.11 mmol) *m*-xylene, isolated amount is 25 mg mixture of **4cb** and **5cb** (total yield 69%, ratio **4cb** : **5cb** =6:1, respectively). Compound **4cb**: ^1^Н-NMR (CDCl_3_, 400 MHz, measured in a mixture with **5cb**) δ, ppm: 1.97 s (3H, CH_3_), 2.35 s (3H, CH_3_), 2.39 s (3H, CH_3_), 6.38 s (1H, C^2^H), 6.94 s (1H, C^5^H), 7.15 d (2H_arom._, ^3^*J* 8.1 Hz), 7.30 s (1H, C^7^H), 7.37-7.43 m (5H_arom._), 7.45 d (2H_arom._, ^3^*J* 8.1 Hz). ^13^C-NMR (CDCl_3_, 100 MHz, measured in a mixture with **5cb**) δ, ppm: 20.2 (CH_3_), 21.1 (CH_3_), 21.5 (CH_3_), 63.4 q (C^1^, *J*_C-F_ 26.6 Hz), 124.3 q (*J*_C-F_ 0.7 Hz), 126.9 q (CF_3_, *J*_C-F_ 282.4 Hz), 127.8 q (*J*_C-F_ 1.8 Hz), 127.9, 128.2, 128.7, 129.4, 132.1, 132.5, 132.6, 133.3 q (*J*_C-F_ 1.7 Hz), 136.5, 137.6, 137.8, 139.2, 144.5 q (*J*_C-F_ 0.7 Hz), 149.1. ^19^F-NMR (CDCl_3_, 376 MHz, measured in a mixture with **5cb**) δ, ppm: –67.56 s (CF_3_). HRMS (MALDI-TOF): C_25_H_22_F_3_ found 379.1661 [M + H]^+^; calcd. 379.1669. Compound **5cb**: ^1^Н-NMR (CDCl_3_, 400 MHz, measured in a mixture with **4cb**) δ, ppm: 2.24 s (3H, CH_3_), 2.35 s (3H, CH_3_), 2.40 s (3H, CH_3_), 6.51 s (1H, C^2^H), 7.08-7.11 m (2H_arom._), 7.14-7.16 m (3H_arom._), 7.20 d (1H_arom._, ^3^*J* 7.7 Hz), 7.32-7.36 m (2H_arom._), 7.49 d (2H_arom._, ^3^*J* 8.5 Hz), 7.67 d (1H_arom._, ^3^*J* 7.1 Hz). ^13^C-NMR (CDCl_3_, 100 MHz, measured in a mixture with **4cb**) δ, ppm: 20.1 (CH_3_), 21.1 (CH_3_), 21.3 (CH_3_), 64.3 q (C^1^, *J*_C-F_ 26.9 Hz), 121.8, 125.5 q (*J*_C-F_ 0.4 Hz), 126.5, 126.6, 126.9 q (CF_3_, *J*_C-F_ 280.9 Hz), 127.8, 127.9, 128.7, 129.2, 129.5, 131.1, 131.3, 133.3 q (*J*_C-F_ 1.7 Hz), 136.3, 138.0, 138.1, 143.4, 145.1, 147.5. ^19^F-NMR (CDCl_3_, 376 MHz, measured in a mixture with **4cb**) δ, ppm: –67.72 s (CF_3_). HRMS (MALDI-TOF): C_25_H_22_F_3_ found 379.1661 [M + H]^+^; calcd. 379.1669.

 **1-(3,4-Dimethylphenyl)-4,6-dimethyl-3-phenyl-1-(trifluoromethyl)-*1H*-indene (4cc)** and **3-(2,4-dimethylphenyl)-1-(3,4-dimethylphenyl)-1-(trifluoromethyl)-*1H*-indene (5cc):** The reaction scale is 30 mg (0.1 mmol) alcohol **1d** and 12 mg (0.11 mmol) *m*-xylene, isolated amount is 27 mg mixture of **4cc** and **5cc** (total yield 69%, ratio **4cc** : **5cc** =5.7:1, respectively). Compound **4cc**: ^1^Н-NMR (CDCl_3_, 400 MHz, measured in a mixture with **5cc**) δ, ppm: 1.97 s (3H, CH_3_), 2.26 s (3H, CH_3_), 2.27 s (3H, CH_3_), 2.39 s (3H, CH_3_), 6.39 s (1H, C^2^H), 6.95 s (1H, C^5^H), 7.11 d (1H_arom._, ^3^*J* 7.8 Hz), 7.30-7.34 m (3H_arom._), 7.35-7.44 m (5H_arom._). ^13^C-NMR (CDCl_3_, 100 MHz, measured in a mixture with **5cc**) δ, ppm: 19.5 (CH_3_), 20.2 (CH_3_), 20.2 (CH_3_), 21.5 (CH_3_), 63.3 q (C^1^, *J*_C-F_ 26.5 Hz), 124.3 q (*J*_C-F_ 0.5 Hz), 125.3 q (*J*_C-F_ 1.7 Hz), 126.9 q (CF_3_, *J*_C-F_ 282.5 Hz), 127.9, 128.2, 128.7, 129.1 q (*J*_C-F_ 1.7 Hz), 130.0, 132.42, 132.44, 132.5, 133.4 q (*J*_C-F_ 1.7 Hz), 136.4, 136.5, 136.9, 137.7, 139.2, 144.5, 149.0. ^19^F-NMR (CDCl_3_, 376 MHz, measured in a mixture with **5cc**) δ, ppm: –67.46 s (CF_3_). HRMS (MALDI-TOF): C_26_H_24_F_3_ found 393.1821 [M + H]^+^; calcd. 393.1825. Compound **5cc**: ^1^Н-NMR (CDCl_3_, 400 MHz, measured in a mixture with **4cc**) δ, ppm: 2.26 s (9H, CH_3_), 2.40 s (3H, CH_3_), 6.52 s (1H, C^2^H), 7.09-7.13 m (2H_arom._), 7.16 s (1H_arom._), 7.21 d (1H_arom._, ^3^*J* 7.7 Hz), 7.30-7.34 m (2H_arom._), 7.35-7.44 m (3H_arom._), 7.68 d (1H_arom._, ^3^*J* 7.1 Hz). ^13^C-NMR (CDCl_3_, 100 MHz, measured in a mixture with **4cc**) δ, ppm: 19.5 (CH_3_), 20.1 (CH_3_), 20.2 (CH_3_), 21.3 (CH_3_), 64.3 q (C^1^, *J*_C-F_ 26.7 Hz), 121.3, 121.8, 125.4 q (*J*_C-F_ 1.9 Hz), 125.5, 126.5, 126.6, 126.7 q (CF_3_, *J*_C-F_ 280.0 Hz), 128.6, 129.2, 130.0, 131.1, 131.3, 132.1, 133.3 q (*J*_C-F_ 1.7 Hz), 136.3, 136.7, 136.9, 138.1, 143.4, 145.1, 147.3. ^19^F-NMR (CDCl_3_, 376 MHz, measured in a mixture with **4cc**) δ, ppm: –67.64 s (CF_3_). HRMS (MALDI-TOF): C_26_H_24_F_3_ found 393.1821 [M + H]^+^; calcd. 393.1825.

 **1-(4-Chlorophenyl)-4,6-dimethyl-3-phenyl-1-(trifluoromethyl)-*1H*-indene (4cd)** and **3-(2,4-dimethylphenyl)-1-(4-chlorophenyl)-1-(trifluoromethyl)-*1H*-indene (5cd):** The reaction scale is 30 mg (0.1 mmol) alcohol **1e** and 12 mg (0.11 mmol) *m*-xylene, isolated amount is 23 mg mixture of **4cd** and **5cd** (total yield 60%, ratio **4cd** : **5cd** =4:1, respectively). Compound **4cd**: ^1^Н-NMR (CDCl_3_, 400 MHz, measured in a mixture with **5cd**) δ, ppm: 1.96 s (3H, CH_3_), 2.38 s (3H, CH_3_), 6.32 s (1H, C^2^H), 6.95 s (1H, C^5^H), 7.23 s (1H, C^7^H), 7.30 d (2H_arom._, ^3^*J* 8.7 Hz), 7.34-7.43 m (5H_arom._), 7.47 d (2H_arom._, ^3^*J* 8.7 Hz). ^13^C-NMR (CDCl_3_, 100 MHz, measured in a mixture with **5cd**) δ, ppm: 20.1 (CH_3_), 21.5 (CH_3_), 63.2 q (C^1^, *J*_C-F_ 26.8 Hz), 122.1, 124.2 q (*J*_C-F_ 0.9 Hz), 126.65, 126.67 q (CF_3_, *J*_C-F_ 282.5 Hz), 128.0, 128.3, 128.6, 128.9, 129.3 q (*J*_C-F_ 1.9 Hz), 131.4, 132.7 q (*J*_C-F_ 1.7 Hz), 132.77, 132.83, 136.9, 137.3, 144.1, 149.7. ^19^F-NMR (CDCl_3_, 376 MHz, measured in a mixture with **5cd**) δ, ppm: –67.51 s (CF_3_). HRMS (MALDI-TOF): C_24_H_19_F_3_Cl found 399.1115 [M + H]^+^; calcd. 399.1122. Compound **5cd**: ^1^Н-NMR (CDCl_3_, 400 MHz, measured in a mixture with **4cd**) δ, ppm: 2.22 s (3H, CH_3_), 2.39 s (3H, CH_3_), 6.45 s (1H, C^2^H), 7.07-7.12 m (2H_arom._), 7.14 s (1H_arom._), 7.18 d (1H_arom._, ^3^*J* 7.7 Hz), 7.28-7.34 m (2H_arom._), 7.34-7.43 m (2H_arom._), 7.50 d (2H_arom._, ^3^*J* 8.7 Hz), 7.6 d (1H_arom._, ^3^*J* 7.1 Hz). ^13^C-NMR (CDCl_3_, 100 MHz, measured in a mixture with **4cd**) δ, ppm: 20.1 (CH_3_), 21.3 (CH_3_), 64.2 q (C^1^, *J*_C-F_ 26.8 Hz), 121.6, 125.4 q (*J*_C-F_ 0.5 Hz), 126.6 q (CF_3_, *J*_C-F_ 283.5 Hz), 126.7, 128.96, 129.0, 129.1, 130.8, 132.7 q (*J*_C-F_ 1.7 Hz), 133.4, 133.8, 134.1, 134.2, 136.2, 138.3, 139.1, 143.0 q (*J*_C-F_ 0.7 Hz), 145.0, 148.1. ^19^F-NMR (CDCl_3_, 376 MHz, measured in a mixture with **4cd**) δ, ppm: –67.65 s (CF_3_). HRMS (MALDI-TOF): C_24_H_19_F_3_Cl found 399.1115 [M + H]^+^; calcd. 399.1122.

 **1-(4-Bromophenyl)-4,6-dimethyl-3-phenyl-1-(trifluoromethyl)-*1H*-indene (4ce)** and **1-(4-bromophenyl)-3-(2,4-dimethylphenyl)-1-(trifluoromethyl)-*1H*-indene (5ce):** The reaction scale is 30 mg (0.09 mmol) alcohol **1f** and 11 mg (0.1 mmol) *m*-xylene, isolated amount is 21 mg mixture of **4ce** and **5ce** (total yield 56%, ratio **4ce** : **5ce** =4.9:1, respectively). Compound **4ce**:^1^Н-NMR (CDCl_3_, 400 MHz, measured in a mixture with **5ce**) δ, ppm: 1.96 s (3H, CH_3_), 2.38 s (3H, CH_3_), 6.32 s (1H, C^2^H), 6.95 s (1H, C^5^H), 7.23 s (1H, C^7^H), 7.35-7.42 m (7H_arom._), 7.45 d (2H_arom._, ^3^*J* 8.9 Hz). ^13^C-NMR (CDCl_3_, 100 MHz, measured in a mixture with **5ce**) δ, ppm: 20.1 (CH_3_), 21.5 (CH_3_), 63.3 q (C^1^, *J*_C-F_ 26.8 Hz), 122.2, 124.1 q (*J*_C-F_ 0.6 Hz), 126.6 q (CF_3_, *J*_C-F_ 282.5 Hz), 126.7, 128.0, 128.3, 128.6, 129.3, 129.6 q (*J*_C-F_ 1.9 Hz), 131.9, 132.8, 134.4, 136.9, 137.3, 139.1, 144.1 q (*J*_C-F_ 1.7 Hz), 149.8. ^19^F-NMR (CDCl_3_, 376 MHz, measured in a mixture with **5ce**) δ, ppm: –67.46 s (CF_3_). HRMS (MALDI-TOF): C_24_H_19_F_3_Br found 443.0622 [M + H]^+^; calcd. 443.0617. Compound **5ce**: ^1^Н-NMR (CDCl_3_, 400 MHz, measured in a mixture with **4ce**) δ, ppm: 2.22 s (3H, CH_3_), 2.39 s (3H, CH_3_), 6.44 s (1H, C^2^H), 7.08-7.12 m (2H_arom._), 7.14 s (1H_arom._), 7.18 d (1H_arom._, ^3^*J* 7.7 Hz), 7.32-7.42 m (2H_arom._), 7.43-7.48 m (4H_arom._), 7.61 d (1H_arom._, ^3^*J* 7.3 Hz). ^13^C-NMR (CDCl_3_, 100 MHz, measured in a mixture with **4ce**) δ, ppm: 20.1 (CH_3_), 21.5 (CH_3_), 63.2 q (C^1^, *J*_C-F_ 27.7 Hz), 114.7, 122.8, 124.1, 126.5 q (CF_3_, *J*_C-F_ 284.5 Hz), 126.7, 128.0, 128.5, 128.9, 129.2 q (*J*_C-F_ 1.2 Hz), 129.2, 129.6, 132.1, 132.7 q (*J*_C-F_ 1.7 Hz), 132.8, 132.8, 134.4, 139.1, 141.1, 149.8. ^19^F-NMR (CDCl_3_, 376 MHz, measured in a mixture with **4ce**) δ, ppm: –67.61 s (CF_3_). HRMS (MALDI-TOF): C_24_H_19_F_3_Br found 443.0622 [M + H]^+^; calcd. 443.0617.

**1-(2-Bromophenyl)-4,6-dimethyl-3-phenyl-1-(trifluoromethyl)-*1H*-indene (4cf):** Colorless solid, mp 177°С (МеОН-CH_2_Cl_2_). The reaction scale is 30 mg (0.09 mmol) alcohol **1g** and 11 mg (0.1 mmol) *m*-xylene, isolated amount is 15 mg **4cf** (yield 40%). ^1^Н-NMR (CDCl_3_, 400 MHz) δ, ppm: 1.96 s (3H, CH_3_), 2.31 s (3H, CH_3_), 6.10 s (1H, C^2^H), 6.91 s (1H, C^7^H), 6.94 s (1H, C^5^H), 7.16 td (1H_arom._, ^3^*J* 7.9 Hz, ^4^*J* 1.5 Hz), 7.36-7.46 m (6H_arom._), 7.53 dd (1H_arom._, ^3^*J* 7.9 Hz, ^4^*J* 1.5 Hz), 7.94-7.98 m (1H_arom._). ^13^C-NMR (CDCl_3_, 100 MHz) δ, ppm: 20.1 (CH_3_), 21.4 (CH_3_), 65.4 q (C^1^, *J*_C-F_ 25.0 Hz), 122.9, 123.5, 126.5 q (CF_3_, *J*_C-F_ 284.1 Hz), 127.5, 127.9, 128.2, 128.3, 129.1, 130.0, 130.7 q (*J*_C-F_ 4.2 Hz), 132.4, 132.6, 134.0, 136.3, 136.7, 137.8, 141.9, 144.6, 152.6. ^19^F-NMR (CDCl_3_, 376 MHz) δ, ppm: –65.41 s (CF_3_). HRMS (MALDI-TOF): C_24_H_19_F_3_Br found 443.0609 [M + H]^+^; calcd. 443.0617.

**1-(Trifluoromethyl)-4,6-dimethyl-1-(4-nitrophenyl)-3-phenyl-*1H*-indene (4cg):** Yellow oil. The reaction scale is 30 mg (0.09 mmol) alcohol **1h**, 35 mg (0.23 mmol) TfOH and 11 mg (0.1 mmol) *m*-xylene, isolated amount is 20 mg **4cg** (yield 54%). ^1^Н-NMR (CDCl_3_, 400 MHz) δ, ppm: 1.98 s (3H, CH_3_), 2.37 s (3H, CH_3_), 6.31 s (1H, C^2^H), 6.98 s (1H_arom._), 7.18 s (1H_arom._), 7.37-7.43 m (5H_arom._), 7.67 d (2H_arom._, ^3^*J* 8.9 Hz), 8.17 d (2H_arom._, ^3^*J* 8.9 Hz). ^13^C-NMR (CDCl_3_, 100 MHz) δ, ppm: 20.1 (CH_3_), 21.5 (CH_3_), 63.7 q (C^1^, *J*_C-F_ 27.1 Hz), 123.9, 124.1, 126.4 q (CF_3_, *J*_C-F_ 282.5 Hz), 128.3, 128.4, 128.5, 128.8 q (*J*_C-F_ 2.0 Hz), 131.9 q (*J*_C-F_ 1.4 Hz), 133.15, 133.22, 136.9, 137.4, 139.1, 142.9, 143.9, 147.6, 150.8. ^19^F-NMR (CDCl_3_, 376 MHz) δ, ppm: –66.95 s (CF_3_). HRMS (MALDI-TOF): C_24_H_19_F_3_NO_2_ found 410.1380 [M + H]^+^; calcd. 410.1363.

 **3-(4-Methylphenyl)-4,6-dimethyl-1-phenyl-1-(trifluoromethyl)-*1H*-indene (4ch)** and **3-(2,4-dimethylphenyl)–6-methyl-1-phenyl-1-(trifluoromethyl)-*1H*-indene (5cf):** The reaction scale is 30 mg (0.1 mmol) alcohol **1i** and 12 mg (0.11 mmol) *m*-xylene, isolated amount is 21 mg mixture of **4ch** and **5cf** (total yield 56%, ratio **4ch** : **5cf** =2.8:1, respectively). Compound **4ch**: ^1^Н-NMR (CDCl_3_, 400 MHz, measured in a mixture with **5cf**) δ, ppm: 1.99 s (3H, CH_3_), 2.38 (3H, CH_3_), 2.41 (3H, CH_3_), 6.35 s (1H, C^2^H), 6.94 s (1H, C^5^H), 7.21 d (2H_arom._, ^3^*J* 7.9 Hz), 7.27-7.29 m (3H_arom._), 7.30-7.37 m (3H_arom._), 7.54-7.56 m (2H_arom._). ^13^C-NMR (CDCl_3_, 100 MHz, measured in a mixture with **5cf**) δ, ppm: 20.2 (CH_3_), 21.4 (CH_3_), 21.5 (CH_3_), 63.6 q (C^1^, *J*_C-F_ 26.7 Hz), 124.3, 126.6, 126.9 q (CF_3_, *J*_C-F_ 282.6 Hz), 127.9 q (*J*_C-F_ 1.4 Hz), 128.0, 128.5, 128.7, 128.9, 132.55, 132.60, 133.1 q (*J*_C-F_ 1.3 Hz), 134.5, 135.3, 136.5, 137.7, 144.5, 149.3. ^19^F-NMR (CDCl_3_, 376 MHz, measured in a mixture with **5cf**) δ, ppm: –67.38 s (CF_3_). HRMS (MALDI-TOF): C_25_H_22_F_3_ found 379.1661 [M + H]^+^; calcd. 379.1669. Compound **5cf**: ^1^Н-NMR (CDCl_3_, 400 MHz, measured in a mixture with **4ch**) δ, ppm: 2.24 s (3H, CH_3_), 2.39 (3H, CH_3_), 2.42 (3H, CH_3_), 6.43 s (1H, C^2^H), 7.69 d (1H_arom._, ^3^*J* 7.8 Hz), 7.08 d (1H_arom._, ^3^*J* 7.6 Hz), 7.14 s (1H_arom._), 7.16 d (1H_arom._, ^3^*J* 7.8 Hz), 7.19-7.21 m (1H_arom._), 7.30-7.37 m (3H_arom._), 7.45 s (1H, C^7^H), 7.54-7.59 m (2H_arom._). ^13^C-NMR (CDCl_3_, 100 MHz, measured in a mixture with **4ch**) δ, ppm: 20.1 (CH_3_), 21.3 (CH_3_), 21.8 (CH_3_), 64.2 q (C^1^, *J*_C-F_ 26.6 Hz), 121.6, 126.3, 126.8 q (CF_3_, *J*_C-F_ 284.2 Hz), 127.9, 128.0, 128.8, 129.2, 129.4, 131.2, 131.3, 132.3, 135.1, 136.3, 136.5, 138.0, 139.3, 142.5, 143.7, 147.5. ^19^F-NMR (CDCl_3_, 376 MHz, measured in a mixture with **4ch**) δ, ppm: –67.41 s (CF_3_). HRMS (MALDI-TOF): C_25_H_22_F_3_ found 379.1661 [M + H]^+^; calcd. 379.1669.

**3-(4-Chlorophenyl)-4,6-dimethyl-1-phenyl-1-(trifluoromethyl)-*1H*-indene (4ci):** Colorless solid, mp 144°С (МеОН-CH_2_Cl_2_). The reaction scale is 30 mg (0.1 mmol) alcohol **1l** and 12 mg (0.11 mmol) *m*-xylene, isolated amount is 25 mg **4ci** (yield 63%). ^1^Н-NMR (CDCl_3_, 400 MHz) δ, ppm: 1.98 s (3H, CH_3_), 2.38 s (3H, CH_3_), 6.38 s (1H, C^2^H), 6.95 s (1H_arom._), 7.28 s (1H, C^7^H), 7.32 d (2H_arom._, ^3^*J* 8.5 Hz), 7.33-7.36 m (3H_arom._), 7.39 d (2H_arom._, ^3^*J* 8.5 Hz), 7.52-7.54 m (2H_arom._). ^13^C-NMR (CDCl_3_, 100 MHz) δ, ppm: 20.3 (CH_3_), 21.5 (CH_3_), 63.8 q (C^1^, *J*_C-F_ 26.6 Hz), 124.4 q (*J*_C-F_ 0.6 Hz), 126.8 q (CF_3_, *J*_C-F_ 282.6 Hz), 127.9 q (*J*_C-F_ 1.8 Hz), 128.1, 128.5, 128.8, 130.0, 132.4, 132.7, 133.7 q (*J*_C-F_ 1.7 Hz), 134.0, 134.9, 136.0, 136.9, 138.9, 144.4, 148.2. ^19^F-NMR (CDCl_3_, 376 MHz) δ, ppm: –67.38 s (CF_3_). HRMS (MALDI-TOF): C_24_H_19_F_3_Cl found 399.1138 [M + H]^+^; calcd. 399.1122.

**3-(4-Bromophenyl)-4,6-dimethyl-1-phenyl-1-(trifluoromethyl)-*1H*-indene (4cj):** Colorless solid, mp 152°С (МеОН-CH_2_Cl_2_). The reaction scale is 30 mg (0.09 mmol) alcohol **1m** and 11 mg (0.1 mmol) *m*-xylene, isolated amount is 23 mg **4cj** (yield 58%). ^1^Н-NMR (CDCl_3_, 400 MHz) δ, ppm: 1.98 s (3H, CH_3_), 2.38 s (3H, CH_3_), 6.38 s (1H, C^2^H), 6.95 s (1H, C^5^H), 7.26 d (2H_arom._, ^3^*J* 8.4 Hz), 7.28 s (1H, C^7^H), 7.31-7.37 m (3H_arom._), 7.51-7.55 m (2H_arom._), 7.54 d (2H_arom_._,_ ^3^*J* 8.4 Hz). ^13^C-NMR (CDCl_3_, 100 MHz) δ, ppm: 20.3 (CH_3_), 21.5 (CH_3_), 63.8 q (C^1^, *J*_C-F_ 26.7 Hz), 122.1, 124.4 q (*J*_C-F_ 0.5 Hz), 126.7 q (CF_3_, *J*_C-F_ 282.5 Hz), 127.9 q (*J*_C-F_ 1.8 Hz), 128.1, 128.8, 130.3, 131.4, 132.4, 132.7, 133.7 q (*J*_C-F_ 1.7 Hz), 134.9, 136.5, 136.9, 138.8, 144.4, 148.2. ^19^F-NMR (CDCl_3_, 376 MHz) δ, ppm: –67.38 s (CF_3_). HRMS (MALDI-TOF): C_24_H_19_F_3_Br found 443.0631 [M + H]^+^; calcd. 443.0617.

**1-(Trifluoromethyl)-3-(4-nitrophenyl)-4,6-dimethyl-1-phenyl-*1H*-indene (4ck):** Pale yellow oil. The reaction scale is 30 mg (0.09 mmol) alcohol **1n**, 35 mg (0.23 mmol) TfOH and 11 mg (0.1 mmol) *m*-xylene, isolated amount is 23 mg **4ck** (yield 63%). ^1^Н-NMR (CDCl_3_, 400 MHz) δ, ppm: 1.95 s (3H, CH_3_), 2.40 s (3H, CH_3_), 6.46 s (1H, C^2^H), 6.97 s (1H, C^5^H), 7.31 s (1H, C^7^H), 7.32-7.38 m (3H_arom._), 7.51-7.53 m (2H_arom._), 7.57 d (2H_arom._, ^3^*J* 8.7 Hz), 8.28 d (2H_arom._, ^3^*J* 8.7 Hz). ^13^C-NMR (CDCl_3_, 100 MHz) δ, ppm: 20.5(CH_3_), 21.5 (CH_3_), 64.1 q (C^1^, *J*_C-F_ 26.7 Hz), 123.6, 124.7, 126.6 q (CF_3_, *J*_C-F_ 282.6 Hz), 127.9 q (*J*_C-F_ 1.7 Hz), 128.3, 128.9, 129.6, 132.2, 132.8, 134.4, 134.6 q (*J*_C-F_ 1.6 Hz), 137.4, 138.3, 144.3, 144.5, 147.3, 147.8. ^19^F-NMR (CDCl_3_, 376 MHz) δ, ppm: –67.34 s (CF_3_). HRMS (MALDI-TOF): C_24_H_19_F_3_NO_2_ found 410.1380 [M + H]^+^; calcd. 410.1363.

**1,3-Bis(4-chlorophenyl)-4,6-dimethyl-1-(trifluoromethyl)-*1H*-indene (4cl):** Colorless solid, mp 152°С (МеОН-CH_2_Cl_2_). The reaction scale is 30 mg (0.09 mmol) alcohol **1q** and 11 mg (0.1 mmol) *m*-xylene, isolated amount is 25 mg **4cl** (yield 64%). ^1^Н-NMR (CDCl_3_, 400 MHz) δ, ppm: 1.97 s (3H, CH_3_), 2.38 s (3H, CH_3_), 6.32 s (1H, C^2^H), 6.96 s (1H_arom._), 7.23 s (1H_arom._), 7.28-7.32 m (4H_arom._), 7.39 d (2H_arom._, ^3^*J* 8.6 Hz), 7.45 d (2H_arom._, ^3^*J* 8.6 Hz). ^13^C-NMR (CDCl_3_, 100 MHz) δ, ppm: 20.3 (CH_3_), 21.5 (CH_3_), 63.3 q (C^1^, *J*_C-F_ 26.9 Hz), 124.3, 126.6 q (CF_3_, *J*_C-F_ 282.4 Hz), 128.5, 128.9, 129.3 q (*J*_C-F_ 1.5 Hz), 130.0, 132.6, 132.9, 133.2 q (*J*_C-F_ 0.7 Hz), 133.5, 134.1, 134.2, 135.7, 137.1, 138.8, 144.0, 148.6. ^19^F-NMR (CDCl_3_, 376 MHz) δ, ppm: –67.51 s (CF_3_). HRMS (MALDI-TOF): C_24_H_18_F_3_Cl_2_ found 433.0745 [M + H]^+^; calcd. 433.0733.

**1,3-Bis(4-bromophenyl)-4,6-dimethyl-1-(trifluoromethyl)-*1H*-indene (4cm):** Yellow oil. The reaction scale is 50 mg (0.12 mmol) alcohol **1r** and 14 mg (0.13 mmol) *m*-xylene, isolated amount is 32 mg **4cm** (yield 50%). ^1^Н-NMR (CDCl_3_, 400 MHz) δ, ppm: 1.97 s (3H, CH_3_), 2.37 s (3H, CH_3_), 6.30 s (1H, C^2^H), 6.95 s (1H_arom._), 7.21 s (1H_arom._), 7.24 d (2H_arom._, ^3^*J* 8.5 Hz), 7.37 d (2H_arom._, ^3^*J* 8.7 Hz), 7.45 d (2H_arom._, ^3^*J* 8.7 Hz), 7.54 d (2H_arom._, ^3^*J* 8.5 Hz). ^13^C-NMR (CDCl_3_, 100 MHz) δ, ppm: 20.3 (CH_3_), 21.5 (CH_3_), 63.4 q (C^1^, *J*_C-F_ 27.0 Hz), 122.2, 122.3, 124.3, 126.5 q (CF_3_, *J*_C-F_ 282.5 Hz), 129.6 q (*J*_C-F_ 1.8 Hz), 130.3, 131.5, 131.9, 132.6, 132.9, 133.1 q (*J*_C-F_ 1.5 Hz), 134.1, 136.2, 137.1, 138.7, 144.0 q (*J*_C-F_ 0.5 Hz), 148.6. ^19^F-NMR (CDCl_3_, 376 MHz) δ, ppm: –67.48 s (CF_3_). HRMS (MALDI-TOF): C_24_H_18_F_3_Br_2_ found 520.9725 [M + H]^+^; calcd. 520.9722.

**1,3-Diphenyl-4,6,7-trimethyl-1-(trifluoromethyl)-*1H*-indene (4da)** and **1,3-diphenyl-4,5,7-trimethyl-1-(trifluoromethyl)-*1H*-indene (4ea):** The reaction scale is 30 mg (0.1 mmol) alcohol **1a** and 13 mg (0.11 mmol) pseudocumene, isolated amount is 31 mg mixture of **4da** and **4ea** (total yield 75%, ratio **4da** : **4ea** = 11.5:1, respectively). Compound **4da**: ^1^Н-NMR (CDCl_3_, 400 MHz, measured in a mixture with **4ea**) δ, ppm: 1.94 s (6H, CH_3_), 2.24 (3H, CH_3_), 6.16 s (1H, C^2^H), 6.97 s (1H, C^5^H), 7.28-7.41 m (10H_arom._). ^13^C-NMR (CDCl_3_, 100 MHz, measured in a mixture with **4ea**) δ, ppm: 16.2 q (CH_3_, *J*_C-F_ 3.8 Hz), 20.0 (CH_3_), 20.1 (CH_3_), 65.3 q (C^1^, *J*_C-F_ 26.1 Hz), 126.9 q (*J*_C-F_ 1.9 Hz), 127.3 q (CF_3_, *J*_C-F_ 283.7 Hz), 127.5, 127.8, 128.2, 128.7, 128.9, 129.2, 132.2, 133.9, 135.1, 135.7 q (*J*_C-F_ 2.0 Hz), 136.6, 137.7, 141.0, 143.6, 148.1. ^19^F-NMR (CDCl_3_, 376 MHz, measured in a mixture with **4ea**) δ, ppm: –62.75 s (CF_3_). HRMS (MALDI-TOF): C_25_H_22_F_3_ found 379.1678 [M + H]^+^; calcd. 379.1669. Compound **4ea:** ^1^Н-NMR (CDCl_3_, 400 MHz, measured in a mixture with **4da**) δ, ppm: 1.88 s (3H, CH_3_), 2.01 s (3H, CH_3_), 2.26 s (3H, CH_3_), 6.22 s (1H, C^2^H), 6.90 s (1H, C^6^H), 7.28-7.41 m (10H_arom._). ^19^F-NMR (CDCl_3_, 376 MHz, measured in a mixture with **4da**) δ, ppm: –62.60 s (CF_3_). HRMS (MALDI-TOF): C_25_H_22_F_3_ found 379.1678 [M + H]^+^; calcd. 379.1669.

 **1-(3-Methylphenyl)-4,6,7-trimethyl-3-phenyl-1-(trifluoromethyl)-*1H*-indene (4db)** and **1-(3-methylphenyl)-4,5,7-trimethyl-3-phenyl-1-(trifluoromethyl)-*1H*-indene (4eb):** The reaction scale is 30 mg (0.1 mmol) alcohol **1b** and 13 mg (0.11 mmol) pseudocumene, isolated amount is 27 mg mixture of **4db** and **4eb** (total yield 69%, ratio **4db** : **4eb** = 11.5:1, respectively). Compound **4db**: ^1^Н-NMR (CDCl_3_, 400 MHz, measured in a mixture with **4eb**) δ, ppm: 1.95 s (3H, CH_3_), 1.97 s (3H, CH_3_), 2.25 s (3H, CH_3_), 2.34 s (3H, CH_3_), 6.15 s (1H, C^2^H), 6.97 s (1H, C^5^H), 7.09-7.12 m (2H_arom._), 7.17 s (1H_arom._), 7.17-7.21 m (1H_arom._), 7.34-7.42 m (5H_arom._). ^13^C-NMR (CDCl_3_, 100 MHz, measured in a mixture with **4eb**) δ, ppm: 16.2 q (CH_3_, *J*_C-F_ 3.8 Hz), 20.0 (CH_3_), 20.2 (CH_3_), 21.8 (CH_3_), 65.3 q (C^1^, *J*_C-F_ 26.0 Hz), 124.0 q (*J*_C-F_ 2.0 Hz), 127.4 q (CF_3_, *J*_C-F_ 283.8 Hz), 127.5 q (*J*_C-F_ 2.0 Hz), 127.7, 128.1, 128.3, 128.67, 128.73, 129.4, 133.2, 133.9, 134.9, 135.8 q (*J*_C-F_ 2.1 Hz), 136.5, 137.8, 138.5, 141.0, 143.6, 148.0. ^19^F-NMR (CDCl_3_, 376 MHz, measured in a mixture with **4eb**) δ, ppm: –62.64 s (CF_3_). HRMS (MALDI-TOF): C_26_H_24_F_3_ found 393.1809 [M + H]^+^; calcd. 393.1825. Compound **4eb:** ^1^Н-NMR (CDCl_3_, 400 MHz, measured in a mixture with **4db**) δ, ppm: 1.89 s (3H, CH_3_), 2.04 s (3H, CH_3_), 2.26 s (3H, CH_3_), 2.33 s (3H, CH_3_), 6.21 s (1H, C^2^H), 6.91 s (1H, C^6^H), 7.09-7.12 m (2H_arom._), 7.16-7.21 m (2H_arom._), 7.34-7.42 m (5H_arom._). ^19^F-NMR (CDCl_3_, 376 MHz, measured in a mixture with **4db**) δ, ppm: –62.47 s (CF_3_). HRMS (MALDI-TOF): C_25_H_24_F_3_ found 393.1809 [M + H]^+^; calcd. 393.1825.

 **1-(4-Methylphenyl)-4,6,7-trimethyl-3-phenyl-1-(trifluoromethyl)-*1H*-indene (4dc) and 1-(4-methylphenyl)-4,5,7-trimethyl-3-phenyl-1-(trifluoromethyl)-*1H*-indene (4ec):** The reaction scale is 30 mg (0.1 mmol) alcohol **1c** and 13 mg (0.11 mmol) pseudocumene, isolated amount is 20 mg mixture of **4dc** and **4ec** (total yield 50%, ratio **4dc** : **4ec** = 13:1, respectively). Compound **4dc**: ^1^Н-NMR (CDCl_3_, 400 MHz, measured in a mixture with **4ec**) δ, ppm: 1.92 s (3H, CH_3_), 1.95 s (3H, CH_3_), 2.23 s (3H, CH_3_), 2.33 s (3H, CH_3_), 6.13 s (1H, C^2^H), 6.95 s (1H, C^5^H), 7.10 d (2H_arom._, ^3^*J* 8.1 Hz), 7.21 d (2H_arom._, ^3^*J* 8.1 Hz), 7.32-7.40 m (5H_arom._). ^13^C-NMR (CDCl_3_, 100 MHz, measured in a mixture with **4ec**) δ, ppm: 16.2 q (CH_3_, *J*_C-F_ 3.7 Hz), 20.0 (CH_3_), 20.2 (CH_3_), 21.2 (CH_3_), 65.0 q (C^1^, *J*_C-F_ 26.2 Hz), 126.8 q (*J*_C-F_ 1.7 Hz), 127.4 q (CF_3_, *J*_C-F_ 283.7 Hz), 127.7, 128.1, 128.7, 129.4, 129.6, 131.9, 132.2, 133.9, 135.9 q (*J*_C-F_ 2.0 Hz), 136.5, 137.2, 137.8, 141.0, 143.6, 147.8. ^19^F-NMR (CDCl_3_, 376 MHz, measured in a mixture with **4ec**) δ, ppm: –62.67 s (CF_3_). HRMS (MALDI-TOF): C_26_H_24_F_3_ found 393.1811 [M + H]^+^; calcd. 393.1825. Compound **4ec**: ^1^Н-NMR (CDCl_3_, 400 MHz, measured in a mixture with **4dc**) δ, ppm: 1.87 s (3H, CH_3_), 2.02 s (3H, CH_3_), 2.24 s (3H, CH_3_), 2.33 s (3H, CH_3_), 6.18 s (1H, C^2^H), 6.89 s (1H, C^6^H), 7.09-7.11 m (2H_arom._), 7.20-7.22 m (2H_arom._), 7.32-7.40 m (5H_arom._). ^19^F-NMR (CDCl_3_, 376 MHz, measured in a mixture with **4dc**) δ, ppm: –62.84 s (CF_3_). HRMS (MALDI-TOF): C_26_H_24_F_3_ found 393.1811 [M + H]^+^; calcd. 393.1825.

 **1-(3,4-Dimethylphenyl)-4,6,7-trimethyl-3-phenyl-1-(trifluoromethyl)-*1H*-indene (4dd)** and **1-(3,4-dimethylphenyl)-4,5,7-trimethyl-3-phenyl-1-(trifluoromethyl)-*1H*-indene (4ed):** The reaction scale is 30 mg (0.1 mmol) alcohol **1d** and 13 mg (0.11 mmol) pseudocumene, isolated amount is 27 mg mixture of **4dd** and **4ed** (total yield 67%, ratio **4dd** : **4ed** = 15.7:1, respectively). Compound **4dd**: ^1^Н-NMR (CDCl_3_, 400 MHz, measured in a mixture with **4ed**) δ, ppm: 1.94 s (3H, CH_3_), 1.98 s (3H, CH_3_), 2.24 s (3H, CH_3_), 2.25 s (6H, CH_3_), 6.14 s (1H, C^2^H), 6.96 s (1H, C^5^H), 7.03-7.08 m (2H_arom._), 7.11 s (1H_arom._), 7.33-7.41 m (5H_arom._). ^13^C-NMR (CDCl_3_, 100 MHz, measured in a mixture with **4ed**) δ, ppm: 16.3 q (CH_3_, *J*_C-F_ 3.8 Hz), 19.5 (CH_3_), 20.0 (CH_3_), 20.1 (CH_3_), 20.2 (CH_3_), 65.1 q (C^1^, *J*_C-F_ 26.0 Hz), 124.4 q (*J*_C-F_ 1.9 Hz), 127.4 q (CF_3_, *J*_C-F_ 283.8 Hz), 127.7, 128.0 q (*J*_C-F_ 1.9 Hz), 128.1, 128.7, 129.4, 130.1, 132.16, 132.24, 133.8, 135.92, 135.93 q (*J*_C-F_ 2.5 Hz), 136.4, 137.0, 137.9, 141.0, 143.5 q (*J*_C-F_ 1.0 Hz), 147.7. ^19^F-NMR (CDCl_3_, 376 MHz, measured in a mixture with **4ed**) δ, ppm: –62.69 s (CF_3_). HRMS (MALDI-TOF): C_27_H_26_F_3_ found 407.1987 [M + H]^+^; calcd. 407.1981. Compound **4ed**: ^1^Н-NMR (CDCl_3_, 400 MHz, measured in a mixture with **4dd**) δ, ppm: 1.88 s (3H, CH_3_), 2.05 s (3H, CH_3_), 2.24 s (3H, CH_3_), 2.25 s (6H, CH_3_), 6.20 s (1H, C^2^H), 6.90 s (1H, C^6^H), 7.03-7.08 m (2H_arom._), 7.11 s (1H_arom._), 7.33-7.41 m (5H_arom._). ^19^F-NMR (CDCl_3_, 376 MHz, measured in a mixture with **4dd**) δ, ppm: –62.52 s (CF_3_). HRMS (MALDI-TOF): C_27_H_26_F_3_ found 407.1987 [M + H]^+^; calcd. 407.1981.

 **1-(4-Chlorophenyl)-4,6,7-trimethyl-3-phenyl-1-(trifluoromethyl)-*1H*-indene (4de)** and **1-(4-chlorophenyl)-4,5,7-trimethyl-3-phenyl-1-(trifluoromethyl)-*1H*-indene (4ee):** The reaction scale is 30 mg (0.1 mmol) alcohol **1e** and 13 mg (0.11 mmol) pseudocumene, isolated amount is 27 mg mixture of **4de** and **4ee** (total yield 66%, ratio **4de** : **4ee** = 11.5:1, respectively). Compound **4de**: ^1^Н-NMR (CDCl_3_, 400 MHz, measured in a mixture with **4ee**) δ, ppm: 1.92 s (3H, CH_3_), 1.93 s (3H, CH_3_), 2.24 s (3H, CH_3_), 6.11 s (1H, C^2^H), 6.97 s (1H, C^5^H), 7.22-7.29 m (4H_arom._), 7.33-7.39 m (5H_arom._). ^13^C-NMR (CDCl_3_, 100 MHz, measured in a mixture with **4ee**) δ, ppm: 16.2 q (CH_3_, *J*_C-F_ 3.8 Hz), 19.9 (CH_3_), 20.1 (CH_3_), 64.9 q (C^1^, *J*_C-F_ 26.5 Hz), 127.2 q (CF_3_, *J*_C-F_ 283.7 Hz), 127.9, 128.2, 128.4 q (*J*_C-F_ 2.1 Hz), 128.6, 129.1, 129.7, 132.1, 133.4, 133.8, 134.1, 135.2 q (*J*_C-F_ 2.0 Hz), 136.8, 137.5, 140.8, 143.2 q (*J*_C-F_ 0.9 Hz), 148.5. ^19^F-NMR (CDCl_3_, 376 MHz, measured in a mixture with **4ee**) δ, ppm: –62.95 s (CF_3_). HRMS (MALDI-TOF): C_25_H_21_F_3_Cl found 413.1280 [M + H]^+^; calcd. 413.1279. Compound **4ee**: ^1^Н-NMR (CDCl_3_, 400 MHz, measured in a mixture with **4de**) δ, ppm: 1.87 s (3H, CH_3_), 2.01 s (3H, CH_3_), 2.29 s (3H, CH_3_), 6.16 s (1H, C^2^H), 6.90 s (1H, C^6^H), 7.22-7.29 m (4H_arom._), 7.33-7.39 m (5H_arom._). ^19^F-NMR (CDCl_3_, 376 MHz, measured in a mixture with **4de**) δ, ppm: –62.79 s (CF_3_). HRMS (MALDI-TOF): C_25_H_21_F_3_Cl found 413.1280 [M + H]^+^; calcd. 413.1279.

 **1-(4-Bromophenyl)-4,6,7-trimethyl-3-phenyl-1-(trifluoromethyl)-*1H*-indene (4df)** and **1-(4-bromophenyl)-4,5,7-trimethyl-3-phenyl-1-(trifluoromethyl)-*1H*-indene (4ef):** The reaction scale is 30 mg (0.09 mmol) alcohol **1f** and 12 mg (0.10 mmol) pseudocumene, isolated amount is 31 mg mixture of **4df** and **4ef** (total yield 80%, ratio **4df** : **4ef** = 24:1, respectively). Compound **4df**: ^1^Н-NMR (CDCl_3_, 400 MHz, measured in a mixture with **4ef**) δ, ppm: 1.92 s (3H, CH_3_), 1.94 s (3H, CH_3_), 2.24 s (3H, CH_3_), 6.10 s (1H, C^2^H), 6.97 s (1H_arom._), 7.19 d (2H_arom._, ^3^*J* 8.5 Hz), 7.33-7.44 m (5H_arom._), 7.42 d (2H_arom._, ^3^*J* 8.5 Hz). ^13^C-NMR (CDCl_3_, 100 MHz, measured in a mixture with **4ef**) δ, ppm: 16.2 q (CH_3_, *J*_C-F_ 3.8 Hz), 19.9 (CH_3_), 20.1 (CH_3_), 64.9 q (C^1^, *J*_C-F_ 26.4 Hz), 121.5, 127.1 q (CF_3_, *J*_C-F_ 283.6 Hz), 127.9, 128.2, 128.6, 128.8 q (*J*_C-F_ 1.8 Hz), 129.7, 132.0, 132.1, 134.2, 134.4, 135.1 q (*J*_C-F_ 1.8 Hz), 136.8, 137.5, 140.8, 143.1, 148.5. ^19^F-NMR (CDCl_3_, 376 MHz, measured in a mixture with **4ef**) δ, ppm: –62.93 s (CF_3_). HRMS (MALDI-TOF): C_25_H_21_F_3_Br found 457.0769 [M + H]^+^; calcd. 457.0774. Compound **4ef**: ^1^Н-NMR (CDCl_3_, 400 MHz, measured in a mixture with **4df**) δ, ppm: 1.87 s (3H, CH_3_), 2.01 s (3H, CH_3_), 2.24 s (3H, CH_3_), 6.16 s (1H, C^2^H), 6.90 s (1H_arom._), 7.18-7.20 m (2H_arom._), 7.33-7.44 m (7H_arom._). ^19^F-NMR (CDCl_3_, 376 MHz, measured in a mixture with **4df**) δ, ppm: –62.77 s (CF_3_). HRMS (MALDI-TOF): C_25_H_21_F_3_Br found 457.0769 [M + H]^+^; calcd. 457.0774.

 **1-(2-Bromophenyl)-4,6,7-trimethyl-3-phenyl-1-(trifluoromethyl)-*1H*-indene (4dg)** and **1-(2-bromophenyl)-4,5,7-trimethyl-3-phenyl-1-(trifluoromethyl)-*1H*-indene (4eg):** The reaction scale is 30 mg (0.09 mmol) alcohol **1g** and 12 mg (0.10 mmol) pseudocumene, isolated amount is 27 mg mixture of **4dg** and **4eg** (total yield 70%, ratio **4dg** : **4eg** = 2.7:1, respectively). Compound **4dg**: ^1^Н-NMR (CDCl_3_, 400 MHz, measured in a mixture with **4eg**) δ, ppm: 1.82 s (3H, CH_3_), 1.90 s (3H, CH_3_), 2.22 s (3H, CH_3_), 5.97 s (1H, C^2^H), 6.94 s (1H_arom._), 7.16 td (1H_arom._, ^3^*J* 7.9 Hz, ^4^*J* 1.4 Hz), 7.35-7.45 m (6H_arom._), 7.53 dd (1H_arom._, ^3^*J* 7.9 Hz, ^4^*J* 1.4 Hz), 7.95-7.98 m (1H_arom._). ^13^C-NMR (CDCl_3_, 100 MHz, measured in a mixture with **4eg**) δ, ppm: 15.4 q (CH_3_, *J*_C-F_ 3.0 Hz), 19.8 (CH_3_), 20.1 (CH_3_), 66.1 q (C^1^, *J*_C-F_ 24.9 Hz), 121.9 q (*J*_C-F_ 0.7 Hz), 127.1 q (CF_3_, *J*_C-F_ 285.2 Hz), 127.80, 127.81, 128.1, 128.2, 128.3, 128.9, 129.9, 130.3 q (*J*_C-F_ 4.4 Hz), 130.4 q (*J*_C-F_ 1.3 Hz), 131.0, 131.9, 133.8, 136.0, 136.1, 136.2, 138.1, 152.2. ^19^F-NMR (DMSO-d_6_, 376 MHz, 100 °C, measured in a mixture with **4eg**) δ, ppm: –61.78 - –62.31 m (CF_3_). HRMS (MALDI-TOF): C_25_H_21_F_3_Br found 457.0789 [M + H]^+^; calcd. 457.0774. Compound **4eg**: ^1^Н-NMR (CDCl_3_, 400 MHz, measured in a mixture with **4dg**) δ, ppm: 1.85 s (3H, CH_3_), 1.86 s (3H, CH_3_), 2.24 s (3H, CH_3_), 6.03 s (1H, C^2^H), 6.84 s (1H_arom._), 7.13-7.18 m (1H_arom._), 7.35-7.45 m (6H_arom._), 7.51-7.54 m (1H_arom._), 7.94-7.98 m (1H_arom._). ^13^C-NMR (CDCl_3_, 100 MHz, measured in a mixture with **4dg**) δ, ppm: 15.9 (CH_3_), 18.4 q (CH_3_, *J*_C-F_ 3.0 Hz), 20.3 (CH_3_), 65.5 q (C^1^, *J*_C-F_ 25.1 Hz), 122.0 q (*J*_C-F_ 1.0 Hz), 127.1 q (CF_3_, *J*_C-F_ 285.1 Hz), 127.7, 127.8, 128.2, 128.9, 129.6, 130.5 q (*J*_C-F_ 4.4 Hz), 131.8 q (*J*_C-F_ 1.1 Hz), 132.2, 133.6, 134.2, 138.6, 138.8, 139.2, 141.9, 142.7 q (*J*_C-F_ 0.5 Hz), 145.1, 152.6. ^19^F-NMR (DMSO-d_6_, 376 MHz, 100 °C, measured in a mixture with **4dg**) δ, ppm: –61.78 - –62.31 m (CF_3_). HRMS (MALDI-TOF): C_25_H_21_F_3_Br found 457.0789 [M + H]^+^; calcd. 457.0774.

 **1-(4-Nitrophenyl)-4,6,7-trimethyl-3-phenyl-1-(trifluoromethyl)-*1H*-indene (4dh)** and **1-(4-nitrophenyl)-4,5,7-trimethyl-3-phenyl-1-(trifluoromethyl)-*1H*-indene (4eh):** The reaction scale is 30 mg (0.09 mmol) alcohol **1h**, 35 mg (0.23 mmol) TfOH and 12 mg (0.10 mmol) pseudocumene, isolated amount is 22 mg mixture of **4dh** and **4eh** (total yield 58%, ratio **4dh** : **4eh** = 1.8:1, respectively). Compound **4dh**: ^1^Н-NMR (CDCl_3_, 400 MHz, measured in a mixture with **4eh**) δ, ppm: 1.89 s (3H, CH_3_), 1.94 s (3H, CH_3_), 2.24 s (3H, CH_3_), 6.10 s (1H, C^2^H), 7.00 s (1H_arom._), 7.33-7.42 m (5H_arom._), 7.47-7.50 m (2H_arom._), 8.16 d (2H_arom._, ^3^*J* 9.1 Hz). ^13^C-NMR (CDCl_3_, 100 MHz, measured in a mixture with **4eh**) δ, ppm: 16.3 q (CH_3_, *J*_C-F_ 3.6 Hz), 19.9 (CH_3_), 20.1 (CH_3_), 65.2 q (C^1^, *J*_C-F_ 26.9 Hz), 124.1, 125.5 q (CF_3_, *J*_C-F_ 272.8 Hz), 128.0 q (*J*_C-F_ 2.0 Hz), 128.1, 128.3, 128.4, 128.5, 130.2, 132.0, 134.2 q (*J*_C-F_ 1.8 Hz), 137.1, 137.2, 139.6, 140.7, 143.3, 147.3, 149.7. ^19^F-NMR (CDCl_3_, 376 MHz, measured in a mixture with **4eh**) δ, ppm: –62.87 s (CF_3_). HRMS (MALDI-TOF): C_25_H_21_F_3_NO_2_ found 424.1528 [M + H]^+^; calcd. 424.1519. Compound **4eh**: ^1^Н-NMR (CDCl_3_, 400 MHz, measured in a mixture with **4dh**) δ, ppm: 1.88 s (3H, CH_3_), 1.96 s (3H, CH_3_), 2.26 s (3H, CH_3_), 6.16 s (1H, C^2^H), 6.92 s (1H_arom._), 7.33-7.42 m (5H_arom._), 7.47-7.50 m (2H_arom._), 8.14 d (2H_arom._, ^3^*J* 9.1 Hz). ^13^C-NMR (CDCl_3_, 100 MHz, measured in a mixture with **4dh**) δ, ppm: 16.0 (CH_3_), 19.2 q (CH_3_, *J*_C-F_ 3.6 Hz), 20.2 (CH_3_), 64.6 q (C^1^, *J*_C-F_ 27.2 Hz), 124.0, 126.5 q (CF_3_, *J*_C-F_ 275.9 Hz), 128.1, 129.0, 129.4, 129.8, 130.0, 132.0, 132.3, 134.6, 135.4 q (*J*_C-F_ 1.9 Hz), 137.6, 140.2, 142.8, 142.9, 143.0, 150.2. ^19^F-NMR (CDCl_3_, 376 MHz, measured in a mixture with **4dh**) δ, ppm: –62.70 s (CF_3_). HRMS (MALDI-TOF): C_25_H_21_F_3_NO_2_ found 424.1528 [M + H]^+^; calcd. 424.1519.

 **3-(4-Methylphenyl)-4,6,7-trimethyl-1-phenyl-1-(trifluoromethyl)-*1H*-indene (4di)** and **3-(4-methylphenyl)-4,5,7-trimethyl-1-phenyl-1-(trifluoromethyl)-*1H*-indene (4ei):** The reaction scale is 30 mg (0.1 mmol) alcohol **1i** and 14 mg (0.11 mmol) pseudocumene, isolated amount is 20 mg mixture of **4di** and **4ei** (total yield 51%, ratio **4di** : **4ei** = 12:1, respectively). Compound **4di**: ^1^Н-NMR (CDCl_3_, 400 MHz, measured in a mixture with **4ei**) δ, ppm: 1.94 s (3H, CH_3_), 1.96 (3H, CH_3_), 2.24 (3H, CH_3_), 2.40 (3H, CH_3_), 6.13 s (1H, C^2^H), 6.96 s (1H, C^5^H), 7.19 d (2H_arom._, ^3^*J* 7.9 Hz), 7.25 d (2H_arom._, ^3^*J* 7.9 Hz), 7.28-7.33 m (5H_arom._). ^13^C-NMR (CDCl_3_, 100 MHz, measured in a mixture with **4ei**) δ, ppm: 16.1 q (CH_3_, *J*_C-F_ 3.7 Hz), 20.0 (CH_3_), 20.1 (CH_3_), 21.4 (CH_3_), 65.2 q (C^1^, *J*_C-F_ 25.8 Hz), 126.9 q (*J*_C-F_ 1.8 Hz), 127.4 q (CF_3_, *J*_C-F_ 283.9 Hz), 127.5, 128.5, 128.8, 128.9, 129.5, 132.1, 133.9, 134.7, 135.2, 135.6 q (*J*_C-F_ 1.9 Hz), 136.5, 137.5, 141.1, 143.6, 148.1. ^19^F-NMR (CDCl_3_, 376 MHz, measured in a mixture with **4ei**) δ, ppm: –62.78 s (CF_3_). HRMS (MALDI-TOF): C_26_H_24_F_3_ found 393.1829 [M + H]^+^; calcd. 393.1825. Compound **4ei**: ^1^Н-NMR (CDCl_3_, 400 MHz, , measured in a mixture with **4di**) δ, ppm: 1.90 s (3H, CH_3_), 2.00 (3H, CH_3_), 2.25 (3H, CH_3_), 2.40 (3H, CH_3_), 6.19 s (1H, C^2^H), 6.89 s (1H, C^6^H), 7.18-7.20 m (2H_arom._), 7.24-7.26 m (2H_arom._), 7.26-7.33 m (5H_arom._). ^19^F-NMR (CDCl_3_, 376 MHz, , measured in a mixture with **4di**) δ, ppm: –62.62 s (CF_3_). HRMS (MALDI-TOF): C_26_H_24_F_3_ found 393.1829 [M + H]^+^; calcd. 393.1825.

 **3-(4-Chlorophenyl)-4,6,7-trimethyl-1-phenyl-1-(trifluoromethyl)-*1H*-indene (4dj) and 3-(4-chlorophenyl)-4,5,7-trimethyl-1-phenyl-1-(trifluoromethyl)-*1H*-indene (4ej):** The reaction scale is 30 mg (0.1 mmol) alcohol **1l** and 14 mg (0.11 mmol) pseudocumene, isolated amount is 22 mg mixture of **4dj** and **4ej** (total yield 54%, ratio **4dj** : **4ej** = 11.5:1, respectively). Compound **4dj**: ^1^Н-NMR (CDCl_3_, 400 MHz, measured in a mixture with **4ej**) δ, ppm: 1.92 s (3H, CH_3_), 1.93 s (3H, CH_3_), 2.23 s (3H, CH_3_), 6.14 s (1H, C^2^H), 6.96 s (1H, C^5^H), 7.28-7.30 m (7H_arom._), 7.36 d (2H_arom._, ^3^*J* 8.6 Hz). ^13^C-NMR (CDCl_3_, 100 MHz, measured in a mixture with **4ej**) δ, ppm: 16.2 q (CH_3_, *J*_C-F_ 3.6 Hz), 20.1 (CH_3_), 20.2 (CH_3_), 65.4 q (C^1^, *J*_C-F_ 26.3 Hz), 126.9 q (*J*_C-F_ 1.8 Hz), 127.2 q (CF_3_, *J*_C-F_ 283.8 Hz), 127.6, 128.4, 128.9, 129.4, 130.0, 132.3, 133.8, 134.0, 134.8, 136.14 q (*J*_C-F_ 2.2 Hz), 136.16, 136.9, 140.6, 143.5, 147.0. ^19^F-NMR (CDCl_3_, 376 MHz, measured in a mixture with **4ej**) δ, ppm: –62.75 s (CF_3_). HRMS (MALDI-TOF): C_25_H_21_F_3_Cl found 413.1288 [M + H]^+^; calcd. 413.1279. Compound **4ej**: ^1^Н-NMR (CDCl_3_, 400 MHz, measured in a mixture with **4dj**) δ, ppm: 1.88 s (3H, CH_3_), 1.99 (3H, CH_3_), 2.25 (3H, CH_3_), 6.20 s (1H, C^2^H), 6.89 s (1H, C^6^H), 7.28-7.30 m (7H_arom._), 7.35-7.37 m (2H_arom._). ^19^F-NMR (CDCl_3_, 376 MHz, measured in a mixture with **4dj**) δ, ppm: –62.59 s (CF_3_). HRMS (MALDI-TOF): C_25_H_21_F_3_Cl found 413.1288 [M + H]^+^; calcd. 413.1279.

 **3-(4-Bromophenyl)-4,6,7-trimethyl-1-phenyl-1-(trifluoromethyl)-*1H*-indene (4dk)** and **3-(4-bromophenyl)-4,5,7-trimethyl-1-phenyl-1-(trifluoromethyl)-*1H*-indene (4ek):** The reaction scale is 30 mg (0.09 mmol) alcohol **1m** and 12 mg (0.1 mmol) pseudocumene, isolated amount is 20 mg mixture of **4dk** and **4ek** (total yield 49%, ratio **4dk** : **4ek** = 10:1, respectively). Compound **4dk**: ^1^Н-NMR (CDCl_3_, 400 MHz, measured in a mixture with **4ek**) δ, ppm: 1.93 s (3H, CH_3_), 1.94 s (3H, CH_3_), 2.24 s (3H, CH_3_), 6.15 s (1H, C^2^H), 6.96 s (1H, C^5^H), 7.24 d (2H_arom._, ^3^*J* 8.4 Hz), 7.27-7.31 m (5H_arom._), 7.52 d (2H_arom._, ^3^*J* 8.4 Hz). ^13^C-NMR (CDCl_3_, 100 MHz, measured in a mixture with **4ek**) δ, ppm: 16.2 q (CH_3_, *J*_C-F_ 3.8 Hz), 20.1 (CH_3_), 20.2 (CH_3_), 65.4 q (C^1^, *J*_C-F_ 26.2 Hz), 121.9, 126.9 q (*J*_C-F_ 1.9 Hz), 127.2 q (CF_3_, *J*_C-F_ 283.8 Hz), 127.6, 128.9, 129.4, 130.4, 131.4, 132.3, 134.0, 134.8, 136.1 q (*J*_C-F_ 2.0 Hz), 136.7, 136.9, 140.5, 143.5, 147.0. ^19^F-NMR (CDCl_3_, 376 MHz, measured in a mixture with **4ek**) δ, ppm: –62.73 s (CF_3_). HRMS (MALDI-TOF): C_25_H_21_F_3_Br found 457.0778 [M + H]^+^; calcd. 457.0774. Compound **4ek**: ^1^Н-NMR (CDCl_3_, 400 MHz, measured in a mixture with **4dk**) δ, ppm: 1.89 s (3H, CH_3_), 2.00 s (3H, CH_3_), 2.25 s (3H, CH_3_), 6.21 s (1H, C^2^H), 6.90 s (1H, C^6^H), 7.23-7.25 m (2H_arom._), 7.27-7.31 m (5H_arom._), 7.51-7.53 m (2H_arom._). ^19^F-NMR (CDCl_3_, 376 MHz, measured in a mixture with **4dk**) δ, ppm: –62.57 s (CF_3_). HRMS (MALDI-TOF): C_25_H_21_F_3_Br found 457.0778 [M + H]^+^; calcd. 457.0774.

 **3-(4-Nitrophenyl)-1-phenyl-4,6,7-trimethyl-1-(trifluoromethyl)-*1H*-indene (4dl)** and **3-(4-nitrophenyl)-1-phenyl-4,5,7-trimethyl-1-(trifluoromethyl)-*1H*-indene (4el):** The reaction scale is 30 mg (0.09 mmol) alcohol **1n**, 35 mg (0.23 mmol) TfOH and 12 mg (0.10 mmol) pseudocumene, isolated amount is 25 mg mixture of **4dl** and **4el** (total yield 66%, ratio **4dl** : **4el** = 13:1, respectively). Compound **4dl**: ^1^Н-NMR (CDCl_3_, 400 MHz, measured in a mixture with **4el**) δ, ppm: 1.92 s (3H, CH_3_), 1.94 s (3H, CH_3_), 2.25 s (3H, CH_3_), 6.22 s (1H, C^2^H), 6.98 s (1H, C^5^H), 7.28-7.33 m (5H_arom._), 7.54 d (2H_arom._, ^3^*J* 8.8 Hz), 8.26 d (2H_arom._, ^3^*J* 8.8 Hz). ^13^C-NMR (CDCl_3_, 100 MHz, measured in a mixture with **4el**) δ, ppm: 16.2 q (CH_3_, *J*_C-F_ 3.8 Hz), 20.2 (CH_3_), 20.3 (CH_3_), 65.7 q (C^1^, *J*_C-F_ 26.4 Hz), 123.5, 126.9 q (*J*_C-F_ 1.9 Hz), 127.1 q (CF_3_, *J*_C-F_ 283.7 Hz), 127.8, 129.1, 129.2, 129.6, 132.6, 134.0, 134.3, 136.9 q (*J*_C-F_ 2.1 Hz), 137.4, 140.0, 143.4 q (*J*_C-F_ 0.8 Hz), 144.7, 146.2, 147.7. ^19^F-NMR (CDCl_3_, 376 MHz, measured in a mixture with **4el**) δ, ppm: –62.64 s (CF_3_). HRMS (MALDI-TOF): C_25_H_21_F_3_NO_2_ found 424.1525 [M + H]^+^; calcd. 424.1519. Compound **4el**: ^1^Н-NMR (CDCl_3_, 400 MHz, measured in a mixture with **4dl**) δ, ppm: 1.86 s (3H, CH_3_), 2.01 s (3H, CH_3_), 2.26 s (3H, CH_3_), 6.28 s (1H, C^2^H), 6.93 s (1H, C^6^H), 7.28-7.33 m (5H_arom._), 7.53-7.55 m (2H_arom._), 8.25−8.27 m (2H_arom._). ^19^F-NMR (CDCl_3_, 376 MHz, measured in a mixture with **4dl**) δ, ppm: –62.48 s (CF_3_). HRMS (MALDI-TOF): C_25_H_21_F_3_NO_2_ found 424.1525 [M + H]^+^; calcd. 424.1519.

 **1,3-Di(4-methylphenyl)-4,6,7-trimethyl-1-(trifluoromethyl)-*1H*-indene (4dm)** and **1,3-di(4-methylphenyl)-4,5,7-trimethyl-1-(trifluoromethyl)-*1H*-indene (4em):** The reaction scale is 30 mg (0.1 mmol) alcohol **1o** and 14 mg (0.11 mmol) pseudocumene, isolated amount is 26 mg mixture of **4dm** and **4em** (total yield 65%, ratio **4dm** : **4em** = 6:1, respectively). Compound **4dm**: ^1^Н-NMR (CDCl_3_, 400 MHz, measured in a mixture with **4em**) δ, ppm: 1.95 s (6H, CH_3_), 2.24 s (3H, CH_3_), 2.33 s (3H, CH_3_), 2.39 s (3H, CH_3_), 6.11 s (1H, C^2^H), 6.95 s (1H, C^5^H), 7.10 d (2H_arom._, ^3^*J* 8.1 Hz), 7.17-7.22 m (4H_arom._), 7.24 d (2H_arom._, ^3^*J* 8.1 Hz). ^13^C-NMR (CDCl_3_, 100 MHz, measured in a mixture with **4em**) δ, ppm: 16.2 q (CH_3_, *J*_C-F_ 3.8 Hz), 20.0 (CH_3_), 20.1 (CH_3_), 21.1 (CH_3_), 21.4 (CH_3_), 65.0 q (C^1^, *J*_C-F_ 25.9 Hz), 126.8 q (*J*_C-F_ 1.7 Hz), 127.4 q (CF_3_, *J*_C-F_ 283.5 Hz), 128.5, 128.8, 129.5, 129.6, 132.0, 132.1, 133.8, 134.8, 135.8 q (*J*_C-F_ 1.9 Hz), 136.4, 137.2, 137.4, 141.1, 143.6, 147.8. ^19^F-NMR (CDCl_3_, 376 MHz, measured in a mixture with **4em**) δ, ppm: –62.85 s (CF_3_). HRMS (MALDI-TOF): C_27_H_26_F_3_ found 407.1998 [M + H]^+^; calcd. 407.1981. Compound **4em**: ^1^Н-NMR (CDCl_3_, 400 MHz, measured in a mixture with **4dm**) δ, ppm: 1.95 s (6H, CH_3_), 2.25 s (3H, CH_3_), 2.33 (3H, CH_3_), 2.39 (3H, CH_3_), 6.13 s (1H, C^2^H), 6.95 s (1H, C^6^H), 7.09-7.11 m (2H_arom._), 7.17-7.25 m (4H_arom._), 7.28-7.31 m (2H_arom._). ^13^C-NMR (CDCl_3_, 100 MHz, measured in a mixture with **4dm**) δ, ppm: 16.2 (CH_3_), 20.0 (CH_3_), 20.1 (CH_3_), 21.1 (CH_3_), 21.4 (CH_3_), 65.0 (C^1^), 127.0 q (*J*_C-F_ 1.9 Hz), 127.5, 127.7 q (CF_3_, *J*_C-F_ 282.7 Hz), 128.81, 128.83, 131.5 q (*J*_C-F_ 1.4 Hz), 132.5, 133.9, 134.7, 135.2, 135.6 q (*J*_C-F_ 1.9 Hz), 136.5, 137.2, 137.5, 141.1, 143.6, 148.1. ^19^F-NMR (CDCl_3_, 376 MHz, measured in a mixture with **4dm**) δ, ppm: –62.78 s (CF_3_). HRMS (MALDI-TOF): C_27_H_26_F_3_ found 407.1998 [M + H]^+^; calcd. 407.1981.

 **1,3-Bis(4-chlorophenyl)-4,6,7-trimethyl-1-(trifluoromethyl)-*1H*-indene (4dn) and 1,3-bis(4-chlorophenyl)-4,5,7-trimethyl-1-(trifluoromethyl)-*1H*-indene (4en):** The reaction scale is 30 mg (0.09 mmol) alcohol **1q** and 12 mg (0.1 mmol) pseudocumene, isolated amount is 19 mg mixture of **4dn** and **4en** (total yield 49%, ratio **4dn** : **4en** = 10:1, respectively). Compound **4dn**: ^1^Н-NMR (CDCl_3_, 400 MHz, measured in a mixture with **4en**) δ, ppm: 1.96 s (6H, CH_3_), 2.26 s (3H, CH_3_), 6.12 s (1H, C^2^H), 6.99 s (1H_arom._), 7.25 d (2H_arom._, ^3^*J* 8.8 Hz), 7.27-7.31 m (2H_arom._), 7.30 d (2H_arom._, ^3^*J* 8.6 Hz), 7.39 d (2H_arom._, ^3^*J* 8.6 Hz). ^13^C-NMR (CDCl_3_, 100 MHz, measured in a mixture with **4en**) δ, ppm: 16.2 q (CH_3_, *J*_C-F_ 3.8 Hz), 20.06 (CH_3_), 20.15 (CH_3_), 64.9 q (C^1^, *J*_C-F_ 26.4 Hz), 127.0 q (CF_3_, *J*_C-F_ 283.2 Hz), 128.4 q (*J*_C-F_ 2.0 Hz), 128.5, 129.1, 129.6, 130.0, 132.2, 133.53, 133.54, 134.0, 134.2, 135.6 q (*J*_C-F_ 1.8 Hz), 135.9, 137.1, 140.5, 143.1, 147.4. ^19^F-NMR (CDCl_3_, 376 MHz, measured in a mixture with **4en**) δ, ppm: –62.92 s (CF_3_). HRMS (MALDI-TOF): C_25_H_20_F_3_Cl_2_ found 447.0890 [M + H]^+^; calcd. 447.0889. Compound **4dn**: ^1^Н-NMR (CDCl_3_, 400 MHz, measured in a mixture with **4dn**) δ, ppm: 1.90 s (3H, CH_3_), 2.02 s (3H, CH_3_), 2.27 s (3H, CH_3_), 6.18 s (1H, C^2^H), 6.93 s (1H_arom._), 7.24-7.27 m (2H_arom._), 7.27-7.32 m (4H_arom._), 7.38-7.41 m (2H_arom._). ^19^F-NMR (CDCl_3_, 376 MHz, measured in a mixture with **4dn**) δ, ppm: –62.76 s (CF_3_). HRMS (MALDI-TOF): C_25_H_20_F_3_Cl_2_ found 447.0890 [M + H]^+^; calcd. 447.0889.

 **1,3-Bis(4-bromophenyl)-4,6,7-trimethyl-1-(trifluoromethyl)-*1H*-indene (4do)** and **1,3-bis(4-bromophenyl)-4,5,7-trimethyl-1-(trifluoromethyl)-*1H*-indene (4eo):** The reaction scale is 40 mg (0.1 mmol) alcohol **1r** and 14 mg (0.12 mmol) pseudocumene, isolated amount is 28 mg mixture of **4do** and **4eo** (total yield 57%, ratio **4do** : **4eo** = 11.5:1, respectively). ^1^Н-NMR (CDCl_3_, 400 MHz, measured in a mixture with **4eo**) δ, ppm: 1.95 s (6H, CH_3_), 2.26 s (3H, CH_3_), 6.12 s (1H, C^2^H), 6.99 s (1H_arom._), 7.19 d (2H_arom._, ^3^*J* 8.6 Hz), 7.24 d (2H_arom._, ^3^*J* 8.4 Hz), 7.44 d (2H_arom._, ^3^*J* 8.6 Hz), 7.54 d (2H_arom._, ^3^*J* 8.6 Hz). ^13^C-NMR (CDCl_3_, 100 MHz, measured in a mixture with **4eo**) δ, ppm: 16.3 q (CH_3_, *J*_C-F_ 3.6 Hz), 20.1 (CH_3_), 20.2 (CH_3_), 65.0 q (C^1^, *J*_C-F_ 26.5 Hz), 121.6, 122.1, 127.0 q (CF_3_, *J*_C-F_ 283.6 Hz), 128.7 q (*J*_C-F_ 1.4 Hz), 129.6, 130.3, 131.4, 132.1, 132.3, 134.1, 134.2, 135.5 q (*J*_C-F_ 1.3 Hz), 136.4, 137.1, 140.4, 143.0, 147.4. ^19^F-NMR (CDCl_3_, 376 MHz, measured in a mixture with **4eo**) δ, ppm: –62.90 s (CF_3_). HRMS (MALDI-TOF): C_25_H_20_F_3_Br_2_ found 534.9885 [M + H]^+^; calcd. 534.9879. Compound **4eo**: ^1^Н-NMR (CDCl_3_, 400 MHz, measured in a mixture with **4do**) δ, ppm: 1.90 s (3H, CH_3_), 2.02 s (3H, CH_3_), 2.27 s (3H, CH_3_), 6.17 s (1H, C^2^H), 6.93 s (1H_arom._), 7.42-7.46 m (2H_arom._), 7.49 d (2H_arom._, ^3^*J* 8.8 Hz), 7.53-7.55 m (2H_arom._), 7.61 d (2H_arom._, ^3^*J* 8.8 Hz). ^19^F-NMR (CDCl_3_, 376 MHz, measured in a mixture with **4do**) δ, ppm: –62.74 s (CF_3_). HRMS (MALDI-TOF): C_25_H_20_F_3_Br_2_ found 534.9885 [M + H]^+^; calcd. 534.9879.

 **5,6-Dimethoxy-1,3-diphenyl-1-(trifluoromethyl)-*1H*-indene (4fa):** Pale yellow solid, mp 157°С (МеОН-CH_2_Cl_2_). Isolated from the reaction of alcohol **1a** (30 mg, 0.11 mmol), veratrole (16 mg, 0.12 mmol) and 1.5 eq. TfOH (26 mg, 0.17 mmol), yield 60% (24 mg, mixture of **4fa** and **3a,** 2.6:1, respectively) or from the reaction with 2.5 eq. TfOH (42 mg, 0.28 mmol), yield 60% (24 mg **4fa**). ^1^Н-NMR (CDCl_3_, 400 MHz) δ, ppm: 3.91 (3H, CH_3_), 3.94 (3H, CH_3_), 6.55 s (1H, C^2^H), 7.08 s (1H, C^4^H), 7.17 s (1H, C^7^H), 7.32-7.36 m (3H_arom._), 7.41-7.53 m (5H_arom._), 7.60-7.62 m (2H_arom._). ^13^C-NMR (CDCl_3_, 100 MHz) δ, ppm: 56.3 (CH_3_), 56.6 (CH_3_), 64.2 q (C^1^, *J*_C-F_ 26.9 Hz), 105.2, 109.7, 126.8 q (CF_3_, *J*_C-F_ 282.5 Hz), 127.8, 127.8 q (*J*_C-F_ 1.6 Hz), 128.1, 128.6, 128.8, 128.9, 131.4 q (*J*_C-F_ 1.5 Hz), 134.7, 135.0, 136.6, 136.6, 147.1, 148.4, 149.9. ^19^F-NMR (CDCl_3_, 376 MHz) δ, ppm: –67.40 s (CF_3_). HRMS (MALDI-TOF): C_24_H_20_F_3_O_2_ found 397.1397 [M + H]^+^; calcd. 397.1410.

 **2-(3,4-Dimethoxyphenyl)-2,4-diphenyl-1,1,1-trifluorobut-3-yne (3a):** The reaction scale is 30 mg (0.11 mmol) alcohol **1a**, 16 mg (0.12 mmol) veratrole and 26 mg TfOH (0.17 mmol), isolated amount is 24 mg mixture of **3a** and **4fa** (total yield 60%, ratio **3a** : **4fa** = 1:2.8, respectively). ^1^Н-NMR (CDCl_3_, 400 MHz, measured in a mixture with **4fa**) δ, ppm: 3.82 (3H, CH_3_), 3.89 (3H, CH_3_), 6.85 d (1H_arom._, ^3^*J* 8.5 Hz), 7.08-7.17 m (2H_arom._), 7.32-7.53 m (7H_arom._), 7.60-7.62 m (3H_arom._). ^13^C-NMR (CDCl_3_, 100 MHz, measured in a mixture with **4fa**) δ, ppm: 56.0 (CH_3_), 56.1 (CH_3_), 64.5 q (C^2^, *J*_C-F_ 23.4 Hz), 86.9 q (C^3^, *J*_C-F_ 2.3 Hz), 87.5 (C^4^), 110.7, 112.7 q (*J*_C-F_ 1.0 Hz), 121.5 q (*J*_C-F_ 1.7 Hz), 122.4, 125.6 q (C^1^, *J*_C-F_ 284.5 Hz), 127.8, 128.2, 128.4, 128.5, 129.0, 130.4, 132.0, 138.3, 148.7, 149.0. ^19^F-NMR (CDCl_3_, 376 MHz, measured in a mixture with **4fa**) δ, ppm: –67.60 s (CF_3_). HRMS (MALDI-TOF): C_24_H_20_F_3_O_2_ found 397.1397 [M + H]^+^; calcd. 397.1410.

**1-(3-Methylphenyl)-5,6-dimethoxy-3-phenyl-1-(trifluoromethyl)-*1H*-indene (4fb)** and **2-(3,4-dimethoxyphenyl)-2-(3-methylphenyl)-4-phenyl-1,1,1-trifluorobut-3-yne (3b):** The reaction scale is 30 mg (0.1 mmol) alcohol **1b**, 16 mg (0.11 mmol) veratrole and 23 mg TfOH (0.15 mmol), isolated amount is 20 mg mixture of **4fb** and **3b** (total yield 49%, ratio **4fb** : **3b** = 3.5:1, respectively). Compound **4fb**: ^1^Н-NMR (CDCl_3_, 400 MHz, measured in a mixture with **3b**) δ, ppm: 2.34 s (3H, CH_3_), 3.92 s (3H, CH_3_), 3.94 s (3H, CH_3_), 6.55 s (1H, C^2^H), 7.09 s (1H, C^4^H), 7.12-7.14 m (1H_arom._), 7.19 s (1H_arom._), 7.23 t (1H_arom._, ^3^*J* 7.6 Hz), 7.32 s (1H_arom._), 7.35-7.37 m (1H_arom._), 7.41-7.45 m (1H_arom._), 7.47-7.51 m (2H_arom._), 7.60-7.63 m (2H_arom._). ^13^C-NMR (CDCl_3_, 100 MHz, measured in a mixture with **3b**) δ, ppm: 21.8 (CH_3_), 56.3 (CH_3_), 56.6 (CH_3_), 64.1 q (C^1^, *J*_C-F_ 26.8 Hz), 105.2, 109.7, 124.9 q (*J*_C-F_ 1.5 Hz), 126.9 q (CF_3_, *J*_C-F_ 282.6 Hz), 127.8, 128.5, 128.6, 128.7, 128.86, 128.92, 131.4 q (*J*_C-F_ 1.2 Hz), 131.9, 134.7, 134.8, 136.6, 138.4, 146.9, 148.3, 149.8. ^19^F-NMR (CDCl_3_, 376 MHz, measured in a mixture with **3b**) δ, ppm: –67.32 s (CF_3_). HRMS (MALDI-TOF): C_25_H_22_F_3_O_2_ found 411.1552 [M + H]^+^; calcd. 411.1567. Compound **3b**: ^1^Н-NMR (CDCl_3_, 400 MHz, measured in a mixture with **4fb**) δ, ppm: 2.37 s (3H, CH_3_), 3.83 s (3H, CH_3_), 3.90 s (3H, CH_3_), 6.86 d (1H_arom._, ^3^*J* 8.3 Hz), 7.12-7.19 m (3H_arom._), 7.25-7.28 m (1H_arom._), 7.32-7.38 m (3H_arom._), 7.39-7.45 m (2H_arom._), 7.53-7.55 m (2H_arom._). ^13^C-NMR (CDCl_3_, 100 MHz, measured in a mixture with **4fb**) δ, ppm: 21.8 (CH_3_), 56.0 (CH_3_), 56.1 (CH_3_), 64.1 (C^2^), 87.1 q (C^3^, *J*_C-F_ 2.4 Hz), 87.4 (C^4^), 110.7, 112.7, 121.5, 122.5, 125.6 q (C^1^, *J*_C-F_ 284.5 Hz), 125.9 q (*J*_C-F_ 1.2 Hz), 127.8, 128.3, 128.5, 129.0, 129.5 q (*J*_C-F_ 0.9 Hz), 130.5, 131.9, 138.10, 138.14, 148.7, 149.0. ^19^F-NMR (CDCl_3_, 376 MHz, measured in a mixture with **4fb**) δ, ppm: –67.50 s (CF_3_). HRMS (MALDI-TOF): C_25_H_22_F_3_O_2_ found 411.1552 [M + H]^+^; calcd. 411.1567.

**1-(4-Methylphenyl)-5,6-dimethoxy-3-phenyl-1-(trifluoromethyl)-*1H*-indene (4fc)** and **2-(3,4-dimethoxyphenyl)-2-(4-methylphenyl)-4-phenyl-1,1,1-trifluorobut-3-yne (3c):** The reaction scale is 30 mg (0.1 mmol) alcohol **1c**, 16 mg (0.11 mmol) veratrole and 23 mg TfOH (0.15 mmol), isolated amount is 20 mg mixture of **4fc** and **3c** (total yield 49%, ratio **4fc** : **3c** = 3.5:1, respectively). Compound **4fc**: ^1^Н-NMR (CDCl_3_, 400 MHz, measured in a mixture with **3c**) δ, ppm: 2.34 s (3H, CH_3_), 3.91 s (3H, CH_3_), 3.95 s (3H, CH_3_), 6.55 s (1H, C^2^H), 7.08 s (1H, C^4^H), 7.15 d (2H_arom._, ^3^*J* 8.1 Hz), 7.19 s (1H, C^7^H), 7.41-7.51 m (3H_arom._), 7.47-7.51 m (2H_arom._), 7.60-7.62 m (2H_arom._). ^13^C-NMR (CDCl_3_, 100 MHz, measured in a mixture with **3c**) δ, ppm: 21.1 (CH_3_), 56.3 (CH_3_), 56.5 (CH_3_), 63.8 q (C^1^, *J*_C-F_ 26.7 Hz), 105.2, 109.6, 126.9 q (CF_3_, *J*_C-F_ 282.5 Hz), 127.7 q (*J*_C-F_ 1.6 Hz), 127.8, 128.6, 128.9, 129.5, 131.4 q (*J*_C-F_ 1.6 Hz), 131.8, 131.9, 134.7, 136.5, 137.9, 146.9, 148.3, 149.8. ^19^F-NMR (CDCl_3_, 376 MHz, measured in a mixture with **3c**) δ, ppm: –67.59 s (CF_3_). HRMS (MALDI-TOF): C_25_H_22_F_3_O_2_ found 411.1582 [M + H]^+^; calcd. 411.1567. Compound **3c**: ^1^Н-NMR (CDCl_3_, 400 MHz, measured in a mixture with **4fc**) δ, ppm: 2.37 s (3H, CH_3_), 3.84 s (3H, CH_3_), 3.90 s (3H, CH_3_), 6.86 d (1H_arom._, ^3^*J* 8.3 Hz), 7.14-7.19 m (4H_arom._), 7.33-7.37 m (3H_arom._), 7.47-7.51 m (2H_arom._), 7.53-7.55 m (2H_arom._). ^13^C-NMR (CDCl_3_, 100 MHz, measured in a mixture with **4fc**) δ, ppm: 21.1 (CH_3_), 56.0 (CH_3_), 56.1 (CH_3_), 64.4 q (C^2^, *J*_C-F_ 23.8 Hz), 87.1 q (C^3^, *J*_C-F_ 2.6 Hz), 87.3 (C^4^), 110.7, 112.6, 121.0, 121.4 q (*J*_C-F_ 1.8 Hz), 125.6 q (C^1^, *J*_C-F_ 284.2 Hz), 128.5, 128.6, 128.7 q (*J*_C-F_ 1.5 Hz), 129.1, 130.5, 135.3, 136.6, 138.1, 148.7, 149.0. ^19^F-NMR (CDCl_3_, 376 MHz, measured in a mixture with **4fc**) δ, ppm: –67.70 s (CF_3_). HRMS (MALDI-TOF): C_25_H_22_F_3_O_2_ found 411.1582 [M + H]^+^; calcd. 411.1567.

**1-(3,4-Dimethylphenyl)-5,6-dimethoxy-3-phenyl-1-(trifluoromethyl)-*1H*-indene (4fd)** and **2-(3,4-dimethoxyphenyl)-2-(3,4-dimethylphenyl)-4-phenyl-1,1,1-trifluorobut-3-yne (3d):** The reaction scale is 30 mg (0.1 mmol) alcohol **1d**, 16 mg (0.11 mmol) veratrole and 23 mg TfOH (0.15 mmol), isolated amount is 32 mg mixture of **4fd** and **3d** (total yield 76%, ratio **4fd** : **3d** = 5:1, respectively). Compound **4fd**: ^1^Н-NMR (CDCl_3_, 400 MHz, measured in a mixture with **3d**) δ, ppm: 2.25 s (6H, CH_3_), 3.91 s (3H, CH_3_), 3.95 s (3H, CH_3_), 6.54 s (1H, C^2^H), 7.08 s (1H, C^4^H), 7.10 d (1H_arom._, ^3^*J* 8.6 Hz), 7.20 s (1H, C^7^H), 7.26-7.29 m (2H_arom._), 7.40-7.44 m (1H_arom._), 7.46-7.50 m (2H_arom._), 7.69-7.62 m (2H_arom._). ^13^C-NMR (CDCl_3_, 100 MHz, measured in a mixture with **3d**) δ, ppm: 19.5 (CH_3_), 20.2 (CH_3_), 56.3 (CH_3_), 56.6 (CH_3_), 63.8 q (C^1^, *J*_C-F_ 26.7 Hz), 105.2, 109.7, 125.3 q (*J*_C-F_ 1.7 Hz), 126.9 q (CF_3_, *J*_C-F_ 282.4 Hz), 127.8, 128.6, 128.9, 129.0 q (*J*_C-F_ 1.6 Hz), 130.0, 131.50 q (*J*_C-F_ 1.6 Hz), 131.52, 132.2, 134.8, 136.58, 136.63, 137.0, 146.8, 148.3, 149.8. ^19^F-NMR (CDCl_3_, 376 MHz, measured in a mixture with **3d**) δ, ppm: –67.52 s (CF_3_). HRMS (MALDI-TOF): C_26_H_24_F_3_O_2_ found 425.1727 [M + H]^+^; calcd. 425.1723. Compound **3d**: ^1^Н-NMR (CDCl_3_, 400 MHz, measured in a mixture with **4fd**) δ, ppm: 2.27 s (6H, CH_3_), 3.84 s (3H, CH_3_), 3.90 s (3H, CH_3_), 6.85 d (1H_arom._, ^3^*J* 9.2 Hz), 7.09-7.12 m (1H_arom._), 7.14-7.16 m (2H_arom._), 7.31-7.36 m (5H_arom._), 7.52-7.55 m (2H_arom._). ^13^C-NMR (CDCl_3_, 100 MHz, measured in a mixture with **4fd**) δ, ppm: 19.5 (CH_3_), 20.2 (CH_3_), 56.0 (CH_3_), 56.1 (CH_3_), 63.8 (C^2^), 87.2 q (C^3^, *J*_C-F_ 2.4 Hz), 87.2 (C^4^), 110.7, 112.7 q (*J*_C-F_ 1.0 Hz), 121.5 q (*J*_C-F_ 1.7 Hz), 122.5, 125.6 q (C^1^, *J*_C-F_ 284.5 Hz), 126.1 q (*J*_C-F_ 1.7 Hz), 128.5, 128.9, 129.6, 129.9 q (*J*_C-F_ 1.5 Hz), 130.6, 135.6, 136.6, 136.7, 136.7, 148.7, 148.9. ^19^F-NMR (CDCl_3_, 376 MHz, measured in a mixture with **4fd**) δ, ppm: –67.61 s (CF_3_). HRMS (MALDI-TOF): C_26_H_24_F_3_O_2_ found 425.1727 [M + H]^+^; calcd. 425.1723.

**1-(4-Chlorophenyl)-5,6-dimethoxy-3-phenyl-1-(trifluoromethyl)-*1H*-indene (4fe)** and **2-(4-chlorophenyl)-2-(3,4-dimethoxyphenyl)-4-phenyl-1,1,1-trifluorobut-3-yne (3e):** The reaction scale is 30 mg (0.1 mmol) alcohol **1e**, 16 mg (0.11 mmol) veratrole and 23 mg TfOH (0.15 mmol), isolated amount is 19 mg mixture of **4fe** and **3e** (total yield 44%, ratio **4fe** : **3e** = 6:1, respectively). Compound **4fe**: ^1^Н-NMR (CDCl_3_, 400 MHz, measured in a mixture with **3e**) δ, ppm: 3.91 s (3H, CH_3_), 3.93 s (3H, CH_3_), 6.49 s (1H, C^2^H), 7.08 s (1H, C^4^H), 7.12 s (1H, C^7^H), 7.30 (2H_arom._, ^3^*J* 8.8 Hz), 7.42-7.54 m (5H_arom._), 7.58-7.61 m (2H_arom._). ^13^C-NMR (CDCl_3_, 100 MHz, measured in a mixture with **3e**) δ, ppm: 56.3 (CH_3_), 56.6 (CH_3_), 63.7 q (C^1^, *J*_C-F_ 27.1 Hz), 105.3, 109.4, 126.7 q (CF_3_, *J*_C-F_ 282.4 Hz), 127.8, 128.8, 128.97, 128.99, 129.2 q (*J*_C-F_ 1.9 Hz), 130.9 q (*J*_C-F_ 1.6 Hz), 132.0, 133.6, 134.1, 134.5, 136.3 q (*J*_C-F_ 1.0 Hz), 136.5, 147.6, 150.0. ^19^F-NMR (CDCl_3_, 376 MHz, measured in a mixture with **3e**) δ, ppm: –67.48 s (CF_3_). HRMS (MALDI-TOF): C_24_H_19_F_3_ClO_2_ found 431.1028 [M + H]^+^; calcd. 431.1021. Compound **3e**: ^1^Н-NMR (CDCl_3_, 400 MHz, measured in a mixture with **4fe**) δ, ppm: 3.83 s (3H, CH_3_), 3.90 s (3H, CH_3_), 6.86 d (1H_arom._, ^3^*J* 8.5 Hz), 7.08-7.12 m (2H_arom._), 7.28-7.38 m (5H_arom._), 7.42-7.54 m (4H_arom._). ^13^C-NMR (CDCl_3_, 100 MHz, measured in a mixture with **4fe**) δ, ppm: 56.0 (CH_3_), 56.1 (CH_3_), 63.7 (C^2^), 86.4 q (C^3^, *J*_C-F_ 2.4 Hz), 87.8 (C^4^), 110.8, 112.5 q (*J*_C-F_ 1.6 Hz), 121.4 q (*J*_C-F_ 1.8 Hz), 122.1, 125.4 q (C^1^, *J*_C-F_ 284.6 Hz), 128.1, 128.56, 128.63, 129.1, 129.9, 130.3 q (*J*_C-F_ 1.7 Hz), 134.4, 136.9, 148.9, 149.2. ^19^F-NMR (CDCl_3_, 376 MHz, measured in a mixture with **4fe**) δ, ppm: –67.79 s (CF_3_). HRMS (MALDI-TOF): C_24_H_19_F_3_ClO_2_ found 431.1028 [M + H]^+^; calcd. 431.1021.

**1-(4-Bromophenyl)-5,6-dimethoxy-3-phenyl-1-(trifluoromethyl)-*1H*-indene (4ff)** and **2-(4-bromophenyl)-2-(3,4-dimethoxyphenyl)-4-phenyl-1,1,1-trifluorobut-3-yne (3f)**: The reaction scale is 30 mg (0.09 mmol) alcohol **1f**, 14 mg (0.1 mmol) veratrole and 21 mg TfOH (0.14 mmol), isolated amount is 28 mg mixture of **4ff** and **3f** (total yield 70%, ratio **4ff**:**3f** = 4:1, respectively). Compound **4ff**: ^1^Н-NMR (CDCl_3_, 400 MHz, measured in a mixture with **3f**) δ, ppm: 3.91 s (3H, CH_3_), 3.93 s (3H, CH_3_), 6.48 s (1H, C^2^H), 7.07 s (1H, C^4^H), 7.11 s (1H, C^7^H), 7.36-7.38 m (2H_arom._), 7.41-7.53 m (5H_arom._), 7.58-7.60 m (2H_arom._). ^13^C-NMR (CDCl_3_, 100 MHz, measured in a mixture with **3f**) δ, ppm: 56.3 (CH_3_), 56.6 (CH_3_), 63.8 q (C^1^, *J*_C-F_ 27.1 Hz), 105.3, 109.4, 122.3, 126.6 q (CF_3_, *J*_C-F_ 282.5 Hz), 127.8, 128.8, 129.0, 129.5 q (*J*_C-F_ 1.9 Hz), 130.8 q (*J*_C-F_ 1.6 Hz), 131.9, 134.2, 134.4, 136.2 q (*J*_C-F_ 0.6 Hz), 136.5, 147.5, 148.6, 150.0. ^19^F-NMR (CDCl_3_, 376 MHz, measured in a mixture with **3f**) δ, ppm: –67.44 s (CF_3_). HRMS (MALDI-TOF): C_24_H_19_F_3_BrO_2_ found 475.0517 [M + H]^+^; calcd. 475.0515. Compound **3e**: ^1^Н-NMR (CDCl_3_, 400 MHz, measured in a mixture with **4ff**) δ, ppm: 3.83 s (3H, CH_3_), 3.90 s (3H, CH_3_), 6.85 d (1H_arom._, ^3^*J* 8.4 Hz), 7.07-7.12 m (2H_arom._), 7.34-7.38 m (3H_arom._), 7.41-7.53 m (6H_arom._). ^13^C-NMR (CDCl_3_, 100 MHz, measured in a mixture with **4ff**) δ, ppm: 56.0 (CH_3_), 56.1 (CH_3_), 63.8 (C^2^), 86.3 q (C^3^, *J*_C-F_ 2.3 Hz), 87.9 (C^4^), 110.8, 112.5 q (*J*_C-F_ 1.2 Hz), 121.4 q (*J*_C-F_ 1.7 Hz), 122.1, 122.6, 125.3 q (C^1^, *J*_C-F_ 284.1 Hz), 128.6, 129.2, 129.8, 130.6 q (*J*_C-F_ 1.6 Hz), 131.6, 132.0, 137.5, 148.9, 149.2. ^19^F-NMR (CDCl_3_, 376 MHz, measured in a mixture with **4ff**) δ, ppm: –67.77 s (CF_3_). HRMS (MALDI-TOF): C_24_H_19_F_3_BrO_2_ found 475.0517 [M + H]^+^; calcd. 475.0515.

**1-(2-Bromophenyl)-5,6-dimethoxy-3-phenyl-1-(trifluoromethyl)-*1H*-indene (4fg):** Yellow oil. The reaction scale is 30 mg (0.09 mmol) alcohol **1g**, 14 mg (0.1 mmol) veratrole and 35 mg TfOH (0.23 mmol), isolated amount is 27 mg **4fg** (yield 82%). ^1^Н-NMR (CDCl_3_, 400 MHz) δ, ppm: 3.83 s (3H, CH_3_), 3.91 s (3H, CH_3_), 6.29 s (1H, C^2^H), 6.82 s (1H, C^7^H), 7.06 s (1H, C^4^H), 7.17 td (1H_arom._, ^3^*J* 7.7 Hz, ^4^*J* 1.4 Hz), 7.40-7.54 m (5H_arom._), 7.62-7.65 m (2H_arom._), 7.95−8.00 m (1H_arom._). ^13^C-NMR (CDCl_3_, 100 MHz) δ, ppm: 56.3 (CH_3_), 56.5 (CH_3_), 65.6 q (C^1^, *J*_C-F_ 25.4 Hz), 105.2, 108.7, 122.9 q (*J*_C-F_ 0.6 Hz), 126.5 q (CF_3_, *J*_C-F_ 284.1 Hz), 127.5, 127.6, 128.2, 128.7, 129.0, 129.2, 130.6 q (*J*_C-F_ 4.4 Hz), 133.6, 135.0, 136.4, 136.7 q (*J*_C-F_ 0.6 Hz), 139.2, 148.7, 149.9, 150.9. ^19^F-NMR (CDCl_3_, 376 MHz) δ, ppm: –65.35 s (CF_3_). HRMS (MALDI-TOF): C_24_H_19_F_3_BrO_2_ found 475.0529 [M + H]^+^; calcd. 475.0515.

**3-(4-Methylphenyl)-5,6-dimethoxy-1-phenyl-1-(trifluoromethyl)-*1H*-indene (4fh) and 2-(3,4-dimethoxyphenyl)-4-(4-methylphenyl)-2-phenyl-1,1,1-trifluorobut-3-yne (3g):** The reaction scale is 30 mg (0.1 mmol) alcohol **1i**, 16 mg (0.11 mmol) veratrole and 23 mg TfOH (0.15 mmol), isolated amount is 22 mg mixture of **4fh** and **3g** (total yield 54%, ratio **4fh** : **3g** = 13:1, respectively). Compound **4fh**: ^1^Н-NMR (CDCl_3_, 400 MHz measured in a mixture with **3g**) δ, ppm: 2.43 s (3H, CH_3_), 3.91 (3H, CH_3_), 3.93 (3H, CH_3_), 6.51 s (1H, C^2^H), 7.08 s (1H, C^4^H), 7.17 s (1H, C^7^H), 7.29 d (2H_arom._, ^3^*J* 8.0 Hz), 7.31-7.36 m (3H_arom._), 7.50 d (2H_arom._, ^3^*J* 8.0 Hz), 7.51-7.53 m (2H_arom._). ^13^C-NMR (CDCl_3_, 100 MHz, measured in a mixture with **3g**) δ, ppm: 21.5 (CH_3_), 56.3 (CH_3_), 56.6 (CH_3_), 64.1 q (C^1^, *J*_C-F_ 26.6 Hz), 105.2, 109.6, 126.9 q (CF_3_, *J*_C-F_ 282.7 Hz), 127.7, 127.8 q (*J*_C-F_ 1.8 Hz), 128.0, 128.8, 129.6, 130.8 q (*J*_C-F_ 1.7 Hz), 131.8, 135.0, 136.6 q (*J*_C-F_ 0.6 Hz), 136.8, 138.6, 147.0, 148.3, 149.8. ^19^F-NMR (CDCl_3_, 376 MHz, measured in a mixture with **3g**) δ, ppm: –67.41 s (CF_3_). HRMS (MALDI-TOF): C_25_H_22_F_3_O_2_ found 411.1556 [M + H]^+^; calcd. 411.1567. Compound **3g**: ^1^Н-NMR (CDCl_3_, 400 MHz, measured in a mixture with **4fh**) δ, ppm: 2.37 s (3H, CH_3_), 3.82 (3H, CH_3_), 3.89 (3H, CH_3_), 6.85 d (1H_arom._, ^3^*J* 8.2 Hz), 7.00 d (1H_arom._, ^3^*J* 8.2 Hz), 7.12 s (1H_arom._), 7.14-7.17 m (2H_arom._), 7.31-7.42 m (3H_arom._), 7.42 d (2H_arom._, ^3^*J* 8.0 Hz), 7.60 d (2H_arom._, ^3^*J* 7.1 Hz). ^19^F-NMR (CDCl_3_, 376 MHz, measured in a mixture with **4fh**) δ, ppm: –67.63 s (CF_3_). HRMS (MALDI-TOF): C_25_H_22_F_3_O_2_ found 411.1556 [M + H]^+^; calcd. 411.1567.

**1-(Trifluoromethyl)-5,6-dimethoxy-3-(2,4-dimethylphenyl)-1-phenyl-1H-indene (4fi):** Yellow solid, mp 119 °С (МеОН-CH_2_Cl_2_). The reaction scale is 30 mg (0.1 mmol) alcohol **1j**, 16 mg (0.11 mmol) veratrole and 38 mg TfOH (0.25 mmol), isolated amount is 28 mg **4fi** (yield 67%). ^1^Н-NMR (CDCl_3_, 400 MHz) δ, ppm: 2.25 s (3H, CH_3_), 2.40 (3H, CH_3_), 3.82 (3H, CH_3_O), 3.92 (3H, CH_3_O), 6.39 s (1H, C^2^H), 6.63 s (1H, C^4^H), 7.09 d (1H_arom._, ^3^*J* 7.7 Hz), 7.15 s (1H_arom._), 7.16 s (1H, C^7^H), 7.21 d (1H_arom._, ^3^*J* 7.7 Hz), 7.31-7.37 m (3H_arom._), 7.52-7.55 m (2H_arom._). ^13^C-NMR (CDCl_3_, 100 MHz) δ, ppm: 20.1 (CH_3_), 21.3 (CH_3_), 56.3 (CH_3_O), 56.6 (CH_3_O), 64.5 q (C^1^, *J*_C-F_ 26.7 Hz), 105.1, 109.4, 126.6, 126.9 q (CF_3_, *J*_C-F_ 282.5 Hz), 127.8 q (*J*_C-F_ 1.8 Hz), 128.0, 128.8, 129.1, 131.2, 131.4, 132.4 q (*J*_C-F_ 1.6 Hz), 135.1, 135.9, 136.3, 138.07, 138.15, 147.2, 148.3, 150.0. ^19^F-NMR (CDCl_3_, 376 MHz) δ, ppm: –67.44 s (CF_3_). HRMS (MALDI-TOF): C_26_H_24_F_3_O_2_ found 425.1712 [M + H]^+^; calcd. 425.1723.

**5,6-Dimethoxy-3-(3,4-dimethylphenyl)-1-phenyl-1-(trifluoromethyl)-1H-indene (4fj):** Colorless solid, mp 133 °С (МеОН-CH_2_Cl_2_). The reaction scale is 30 mg (0.1 mmol) alcohol **1k**, 16 mg (0.11 mmol) veratrole and 38 mg TfOH (0.25 mmol), isolated amount is 24 mg **4fj** (yield 57%). ^1^Н-NMR (CDCl_3_, 400 MHz) δ, ppm: 2.34 s (6H, CH_3_), 3.91 s (3H, CH_3_O), 3.93 s (3H, CH_3_O), 6.51 s (1H, C^2^H), 7.10 s (1H_arom._), 7.17 s (1H_arom._), 7.24-7.26 m (1H_arom._), 7.29-7.37 m (4H_arom._), 7.38 s (1H_arom._), 7.51-7.55 m (2H_arom._). ^13^C-NMR (CDCl_3_, 100 MHz) δ, ppm: 19.8 (CH_3_), 20.0 (CH_3_), 56.3 (CH_3_), 56.5 (CH_3_), 64.1 q (C^1^, *J*_C-F_ 26.8 Hz), 105.3, 109.6, 125.1, 126.9 q (CF_3_, *J*_C-F_ 282.5 Hz), 127.8, 128.0, 128.8, 129.0, 130.1, 130.7, 132.2, 135.1, 136.6, 136.8, 137.2 (2С), 147.0, 148.3, 149.8. ^19^F-NMR (CDCl_3_, 376 MHz) δ, ppm: –67.41 s (CF_3_). HRMS (MALDI-TOF): C_26_H_24_F_3_O_2_ found 425.1715 [M + H]^+^; calcd. 425.1723.

**3-(4-Chlorophenyl)-5,6-dimethoxy-1-phenyl-1-(trifluoromethyl)-*1H*-indene (4fk)** and **4-(4-chlorophenyl)-2-(3,4-dimethoxyphenyl)-2-phenyl-1,1,1-trifluorobut-3-yne (3h):** The reaction scale is 30 mg (0.1 mmol) alcohol **1l**, 16 mg (0.11 mmol) veratrole and 23 mg TfOH (0.15 mmol), isolated amount is 19 mg mixture of **4fk** and **3h** (total yield 65%, ratio **4fk** : **3h** = 10:1, respectively). Compound **4fk**: Colorless solid, mp 173 °С (МеОН-CH_2_Cl_2_). ^1^Н-NMR (CDCl_3_, 400 MHz) δ, ppm: 3.91 s (3H, CH_3_), 3.93 s (3H, CH_3_), 6.54 s (1H, C^2^H), 7.00 s (1H, C^4^H), 7.17 s (1H, C^7^H), 7.29-7.38 d (3H_arom._), 7.45 d (2H_arom._, ^3^*J* 8.5 Hz), 7.49-7.52 m (2H_arom._), 7.53 d (2H_arom._, ^3^*J* 8.5 Hz). ^13^C-NMR (CDCl_3_, 100 MHz) δ, ppm: 56.3 (CH_3_), 56.6 (CH_3_), 64.2 q (C^1^, *J*_C-F_ 26.9 Hz), 105.0, 109.7, 126.7 q (CF_3_, *J*_C-F_ 282.5 Hz), 127.8 q (*J*_C-F_ 1.8 Hz), 128.2, 128.9, 129.1, 129.2, 131.7 q (*J*_C-F_ 1.6 Hz), 133.1, 134.6, 134.7, 136.2, 136.5, 146.0, 148.5, 149.9. ^19^F-NMR (CDCl_3_, 376 MHz) δ, ppm: –67.41 s (CF_3_). HRMS (MALDI-TOF): C_24_H_19_F_3_ClO_2_ found 431.1007 [M + H]^+^; calcd. 431.1021. Compound **3h**: ^1^Н-NMR (CDCl_3_, 400 MHz, measured in a mixture with **4fk**) δ, ppm: 3.81 s (3H, CH_3_), 3.89 s (3H, CH_3_), 6.85 d (1H_arom._, ^3^*J* 8.5 Hz), 7.05 d (1H_arom._, ^4^*J* 1.6 Hz), 7.13 dd (1H_arom._, ^3^*J* 8.5 Hz, ^4^*J* 1.6 Hz), 7.29-7.38 m (5H_arom._), 7.43-7.46 m (2H_arom._), 7.57-7.59 m (2H_arom._). ^13^C-NMR (CDCl_3_, 100 MHz, measured in a mixture with **4fk**) δ, ppm: 56.0 (CH_3_), 56.1 (CH_3_), 64.2 (C^2^), 86.4 (C^4^), 88.0 q (C^3^, *J*_C-F_ 2.2 Hz), 110.7, 112.6, 121.5 q (*J*_C-F_ 1.8 Hz), 125.5 q (C^1^, *J*_C-F_ 284.6 Hz), 128.3, 128.5, 128.8 q (*J*_C-F_ 1.6 Hz), 128.9, 132.07, 132.14, 133.2, 135.1, 138.1, 148.8, 149.1. ^19^F-NMR (CDCl_3_, 376 MHz, measured in a mixture with **4fk**) δ, ppm: –67.57 s (CF_3_). HRMS (MALDI-TOF): C_24_H_19_F_3_ClO_2_ found 431.1007 [M + H]^+^; calcd. 431.1021.

**3-(4-Bromophenyl)-5,6-dimethoxy-1-phenyl-1-(trifluoromethyl)-*1H*-indene (4fl) and 4-(4-bromophenyl)-2-(3,4-dimethoxyphenyl)-2-phenyl-1,1,1-trifluorobut-3-yne (3i)**: The reaction scale is 48 mg (0.14 mmol) alcohol **1m**, 22 mg (0.16 mmol) veratrole and 32 mg TfOH (0.21 mmol), isolated amount is 24 mg mixture of **4fl** and **3i** (total yield 36%, ratio **4fl** : **3i** = 7:1, respectively). Compound **4fl**: ^1^Н-NMR (CDCl_3_, 400 MHz, measured in a mixture with **3i**) δ, ppm: 3.90 s (3H, CH_3_), 3.93 s (3H, CH_3_), 6.54 s (1H, C^2^H), 6.99 s (1H_arom._), 7.16 s (1H_arom._), 7.31-7.37 m (3H_arom._), 7.46 d (2H_arom._, ^3^*J* 8.4 Hz), 7.49-7.51 m (2H_arom._), 7.61 d (2H_arom._, ^3^*J* 8.4 Hz). ^13^C-NMR (CDCl_3_, 100 MHz, measured in a mixture with **3i**) δ, ppm: 56.3 (CH_3_), 56.6 (CH_3_), 64.3 q (C^1^, *J*_C-F_ 26.9 Hz), 104.9, 109.7, 122.7, 126.7 q (CF_3_, *J*_C-F_ 282.5 Hz), 127.8 q (*J*_C-F_ 1.6 Hz), 128.2, 128.9, 129.4, 131.7 q (*J*_C-F_ 1.4 Hz), 132.1, 133.6, 134.6, 136.1, 136.5 q (*J*_C-F_ 0.6 Hz), 146.1, 148.5, 149.9. ^19^F-NMR (CDCl_3_, 376 MHz, measured in a mixture with **3i**) δ, ppm: –67.42 s (CF_3_). HRMS (MALDI-TOF): C_24_H_19_F_3_BrO_2_ found 475.0523 [M + H]^+^; calcd. 475.0515. Compound **3h**: ^1^Н-NMR (CDCl_3_, 400 MHz, measured in a mixture with **4fl**) δ, ppm: 3.80 s (3H, CH_3_), 3.89 s (3H, CH_3_), 6.85 d (1H_arom._, ^3^*J* 8.5 Hz), 7.05 d (1H_arom._, ^4^*J* 1.4 Hz), 7.12 dd (1H_arom._, ^3^*J* 8.5 Hz, ^4^*J* 1.4 Hz), 7.29–7.39 s (5H_arom._), 7.45-7.51 m (2H_arom._), 7.56-7.59 m (2H_arom._). ^13^C-NMR (CDCl_3_, 100 MHz, measured in a mixture with **4fl**) δ, ppm: 56.0 (CH_3_), 56.1 (CH_3_), 64.5 (C^2^), 86.4 (C^4^), 88.1 q (C^3^, *J*_C-F_ 2.3 Hz), 110.7, 112.6 q (*J*_C-F_ 1.6 Hz), 121.3, 121.5 q (*J*_C-F_ 1.6 Hz), 123.3, 125.4 q (C^1^, *J*_C-F_ 284.2 Hz), 128.3, 128.5, 128.8 q (*J*_C-F_ 1.5 Hz), 130.2, 131.8, 133.4, 138.1, 148.7, 149.1. ^19^F-NMR (CDCl_3_, 376 MHz, measured in a mixture with **4fl**) δ, ppm: –67.57 s (CF_3_). HRMS (MALDI-TOF): C_24_H_19_F_3_BrO_2_ found 475.0523 [M + H]^+^; calcd. 475.0515.

**1-(Trifluoromethyl)-1,3-di(4-methylphenyl)-5,6-dimethoxy-*1H*-indene (4fm):** Colorless oil. The reaction scale is 30 mg (0.1 mmol) alcohol **1o**, 16 mg (0.11 mmol) veratrole and 38 mg TfOH (0.25 mmol), isolated amount is 29 mg **4fm** (yield 69%). ^1^Н-NMR (CDCl_3_, 400 MHz) δ, ppm: 2.34 s (3H, CH_3_), 2.43 s (3H, CH_3_), 3.91 s (3H, CH_3_), 3.94 s (3H, CH_3_), 6.51 s (1H, C^2^H), 7.08 s (1H, C^4^H), 7.14 d (2H_arom._, ^3^*J* 8.1 Hz), 7.18 s (1H, C^7^H), 7.29 d (2H_arom._, ^3^*J* 8.1 Hz), 7.42 d (2H_arom._, ^3^*J* 8.1 Hz), 7.50 d (2H_arom._, ^3^*J* 8.1 Hz). ^13^C-NMR (CDCl_3_, 100 MHz) δ, ppm: 21.1 (CH_3_), 21.4 (CH_3_), 56.3 (CH_3_), 56.5 (CH_3_), 63.8 q (C^1^, *J*_C-F_ 26.9 Hz), 105.2, 109.6, 126.9 q (CF_3_, *J*_C-F_ 282.6 Hz), 127.7, 127.8 q (*J*_C-F_ 1.0 Hz), 129.5, 129.6, 130.9, 131.8, 132.0, 136.6, 136.7, 137.9, 138.5, 146.8, 148.2, 149.7. ^19^F-NMR (CDCl_3_, 376 MHz) δ, ppm: –67.62 s (CF_3_). HRMS (MALDI-TOF): C_26_H_24_F_3_O_2_ found 425.1706 [M + H]^+^; calcd. 425.1723.

**1,3-Bis(4-chlorophenyl)-1-(trifluoromethyl)-5,6-dimethoxy-*1H*-indene (4fn)** and **2,4-bis(4-chlorophenyl)-1,1,1-trifluoro-2-(3,4-dimethoxyphenyl)-but-3-yne (3j)**: The reaction scale is 30 mg (0.09 mmol) alcohol **1q**, 14 mg (0.1 mmol) veratrole and 21 mg TfOH (0.14 mmol), isolated amount is 23 mg mixture of **4fn** and **3j** (total yield 50%, ratio **4fn** : **3j** = 2.8:1, respectively).Compound **4fn**: ^1^Н-NMR (CDCl_3_, 400 MHz, measured in a mixture with **3j**) δ, ppm: 3.90 s (3H, CH_3_), 3.93 s (3H, CH_3_), 6.47 s (1H, C^2^H), 6.99 s (1H, C^4^H), 7.11 s (1H, C^7^H), 7.30 (2H_arom._, ^3^*J* 8.6 Hz), 7.41 d (2H_arom._, ^3^*J* 8.6 Hz), 7.45 d (2H_arom._, ^3^*J* 8.6 Hz), 7.52 d (2H_arom._, ^3^*J* 8.6 Hz). ^13^C-NMR (CDCl_3_, 100 MHz, measured in a mixture with **3j**) δ, ppm: 56.4 (CH_3_), 56.6 (CH_3_), 63.8 q (C^1^, *J*_C-F_ 27.2 Hz), 105.1, 109.5, 126.6 q (CF_3_, *J*_C-F_ 282.5 Hz), 129.0, 129.1, 129.2 q (*J*_C-F_ 1.9 Hz), 129.2, 131.2 q (*J*_C-F_ 1.4 Hz), 132.9, 133.2, 134.2, 134.7, 136.1, 136.2, 146.4, 148.7, 150.1. ^19^F-NMR (CDCl_3_, 376 MHz, measured in a mixture with **3j**) δ, ppm: –67.50 s (CF_3_). HRMS (MALDI-TOF): C_24_H_18_F_3_Cl_2_O_2_ found 465.0626 [M + H]^+^; calcd. 465.0630. Compound **3j**: ^1^Н-NMR (CDCl_3_, 400 MHz, measured in a mixture with **4fn**) δ, ppm: 3.81 s (3H, CH_3_), 3.89 s (3H, CH_3_), 6.85 d (1H_arom._, ^3^*J* 8.5 Hz), 7.03 dd (1H_arom._, ^4^*J* 1.9 Hz), 7.08-7.11 m (1H_arom._), 7.33 d (2H_arom._, ^3^*J* 8.6 Hz), 7.34 d (2H_arom._, ^3^*J* 8.6 Hz), 7.44 d (2H_arom._, ^3^*J* 8.6 Hz), 7.49-7.53 m (2H_arom._). ^13^C-NMR (CDCl_3_, 100 MHz, measured in a mixture with **4fn**) δ, ppm: 56.0 (CH_3_), 56.1 (CH_3_), 62.4 q (C^2^, *J*_C-F_ 28.0 Hz), 86.7 (C^4^), 87.4 q (C^3^, *J*_C-F_ 2.5 Hz), 110.8, 112.4, 120.5, 121.4 q (*J*_C-F_ 1.7 Hz), 125.3 q (C^1^, *J*_C-F_ 284.8 Hz), 128.7, 128.95, 129.03, 130.3 q (*J*_C-F_ 1.5 Hz), 133.3, 134.5, 135.3, 136.7, 148.9, 149.3. ^19^F-NMR (CDCl_3_, 376 MHz, measured in a mixture with **4fn**) δ, ppm: –67.76 s (CF_3_). HRMS (MALDI-TOF): C_24_H_18_F_3_Cl_2_O_2_ found 465.0626 [M + H]^+^; calcd. 465.0630.

**1,3-Bis(4-bromophenyl)-1-(trifluoromethyl)-5,6-dimethoxy-*1H*-indene (4fo)** and **2,4-bis(4-bromophenyl)-1,1,1-trifluoro-2-(3,4-dimethoxyphenyl)-but-3-yne (3k)**: The reaction scale is 40 mg (0.1 mmol) alcohol **1r**, 16 mg (0.12 mmol) veratrole and 23 mg TfOH (0.15 mmol), isolated amount is 30 mg mixture of **4fo** and **3k** (total yield 50%, ratio **4fo** : **3k** = 2.8:1, respectively). Compound **4fo**: Colorless solid, mp 135 °С (МеОН-CH_2_Cl_2_). ^1^Н-NMR (CDCl_3_, 400 MHz) δ, ppm: 3.90 s (3H, CH_3_), 3.92 s (3H, CH_3_), 6.47 s (1H, C^2^H), 6.98 s (1H, C^4^H), 7.10 s (1H, C^7^H), 7.34 d (2H_arom._, ^3^*J* 8.6 Hz), 7.44-7.46 m (4H_arom._), 7.61 d (2H_arom._, ^3^*J* 8.6 Hz). ^13^C-NMR (CDCl_3_, 100 MHz) δ, ppm: 56.4 (CH_3_), 56.6 (CH_3_), 63.9 q (C^1^, *J*_C-F_ 27.5 Hz), 105.1, 109.5, 122.4, 122.9, 126.5 q (CF_3_, *J*_C-F_ 282.6 Hz), 129.4, 129.5 q (*J*_C-F_ 1.7 Hz), 131.1 q (*J*_C-F_ 1.3 Hz), 132.0, 132.2, 133.3, 133.8, 136.06, 136.14, 146.5, 148.7, 150.1. ^19^F-NMR (CDCl_3_, 376 MHz) δ, ppm: –67.46 s (CF_3_). HRMS (MALDI-TOF): C_24_H_18_F_3_Br_2_O_2_ found 552.9626 [M + H]^+^; calcd. 552.9620. Compound **3k**: ^1^Н-NMR (CDCl_3_, 400 MHz, measured in a mixture with **4fo**) δ, ppm: 3.81 s (3H, CH_3_), 3.89 s (3H, CH_3_), 6.85 d (1H_arom._, ^3^*J* 8.6 Hz), 7.02 dd (1H_arom._, ^4^*J* 2.0 Hz), 7.07-7.10 m (1H_arom._), 7.37 d (2H_arom._, ^3^*J* 8.6 Hz), 7.43 d (2H_arom._, ^3^*J* 8.6 Hz), 7.49 d (2H_arom._, ^3^*J* 8.6 Hz), 7.50 d (2H_arom._, ^3^*J* 8.6 Hz). ^13^C-NMR (CDCl_3_, 100 MHz, measured in a mixture with **4fo**) δ, ppm: 56.0 (CH_3_), 56.1 (CH_3_), 63.9 q (C^2^, *J*_C-F_ 27.2 Hz), 86.8 (C^4^), 87.5 q (C^3^, *J*_C-F_ 2.2 Hz), 110.8, 112.4 q (*J*_C-F_ 1.2 Hz), 121.0, 121.4 q (*J*_C-F_ 1.7 Hz), 122.7, 123.6, 125.2 q (C^1^, *J*_C-F_ 284.4 Hz), 129.6, 130.6 q (*J*_C-F_ 1.5 Hz), 131.7, 131.9, 133.4, 137.2, 148.9, 149.3. ^19^F-NMR (CDCl_3_, 376 MHz, measured in a mixture with **4fo**) δ, ppm: –67.74 s (CF_3_). HRMS (MALDI-TOF): C_24_H_18_F_3_Br_2_O_2_ found 552.9626 [M + H]^+^; calcd. 552.9620.

**1-(Trifluoromethyl)-5,6-dimethyl-1,3-diphenyl-*1H*-indene (5ac):** Colorless solid, mp 133°С (МеОН). Isolated from the reaction of alcohol **1k** (30 mg, 0.11 mmol) and benzene (0.4 mL), yield 50% (18 mg, mixture of **5ac** and **5ad** 1.6:1, respectively) or from the reaction of alcohol **1a** (30 mg, 0.11 mmol) and *o*-xylene (12 mg, 0.12 mmol), yield 48% (19 mg). ^1^Н-NMR (CDCl_3_, 400 MHz) δ, ppm: 2.32 s (3H, CH_3_), 2.35 s (3H, CH_3_), 6.58 s (1H, C^2^H), 7.30-7.35 m (4H_arom._), 7.40-7.43 m (2H_arom._), 7.46-7.50 m (2H_arom._), 7.55-7.57 m (2H_arom._), 7.60-7.63 m (2H_arom._). ^13^C-NMR (CDCl_3_, 100 MHz) δ, ppm: 20.28 (CH_3_), 20.33 (CH_3_), 64.0 q (C^1^, *J*_C-F_ 27.2 Hz), 123.0, 127.0 q (CF_3_, *J*_C-F_ 282.1 Hz), 127.1, 127.92, 127.97, 128.0, 128.6, 128.7, 128.8, 131.4, 134.7, 135.1, 135.2, 137.1, 141.4, 141.7, 147.4. ^19^F-NMR (CDCl_3_, 376 MHz) δ, ppm: –67.50 s (CF_3_). HRMS (MALDI-TOF): C_24_H_20_F_3_ found 365.1520 [M + H]^+^; calcd. 365.1512.

**6,7-Dimethyl-1,3-diphenyl-1-(trifluoromethyl)-*1H*-indene (5ad):** Isolated from the reaction of alcohol **1k** (30 mg, 0.11 mmol) and benzene (0.4 mL), yield 50% (18 mg, mixture of **5ad** and **5ac** 1:1.6, respectively). ^1^Н-NMR (CDCl_3_, 400 MHz, measured in a mixture with **5ac**) δ, ppm: 1.99 s (3H, CH_3_), 2.29 s (3H, CH_3_), 6.36 s (1H, C^2^H), 7.24 d (1H_arom._, ^3^*J* 7.9 Hz), 7.27-7.33 m (6H_arom._), 7.39-7.47 m (3H_arom._), 7.57-7.60 m (2H_arom._). ^13^C-NMR (CDCl_3_, 100 MHz, measured in a mixture with **5ac**) δ, ppm: 16.5 q (CH_3_, *J*_C-F_ 3.6 Hz), 20.4 (CH_3_), 65.7 q (C^1^, *J*_C-F_ 26.3 Hz), 118.9, 124.4 q (CF_3_, *J*_C-F_ 290.8 Hz), 127.0 q (*J*_C-F_ 1.8 Hz), 127.6, 128.0, 128.5, 128.8, 128.9, 130.5, 133.9 q (*J*_C-F_ 2.0 Hz), 134.7, 134.8, 134.9, 136.7, 142.9, 143.6, 146.2. ^19^F-NMR (CDCl_3_, 376 MHz, measured in a mixture with **5ac**) δ, ppm: –62.71 s (CF_3_). HRMS (MALDI-TOF): C_24_H_20_F_3_ found 365.1520 [M + H]^+^; calcd. 365.1512.

**3-(2,5-Dimethylphenyl)-5,6-dimethyl-1-phenyl-1-(trifluoromethyl)-*1H*-indene (5bb):** Colorless oil. The reaction scale is 30 mg (0.1 mmol) alcohol **1k** and 12 mg (0.11 mmol) *p*-xylene, isolated amount is 13 mg **5bb** (yield 34%). ^1^Н-NMR (CDCl_3_, 400 MHz) δ, ppm: 2.22 s (3H, CH_3_), 2.26 s (3H, CH_3_), 2.33 s (3H, CH_3_), 2.36 s (3H, CH_3_), 6.42 s (1H, C^2^H), 6.86 s (1H_arom._), 7.11 s (1H_arom._), 7.13 d (1H_arom._, ^3^*J* 7.8 Hz), 7.20 d (1H_arom._, ^3^*J* 7.8 Hz), 7.31-7.37 m (3H_arom._), 7.42 s (1H_arom._), 7.59 d (2H_arom._, ^3^*J* 7.3 Hz). ^13^C-NMR (CDCl_3_, 100 MHz) δ, ppm: 19.7 (CH_3_), 20.1 (CH_3_), 20.3 (CH_3_), 21.0 (CH_3_), 64.3 q (C^1^, *J*_C-F_ 26.7 Hz), 123.0, 126.8, 126.9 q (CF_3_, *J*_C-F_ 282.4 Hz), 127.9 q (*J*_C-F_ 1.7 Hz), 128.0, 128.7, 129.0, 129.8, 130.4, 132.3 q (*J*_C-F_ 1.5 Hz), 133.3, 134.2, 135.1, 135.27, 135.35, 137.2, 141.1, 142.9, 147.8. ^19^F-NMR (CDCl_3_, 376 MHz) δ, ppm: –67.54 s (CF_3_). HRMS (MALDI-TOF): C_26_H_24_F_3_ found 393.1814 [M + H]^+^; calcd. 393.1825.

**1-(3,4-Dimethylphenyl)-3-(2,5-dimethylphenyl)-5,6-dimethyl-1-(trifluoromethyl)-*1H*-indene (5bd):** Colorless solid, mp 138°С (МеОН-CH_2_Cl_2_). The reaction scale is 50 mg (0.16 mmol) alcohol **1p** and 20 mg (0.18 mmol) *p*-xylene, isolated amount is 16 mg **5bd** (yield 25%). ^1^Н-NMR (CDCl_3_, 400 MHz) δ, ppm: 2.22 s (3H, CH_3_), 2.24 s (6H, CH_3_), 2.25 s (3H, CH_3_), 2.32 s (3H, CH_3_), 2.36 s (3H, CH_3_), 6.40 s (1H, C^2^H), 6.84 d (1H_arom._, ^3^*J* 7.6 Hz), 7.09-7.14 m (3H_arom._), 7.20 d (1H_arom._, ^3^*J* 7.7 Hz), 7.32 d (1H_arom._, ^3^*J* 8.1 Hz), 7.34 s (1H_arom._), 7.42 s (1H_arom._). ^13^C-NMR (CDCl_3_, 100 MHz) δ, ppm: 19.5 (CH_3_), 19.7 (CH_3_), 20.1 (CH_3_), 20.2 (CH_3_), 20.3 (CH_3_), 21.0 (CH_3_), 64.0 q (C^1^, *J*_C-F_ 26.6 Hz), 122.9, 125.3 q (*J*_C-F_ 1.7 Hz), 126.7, 127.0 q (CF_3_, *J*_C-F_ 282.4 Hz), 128.9, 129.1 q (*J*_C-F_ 1.7 Hz), 129.8, 130.0, 130.3, 132.5 q (*J*_C-F_ 1.7 Hz), 132.5, 133.3, 134.3, 135.0, 135.3, 136.5, 136.9, 137.1, 141.2 q (*J*_C-F_ 1.1 Hz), 142.8, 147.5. ^19^F-NMR (CDCl_3_, 376 MHz) δ, ppm: –67.63 s (CF_3_). HRMS (MALDI-TOF): C_28_H_28_F_3_ found 421.2124 [M + H]^+^; calcd. 421.2138.

**5,6-Dimethyl-3,3-diphenyl-1-(trifluoromethyl)-*1H*-indene (6a):** The reaction scale is 30 mg (0.1 mmol) alcohol **1d** and 0.4 mL benzene, isolated amount is 17 mg mixture of **6a, 4ad** and **6b** (total yield 47%, ratio **6a** : **4ad** : **6b** = 2:5:1, respectively). ^1^Н-NMR (CDCl_3_, 400 MHz, measured in a mixture with **6b**) δ, ppm: 2.27 s (3H, CH_3_), 2.31 s (3H, CH_3_), 7.12 q (1H, C^2^H, ^3^*J* 1.8 Hz), 7.12 s (1H_arom._), 7.17-7.22 m (4H_arom._), 7.22-7.32 m (7H_arom._). ^13^C-NMR (CDCl_3_, 100 MHz, measured in a mixture with **6b**) δ, ppm: 20.1 (CH_3_), 20.5 (CH_3_), 65.9 (C^3^), 122.6 q (CF_3_, *J*_C-F_ 270.3 Hz), 127.3, 127.9, 128.5, 128.7, 128.9, 132.5 q (C^1^, *J*_C-F_ 34.3 Hz), 134.5, 135.8, 136.2, 142.2, 146.9 q (C^2^, *J*_C-F_ 5.1 Hz), 149.4. ^19^F-NMR (CDCl_3_, 376 MHz, measured in a mixture with **6b**) δ, ppm: –63.87 - –63.88 s (CF_3_). HRMS (MALDI-TOF): C_24_H_20_F_3_ found 365.1519 [M + H]^+^; calcd. 365.1512.

**4,5-Dimethyl-3,3-diphenyl-1-(trifluoromethyl)-*1H*-indene (6b):** The reaction scale is 30 mg (0.1 mmol) alcohol **1d** and 0.4 mL benzene, isolated amount is 17 mg mixture of **6b, 4ad** and **6a** (total yield 47%, ratio **6b** : **4ad** : **6a** = 1:5:2, respectively). ^1^Н-NMR (CDCl_3_, 400 MHz, measured in a mixture with **6a**) δ, ppm: 1.88 s (3H, CH_3_), 2.26 s (3H, CH_3_), 6.98 q (1H, C^2^H, ^3^*J* 1.7 Hz), 7.17-7.22 m (3H_arom._), 7.22-7.32 m (9H_arom._). ^13^C-NMR (CDCl_3_, 100 MHz, measured in a mixture with **6a**) δ, ppm: 16.5 (CH_3_), 20.2 (CH_3_), 66.5 (C^3^), 118.9, 122.64 q (CF_3_, *J*_C-F_ 270.4 Hz), 122.65, 127.0, 127.3, 130.0, 131.3 q (C^1^, *J*_C-F_ 34.3 Hz), 135.1 q (*J*_C-F_ 1.0 Hz), 136.6, 137.9, 142.2, 145.0 q (C^2^, *J*_C-F_ 5.1 Hz), 148.0. ^19^F-NMR (CDCl_3_, 376 MHz, measured in a mixture with **6a**) δ, ppm: –63.98–−63.99 m (CF_3_). HRMS (MALDI-TOF): C_24_H_20_F_3_ found 365.1519 [M + H]^+^; calcd. 365.1512.

**References**

1.	Dolomanov, O. V.; Bourhis, L. J.; Gildea, R. J.; Howard, J. A. K.; Puschmann, H. *J. Appl. Cryst*. **2009**, *42*, 339-341.

2.	*SHELXS*; Sheldrick, G. M. *Acta Cryst.* **2008**, *A64*, 112-122.

3.	Frisch, M. J.; Trucks, G. W.; Schlegel, H. B.; Scuseria, G. E.; Robb, M. A.; Cheeseman, J. R.; Montgomery Jr., J. A.; Vreven, T.; Kudin, K. N.; Burant, J. C.; Millam, J. M.; Iyengar, S. S.; Tomasi, J.; Barone, V.; Mennucci, B.; Cossi, M.; Scalmani, G.; Rega, N.; Petersson, G. A.; Nakatsuji, H.; Hada, M.; Ehara, M.; Toyota, K.; Fukuda, R.; Hasegawa, J.; Ishida, M.; Nakajima, T.; Honda, Y.; Kitao, O.; Nakai, H.; Klene, M.; Li, X.; Knox, J. E.; Hratchian, H. P.; Cross, J. B.; Adamo, C.; Jaramillo, J.; Gomperts, R.; Stratmann, R. E.; Yazyev, O.; Austin, A. J.; Cammi, R.; Pomelli, C.; Ochterski, J. W.; Ayala, P. Y.; Morokuma, K.; Voth, G. A.; Salvador, P.; Dannenberg, J. J.; Zakrzewski, V. G.; Dapprich, S.; Daniels, A. D.; Strain, M. C.; Farkas, O.; Malick, D. K.; Rabuck, D.; Raghavachari, K.; Foresman, J. B.; Ortiz, J. V.; Cui, Q.; Baboul, A. G.; Clifford, S.; Cioslowski, J.; Stefanov, B. B.; Liu, G.; Liashenko, A.; Piskorz, P.; Komaromi, I.; Martin, R. L.; Fox, D. J.; Keith, T.; Al-Laham, M. A.; Peng, C. Y.; Nanayakkara, A.; Challacombe, M.; Gill, P. M. W.; Johnson, B.; Chen, W.; Wong, M. W.; Gonzalez, C.; Pople, J. A. *GAUSSIAN 03, Revision D.02*; Gaussian, Inc., Wallingford CT, **2004**.

4.	Roy, S.; Davydova, M. P.; Pal, R.; Gilmore, K.; Tolstikov, G. A.; Vasilevsky, S. F.; Alabugin I. V. |*J. Org. Chem.* **2011**, *76*, 7482–7490.

5.	Large, S.; Roques, N.; Langlois, B. R. *J. Org. Chem.* **2000**, *65*, 8848–8856.

6.	Singh, R. P.; Kirchmeier, R. L.; Shreeve, J. M. *Org. Lett.* **1999**, *1*, 1047–1049.

7.	Cort, A. D. *Synth. Commun.* **1990**, *20*, 757–760.

8.	Correia, C.A.; McQuade, D.T.; Seeberger, P.H. *Adv. Synth. Catal.* **2013**, *355*, 3517-3521.

9.	Motoki, R.; Kanai, M.; Shibasaki, M. *Org. Lett.* **2007**, *9* (16), 2997-3000.

10.	Zhang, G.-W.; Meng, W.; Ma, H.; Nie, J.; Zhang, W.-Q.; Ma, J.-A. *Angew. Chem. Int. Ed.* **2011**, *50*, 3538-3542.

11.	Wang, L.; Liu, N.; Dai, B.; Ma, X.; Shi, L. *RSC Adv.* **2015**, *5,* 10089-10093.

12.	Wang, H.; Yang, K.-F.; Li, L.; Bai, Y.; Zheng, Z.-J.; Zhang, W.-Q.; Gao, Z.-W.; Xu L.-W. *ChemCatChem* **2014**, *6*, 580-591.
