# Supplementary material for: TfOH-Promoted Reaction of 2,4-Diaryl-1,1,1-Trifluorobut-3-yn-2-oles with Arenes: Synthesis of 1,3-Diaryl-1-CF3-Indenes and Versatility of the Reaction Mechanisms
Source: Molecules. 2018 Nov 25;23(12):3079. doi: 10.3390/molecules23123079 (PMC6321356; doi:10.3390/molecules23123079)
Supplement: Supplementary file 1 [file molecules-23-03079-s001.zip › supplyment/SI part 3-1-edited.doc]

CH_3_

CH_3_

C^4^H

C^7^H

H_arom_

C^2^H

C^4^H

H_arom_

H_arom_

H_arom_

Figure S411. ^1^Н-NMR spectrum of the compound **4fa** (CDCl_3_, 400 MHz).

CH_3_

C^1^

C^1^

CF_3_

Figure S412. ^13^C-NMR spectrum of the compound **4fa** (CDCl_3_, 100 MHz).

Figure S413. ^19^F-NMR spectrum of the compound **4fa** (CDCl_3_, 376 MHz).

CH_3_

CH_3_

CH_3_

CH_3_

C^2^H

C^4^H

C^7^H

H_arom_

H_arom_

H_arom_

C^2^H

C^4^H

H_arom_

H_arom_

H_arom_

H_arom_

Figure S414. ^1^Н-NMR spectrum of the compounds **4fa** and **3a** (CDCl_3_, 400 MHz).

C^1^

CH_3_

CH_3_

C^1^

C^2^

C^3^

C^3^

C^4^

C^4^

C^1^

CF_3_

CH_3_

CH_3_

Figure S415. ^13^C-NMR spectrum of the compounds **4fa** and **3a** (CDCl_3_, 100 MHz).

CF_3_

CF_3_

CF_3_

CF_3_

Figure S416. ^19^F-NMR spectrum of the compounds **4fa** and **3a** (CDCl_3_, 376 MHz).

Figure S417. NOESY H-H spectrum of the compounds **4fa** and **3a** (CDCl_3_, 400 MHz).

Figure S418. NOESY H-F spectrum of the compounds **4fa** and **3a** (CDCl_3_, 400 MHz).

CH_3_

CH_3_

CH_3_

CH_3_

C^4^H

H_arom_

H_arom_

H_arom_

C^2^H

C^4^H

H_arom_

H_arom_

H_arom_

H_arom_

H_arom_

H_arom_

H_arom_

H_arom_

H_arom_

Figure S419. ^1^Н-NMR spectrum of the compounds **4fb** and **3b** (CDCl_3_, 400 MHz).

CH_3_

CH_3_

C^1^

CH_3_

CH_3_

C^3^

C^4^

C^1^

CF_3_

C^1^

Figure S420. ^13^C-NMR spectrum of the compounds **4fb** and **3b** (CDCl_3_, 100 MHz).

CH_3_

CH_3_

CH_3_

CH_3_

CH_3_

CH_3_

Figure S421. DEPT spectrum of the compounds **4fb** and **3b** (CDCl_3_, 100 MHz).

CF_3_

CF_3_

CF_3_

CF_3_

Figure S422. ^19^F-NMR spectrum of the compounds **4fb** and **3b** (CDCl_3_, 376 MHz).

Figure S423. NOESY H-H spectrum of the compounds **4fb** and **3b** (CDCl_3_, 400 MHz).

Figure S424. NOESY H-F spectrum of the compounds **4fb** and **3b** (CDCl_3_, 400 MHz).

CH_3_

CH_3_

CH_3_

CH_3_

C^4^H

C^7^H

H_arom_

H_arom_

H_arom_

C^2^H

C^4^H

H_arom_

H_arom_

H_arom_

H_arom_

CH_3_

CH_3_

H_arom_

H_arom_

H_arom_

H_arom_

Figure S425. ^1^Н-NMR spectrum of the compounds **4fc** and **3c** (CDCl_3_, 400 MHz).

CH_3_

CH_3_

C^1^

CH_3_

CH_3_

C^1^

C^2^

C^3^

C^3^

C^4^

C^4^

C^1^

CF_3_

Figure S426. ^13^C-NMR spectrum of the compounds **4fc** and **3c** (CDCl_3_, 100 MHz).

CH_3_

CH_3_

CH_3_

CH_3_

CH_3_

CH_3_

Figure S427. DEPT spectrum of the compounds **4fc** and **3c** (CDCl_3_, 100 MHz).

CF_3_

CF_3_

CF_3_

CF_3_

Figure S428. ^19^F-NMR spectrum of the compounds **4fc** and **3c** (CDCl_3_, 376 MHz).

Figure S429. COSY H-H spectrum of the compounds **4fc** and **3c** (CDCl_3_, 400 MHz).

Figure S430. NOESY H-H spectrum of the compounds **4fc** and **3c** (CDCl_3_, 400 MHz).

Figure S431. NOESY H-F spectrum of the compounds **4fc** and **3c** (CDCl_3_, 400 MHz).

CH_3_

CH_3_

CH_3_

CH_3_

C^4^H

C^7^H

H_arom_

H_arom_

H_arom_

C^2^H

C^4^H

H_arom_

H_arom_

H_arom_

H_arom_

CH_3_

CH_3_

H_arom_

H_arom_

H_arom_

Figure S432. ^1^Н-NMR spectrum of the compounds **4fd** and **3d** (CDCl_3_, 400 MHz).

CH_3_

CH_3_

C^1^

CH_3_

CH_3_

C^1^

C^3^

C^4^

C^1^

CF_3_

CH_3_

Figure S433. ^13^C-NMR spectrum of the compounds **4fd** and **3d** (CDCl_3_, 100 MHz).

CH_3_

CH_3_

CH_3_

CH_3_

CH_3_

CH_3_

Figure S434. DEPT spectrum of the compounds **4fd** and **3d** (CDCl_3_, 100 MHz).

CF_3_

CF_3_

CF_3_

CF_3_

Figure S435. ^19^F-NMR spectrum of the compounds **4fd** and **3d** (CDCl_3_, 376 MHz).

Figure S436. NOESY H-H spectrum of the compounds **4fd** and **3d** (CDCl_3_, 400 MHz).

Figure S437. NOESY H-F spectrum of the compounds **4fd** and **3d** (CDCl_3_, 400 MHz).

CH_3_

CH_3_

CH_3_

CH_3_

C^4^H

C^7^H

H_arom_

H_arom_

H_arom_

C^2^H

C^4^H

H_arom_

H_arom_

H_arom_

H_arom_

H_arom_

H_arom_

Figure S438. ^1^Н-NMR spectrum of the compounds **4fe** and **3e** (CDCl_3_, 400 MHz).

CH_3_

CH_3_

C^1^

CH_3_

CH_3_

C^1^

C^3^

C^4^

C^1^

CF_3_

Figure S439. ^13^C-NMR spectrum of the compounds **4fe** and **3e** (CDCl_3_, 100 MHz).

CH_3_

CH_3_

CH_3_

CH_3_

Figure S440. DEPT spectrum of the compounds **4fe** and **3e** (CDCl_3_, 100 MHz).

CF_3_

CF_3_

CF_3_

CF_3_

Figure S441. ^19^F-NMR spectrum of the compounds **4fe** and **3e** (CDCl_3_, 376 MHz).

Figure S442. NOESY H-H spectrum of the compounds **4fe** and **3e** (CDCl_3_, 400 MHz).

Figure S443. NOESY H-F spectrum of the compounds **4fe** and **3e** (CDCl_3_, 400 MHz).

CH_3_

CH_3_

CH_3_

CH_3_

C^4^H

C^7^H

H_arom_

H_arom_

H_arom_

C^2^H

C^4^H

H_arom_

H_arom_

H_arom_

H_arom_

H_arom_

H_arom_

H_arom_

CH_3_

Figure S444. ^1^Н-NMR spectrum of the compounds **4ff** and **3f** (CDCl_3_, 400 MHz).

CH_3_

CH_3_

C^1^

C^3^

C^4^

C^1^

CF_3_

CH_3_

CH_3_

C^1^

Figure S445. ^13^C-NMR spectrum of the compounds **4ff** and **3f** (CDCl_3_, 100 MHz).

CH_3_

CH_3_

CH_3_

CH_3_

Figure S446. DEPT spectrum of the compounds **4ff** and **3f** (CDCl_3_, 100 MHz).

CF_3_

CF_3_

CF_3_

CF_3_

Figure S447. ^19^F-NMR spectrum of the compounds **4ff** and **3f** (CDCl_3_, 376 MHz).

Figure S448. NOESY H-H spectrum of the compounds **4ff** and **3f** (CDCl_3_, 400 MHz).

Figure S449. NOESY H-F spectrum of the compounds **4ff** and **3f** (CDCl_3_, 400 MHz).

CH_3_

CH_3_

C^4^H

C^7^H

H_arom_

C^2^H

C^4^H

H_arom_

H_arom_

H_arom_

H_arom_

H_arom_

Figure S450. ^1^Н-NMR spectrum of the compound **4fg** (CDCl_3_, 400 MHz).

CH_3_

C^1^

CF_3_

C^1^

CH_3_

Figure S451. ^13^C-NMR spectrum of the compound **4fg** (CDCl_3_, 100 MHz).

CH_3_

Figure S452. DEPT spectrum of the compound **4fg** (CDCl_3_, 100 MHz).

Figure S453. ^19^F-NMR spectrum of the compound **4fg** (CDCl_3_, 376 MHz).

CH_3_

CH_3_

C^4^H

C^7^H

H_arom_

C^2^H

C^4^H

H_arom_

H_arom_

H_arom_

CH_3_

CH_3_

CH_3_

H_arom_

H_arom_

H_arom_

H_arom_

H_arom_

H_arom_

H_arom_

Figure S454. ^1^Н-NMR spectrum of the compounds **4fh** and **3g** (CDCl_3_, 400 MHz).

CH_3_

C^1^

CF_3_

CH_3_

C^1^

Figure S455. ^13^C-NMR spectrum of the compound **4fh** (CDCl_3_, 100 MHz).

CH_3_

CH_3_

Figure S456. DEPT spectrum of the compound **4fh** (CDCl_3_, 100 MHz).

CF_3_

CF_3_

CF_3_

CF_3_

Figure S457. ^19^F-NMR spectrum of the compounds **4fh** and **3g** (CDCl_3_, 376 MHz).

Figure S458. NOESY H-H spectrum of the compounds **4fh** and **3g** (CDCl_3_, 400 MHz).

CH_3_

CH_3_

C^7^H

H_arom_

C^2^H

C^4^H

H_arom_

H_arom_

H_arom_

CH_3_

CH_3_

H_arom_

H_arom_

Figure S459. ^1^Н-NMR spectrum of the compound **4fi** (CDCl_3_, 400 MHz).

CH_3_

C^1^

C^1^

CF_3_

CH_3_

CH_3_

Figure S460. ^13^C-NMR spectrum of the compound **4fi** (CDCl_3_, 100 MHz).

CH_3_

CH_3_

CH_3_

Figure S461. DEPT spectrum of the compound **4fi** (CDCl_3_, 100 MHz).

Figure S462. ^19^F-NMR spectrum of the compound **4fi** (CDCl_3_, 376 MHz).

CH_3_

CH_3_

C^4^H

C^7^H

H_arom_

C^2^H

C^4^H

H_arom_

H_arom_

H_arom_

CH_3_

Figure S463. ^1^Н-NMR spectrum of the compound **4fj** (CDCl_3_, 400 MHz).

CH_3_

C^1^

C^1^

CF_3_

CH_3_

Figure S464. ^13^C-NMR spectrum of the compound **4fj** (CDCl_3_, 100 MHz).

CH_3_

CH_3_

Figure S465. DEPT spectrum of the compound **4fj** (CDCl_3_, 100 MHz).

Figure S466. ^19^F-NMR spectrum of the compound **4fj** (CDCl_3_, 376 MHz).

CH_3_

CH_3_

C^4^H

C^7^H

H_arom_

C^2^H

C^4^H

H_arom_

H_arom_

H_arom_

Figure S467. ^1^Н-NMR spectrum of the compound **4fk** (CDCl_3_, 400 MHz).

CH_3_

C^1^

CF_3_

C^1^

Figure S468. ^13^C-NMR spectrum of the compound **4fk** (CDCl_3_, 100 MHz).

CH_3_

Figure S469. DEPT spectrum of the compound **4fk** (CDCl_3_, 100 MHz).

Figure S470. ^19^F-NMR spectrum of the compound **4fk** (CDCl_3_, 376 MHz).

CH_3_

CH_3_

CH_3_

CH_3_

C^4^H

C^7^H

H_arom_

H_arom_

H_arom_

C^2^H

C^4^H

H_arom_

H_arom_

H_arom_

H_arom_

H_arom_

H_arom_

H_arom_

Figure S471. ^1^Н-NMR spectrum of the compounds **4fk** and **3h** (CDCl_3_, 400 MHz).

CH_3_

CH_3_

C^1^

C^3^

C^3^

C^4^

C^4^

C^1^

CF_3_

Figure S472. ^13^C-NMR spectrum of the compounds **4fk** and **3h** (CDCl_3_, 100 MHz).

CH_3_

CH_3_

Figure S473. DEPT spectrum of the compounds **4fk** and **3h** (CDCl_3_, 100 MHz).

CF_3_

CF_3_

CF_3_

CF_3_

Figure S474. ^19^F-NMR spectrum of the compounds **4fk** and **3h** (CDCl_3_, 376 MHz).

CH_3_

CH_3_

H_arom_

C^2^H

H_arom_

H_arom_

H_arom_

CH_3_

CH_3_

H_arom_

H_arom_

H_arom_

H_arom_

H_arom_

H_arom_

H_arom_

Figure S475. ^1^Н-NMR spectrum of the compounds **4fl** and **3i** (CDCl_3_, 400 MHz).

CH_3_

C^1^

CF_3_

C^1^

CH_3_

CH_3_

CH_3_

CF_3_

C^4^

C^3^

Figure S476. ^13^C-NMR spectrum of the compounds **4fl** and **3i** (CDCl_3_, 100 MHz).

CH_3_

CH_3_

CH_3_

CH_3_

Figure S477. DEPT spectrum of the compounds **4fl** and **3i** (CDCl_3_, 100 MHz).

CF_3_

CF_3_

CF_3_

CF_3_

Figure S478. ^19^F-NMR spectrum of the compounds **4fl** and **3i** (CDCl_3_, 376 MHz).

Figure S479. NOESY H-H spectrum of the compounds **4fl** and **3i** (CDCl_3_, 400 MHz).

Figure S480. NOESY H-F spectrum of the compounds **4fl** and **3i** (CDCl_3_, 400 MHz).

CH_3_

CH_3_

C^4^H

C^7^H

H_arom_

C^2^H

C^4^H

H_arom_

H_arom_

H_arom_

CH_3_

CH_3_

H_arom_

Figure S481. ^1^Н-NMR spectrum of the compound **4fm** (CDCl_3_, 400 MHz).

CH_3_

C^1^

C^1^

CF_3_

CH_3_

Figure S482. ^13^C-NMR spectrum of the compound **4fm** (CDCl_3_, 100 MHz).

CH_3_

CH_3_

Figure S483. DEPT spectrum of the compound **4fm** (CDCl_3_, 100 MHz).

Figure S484. ^19^F-NMR spectrum of the compound **4fm** (CDCl_3_, 376 MHz).

CH_3_

CH_3_

CH_3_

CH_3_

C^4^H

C^7^H

H_arom_

H_arom_

H_arom_

C^2^H

C^4^H

H_arom_

H_arom_

H_arom_

H_arom_

H_arom_

H_arom_

Figure S485. ^1^Н-NMR spectrum of the compounds **4fn** and **3j** (CDCl_3_, 400 MHz).

CH_3_

CH_3_

C^1^

CH_3_

CH_3_

C^1^

C^2^

C^3^

C^4^

C^1^

CF_3_

Figure S486. ^13^C-NMR spectrum of the compounds **4fn** and **3j** (CDCl_3_, 100 MHz).

CH_3_

CH_3_

CH_3_

CH_3_

Figure S487. ^13^C-NMR spectrum of the compounds **4fn** and **3j** (CDCl_3_, 100 MHz).

CF_3_

CF_3_

CF_3_

CF_3_

Figure S488. ^19^F-NMR spectrum of the compounds **4fn** and **3j** (CDCl_3_, 376 MHz).

CH_3_

CH_3_

C^4^H

C^7^H

H_arom_

C^2^H

C^4^H

H_arom_

H_arom_

H_arom_

Figure S489. ^1^Н-NMR spectrum of the compound **4fo** (CDCl_3_, 400 MHz).

CH_3_

C^1^

CF_3_

C^1^

Figure S490. ^13^C-NMR spectrum of the compound **4fo** (CDCl_3_, 100 MHz).

Figure S491. ^19^F-NMR spectrum of the compound **4fo** (CDCl_3_, 376 MHz).

CH_3_

CH_3_

CH_3_

CH_3_

C^4^H

C^7^H

H_arom_

H_arom_

H_arom_

C^2^H

H_arom_

H_arom_

H_arom_

H_arom_

H_arom_

H_arom_

H_arom_

CH_3_

Figure S492. ^1^Н-NMR spectrum of the compounds **4fo** and **3k** (CDCl_3_, 400 MHz).

CH_3_

CH_3_

C^1^

C^3^

C^4^

C^1^

CF_3_

CH_3_

CH_3_

C^2^

C^3^

Figure S493. ^13^C-NMR spectrum of the compounds **4fo** and **3k** (CDCl_3_, 100 MHz).

CH_3_

CH_3_

Figure S494. DEPT spectrum of the compounds **4fo** and **3k** (CDCl_3_, 100 MHz).

CF_3_

CF_3_

CF_3_

CF_3_

Figure S495. ^19^F-NMR spectrum of the compounds **4fo** and **3k** (CDCl_3_, 376 MHz).

CH_3_

CH_3_

C^2^H

H_arom._

H_arom._

H_arom._

H_arom._

Figure S496. ^1^Н-NMR spectrum of the compound **5ac** (CDCl_3_, 400 MHz).

CH_3_

CH_3_

C^1^

C^1^

CF_3_

Figure S497. ^13^C-NMR spectrum of the compound **5ac** (CDCl_3_, 100 MHz).

CH_3_

CH_3_

Figure S498. DEPT spectrum of the compound **5ac** (CDCl_3_, 100 MHz).

Figure S499. ^19^F-NMR spectrum of the compound **5ac** (CDCl_3_, 376 MHz).

C^2^H

H_arom._

H_arom._

H_arom._

H_arom._

H_arom._

H_arom._

CH_3_

CH_3_

Figure S500. ^1^Н-NMR spectrum of the compound **5ad** (CDCl_3_, 400 MHz, measured in a mixture with **5ac**).

C^1^

C^1^

CF_3_

CH_3_

CH_3_

CH_3_

Figure S501. ^13^C-NMR spectrum of the compound **5ad** (CDCl_3_, 100 MHz, measured in a mixture with **5ac**).

Figure S502. ^19^F-NMR spectrum of the compound **5ad** (CDCl_3_, 376 MHz, measured in a mixture with **5ac**).

Figure S503. NOESY H-F spectrum of the compound **5ad** (CDCl_3_, 400 MHz, measured in a mixture with **5ac**).

CH_3_

CH_3_

C^2^H

H_arom._

H_arom._

H_arom._

H_arom._

H_arom._

Figure S504. ^1^Н-NMR spectrum of the compound **5bb** (CDCl_3_, 400 MHz).

CH_3_

C^1^

CH_3_

C^1^

CF_3_

Figure S505. ^13^C-NMR spectrum of the compound **5bb** (CDCl_3_, 100 MHz).

CH_3_

CH_3_

Figure S506. DEPT spectrum of the compound **5bb** (CDCl_3_, 100 MHz).

Figure S507. ^19^F-NMR spectrum of the compound **5bb** (CDCl_3_, 376 MHz).

CH_3_

CH_3_

C^2^H

H_arom._

H_arom._

H_arom._

CH_3_

H_arom._

H_arom._

H_arom._

Figure S508. ^1^Н-NMR spectrum of the compound **5bd** (CDCl_3_, 400 MHz).

CH_3_

CH_3_

C^1^

CH_3_

C^1^

CF_3_

Figure S509. ^13^C-NMR spectrum of the compound **5bd** (CDCl_3_, 100 MHz).

CH_3_

CH_3_

CH_3_

Figure S510. DEPT spectrum of the compound **5bd** (CDCl_3_, 100 MHz).

Figure S511. ^19^F-NMR spectrum of the compound **5bd** (CDCl_3_, 376 MHz).

Figure S512. NOESY H-H spectrum of the compound **5bd** (CDCl_3_, 400 MHz).

Figure S513. NOESY H-F spectrum of the compound **5bd** (CDCl_3_, 400 MHz).

C^2^H

H_arom._

H_arom._

CH_3_

H_arom._

H_arom._

CH_3_

H_arom._

H_arom._

C^2^H

CH_3_

CH_3_

C^2^H

H_arom._

Figure S514. ^1^Н-NMR spectrum of the compounds **6a** and **6b** (CDCl_3_, 400 MHz).

C^3^

C^3^

CF_3_

CH_3_

CH_3_

CH_3_

CH_3_

CH_3_

CF_3_

C^1^

C^1^

C^2^

C^2^

Figure S515. ^13^C-NMR spectrum of the compounds **6a** and **6b** (CDCl_3_, 100 MHz).

CH_3_

CH_3_

CH_3_

C^2^

C^2^

Figure S516. DEPT spectrum of the compounds **6a** and **6b** (CDCl_3_, 100 MHz).

CF_3_

CF_3_

CF_3_

CF_3_

Figure S517. ^19^F-NMR spectrum of the compounds **6a** and **6b** (CDCl_3_, 376 MHz).

Figure S518. NOESY H-H spectrum of the compounds **6a** and **6b** (CDCl_3_, 400 MHz).

Figure S519. NOESY H-F spectrum of the compounds **6a** and **6b** (CDCl_3_, 400 MHz).

**X-ray data**

| **Table S1.Crystal data and structure refinement for 4bg** | |
| --- | --- |
| Empirical formula | C_24_H_18_BrF_3_ |
| Formula weight | 443.29 |
| Temperature/K | 100(2) |
| Crystal system | monoclinic |
| Space group | P2_1_/c |
| a/Å | 17.9133(7) |
| b/Å | 7.8377(4) |
| c/Å | 15.1333(6) |
| α/° | 90 |
| β/° | 112.505(5) |
| γ/° | 90 |
| Volume/Å^3^ | 1962.88(16) |
| Z | 4 |
| ρ_calc_g/cm^3^ | 1.500 |
| μ/mm^−1^ | 2.128 |
| F(000) | 896.0 |
| Crystal size/mm^3^ | 0.4 × 0.3 × 0.2 |
| Radiation | MoKα (λ = 0.71073) |
| 2Θ range for data collection/° | 5.752 to 54.996 |
| Index ranges | -23 ≤ h ≤ 22, -8 ≤ k ≤ 9, −19 ≤ l ≤ 18 |
| Reflections collected | 8335 |
| Independent reflections | 4356 [R_int_ = 0.0275, R_sigma_ = 0.0462] |
| Data/restraints/parameters | 4356/0/255 |
| Goodness-of-fit on F^2^ | 1.042 |
| Final R indexes [I>=2σ (I)] | R_1_ = 0.0357, wR_2_ = 0.0775 |
| Final R indexes [all data] | R_1_ = 0.0487, wR_2_ = 0.0838 |
| Largest diff. peak/hole / e Å^-3^ | 0.43/−0.42 |

| **Table S2. Crystal data and structure refinement for 4ci** | |
| --- | --- |
| Empirical formula | C_24_H_18_ClF_3_ |
| Formula weight | 398.83 |
| Temperature/K | 100(2) |
| Crystal system | triclinic |
| Space group | P-1 |
| a/Å | 9.2142(5) |
| b/Å | 9.6055(5) |
| c/Å | 11.9737(6) |
| α/° | 108.499(5) |
| β/° | 97.360(5) |
| γ/° | 103.207(5) |
| Volume/Å^3^ | 955.05(10) |
| Z | 2 |
| ρ_calc_g/cm^3^ | 1.387 |
| μ/mm^−1^ | 0.234 |
| F(000) | 412.0 |
| Crystal size/mm^3^ | 0.3 × 0.25 × 0.15 |
| Radiation | MoKα (λ = 0.71073) |
| 2Θ range for data collection/° | 5.568 to 54.992 |
| Index ranges | −11 ≤ h ≤ 11, −11 ≤ k ≤ 12, −15 ≤ l ≤ 15 |
| Reflections collected | 7734 |
| Independent reflections | 4387 [R_int_ = 0.0214, R_sigma_ = 0.0401] |
| Data/restraints/parameters | 4387/0/255 |
| Goodness-of-fit on F^2^ | 1.050 |
| Final R indexes [I>=2σ (I)] | R_1_ = 0.0405, wR_2_ = 0.0877 |
| Final R indexes [all data] | R_1_ = 0.0517, wR_2_ = 0.0960 |
| Largest diff. peak/hole / e Å^-3^ | 0.34/−0.27 |

| **Table S3. Crystal data and structure refinement for 4dc** | |
| --- | --- |
| Empirical formula | C_26_H_23_F_3_ |
| Formula weight | 392.44 |
| Temperature/K | 100(2) |
| Crystal system | monoclinic |
| Space group | P2_1_/c |
| a/Å | 9.5745(4) |
| b/Å | 18.8280(7) |
| c/Å | 11.7040(5) |
| α/° | 90 |
| β/° | 105.638(4) |
| γ/° | 90 |
| Volume/Å^3^ | 2031.76(15) |
| Z | 4 |
| ρ_calc_g/cm^3^ | 1.283 |
| μ/mm^−1^ | 0.092 |
| F(000) | 824.0 |
| Crystal size/mm^3^ | 0.2 × 0.15 × 0.1 |
| Radiation | MoKα (λ = 0.71073) |
| 2Θ range for data collection/° | 5.638 to 54.994 |
| Index ranges | −12 ≤ h ≤ 12, −12 ≤ k ≤ 24, -9 ≤ l ≤ 15 |
| Reflections collected | 9663 |
| Independent reflections | 4639 [R_int_ = 0.0274, R_sigma_ = 0.0439] |
| Data/restraints/parameters | 4639/0/266 |
| Goodness-of-fit on F^2^ | 1.008 |
| Final R indexes [I>=2σ (I)] | R_1_ = 0.0465, wR_2_ = 0.0977 |
| Final R indexes [all data] | R_1_ = 0.0669, wR_2_ = 0.1071 |
| Largest diff. peak/hole / e Å^-3^ | 0.34/−0.25 |

| **Table S4. Crystal data and structure refinement for 4de** | |
| --- | --- |
| Empirical formula | C_25_H_20_ClF_3_ |
| Formula weight | 412.86 |
| Temperature/K | 100(2) |
| Crystal system | monoclinic |
| Space group | P2_1_/c |
| a/Å | 9.5351(3) |
| b/Å | 18.8209(5) |
| c/Å | 11.7111(4) |
| α/° | 90 |
| β/° | 105.722(4) |
| γ/° | 90 |
| Volume/Å^3^ | 2023.04(12) |
| Z | 4 |
| ρ_calc_g/cm^3^ | 1.356 |
| μ/mm^−1^ | 0.224 |
| F(000) | 856.0 |
| Crystal size/mm^3^ | 0.3 × 0.3 × 0.3 |
| Radiation | MoKα (λ = 0.71073) |
| 2Θ range for data collection/° | 5.362 to 56.99 |
| Index ranges | −11 ≤ h ≤ 12, -25 ≤ k ≤ 25, −15 ≤ l ≤ 15 |
| Reflections collected | 19954 |
| Independent reflections | 5120 [R_int_ = 0.0304, R_sigma_ = 0.0277] |
| Data/restraints/parameters | 5120/0/265 |
| Goodness-of-fit on F^2^ | 1.044 |
| Final R indexes [I>=2σ (I)] | R_1_ = 0.0412, wR_2_ = 0.0975 |
| Final R indexes [all data] | R_1_ = 0.0517, wR_2_ = 0.1037 |
| Largest diff. peak/hole / e Å^-3^ | 0.57/−0.36 |

| **Table S5. Crystal data and structure refinement for 4dg** | |
| --- | --- |
| Empirical formula | C_25_H_20_BrF_3_ |
| Formula weight | 457.32 |
| Temperature/K | 100(2) |
| Crystal system | monoclinic |
| Space group | P2_1_/c |
| a/Å | 8.5039(4) |
| b/Å | 11.7696(7) |
| c/Å | 20.4616(11) |
| α/° | 90 |
| β/° | 97.457(5) |
| γ/° | 90 |
| Volume/Å^3^ | 2030.6(2) |
| Z | 4 |
| ρ_calc_g/cm^3^ | 1.496 |
| μ/mm^−1^ | 2.059 |
| F(000) | 928.0 |
| Crystal size/mm^3^ | 0.35 × 0.35 × 0.25 |
| Radiation | MoKα (λ = 0.71073) |
| 2Θ range for data collection/° | 5.868 to 54.994 |
| Index ranges | −10 ≤ h ≤ 11, −15 ≤ k ≤ 13, −26 ≤ l ≤ 17 |
| Reflections collected | 9899 |
| Independent reflections | 4660 [R_int_ = 0.0332, R_sigma_ = 0.0533] |
| Data/restraints/parameters | 4660/0/265 |
| Goodness-of-fit on F^2^ | 1.048 |
| Final R indexes [I>=2σ (I)] | R_1_ = 0.0428, wR_2_ = 0.0918 |
| Final R indexes [all data] | R_1_ = 0.0605, wR_2_ = 0.1012 |
| Largest diff. peak/hole / e Å^-3^ | 0.93/−0.54 |

| **Table S6. Crystal data and structure refinement for 4di** | |
| --- | --- |
| Empirical formula | C_26_H_23_F_3_ |
| Formula weight | 392.44 |
| Temperature/K | 100(2) |
| Crystal system | triclinic |
| Space group | P-1 |
| a/Å | 9.2810(8) |
| b/Å | 9.9509(10) |
| c/Å | 12.2245(8) |
| α/° | 107.911(8) |
| β/° | 97.959(7) |
| γ/° | 105.707(9) |
| Volume/Å^3^ | 1003.39(16) |
| Z | 2 |
| ρ_calc_g/cm^3^ | 1.299 |
| μ/mm^−1^ | 0.093 |
| F(000) | 412.0 |
| Crystal size/mm^3^ | 0.23 × 0.17 × 0.15 |
| Radiation | MoKα (λ = 0.71073) |
| 2Θ range for data collection/° | 6.162 to 55 |
| Index ranges | −11 ≤ h ≤ 12, −11 ≤ k ≤ 12, −15 ≤ l ≤ 11 |
| Reflections collected | 8106 |
| Independent reflections | 4582 [R_int_ = 0.0204, R_sigma_ = 0.0378] |
| Data/restraints/parameters | 4582/0/266 |
| Goodness-of-fit on F^2^ | 1.106 |
| Final R indexes [I>=2σ (I)] | R_1_ = 0.0573, wR_2_ = 0.1309 |
| Final R indexes [all data] | R_1_ = 0.0672, wR_2_ = 0.1372 |
| Largest diff. peak/hole / e Å^-3^ | 0.62/−0.29 |

| **Table S7. Crystal data and structure refinement for 4dk** | |
| --- | --- |
| Empirical formula | C_25_H_20_BrF_3_ |
| Formula weight | 457.32 |
| Temperature/K | 100(2) |
| Crystal system | triclinic |
| Space group | P-1 |
| a/Å | 9.2255(5) |
| b/Å | 9.9831(4) |
| c/Å | 12.2087(5) |
| α/° | 106.806(4) |
| β/° | 97.751(4) |
| γ/° | 106.169(4) |
| Volume/Å^3^ | 1005.03(8) |
| Z | 2 |
| ρ_calc_g/cm^3^ | 1.511 |
| μ/mm^−1^ | 2.080 |
| F(000) | 464.0 |
| Crystal size/mm^3^ | 0.35 × 0.3 × 0.3 |
| Radiation | MoKα (λ = 0.71073) |
| 2Θ range for data collection/° | 6.178 to 56.996 |
| Index ranges | −12 ≤ h ≤ 12, −13 ≤ k ≤ 13, −16 ≤ l ≤ 16 |
| Reflections collected | 17908 |
| Independent reflections | 5099 [R_int_ = 0.0335, R_sigma_ = 0.0338] |
| Data/restraints/parameters | 5099/0/265 |
| Goodness-of-fit on F^2^ | 1.036 |
| Final R indexes [I >= 2σ (I)] | R_1_ = 0.0358, wR_2_ = 0.0799 |
| Final R indexes [all data] | R_1_ = 0.0460, wR_2_ = 0.0844 |
| Largest diff. peak/hole / e Å^-3^ | 0.91/−0.85 |

| **Table S8.  Crystal data and structure refinement for 4dm** | |
| --- | --- |
| Empirical formula | C_27_H_25_F_3_ |
| Formula weight | 406.47 |
| Temperature/K | 100(2) |
| Crystal system | triclinic |
| Space group | P1 |
| a/Å | 9.6392(4) |
| b/Å | 10.2186(4) |
| c/Å | 11.6616(5) |
| α/° | 73.306(4) |
| β/° | 79.826(3) |
| γ/° | 76.700(3) |
| Volume/Å^3^ | 1063.17(8) |
| Z | 2 |
| ρ_calc_g/cm^3^ | 1.270 |
| μ/mm^−1^ | 0.090 |
| F(000) | 428.0 |
| Crystal size/mm^3^ | 0.25 × 0.2 × 0.2 |
| Radiation | MoKα (λ = 0.71073) |
| 2Θ range for data collection/° | 5.482 to 55 |
| Index ranges | −12 ≤ h ≤ 12, −13 ≤ k ≤ 13, −15 ≤ l ≤ 15 |
| Reflections collected | 17185 |
| Independent reflections | 9726 [R_int_ = 0.0242, R_sigma_ = 0.0469] |
| Data/restraints/parameters | 9726/3/551 |
| Goodness-of-fit on F^2^ | 1.036 |
| Final R indexes [I>=2σ (I)] | R_1_ = 0.0425, wR_2_ = 0.0924 |
| Final R indexes [all data] | R_1_ = 0.0510, wR_2_ = 0.0982 |
| Largest diff. peak/hole / e Å^-3^ | 0.27/−0.23 |
| Flack parameter | 0.5 |

| **Table S9. Crystal data and structure refinement for 4fb** | |
| --- | --- |
| Empirical formula | C_25_H_21_F_3_O_2_ |
| Formula weight | 410.42 |
| Temperature/K | 100(2) |
| Crystal system | monoclinic |
| Space group | P2_1_/n |
| a/Å | 10.8392(4) |
| b/Å | 8.9013(3) |
| c/Å | 20.8499(10) |
| α/° | 90 |
| β/° | 90.360(4) |
| γ/° | 90 |
| Volume/Å^3^ | 2011.62(15) |
| Z | 4 |
| ρ_calc_g/cm^3^ | 1.355 |
| μ/mm^−1^ | 0.103 |
| F(000) | 856.0 |
| Crystal size/mm^3^ | 0.3 × 0.3 × 0.3 |
| Radiation | MoKα (λ = 0.71073) |
| 2Θ range for data collection/° | 5.922 to 56.998 |
| Index ranges | −14 ≤ h ≤ 13, -9 ≤ k ≤ 11, -27 ≤ l ≤ 26 |
| Reflections collected | 14256 |
| Independent reflections | 5060 [R_int_ = 0.0225, R_sigma_ = 0.0270] |
| Data/restraints/parameters | 5060/0/274 |
| Goodness-of-fit on F^2^ | 1.039 |
| Final R indexes [I>=2σ (I)] | R_1_ = 0.0416, wR_2_ = 0.0974 |
| Final R indexes [all data] | R_1_ = 0.0511, wR_2_ = 0.1032 |
| Largest diff. peak/hole / e Å^-3^ | 0.38/−0.22 |

| **Table S10. Crystal data and structure refinement for 4fc** | |
| --- | --- |
| Empirical formula | C_25_H_21_F_3_O_2_ |
| Formula weight | 410.42 |
| Temperature/K | 100(2) |
| Crystal system | monoclinic |
| Space group | P2_1_/c |
| a/Å | 18.9671(6) |
| b/Å | 9.3263(2) |
| c/Å | 23.7279(7) |
| α/° | 90 |
| β/° | 106.024(3) |
| γ/° | 90 |
| Volume/Å^3^ | 4034.2(2) |
| Z | 8 |
| ρ_calc_g/cm^3^ | 1.351 |
| μ/mm^−1^ | 0.103 |
| F(000) | 1712.0 |
| Crystal size/mm^3^ | 0.2 × 0.2 × 0.15 |
| Radiation | MoKα (λ = 0.71073) |
| 2Θ range for data collection/° | 5.428 to 54.994 |
| Index ranges | -24 ≤ h ≤ 24, −12 ≤ k ≤ 12, -30 ≤ l ≤ 30 |
| Reflections collected | 37080 |
| Independent reflections | 9257 [R_int_ = 0.0402, R_sigma_ = 0.0544] |
| Data/restraints/parameters | 9257/0/547 |
| Goodness-of-fit on F^2^ | 1.067 |
| Final R indexes [I>=2σ (I)] | R_1_ = 0.0452, wR_2_ = 0.0868 |
| Final R indexes [all data] | R_1_ = 0.0640, wR_2_ = 0.0966 |
| Largest diff. peak/hole / e Å^-3^ | 0.28/−0.24 |

| **Table S11. Crystal data and structure refinement for 4fh** | |
| --- | --- |
| Empirical formula | C_25_H_21_F_3_O_2_ |
| Formula weight | 410.42 |
| Temperature/K | 100.01(10) |
| Crystal system | monoclinic |
| Space group | P2_1_/n |
| a/Å | 11.3845(9) |
| b/Å | 8.6894(7) |
| c/Å | 20.5173(16) |
| α/° | 90 |
| β/° | 91.464(7) |
| γ/° | 90 |
| Volume/Å^3^ | 2029.0(3) |
| Z | 4 |
| ρ_calc_g/cm^3^ | 1.344 |
| μ/mm^−1^ | 0.103 |
| F(000) | 856.0 |
| Crystal size/mm^3^ | 0.2 × 0.15 × 0.15 |
| Radiation | MoKα (λ = 0.71073) |
| 2Θ range for data collection/° | 5.898 to 54.998 |
| Index ranges | −14 ≤ h ≤ 13, −11 ≤ k ≤ 9, -26 ≤ l ≤ 25 |
| Reflections collected | 11210 |
| Independent reflections | 4658 [R_int_ = 0.0379, R_sigma_ = 0.0468] |
| Data/restraints/parameters | 4658/0/274 |
| Goodness-of-fit on F^2^ | 1.026 |
| Final R indexes [I>=2σ (I)] | R_1_ = 0.0453, wR_2_ = 0.1129 |
| Final R indexes [all data] | R_1_ = 0.0577, wR_2_ = 0.1237 |
| Largest diff. peak/hole / e Å^-3^ | 0.31/−0.21 |

| **Table S12. Crystal data and structure refinement for 4fk** | |
| --- | --- |
| Empirical formula | C_24_H_18_ClF_3_O_2_ |
| Formula weight | 430.83 |
| Temperature/K | 100(2) |
| Crystal system | monoclinic |
| Space group | P2_1_/n |
| a/Å | 13.8143(3) |
| b/Å | 8.49817(19) |
| c/Å | 16.9667(4) |
| α/° | 90 |
| β/° | 100.564(2) |
| γ/° | 90 |
| Volume/Å^3^ | 1958.07(7) |
| Z | 4 |
| ρ_calc_g/cm^3^ | 1.461 |
| μ/mm^−1^ | 0.242 |
| F(000) | 888.0 |
| Crystal size/mm^3^ | 0.3 × 0.3 × 0.2 |
| Radiation | MoKα (λ = 0.71073) |
| 2Θ range for data collection/° | 5.38 to 54.998 |
| Index ranges | −17 ≤ h ≤ 17, −11 ≤ k ≤ 11, -22 ≤ l ≤ 22 |
| Reflections collected | 30718 |
| Independent reflections | 4479 [R_int_ = 0.0349, R_sigma_ = 0.0213] |
| Data/restraints/parameters | 4479/0/273 |
| Goodness-of-fit on F^2^ | 1.025 |
| Final R indexes [I>=2σ (I)] | R_1_ = 0.0342, wR_2_ = 0.0801 |
| Final R indexes [all data] | R_1_ = 0.0416, wR_2_ = 0.0843 |
| Largest diff. peak/hole / e Å^-3^ | 0.35/−0.24 |

| **Table S13. Crystal data and structure refinement for 5ac** | |
| --- | --- |
| Empirical formula | C_24_H_19_F_3_ |
| Formula weight | 364.39 |
| Temperature/K | 100(2) |
| Crystal system | triclinic |
| Space group | P-1 |
| a/Å | 10.3000(8) |
| b/Å | 10.5159(6) |
| c/Å | 17.5695(11) |
| α/° | 91.086(5) |
| β/° | 103.868(6) |
| γ/° | 103.835(6) |
| Volume/Å^3^ | 1788.1(2) |
| Z | 4 |
| ρ_calc_g/cm^3^ | 1.354 |
| μ/mm^−1^ | 0.099 |
| F(000) | 760.0 |
| Crystal size/mm^3^ | 0.3 × 0.3 × 0.25 |
| Radiation | MoKα (λ = 0.71073) |
| 2Θ range for data collection/° | 5.244 to 54.998 |
| Index ranges | −12 ≤ h ≤ 13, −13 ≤ k ≤ 13, -22 ≤ l ≤ 22 |
| Reflections collected | 24400 |
| Independent reflections | 8212 [R_int_ = 0.0335, R_sigma_ = 0.0374] |
| Data/restraints/parameters | 8212/0/491 |
| Goodness-of-fit on F^2^ | 1.024 |
| Final R indexes [I>=2σ (I)] | R_1_ = 0.0429, wR_2_ = 0.1026 |
| Final R indexes [all data] | R_1_ = 0.0563, wR_2_ = 0.1110 |
| Largest diff. peak/hole / e Å^-3^ | 0.40/−0.23 |

**DFT-calculations of Cations Aa, Ba**

**Cation Aa**

**E =** **−991.686419056 h, G^298^ = −991.495289 h, μ = 6.37 D**

Optimized geometry, A

N_at_	Atom	x	y	z

1	C	3.105852	0.474916	−0.786045

2	C	2.017621	0.577569	0.095499

3	C	1.750350	1.803724	0.732338

4	C	2.580328	2.898363	0.519488

5	C	3.663825	2.792330	−0.358056

6	C	3.915594	1.586111	−1.013499

7	C	1.074449	−0.561584	0.348104

8	O	1.387921	−1.013430	2.009227

9	C	−0.301215	−0.257969	0.241476

10	C	−1.492433	−0.059433	0.118295

11	C	−2.874425	0.209884	−0.010673

12	C	−3.333722	1.543052	−0.063259

13	C	−4.693844	1.799059	−0.192210

14	C	−5.602358	0.738801	−0.270964

15	C	−5.152795	−0.584698	−0.221656

16	C	−3.795693	−0.855793	−0.092312

17	C	1.430709	−1.951787	−0.254430

18	F	0.586124	−2.877058	0.238658

19	F	1.297084	−1.927662	−1.587001

20	F	2.674053	−2.337644	0.048906

21	H	−2.621369	2.358171	−0.001448

22	H	−3.437067	−1.878264	−0.053882

23	H	−5.047729	2.823606	−0.231668

24	H	−5.861741	−1.403241	−0.283976

25	H	−6.663068	0.944325	−0.371266

26	H	0.886593	1.915870	1.382594

27	H	3.331631	−0.445782	−1.304811

28	H	2.373520	3.833981	1.027549

29	H	4.748255	1.501563	−1.702949

30	H	4.305207	3.649761	−0.533955

31	H	1.418960	−0.220810	2.579074

32	H	0.694639	−1.605725	2.363280

Bond lengths, Å

**Summary of Natural Population Analysis:**

Natural Population

Natural

Atom No. Charge	Core	Valence	Rydberg	Total

-----------------------------------------------------------------------

C	1	−0.16647	1.99907	4.14707	0.02033	6.16647

C	2	−0.40497	1.99855	4.30657	0.09986	6.40497

C	3	−0.18926	1.99901	4.16174	0.02851	6.18926

C	4	−0.19023	1.99916	4.16987	0.02121	6.19023

C	5	−0.15357	1.99916	4.13575	0.01866	6.15357

C	6	−0.18925	1.99916	4.17076	0.01933	6.18925

C	7	1.00419	1.99865	2.61756	0.37961	4.99581

O	8	−0.73669	1.99977	6.71172	0.02520	8.73669

C	9	−0.24280	1.99846	4.18630	0.05804	6.24280

C	10	−0.34016	1.99854	3.14931	1.19232	6.34016

C	11	−0.18598	1.99890	4.16210	0.02498	6.18598

C	12	−0.13162	1.99907	4.11371	0.01884	6.13162

C	13	−0.20432	1.99915	4.18531	0.01986	6.20432

C	14	−0.15400	1.99916	4.13532	0.01951	6.15400

C	15	−0.20398	1.99915	4.18506	0.01978	6.20398

C	16	−0.12980	1.99906	4.11250	0.01823	6.12980

C	17	0.68658	1.99934	3.19756	0.11651	5.31342

F	18	−0.35083	1.99991	7.33707	0.01385	9.35083

F	19	−0.33316	1.99991	7.32571	0.00754	9.33316

F	20	−0.33668	1.99991	7.32648	0.01030	9.33668

H	21	0.22799	0.00000	0.77019	0.00182	0.77201

H	22	0.22823	0.00000	0.77012	0.00165	0.77177

H	23	0.22610	0.00000	0.77221	0.00169	0.77390

H	24	0.22605	0.00000	0.77228	0.00167	0.77395

H	25	0.22301	0.00000	0.77547	0.00152	0.77699

H	26	0.22640	0.00000	0.76829	0.00531	0.77360

H	27	0.23103	0.00000	0.76670	0.00227	0.76897

H	28	0.22884	0.00000	0.76957	0.00159	0.77116

H	29	0.22831	0.00000	0.77006	0.00163	0.77169

H	30	0.22575	0.00000	0.77271	0.00154	0.77425

H	31	0.55095	0.00000	0.44546	0.00359	0.44905

H	32	0.55095	0.00000	0.44508	0.00397	0.44905

=======================================================================

* Total *	0.42062	39.98308	100.43559	2.16071	142.57938

**Cation Ba**

**E=−915.274400001 h, G^298^= −915.107551 h, μ= 6.61 D**

Optimized geometry, A

N_at_	Atom	x	y	z

1	C	−3.741014	0.897388	0.000014

2	C	−2.829081	−0.188743	−0.000132

3	C	−3.312055	−1.522302	−0.000173

4	C	−4.671568	−1.752407	−0.000015

5	C	−5.560548	−0.673370	0.000172

6	C	−5.096683	0.645714	0.000179

7	C	−1.464121	0.051818	−0.000231

8	C	−0.253068	0.250220	−0.000325

9	C	1.080739	0.503676	−0.000098

10	H	−2.607359	−2.340232	−0.000355

11	H	−3.360522	1.907620	0.000010

12	H	−5.049414	−2.763917	−0.000031

13	H	−5.799354	1.465645	0.000305

14	H	−6.624809	−0.861985	0.000306

15	C	2.083385	−0.510475	−0.000039

16	C	1.687103	−1.874809	0.000080

17	C	3.469529	−0.202829	−0.000047

18	C	2.630523	−2.876349	0.000117

19	C	4.403502	−1.217135	−0.000005

20	C	3.991016	−2.551197	0.000073

21	H	0.636341	−2.122008	0.000150

22	H	3.807905	0.819736	−0.000134

23	H	2.320925	−3.910744	0.000214

24	H	5.456103	−0.976795	−0.000051

25	H	4.730006	−3.339877	0.000120

26	C	1.469962	1.990480	0.000031

27	F	2.199797	2.291506	−1.087486

28	F	0.396278	2.784383	0.000185

29	F	2.199976	2.291276	1.087507

HOMO -0.28264

LUMO -0.18473

**Summary of Natural Population Analysis:**

Natural Population

Natural -----------------------------------------------

Atom No	Charge	Core	Valence	Rydberg	Total

-----------------------------------------------------------------------

C	1	−0.09475	1.99910	4.07733	0.01832	6.09475

C	2	−0.15095	1.99864	4.13261	0.01969	6.15095

C	3	−0.09403	1.99910	4.07670	0.01822	6.09403

C	4	−0.20590	1.99915	4.18743	0.01933	6.20590

C	5	−0.09049	1.99917	4.07210	0.01922	6.09049

C	6	−0.20539	1.99915	4.18694	0.01931	6.20539

C	7	0.23086	1.99866	3.75696	0.01352	5.76914

C	8	−0.03627	1.99851	4.02446	0.01330	6.03627

C	9	0.04318	1.99874	3.93526	0.02282	5.95682

H	10	0.23476	0.00000	0.76378	0.00146	0.76524

H	11	0.23506	0.00000	0.76341	0.00153	0.76494

H	12	0.23361	0.00000	0.76477	0.00161	0.76639

H	13	0.23362	0.00000	0.76477	0.00160	0.76638

H	14	0.22808	0.00000	0.77049	0.00143	0.77192

C	15	−0.08977	1.99875	4.06830	0.02271	6.08977

C	16	−0.10279	1.99909	4.08590	0.01780	6.10279

C	17	−0.11006	1.99909	4.09296	0.01800	6.11006

C	18	−0.20050	1.99915	4.18212	0.01923	6.20050

C	19	−0.20128	1.99915	4.18264	0.01949	6.20128

C	20	−0.07861	1.99917	4.06040	0.01904	6.07861

H	21	0.23282	0.00000	0.76459	0.00259	0.76718

H	22	0.23287	0.00000	0.76506	0.00207	0.76713

H	23	0.23405	0.00000	0.76436	0.00159	0.76595

H	24	0.23427	0.00000	0.76414	0.00159	0.76573

H	25	0.22900	0.00000	0.76959	0.00141	0.77100

C	26	1.08188	1.99928	2.86598	0.05286	4.91812

F	27	−0.34220	1.99991	7.33251	0.00978	9.34220

F	28	−0.33851	1.99991	7.32833	0.01027	9.33851

F	29	−0.34221	1.99991	7.33252	0.00978	9.34221 ============================================================

* Total *	1.00034	37.98365	93.63643	0.37958	131.99966
